# Does Effectiveness of Adolescent Smoking-Cessation Intervention Endure Into Young Adulthood? 7-Year Follow-Up Results from a Group-Randomized Trial

**DOI:** 10.1371/journal.pone.0146459

**Published:** 2016-02-01

**Authors:** Arthur V. Peterson, Patrick M. Marek, Kathleen A. Kealey, Jonathan B. Bricker, Evette J. Ludman, Jaimee L. Heffner

**Affiliations:** 1 Division of Public Health Sciences, Fred Hutchinson Cancer Research Center, Seattle, WA, United States of America; 2 Department of Biostatistics, University of Washington, Seattle, WA, United States of America; 3 Department of Psychology, University of Washington, Seattle, WA, United States of America; 4 Group Health Research Institute, Seattle, WA, United States of America; Centre for Addiction and Mental Health, CANADA

## Abstract

**Background:**

The Hutchinson Study of High School Smoking was the first randomized trial to show effectiveness of a smoking cessation intervention on 6-months prolonged smoking abstinence at one year post-intervention in a large population-based sample of adolescent smokers. An important question remains: Do the positive effects from teen smoking cessation interventions seen at up to 12 months post-intervention *endure into young adulthood*? This study examines for the first time whether such positive early effects from teen smoking cessation intervention can endure into young adulthood in the absence of additional intervention.

**Methods:**

High school smokers (n = 2,151) were proactively recruited into the trial from fifty randomly selected Washington State high schools randomized to the experimental (Motivational Interviewing + Cognitive Behavioral Skills Training telephone counseling intervention) or control (no intervention) condition. These smokers were followed to 7 years post high school to ascertain rates of six-year prolonged smoking abstinence in young adulthood. All statistical tests are two-sided.

**Results:**

No evidence of intervention impact at seven years post high school was observed for the main endpoint of six-year prolonged abstinence, neither among all smokers (14.2% in the experimental condition vs. 13.1% in the control condition, difference = +1.1%, 95% confidence interval (CI) = -3.4 to 5.8, p = .61), nor among the subgroups of daily smokers and less-than-daily smokers, nor among other a priori subgroups. But, observed among males was some evidence of an intervention impact on two endpoints related to progress towards quitting: reduction in number of days smoked in the past month, and increase in the length of the longest quit attempt in the past year.

**Conclusions:**

There was no evidence from this trial among adolescent smokers that positive effectiveness of the proactive telephone intervention for smoking abstinence, observed previously at one year post-intervention, was sustained for the long-term into young adulthood. In light of the positive short-term effectiveness consistently observed from this and other trials for teen smokers, together with the lack of evidence from this study that such short-term impact can endure into young adulthood, sustained interventions that continue into young adulthood should be developed and tested for long-term impact.

**Trial Registration:**

ClinicalTrials.gov NCT00115882

## Introduction

Tobacco use is the leading cause of preventable disease and death in the United States, resulting in more than 480,000 premature deaths each year [1]. Smoking prevalence among teens and young adults continues to be a severe problem, putting young people at risk for a lifetime of smoking and its associated health problems, including premature death. Smoking prevalence in the 18–24 age group is 17.8%, second only to that of the 25–44 age group (20.1%) [2]. Finding ways to reduce smoking prevalence among teens and young adults is a high research priority.

The Hutchinson Study of High School Smoking (HS), the setting for this paper, was the first randomized trial to show the effectiveness of a smoking-cessation intervention in a large population-based sample of adolescent smokers in a non-medical population setting [3,4]. Motivational Interviewing plus Cognitive Behavioral Skills Training (MI+CBST) was proactively delivered by telephone to high school seniors who smoke. The cessation endpoint for evaluating intervention impact was six-month prolonged abstinence at one year post-intervention (one year after high school). Results showed that 21.8% of baseline smokers in the experimental condition, compared with 17.7% in the control condition, achieved 6-month prolonged smoking abstinence (difference = 4.0%, 95% confidence interval = -0.2 to 8.1, p = .06), and in particular among baseline daily smokers: 10.1% vs. 5.9% (difference = 4.1%, 95% CI = 0.8 to 7.1, p = .02). Not only was the intervention effective with respect to *impact on smoking cessation*, but also with respect to *reach*: the intervention’s proactive approach for recruitment engaged two-thirds of proactively-identified high school smokers in the telephone intervention [5].

Additional evidence for smoking-cessation effectiveness in teen smokers from interventions that incorporated Motivational Interviewing were reported by two other large randomized trials [6,7]. These two trials, conducted in family practice and pediatric clinics, reported statistically significant intervention effects for teen smokers with respect to 30-day smoking abstinence, at a follow-up of 1 and 2 years post intervention, and at 6 weeks and 3 months post intervention, respectively, for the Hollis et al. [6] trial and Pbert et al. [7] trials. Commonalities among these two trials and the HS trial included: (1) proactive identification and recruitment of adolescent smokers, (2) liberal definition of smokers that included less-than-daily smokers in addition to daily smokers, (3) personalized multicomponent interventions that included MI. All three trials were able to overcome methodological challenges inherent in teen cessation research [8,9], so that the design and execution of these trials were rigorous, thereby providing confidence in the findings that the interventions were indeed effective. The sum of the evidence from these three trials point to the short-term effectiveness for adolescent smokers of multicomponent smoking-cessation interventions that include MI.

Recent reviews and meta-analyses [10–14], which include the three trials mentioned above plus other (mostly smaller) ones, have concluded that interventions that include MI are effective for adolescent smoking cessation at 6–24 months post intervention.

But these promising findings about effectiveness of teen smoking cessation interventions have thus far been evaluated *only for the shorter-term*: at six months to one year post-intervention. An important question remains: Do the positive effects from teen smoking cessation interventions seen at 6–12 months post-intervention *endure into young adulthood*? We know of no teen smoking cessation intervention that has studied this important question about durability [11]. From a public health perspective the question of *durable long-term effectiveness* is important, because smoking cessation that lasts beyond the immediate post-intervention period is what leads to permanent smoking abstinence and contributes reductions in tobacco-related health problems, disease and premature death. Given the lack of any empirical evidence about long-term effectiveness, we undertook this long-term follow-up.

Here we report to what extent the positive smoking abstinence results of the HS trial’s proactive MI+CBST teen smoking cessation intervention observed at one year post intervention were maintained seven years later when study participants were young adults (~age 25).

## Methods

### Recruitment

The trial’s original population of 2,151 smokers, high school juniors in 50 Washington State public high schools, were identified via self-report using a study-administered baseline classroom survey. The baseline survey was designed both to proactively identify smokers for recruitment to the intervention in experimental schools, and to provide baseline data for the trial. (The baseline survey is available as supporting information—see S3 File.)

For trial management purposes, the 50 participating high schools joined the study in three groups over three years (referred to as Waves I, II, III). Baseline data collections took place in the period March–June in years 2002, 2003, and 2004, respectively, for Waves I, II, and III.

### Consent Procedures

Because most of the high school juniors were minors at the time of the baseline survey, we sought consent from parents/guardians first. About three weeks prior to the baseline survey, we mailed to the parents/guardians, on high school letterhead and co-signed by the high school principal and the study’s principal investigator, an informational letter. The outside of the envelope included instructions to the postal service to return undeliverable mail to the study office. The letter described the study and the survey, the saliva sample procedure, the confidential treatment of the data collected, and the voluntary nature of participation. The letter invited parents/guardians to call a toll-free telephone number with questions or to decline their high school junior’s participation in the survey. This passive consent procedure was used to minimize burden for parents/guardians, to provide a reliable and easy way of declining participation for those parents/guardians who didn’t want their teen to take part, and to maximize participation rates [15–19]. It was chosen for these older adolescents (16- to 17-year-olds), and approved by the Hutchinson Center’s Institutional Review Board, in accordance with the no-more-than-minimal risk nature of the questionnaire.

The baseline survey was conducted primarily in school classrooms, with in-class “clean-up” data collections two weeks later for those students who were absent from the initial data collection, and with mail and telephone follow-up for those students who were absent from both in-class data collections. Students were informed about the baseline survey in the following manner: Trained project data collectors presented a scripted explanation of procedures to students at the outset of each data collection class. The in-class explanation of procedures (script), similar in content to the parent letter, was designed to appeal to and be understood by high school students. In particular, the scripted in-class communication told students about the Hutchinson Center, the nature and rationale for the research study, what we hoped to learn from the survey, and a summary of the content of the questionnaire. We told students that their saliva sample could be tested for cotinine, explained that the test for cotinine is accurate and specific for nicotine, demonstrated how the saliva was to be collected (via cotton dental rolls), and assured them that their saliva would not be tested for anything other than cotinine. The script stated that participation in the survey activities was voluntary, and that they could decline all or part of the survey if they wished, and that we would keep their individual information confidential. It also invited students to ask questions, and stated that they could leave some questions blank, opt out of the survey and/or saliva sample, and stop at any time.

Staff administering the baseline survey informed prospective participants that “Some of you may be invited to participate in future research activities.” Also, the last page of the questionnaire requested contact information, and stated “We may invite you to take part in another activity” and “We’ll use the information below to contact you to see if you want to take part.”

Consent for the in-class survey and for the mailed baseline survey was documented by the return of an at-least-partially completed questionnaire and its entry into the study database. For telephone surveys, research project interviewers telephoned a parent/guardian first to explain the survey and its voluntary nature, and to seek verbal consent to talk to their student. If parental consent was provided, then the interviewer sought verbal consent from the student to survey him/her over the phone. Project interviewers documented all verbal consent conversations and consents in the study database. Students who indicated their decline or who returned a blank questionnaire were documented as nonparticipants for that survey. Students who requested no further contact from us were documented as nonparticipants for the survey *and* for the remainder of the trial; in accordance with their request we did not contact them further.

These data collection procedures, scripts, and the questionnaire were reviewed and approved in advance by the Hutchinson Center’s Institutional Review Board.

### Study Population

In the 50 schools, 93.1% (12,141 out of 13,042) of all enrolled juniors completed a baseline survey. Of the 901 (6.9%) who did not complete a baseline survey, 524 (4.0%) did not reply to the follow-up telephone or mailed survey after being absent during the in-class surveys, and 377 (2.9%) declined (by their own decision or that of their parents).

A total of 2175 smokers were proactively identified; 24 (1.1%) declined contact for future activities, leaving 2151 (98.9%) eligible for the trial. These trial participants were 47% female, and 25% nonwhite. Consistent with their being juniors in high school, most were 16 (30.5%) or 17 (62%) years old. (See [20] for more detail about characteristics the original cohort and their high schools.)

To capture the varied smoking patterns typical of adolescents, baseline smokers were defined as those who reported at least monthly smoking via a positive response to *any* of the following three baseline survey items: (1) How often do you currently smoke cigarettes? (Responded: once a month or more, but less than once a week; or once a week or more, but not daily; or at least daily); (2) Have you smoked one or more cigarettes in the last 30 days? (Responded: yes); and (3) When was the last time you smoked, or even tried, a cigarette? (Responded: 8–30 days ago, or 1–7 days ago, or earlier today). The steps for identifying and recruiting smokers were identical in the control and experimental conditions.

A sample of 743 baseline *nonsmokers* were also included in the trial (and in the experimental group for proactive intervention contact). The rationale for their inclusion was to maintain confidentiality of a participant’s smoking/non-smoking status, and in particular to ensure that our contacting individuals for participation in the trial would not reveal a participant’s smoking status. Nonsmokers received a different, shorter intervention than smokers, to enforce their choice to be a nonsmoker and to give them tips for supporting their friends who want to quit smoking.

The sample size of 50 high schools and 2151 teen smokers, with a minimum 80% design follow-up to Plus-7, was sufficiently large to accommodate intraclass correlation of outcome in achieving adequate statistical power: It provided 89% statistical power to detect a 6% absolute difference in smoking cessation percentage between the experimental and control groups.

### Experimental Design

**Fig 1:**
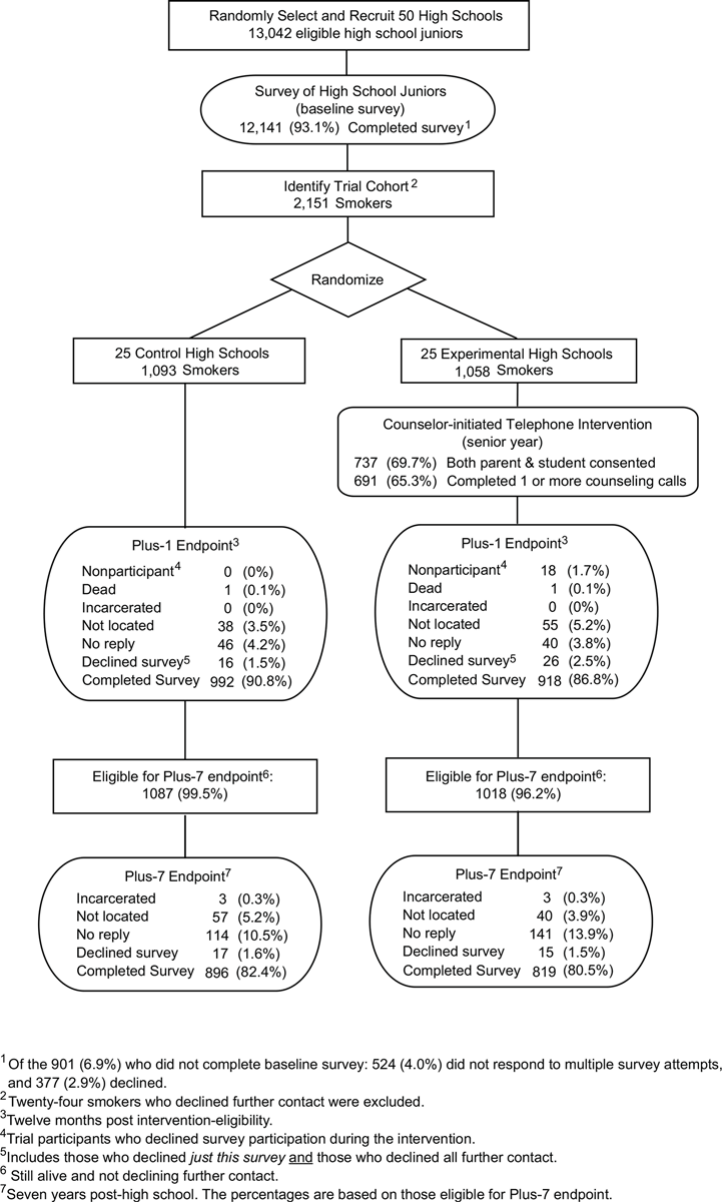
Follow-up flow chart for the HS trial including Plus-1 and Plus-7 follow-ups.

The HS trial used a two-arm, group-randomized design (Fig 1). The 50 participating high schools, recruited over a 3-year period, were randomly assigned either to the experimental condition (the MI+CBST proactive telephone intervention) or the control condition (no-intervention).

A matched-pair randomization was performed via a computerized coin flip for each of 25 pairs of high schools, using pair-matching of schools based on number of smokers, smoking prevalence, fraction of students eligible for free/reduced-priced meals, and average stage of readiness to quit, so that the experimental and control conditions were balanced on these criteria. Using this procedure, 25 high schools were assigned to the experimental condition, and 25 to the control condition. The original trial experimental design and randomized assignment were maintained and used for the Plus-7 follow-up evaluation of long-term impact reported in this paper.

The first outcome data for the trial were collected at one year post-intervention (Plus-1), and positive intervention impact at Plus 1 was reported earlier [3,4]. The second outcome data, collected at seven years post-intervention (Plus-7), are reported here.

The trial’s experimental design, procedures, and intervention were reviewed and approved at the start (IRB approval: March 1, 2001) and annually by the Hutchinson Center’s Institutional Review Board. In accordance with the ICMJE guideline that trials that began prior to July 1, 2005 be registered by Sept. 13, 2005, we registered this trial with ClinicalTrials.gov on June 26, 2005 (ClinicalTrials.gov identifier: NCT00115882). The authors confirm that all ongoing and related trials for this intervention are registered. The overall protocol for this trial and CONSORT checklist are available as supporting information: S1 File and S2 File.

## Intervention

### Telephone Counseling Component

The HS intervention was protocol-guided, personalized telephone counseling, conducted by trained counselors, and based on social cognitive theory [21]. Proactive in contacting and engaging smokers [3,4], the intervention was aimed at changing the theoretical processes of smoking and quitting expectancies, quitting self-efficacy, quitting outcome expectations, behavioral capability, and perceptions of social norms [22,23]. Various strategies were used to address barriers of proactive recruitment [4,5]. Because teen smokers in the population-based cohort did not self-select but were proactively recruited to the intervention, in order to engage those smokers initially not interested in quitting [24] the intervention used Motivational Interviewing (MI) and Cognitive Behavioral Skills Training (CBST). MI was used to engage eligible participants, develop rapport, and build and maintain motivation and self-efficacy for quitting [25,26]. CBST was included to build skills for quitting and prevent relapse [27,28]. (For more detail, see Section 1.6 of the intervention protocol in S4 File.) Up to ten proactive telephone calls, planned to be about 15 minutes long, were personalized to the participant’s readiness to quit and progress toward quitting [4]. For more detail, see Sections 6–9 of S4 File. Telephone counselors were trained, certified, and monitored, according to procedures described in [4].

#### Intervention Consent Procedures

The telephone counseling intervention took place in the period September–August in years 2003–2004, 2004–2005, and 2005–2006, respectively, for high school Waves I, II, and III.

The telephone counseling intervention, which targeted baseline self-reported smokers and a sample of baseline self-reported nonsmokers, used active parental consent procedures for those trial participants who were less than 18 years old. We mailed letters to the parents of these trial participants in the experimental arm. With the letter was a brochure that described the telephone counseling intervention. The letter emphasized both the intervention’s inclusion of both of both smokers and nonsmokers, so that the smoking status of the participants was not revealed, and the confidential nature of all intervention conversations. Parents were requested to provide their written consent, or written decline, on an enclosed consent form, and mail it back in an enclosed postage-paid envelope. For those parents whose consent form was not received by us, project staff telephoned them to invite their questions and to allow them to provide (or decline) verbal consent for their teen’s participation. Staff recorded and kept in the Project’s secure project database a computerized record of the consent conversation and its outcome.

For those trial participants who were already 18, parental consent was not required from parents. Nonetheless, we mailed these parents an informational letter about the intervention, and let them know that we would be contacting their teen to invite them to participate in the telephone counseling intervention.

All 18-year old trial participants in the experimental high schools, plus those under 18 whose parents had provided consent, were then mailed an informational letter and brochure describing the intervention. Within two weeks of this mailing, trained counselors telephoned the trial participant to invite participation in the intervention. The counselors conducted informed consent on the telephone using a documented scripted procedure, informing them of the nature of the telephone conversations, stating that participation was voluntary and confidential, and invited questions. Consent or decline, given verbally over the telephone, was documented both by voice recording and by the counselor’s recording of it into a secure project database of the consent conversation and its outcome. For more detail see S4 File.

All intervention consent procedures, letters, forms and scripts were reviewed and approved in advance by the Hutchinson Center’s Institutional Review Board.

## Follow-up

The first follow-up of the trial participants, reported previously [3], took place about one year post-intervention (Plus-1), when participants were around age 19. The focus of this paper is the second follow-up, which took place at seven years post-intervention (Plus-7), when trial participants were around age 25.

The Plus-7 data collection was conducted for trial participant baseline smokers by mailed questionnaire, with mail, telephone, and web follow-up of non-responders. In accordance with the scientific question (long-term impact on cessation), only baseline smokers were followed. The follow-up survey was minimal risk, using questionnaires that contained no sensitive items. Trial participant consent procedures, based on those that we had used successfully in a follow-up survey of young adults in a previous research project, are as follows.

We sent letters to trial participants that stated: the purpose of the survey, that responses would be kept strictly confidential, that participation was voluntary, and that the trial participant could decline the survey or leave any questions blank. A toll-free number was offered for asking questions about the survey. This information was also provided at subsequent telephone contact of non-responders.

Consent for the mailed survey was signified by the return of an at-least-partially completed questionnaire. For telephone and web surveys, participant’s consent was documented by us. Trial participants who returned a blank questionnaire or refused participation verbally or in writing were documented as survey decliners. Also, trial participants who requested no further contact from us were documented as nonparticipants for the survey *and* for the remainder of the trial, and in accordance with their request received no further contacts.

The Plus-7 survey procedure was multi-mode (mail, web, phone), according to the Tailored Design Method [29]. This survey, targeting all trial study participants in young adulthood, achieved a response rate of 69.9%, 11.1 percentage points below our 80% goal. To increase response, we administered a second survey that targeted the nonrespondents of the first survey, among whom the second survey achieved a response rate of 42.9%. This provided an overall response rate from the two surveys of 81.5%, surpassing our 80% goal. Both the first survey and the second survey are available as supporting information: See S6 File and S7 File.

Survey administration at Plus-7 took place for the main survey (TOPS) during August–July in years 2010–2011, 2011–2012, and 2012–2013, respectively, for high school Waves I, II, and III; and for the second survey (SNAP) during July 2013 –March 2014 for Waves I and II, and during September 2013 –March 2014 for Wave III.

### Main Endpoint

The main endpoint at Plus-7 for evaluating *sustained* effectiveness of the intervention into young adulthood was *six-years prolonged abstinence*: abstinence that began shortly after the intervention and *was sustained for six years*, to the Plus-7 data collection at age ~25. This main endpoint captured (1) the main impact intended by the intervention [30–32]: quitting at or near the time of the intervention’s implementation (eg, [33–37]), *and* (2) a meaningfully long (six year) period of prolonged abstinence.

Moreover, six years has been shown to be long enough to capture almost all relapse; although few studies have done longer follow-up, those that do have showed low relapse rates after six years. Krall et al. [38] showed a relapse rate of less than 1% after 10 years of abstinence; Wetter et al. [39] showed a relapse rate of 2.1% among those abstinent after five years. Hawkins et al. [40] showed annual relapse rates of between 0 and 1.4% for lengths of abstinence beyond six years out to 10 years. All of these findings are consistent with the familiar relapse curve that flattens out to nearly horizontal after 5–10 years. Accordingly, six-year prolonged abstinence is a good predictor of permanent abstinence; long-term studies of relapse show very low levels of relapse among former smokers after having been continuously abstinent for such a long period of time [39–41].

The trial’s Plus-7 endpoint of prolonged six-year abstinence was defined conservatively as consistent reporting of abstinence (bolded, below) on three abstinence items. 1) When was the last time you smoked, or even tried, a cigarette? (Response choices: I have never smoked, or even tried, a cigarette; **over 6 years ago;** over 3 years ago; over 12 months ago; between 6 months and 12 months ago; between 3 months and 6 months ago; between 1 month and 3 months ago; 8–30 days ago; 1–7 days ago; earlier today). 2) How often do you currently smoke cigarettes? (Response choices: **not at all;** less than once a month; once a month or more, but less than once a week; once a week or more, but not daily; at least daily). 3) Think about the last 30 days. On how many of the last 30 days have you smoked one or more cigarette? (Response choices: Every day; 20–29 days; 10–19 days; 2–9 days; 2–4 days; 1 day; **0 days**). Such an endpoint conservatively defined as consistent reporting on multiple survey items was used in lieu of biochemical validation to support validity of self-report [42–44].

### Secondary Endpoints

To further evaluate a range of possible long-term outcomes, two secondary investigations were undertaken:

1. Evaluation of quitting that started well *after* the intervention period, and was sustained continuously thereafter. Such a sleeper effect is a conceivable result from a motivational interviewing and skills building intervention: intervention-induced motivations to consider quitting could persist, and result later in quitting when life circumstances change and certain motivations become more pertinent.
  Three sleeper-effect endpoints were investigated: in order of length and importance–3-year prolonged abstinence, 1-year prolonged abstinence, and 6-months prolonged abstinence.
2. Evaluation of intervention impact on *sustained* progress toward quitting: progress from baseline to Plus-1, followed by *additional* progress from Plus-1 to Plus-7. The fact that identical items appeared on both the Plus-1 questionnaire (See S5 File) and the Plus-7 questionnaires (S6 File and S7 File) made possible these measures of progress. Three measures of sustained progress were evaluated: (a) sustained reduction in level of smoking: ie, reductions in smoking frequency number of cigarettes smoked, and number of days smoked in the last month. (Tindle and Shiffman [45] found that conversion from daily smoking to less-than-daily smoking is a predictor of successful quitting.); (b) sustained increase in length of quit attempts; and (c) sustained positive changes in readiness to change, both in Contemplation Ladder [46] and in stage of change (from the Transtheoretical Model [47]).

### Statistical Analyses

In accordance with the group randomized design of this trial, the randomization-based permutation method of analysis [48–50] was used for the Plus-7 evaluation implemented by a local coding (at http://dx.doi.org/10.5281/zenodo.44485) of the permutation method as described in [49]. For all smokers, for daily smokers, and for less-than-daily smokers separately, the permutation method was used to evaluate intervention effectiveness and provide two-sided *p*-values and 95% confidence intervals. This method is appropriate for this study because its basis for inference is the experimental design: the group randomization of the trial’s high schools. Importantly, permutation methods do not rely on *any* modeling or distributional assumptions. By permuting high schools, intraclass correlation arising from extra-binomial heterogeneity between high schools, and from social influences among former high school classmates that persist beyond high school, is accommodated [48–50]. Further, because a high level of follow-up (>80%) was part of the trial’s design, and to avoid assumptions about imputation of missing data, our analysis plan specified a complete-case analysis: only those trial participants followed to Plus 7 were included in the analyses.

The random assignment of high schools resulted in good balance of baseline characteristics of smoker trial participants between the experimental and control conditions, with the exception of daily smoking [20]. Because we have had relatively little attrition from our original cohort due to death or decline of follow-up, baseline characteristics were similarly balanced for the surviving cohort who responded at Plus-7. Despite the random assignment, the experimental group included a somewhat higher percentage of baseline daily smokers than the control group (39.9% vs 34.5%, *p* = .02). The imbalance in daily smoking at baseline was accounted for by use of a stratified analysis, as described in [3].

We considered that results may differ between female and male smokers, as they did at the first follow-up [3]. Therefore, in accordance with our a priori analysis plan, we report results by gender, and by baseline subgroups: quit attempts in the past year (none; one or more), and stage of change (precontemplation; contemplation; preparation).

The datasets in the analyses of this paper appear as supporting information in S9 File.

## Results

Results for intervention participation have been reported elsewhere [3,4].

### Follow-up

Of the original 2,151 smokers in the HS trial cohort, 46 had died or declined further follow-up prior to the start of the Plus-7 data collection, leaving 2,105 eligible for Plus-7 follow-up. Of these, 81.5% were successfully located and surveyed (see Fig 1). Plus-7 survey respondents were 49.0% female, and 21.9% nonwhite or Hispanic; average age at time of survey was 25.83 and median age was 26.

### Intervention Impact at 7-year Follow-up

Shown in Table 1 are the results for intervention impact at the Plus-7 follow-up for the main endpoint, six-year prolonged abstinence. As shown, there is no evidence of an intervention impact at 7-year follow-up for the entire cohort of baseline smokers (Δ = 1.1%; CI = -3.4, 5.8; *p* = .61), nor for the subsets baseline daily smokers (Δ = -0.9%; CI = -4.2, 2.3; *p* = .56) and baseline less-than-daily (LTD) smokers (Δ = 2.3%; CI = -4.6, 9.5; *p* = .51).

**Table 1:** Percent of teen smokers who achieved six-year prolonged smoking abstinence at Plus-7.

Baseline Group	Females				Males				All participants			
	Control	Experi-mental	Δ*, % (95% CI)	*P*†	Control	Experi-mental	Δ*, % (95% CI)	*P*	Control	Experi-mental	Δ*, % (95% CI)	*P*
All smokers (*n* = 822, 854, 1676) ††	14.8	16.4	1.6 (-4.8, 8.9)	.65	11.6	12.2	0.6 (-4.5, 5.2)	.80	13.1	14.2	1.1 (-3.4, 5.8)	.61
Daily smokers (*n* = 318, 303, 621)	5.9	4.2	-1.5 (-7.1, 4.0)	.56	7.0	6.8	0.01 (-5.7, 5.7)	1.0	6.4	5.5	-0.9 (-4.2, 2.3)	.56
Less-than-daily smokers (*n* = 504, 551, 1055)	20.1	23.7	3.6 (-6.5, 14.7)	.50	14.2	14.9	0.9 (-5.7, 7.0)	.77	17.0	19.3	2.3 (-4.6, 9.5)	.51

* Δ = difference: percent in experimental high schools *minus* percent in control high schools. (However, whenever some high schools had no baseline smokers in the subgroup of interest, neither these high schools, *nor their pairs*, are included in the (matched-pair) permutation test, or in the computation of Δ reported here.)

CI = confidence interval

† *P* values (two-sided) were calculated using the group-randomized exact permutation test.

†† *n* represents the number of valid responses for females, males, and all, respectively.

Nor do the results for the sleeper-effect endpoints, the shorter-duration (3-year, 1-year, and 6-months) prolonged abstinence endpoints (Table 2), show any evidence of intervention impact at Plus-7.

**Table 2:** Percent of teen smokers who achieved 3-year, 1-year, and 6-months prolonged abstinence at Plus-7.

Abstinence outcome	Females				Males				All participants			
	Control	Experi-mental	Δ*, % (95% CI)	*P*†	Control	Experi-mental	Δ*, % (95% CI)	*P*†	Control	Experi-mental	Δ*, % (95% CI)	*P*†
***All smokers***												
3-yr smoking abstinence, % (*n* = 822, 854, 1676) ††	27.9	25.6	-2.2 (-9.8, 5.9)	.60	18.9	21.5	2.6 (-3.7, 7.8)	.37	23.2	23.3	0.1 (-4.8, 4.9)	.97
1-yr smoking abstinence, % (*n* = 822, 854, 1676)	37.2	35.4	-1.8 (-11.3, 7.9)	.71	29.0	30.2	1.3 (-5.1, 6.4)	.65	32.9	32.5	-0.3 (-5.3, 4.3)	.89
6-mo smoking abstinence, % (*n* = 822, 854, 1676)	43.0	42.3	-0.6 (-10.4, 9.4)	.90	35.4	34.9	-0.5 (-6.6, 4.4)	.84	39.1	38.4	-0.7 (-6.2, 4.2)	.78
***Daily smokers***												
3-yr smoking abstinence, % (*n* = 318, 303, 621)	17.0	11.5	-5.2 (-13.4, 2.9)	.19	10.6	11.8	1.6 (-6.3, 8.9)	.67	13.9	11.7	-2.2 (-6.8, 2.2)	.30
1-yr smoking abstinence, % (*n* = 318, 303, 621)	25.5	20.0	-5.6 (-17.0, 6.5)	.34	16.9	17.4	1.0 (-10.3, 10.7)	.84	21.4	18.7	-2.6 (-9.7, 4.2)	.43
6-mo smoking abstinence, % (*n* = 318, 303, 621)	32.0	24.8	-7.2 (-19.6, 6.4)	.28	23.2	20.5	-2.1 (-14.0, 7.9)	.70	27.8	22.7	-5.1 (-13.3, 2.4)	.17
***Less-than-daily smokers***												
3-yr smoking abstinence, % (*n* = 504, 551, 1055)	34.3	33.9	-0.4 (-10.3, 10.0)	.94	23.9	26.6	3.1 (-5.5, 10.4)	.44	28.7	30.2	1.5 (-5.3, 8.2)	.66
1-yr smoking abstinence, % (*n* = 504, 551, 1055)	44.0	44.5	0.5 (-12.3, 13.1)	.94	35.8	36.9	1.4 (-6.7, 8.6)	.70	39.6	40.7	1.1 (-5.2, 7.0)	.72
6-mo smoking abstinence, % (*n* = 504, 551, 1055)	49.6	53.0	3.3 (-7.5, 14.1)	.52	42.3	42.3	0.3 (-6.4, 6.2)	.92	45.7	47.6	1.9 (-3.6, 7.1)	.46

* Δ = difference: percent in experimental high schools *minus* percent in control high schools. (However, whenever some high schools had no baseline smokers in the subgroup of interest, neither these high schools, *nor their pairs*, are included in the (matched-pair) permutation test, or in the computation of Δ reported here.)

CI = confidence interval

† *P* values (two-sided) were calculated using the group-randomized exact permutation test.

†† *n* represents the number of valid responses for females, males, and all, respectively.

Of note from Table 2, apart from the lack of evidence of intervention impact, are the large fractions of baseline smokers, *both* experimentals and controls, who achieved some duration of prolonged abstinence at Plus-7. In particular, among all baseline smokers, 39.1% of the control cohort and 38.4% of the experimental cohort achieved a short-duration 6-*month* prolonged abstinence at Plus-7. These fractions that achieved 6-months prolonged abstinence at Plus-7 are about double those at Plus-1 (39.1% vs. 17.8%, and 38.4% vs. 21.8%, among the control and experimental cohorts respectively). Also, the fractions of baseline smokers who achieved 1-year prolonged abstinence (32.9% and 32.5% among controls and experimentals, respectively) and 3-year prolonged abstinence (23.2 and 23.3%) are also substantial.

Table 3 shows the intervention impact for five different subgroups defined by two variables: (1) whether or not at least one quit attempt within the last year at baseline (no quit attempts, one or more quit attempts), and (2) baseline stage of change (precontemplation, contemplation, preparation). From Table 3 we see no evidence of intervention impact on 6-year prolonged abstinence at Plus-7 for any of the five subgroups. Similarly (not shown), in these subgroups there was no evidence of intervention impact at Plus-7 for the shorter-duration (sleeper-effect) prolonged abstinence endpoints.

**Table 3:** Impact on main endpoint at Plus-7, by a priori subgroups: Percent of teen smokers achieving six-year prolonged smoking abstinence.

Baseline subgroup	Females				Males				All participants			
	Control	Experi-mental	Δ*, % (95% CI)	*P*†	Control	Experi-mental	Δ*, % (95% CI)	*P*†	Control	Experi-mental	Δ*, % (95% CI)	*P*†
*Quit attempt in past year*												
None (n = 340, 415, 755)††	14.9	15.2	0.3 (-10.2, 12.5)	.95	9.0	11.6	2.6 (-4.4, 8.3)	.42	11.6	13.5	2.0 (-3.8, 7.8)	.47
One or more (n = 379, 320, 699)	10.3	12.7	2.4 (-3.7, 9.4)	.43	10.5	5.2	-5.3 (-11.3, 0.8)	.09	10.1	9.2	-1.0 (-5.3, 3.5)	.64
*Stage of change*												
Precontemplation (n = 440, 465, 905)	12.4	11.7	-0.7 (-7.5, 7.8)	.85	9.3	9.6	0.3 (-5.1, 5.7)	.90	10.6	10.4	-0.2 (-4.6, 4.7)	.95
Contemplation (n = 142, 120, 262)	10.3	10.5	0.2 (-11.1, 10.2)	.97	6.5	3.9	-2.6 (-12.8, 8.8)	.51	8.6	8.6	0.0 (-8.3, 8.1)	.99
Preparation (n = 142, 143, 285)	16.3	20.7	4.5 (-11.8, 23.9)	.59	10.0	11.8	1.8 (-13.8, 16.9)	.83	12.3	15.2	2.9 (-8.6, 14.7)	.61

* Δ = difference: percent in experimental high schools *minus* percent in control high schools. (However, whenever some high schools had no baseline smokers in the subgroup of interest, neither these high schools, *nor their pairs*, are included in the (matched-pair) permutation test, or in the computation of Δ reported here.)

CI = confidence interval

† *P* values (two-sided) were calculated using the group-randomized exact permutation test.

†† *n* represents the number of valid responses for females, males, and all, respectively.

Did the intervention have any effect on the six measures of sustained progress toward quitting? As show in Tables A1, A2, and A3 in Appendix A, among females, there was no evidence that the intervention helped them make sustained progress toward smoking cessation. In contrast, among the males, there was evidence that a higher percentage of experimental participants (32.8%) than control participants (24.4%) reduced, consistently from baseline to Plus-1 to Plus-7, the number of days smoked in the last month: Δ = 8.4%; CI = 1.3, 14.7; *p* = .024. Also among the males, there was evidence that a higher fraction of experimental participants (23.8%) than control participants (18.0%) increased, consistently from baseline to Plus-1 to Plus-7, the length of their longest quit attempt: Δ = 5.8%; CI = 0.02, 11.2; *p* = .049. For the other four measures of sustained progress from baseline to Plus-1 to Plus-7 among the males, there was mild evidence (p-values ranging from .15 to .21) that more experimental participants than control participants showed progress. In sum, among the males, there was a consistent finding of mild to strong evidence, across all six progress-to-quitting variables, that the intervention had a positive impact on sustained progress toward quitting.

### Contributions to the Decay of Intervention Effect from Plus-1 to Plus-7

The HS trial’s long-term follow-up shows that the positive intervention effect on smoking abstinence that had been observed at Plus-1 was *not* maintained at Plus-7. Possible contributors to this result are: (1) substantial smoking relapse by Plus-7 among those six-months abstinent at Plus-1, in *both* the control and experimental groups; (2) *differential* (C. vs. E.) relapse between Plus-1 and Plus-7; (3) delayed abstinence at Plus-7 among those *not* six-months abstinent at Plus-1; and/or (4) *differential* (C. vs. E.) delayed abstinence at Plus-7. The results for these four possibilities are shown in Table 4.

**Table 4:** Contributions to the long-term (Plus-7) decay of short-term (Plus-1) intervention impact: (1) Percent (and number of) smoking relapses at Plus-7 among those abstinent at Plus-1; (2) Percent (and number of) new abstinences at Plus-7 among those *not* abstinent at Plus-1.

	Females		Males		All participants	
	Control	Experimental	Control	Experimental	Control	Experimental
**A. Smoking relapse at Plus-7 among those six-months-abstinent at Plus-1**						
Among all baseline smokers (*n* = 147, 155, 302)	59.2 (42)	59.2 (45)	67.9 (53)	68.8 (53)	63.8 (95)	64.1 (98)
Among daily smokers at baseline (*n* = 19, 25, 44)	77.8 (7)	90.0 (9)	50.0 (4)	70.6 (12)	64.7 (11)	77.8 (21)
Among less-than-daily smokers at baseline (*n* = 126, 125, 251)	58.3 (35)	54.5 (36)	70.1 (47)	70.7 (41)	64.6 (82)	62.1 (77)
**B. Delayed six-year abstinence at Plus-7 among those *not* six-months-abstinent at Plus-1**						
Among all baseline smokers (*n* = 633, 630, 1,263)	9.4 (32)	7.8 (23)	7.7 (26)	6.5 (19)	8.6 (58)	7.2 (42)
Among daily smokers at baseline (*n* = 276, 250, 526)	5.0 (7)	2.9 (4)	5.0 (6)	3.1 (4)	5.0 (13)	3.0 (8)
Among less-than-daily smokers at baseline (*n* = 346, 369, 715)	12.8 (25)	12.6 (19)	9.3 (20)	9.7 (15)	11.0 (45)	11.1 (34)

The first three lines of Table 4 show, among those abstinent at Plus-1, the percent relapse at Plus-7 both for all baseline smokers (line 1) and for the subgroups baseline *daily* smokers (line 2) and baseline *less-than-daily* smokers (line 3). We see that the percent smoking relapse that occurred between Plus-1 and Plus-7 was large, almost 65% overall, and that relapse was virtually identical for the control and experimental conditions: 64.6% (control) and 64.9% (experimental). In particular, there was no evidence (p = .75, not shown) for baseline daily smokers, the group for which the evidence was strongest of an intervention effect at Plus-1, that the percent relapse between Plus-1 and Plus-7 was greater for experimentals (77.8%) than for controls (64.7%).

The results for delayed six-year prolonged abstinence at Plus-7 are show in lines 4, 5 and 6 of Table 4. We see that among those baseline smokers not abstinent at Plus-1 the percent of delayed abstinence at Plus-7 was very low: 7–9% overall, and not differing much by experimental condition: 8.6% for controls; 7.2% for experimentals.

In sum, we see no six-year sustained abstinence as a result of the high school intervention. Instead, there was a high percent of relapse (65%) that occurred throughout young adulthood, between Plus-1 and Plus-7, in both the control and experimental conditions. With respect to delayed abstinence, this was small in both the control and experimental groups, with no evidence of an intervention impact.

Finally, because it seems unlikely that delayed abstinence could be attributed to the MI intervention, which occurred one year prior to Plus-1, we explored whether or not there is evidence from the HS trial for a “consistent intervention effect” at *both* Plus-1 and Plus-7, which excludes delayed abstinence as a positive endpoint. We did this by considering the restricted outcome “Plus-1 abstinence followed by Plus-7 abstinence” in the subset of the study population (n = 1,584) for whom data were available at both the Plus-1 and Plus-7 follow-ups. Results (not shown) showed no evidence for an intervention effect overall (p = .32), either among daily smokers (p = .79) or among less-than-daily smokers (p = .29), or among females (.26) or among males (.77). This result underscores that the high degree of relapse from Plus-1 to Plus-7, and not differential (experimental vs. control) delayed abstinence at Plus-7, was responsible for the decay of intervention impact from Plus-1 to Plus-7.

## Discussion

The HS trial was the first adolescent smoking cessation trial to report statistically significant increases in 6-month prolonged abstinence, as measured 12-months-post intervention, in a large population of adolescent smokers in a nonmedical setting [3]. It is also the first to follow the study cohort into young adulthood to ascertain long-term effectiveness of the intervention.

The results of the Plus-7 follow-up *show no evidence of intervention effectiveness on six-years prolonged abstinence as measured at seven years post-intervention*. That is, the intervention impact on smoking cessation observed at Plus-1 was not sustained at Plus-7.

The effectiveness of the intervention demonstrated earlier at Plus-1 [3] was based on a strong definition of quitting: 6-months prolonged abstinence measured one year post-intervention, a high standard for short-term cessation that has been recommended as a preferred measure of abstinence by both the SRNT workshop on measures of abstinence in clinical trials [43], and the Russell Standards [51]. Because 6-months abstinence is a substantial period to be without cigarettes, even a single puff, and thus a strong measure of cessation at Plus-1, the results reported here cannot be attributed to use of a weak measure of cessation at Plus-1.

Several elements of this trial’s design and execution contributed to the validity of these findings: (1) large number of high schools (50), and large numbers of trial participant smokers at both Plus-1 (2,151) and Plus-7 (2,105), (2) experimental conditions (intervention, control) assigned via (group) randomization, (3) data collection and endpoint ascertainment conducted per protocol, identical in control and experimental cohorts, (4) tracking, follow-up and contact procedures that resulted in 88.8% response at Plus-1 (1,910 of 2,151 eligibles) and 81.5% response at Plus-7 (1,715 of 2,105 eligibles), and (5) conservative definition of cessation at seven-years post-intervention: ie, six-year prolonged abstinence, measured by consistent reports among multiple survey items.

A high degree of generalizability of these results to the adolescent smoker population is suggested by (1) the high level (93%) of participation in the baseline survey used to identify smokers, (2) liberal definition of smoker: at least monthly, as determined from *any* of three survey items, and (3) high degree (81.5%) of endpoint ascertainment at the Plus-7 follow-up in young adulthood. A limitation of this study is the results do not pertain to the 18.5% who were not successfully followed.

The public-health significance of these findings is substantial: whereas there was evidence that the MI + CBST smoking-cessation intervention delivered during the teen years to proactively identified and recruited teens can effectively promote cessation *at one year post high school*, the evidence reported here for the HS trial’s seven-year follow-up is that the intervention effectiveness was not sustained into young adulthood. *This finding underscores the unmet need for teen smoking cessation interventions that can affect long-term abstinence into and beyond young adulthood*. New intervention approaches for teen smokers that extend past high school need to be conceptualized, developed, and rigorously tested. Also, the processes by which young adults can relapse, even after a 6-month prolonged abstinence, need to be better understood.

In sum, to what extent was this MI-plus-CBST smoking cessation intervention for teen smokers effective? The evidence says that the intervention succeeded in effecting six-months prolonged abstinence, at one year post intervention, a notable achievement given this strong abstinence measure. Also, at the follow-up seven years post intervention it succeeded among males in effecting *progress* toward quitting. Two possible processes may be at work: either (1) that the progress among males is real, and would possibly result in greater observed cessation eventually among the experimental male smokers, if they were followed even longer; or (2) that some male smokers were influenced by the intervention to reduce their smoking to the level of intermittent or social smoking, but without a clear commitment to quitting completely.

Also, it is noteworthy that delayed abstinence at Plus-7—ie, six-year abstinence among those who were not abstinent at Plus-1—was very low: Even for less-than-daily smokers at baseline who were not abstinent at Plus-1, only 11.0% managed a long-term (six-years) prolonged abstinence on their own in young adulthood; for daily smokers at baseline, only 4% did so. These figures reinforce the need for new interventions that can help teen and young adult smokers to quit.

Where does teen smoking cessation go from here? What kind of intervention could be hypothesized to have some promise to last not only for the short term, but also for long-term, into young adulthood and beyond?

Several challenges would need to be overcome. First, smoking cessation is recognized as a complex and variable process [52], with the risk of relapse very high, even after an initial quit lasting six months or more. Moreover, the risk of relapse appears to be highly dependent on changing life circumstances and stressful life events [38,39,53]. Second, successful long-term smoking cessation for teens would need to address and overcome the special developmental processes of young adulthood, characterized by multiple and unpredictable life changes stimulated by young adults’ exploration and adaptation to new opportunities, difficulties and challenges [54]. Moreover, recent theories of life-span development (eg, [55]) posit that motivations play a key role in the individual’s selection and pursuit of goals in response to changes in life-course circumstances (opportunities and constraints, difficulties and worries, success and failures). According to such theories, the individual is an active agent, active in his/her development, adapting to life changes. These processes of young adulthood are rich with changes in motivations, attitudes, interests and life circumstances, which can influence smoking behavior, both in the direction of abstinence and in the direction of relapse.

To address these challenges, we recommend development and evaluation of a long-term MI+CBST intervention that starts in the adolescent years *and that is sustained* throughout young adulthood, in parallel with (and in engagement with) the processes of young adult smoking cessation and young adult development. (Such an approach is consistent with the chronic disease model for treating tobacco use and dependence [24].)

In our judgment, multiple factors lend credence to the potential of such a sustained MI+CBST-intervention process: (1) MI’s client-centered deferential approach, (2) The flexibility of an MI approach to adjust to client changes: new issues and circumstances, and new motivations, (3) The demonstrated success of proactive MI+CBST in overcoming barriers to recruitment and retention of teen smokers [4], (4) Older teens’ willingness to participate in counseling calls; indeed, due to participant interest, the average call length during the HS trial was twice what was planned, (5) The in-kind resource of rapport and trust established early in the intervention via MI, between client and counselor, that provides an already-established strong base for productive interactions in a long-term intervention, (6) The inclusion of training in skills for quitting and preventing relapse, so that once motivated, young adults know more about how to quit and are confident in their ability to do so, (7) The demonstrated short-term effectiveness of MI+CBST for teen smokers, (8) Evidence from this trial that at Plus-7 there may be an intervention impact, among males at least, on intermediate progress endpoints (cutting down on smoking, longer length of quit attempts, and increase in readiness to quit), and (9) The spontaneous quitting success, among both control and experimental cohorts, observed at Plus 7, suggesting that young adults want to quit but need help.

The short-term success of MI+CBST intervention in effecting smoking cessation in teens suggests that extending a similar intervention into young adulthood may decrease relapse among those who quit early in the intervention, and among those who didn’t quit early increase cessation later in young adulthood, when circumstances change in response to new life circumstances and motivations.

We need to learn more also about the process of smoking cessation and relapse during adolescence and young adulthood, building on Hawkins et al. [40], Wetter et al. [39], Gilpin et al. [56], and Fernandez et al. [57]. The recent Cochrane report [11] on youth smoking cessation stated: “As a complementary measure, long-term prospective studies of the natural smoking history of those making quit attempts in adolescence are needed.” Also, Hawkins et al. [40] noted that “a better understanding of smoking abstinence and relapse is needed to inform public health interventions to increase sustainable smoking cessation.” Such understanding would of course be especially crucial for the period of young adulthood, when smoking prevalence is high and the processes of cessation and relapse are especially complex, depending on a host of psychological, social, and external factors related to long-term relapse.

The most recent Cochrane review [11] stated: “There continues to be a need for well-designed adequately powered randomized controlled trials of interventions for [youth] smokers.” The results of the HS trial reported here underscore two needs, one concerning follow-up and one concerning intervention. With regard to follow-up, this trial underscores the need for long-term follow-up of those trials of teen smoking cessation that succeed in finding a short-term intervention impact, because a finding in the short-term of an intervention impact does not at all suggest that intervention impact would persist for the long-term. With regard to intervention, youth cessation programs are needed that can effect quitting that lasts throughout and beyond young adulthood. An intervention that incorporates MI and cognitive skills building, with delivery sustained into young adulthood, is recommended for consideration. Research is now needed to test the effectiveness of proactively-delivered interventions—using the telephone or new technologies—that span adolescence into young adulthood.

## Conclusion

There was no evidence from this trial among adolescent smokers that the short-term effectiveness observed earlier from an MI-based proactive telephone intervention was sustained into young adulthood. In light of the positive short-term effectiveness consistently observed from this and other trials for teen smokers, together with the lack of evidence that such short-term impact can endure into young adulthood, sustained teen interventions that are sustained into young adulthood should be developed and tested for long-term impact.

## Supporting Information

S1 File Overall Trial Protocol. (PDF)

S2 File CONSORT Checklist. (PDF)

S3 File Baseline Questionnaire. (PDF)

S4 File Intervention Protocol (“Matchbreaker”). (PDF)

S5 File Plus-1 Outcome Questionnaire (“Wrap-Up Survey”). (PDF)

S6 File Plus-7 First Survey (“TOPS”). (PDF)

S7 File Plus-7 Second Survey (“SNAP”). (PDF)

S8 File Appendix. Intervention Impact on Progress Endpoints.
(DOCX)

S9 File Data sets. (ZIP)
