# Supplementary material for: Does Effectiveness of Adolescent Smoking-Cessation Intervention Endure Into Young Adulthood? 7-Year Follow-Up Results from a Group-Randomized Trial
Source: PLoS One. 2016 Feb 1;11(2):e0146459. doi: 10.1371/journal.pone.0146459 (PMC4734743; doi:10.1371/journal.pone.0146459)
Supplement: S1 File — (PDF) [file pone.0146459.s001.pdf]

# Overview of Trial Protocols and Procedures for the Hutchinson Study of High School Smoking ("HS Study")

## A. School District/High School Selection and Recruitment

1. School District/High School Eligibility Criteria and Selection:

   a) Washington State public school districts with at least one high school.
   b) High school had to have at least 100, and less than 501, seniors – as recorded in the 2000-2001 Washington State Office of Superintendent of Public Instruction database.
   c) High school had to be within 200 miles of the Fred Hutchinson Cancer Research Center (FHCRC).
   d) Selected only one high school per school district.

2. School District/High School Recruitment:

   First, the principal investigator called the school district superintendent to inform her/him about the study and the opportunity for the selected districts and high schools to collaborate with the FHCRC and to invite him/her to come to a meeting at the FHCRC to further explore this possibility. Then, the principal investigator made a similar call to the high school principal. These calls were followed by a formal Letter of Invitation to the Meeting. At the meeting, the principal investigator and study staff presented the study, answered all questions from attendees and worked with attendees to prepare a plan for deciding about their interest in joining the study. The study leadership and staff supported these plans until a district/school decided they were ready to join. Then, the study mailed a formal letter of invitation to join the study to the superintendent (cc to the principal), and asked for a formal letter of commitment signed by the superintendent and high school principal. Upon receipt of the district's letter of commitment, the study mailed the superintendent (cc to the principal) a formal letter of welcome. To aid research logistics, schools/districts were recruited in three waves. Wave I (recruited in Fall 2001) had 14 schools, Wave II (recruited in Fall 2002) had 20 schools, and Wave III (recruited in Fall 2003) had 16 schools.

   These procedures worked well, achieving an overall recruitment rate of 84.7% (50 schools recruited/59 schools contacted).

## B. Survey of High School Juniors (Baseline Survey)

1. Eligibility

   Students eligible for the baseline survey were 11th graders (by credit) who were enrolled in at least one on-campus course, and who could understand simple written and spoken English. Foreign exchange students were excluded since they will not be in the country during their senior year.

2. Baseline Survey Parental Consent Procedures

   Parental consent procedures were identical to the successful Hutchinson Smoking Prevention Program passive consent procedure for informing parents of children in grades 5-12 who were eligible for a very similar survey activity. A passive consent procedure was especially appropriate for these older adolescents (16- to 17-year-olds) because no sensitive questions were included in the questionnaire. The procedure was as follows: Three weeks prior to the survey, using names and addresses provided to the study by the collaborating high school, we mailed a first class letter directly to the parent/guardian (here forward referred to as "parent") of each high school student eligible for the baseline survey. The letter was printed on school stationery and cosigned by the high school principal and the principal investigator. To make sure parents of all potential participants received a letter prior to the in-class survey, three calls were made to the mail handler at the school (2 weeks-, one week-, and 2-3 days-prior to the survey). During these calls, we determined if any parent letters were returned to the school by the postal service and whether or not parents had contacted the school to decline. If a correct address for parents whose letters were returned at the 2-

week-prior-to-survey call could be obtained right away, we mailed another parent mailing to the corrected address. The 16-17 year old children of parents whose information letters were returned undeliverable (and for whom we were not successful in contacting subsequently) were not included in the in-class survey.

The parent information letter included general information about the project and the district and high school's agreement to collaborate, and specific information about the in-class baseline survey, its questionnaire, the saliva sample procedure, the confidential treatment of the data collected, and the voluntary nature of participation. Importantly, also included were the project's toll-free phone number and an invitation to the parents to call with questions or to decline their student's participation in either the survey and/or the saliva sample collection.

For the few parents who had specifically asked their school not to release their names, addresses, and phone numbers, our procedure was to offer these families an opportunity to be included in the study by asking their school to mail their survey parent letters directly from the school, thereby not releasing the addresses to us. Also, for the few parents who didn't read English, we follow the school's procedures for informing them about the survey.

3. Baseline Survey Student Consent Procedures

Trained project data collectors presented a scripted explanation of procedures to students at the outset of each data collection class. The in-class explanation of procedures (script), similar in content to the parent letter, was designed to appeal to and be understood by high school students. The script assured students that we would keep their individual information confidential, that participation in the survey activities was voluntary, and that they may decline all or part of the survey if they wished. We also told students about the FHCRC, the rationale for the study, what we hoped to learn from the survey, and the content of the questionnaire. As part of the "pipeline" procedure, we told students that their saliva sample could be tested for cotinine, assured them that their saliva would NOT be tested for anything other than cotinine, explained that the test for cotinine was accurate and specific, and demonstrated how the saliva was to be collected.

4. Baseline Survey In-Class Data Collection Procedures

Procedures for the in-class baseline survey, conducted among high school juniors at the end of their junior year, were modeled after the successful Hutchinson Smoking Prevention Program in-class data collection procedures. The procedures were designed to meet the following goals: (1) Be sensitive to the interests and needs of students, parents, and schools (i.e., keep parents & students well-informed, keep responses confidential, make response voluntary, make the survey easy to read and complete, survey fits easily into one class period – even for slow readers, student questionnaire contains no questions about parents, questionnaire questions are not unnecessarily sensitive, data collection procedures don't unnecessarily infringe upon schools, classrooms, and teachers). (2) Obtain accurate data. (3) Obtain complete data. (4) Identify trial cohort members (smokers and non-smokers). (5) Develop rapport with participants, and motivate them to want to participate (which may translate into subsequent participation in the telephone counseling intervention and participation in the outcome data collection). (6) Use identical procedures and questionnaires in all participating schools. (7) Obtain data needed for the matched-pair randomization (e.g., prevalence of daily smoking and heavy smoking). (8) Obtain baseline levels of primary outcome measures (e.g., level/frequency of smoking, smokers' quitting history and readiness to quit). (9) Obtain baseline levels of secondary outcome measures (e.g., nicotine dependence, support for quit attempts of others, student perception of school policy and the school/community environment). (10) Obtain information on possible effect modifiers (e.g., dependence on nicotine, interest in quitting, intentions to quit, level of smoking in the student's social environment, demographic variables). (11) Obtain information on risk factors for not quitting (e.g., reasons for smoking, rebelliousness, history of smoking). (12) Obtain baseline levels of mediating variables (e.g., motivation for smoking/quitting, reasons and skills for quitting, beliefs, social norms, behavioral capacity/self efficacy). (13) Include survey items (relevant to the purpose of the survey) that participants will find interesting. (14) Obtain information on what smoking-related issues teens are interested. (15) Obtain each participant's future contact information.

To meet these goals, the in-class baseline survey was administered by trained project staff, an in-class "clean up" survey was conducted among those absent on the initial survey date, "pipeline" saliva samples were collected to encourage accurate self-reported smoking behavior, and, to achieve the required response rate, mailed and/or telephone surveys were conducted among students absent from both the initial and clean up in-class surveys.

Students absent from the initial and clean-up in-class surveys were asked to complete a telephone survey. If there had been no phone contact after several attempts, a survey was mailed to the student's home. The cover letter for the mailed survey and the script for the telephone survey were designed to mimic the in-class script.

An attempt was made to conduct a telephone survey with students who did not take part in the in-class survey because their parents did not receive a parent information letter. For these students, parental permission was obtained by reading them the parent information letter over the phone. If parental permission was obtained, the student telephone script was used to obtain student consent prior to conducting the telephone survey.

These procedures were successfully used in this study's Pilot (Spring 2001) and all 50 collaborating high schools. Participation among the 13,042 eligible juniors was 93.1%.

## C. Random Assignment of Schools to Experimental or Control Conditions

The HS Study trial used a two-arm, group-randomized design. The 50 participating high schools were randomly assigned either to the experimental condition (a Motivational Interviewing (MI) plus Cognitive Behavioral Skills Training (CBST) proactive telephone counseling intervention) or the control condition (no-intervention).

The randomization was performed via a computerized coin flip for each of the randomly ordered matched-pairs of high schools (formed from the baseline survey data by matching on prevalence of daily smoking and of heavy smoking). The randomization was witnessed, recorded and signed by two non-study FHCRC scientists. As soon as randomization was complete, the principal investigator telephoned the superintendents and high school principals to communicate the randomization results, and to reinforce the importance of the procedure and each district/high school's role to the integrity of the study.

These procedures were successfully used to randomly assign all 50 collaborating high schools to treatment arm.

## D. Trial Participant Selection

The cohort of trial participants consisted of 2,895 high school seniors selected from the students participating in the baseline survey. The cohort included both smokers and non-smokers. For this study, "smokers" were those students whose baseline smoking level was at least once a month. Non-smokers were a 15% sample of those students not smoking at baseline. (The latter sample was comprised of two types of non-smokers: (1) never smokers who were interested in helping smokers quit, and who had friends who smoke, and (2) ex-smokers who were interested in helping smokers quit, who had friends who smoke, and who had been abstinent from cigarettes for 3 months or more.)

## E. Summary of Telephone Counseling Intervention ("Matchbreaker")

1. Obtaining Consent for the Telephone Counseling Intervention

   Consent procedures for the intervention were developed to meet the following criteria: (1) Honor the rights of parents and the study cohort member. (2) Be sensitive to the needs and interest of parents, students, and schools. (3) Provide an accurate description of the intervention. (4) Use procedures designed to maximize

participation (a) by parents in the active consent process and (b) by study cohort members in the intervention. (5) Develop rapport with parents, motivating them to cooperate with the study, and in particular, to want their study cohort member to participate in the intervention. (6) In all consent communications (letters and telephone scripts), use simple, clear language that is easy to read and understand, and that is clean, pleasant looking, and simple to complete and send back. (7) Make the agreement to participation as easy as possible to understand, to fill out and to do.

a) Parental Consent Procedure

The first step of the Telephone Counseling Consent Procedure was to develop rapport with families of potential participants by mailing to the parent a rapport-building postcard in advance of the informed consent mailing. This card thanked the family for their role in the success of the Survey of High School Juniors, and reported the participation rate for the survey.

Active parental consent was required for those high school senior trial participants under 18 years of age. (However, if we learned from the schools that a trial participant < age 18 was an “emancipated minor”, or otherwise living on he/her own, we dropped the requirement for consent from his/her parents.)

Even though 18 year olds are considered adults in the state of WA, and therefore parental consent was not required, we informed the parents of 18 year old students about the survey by mailing them an informational letter (Parent Info Letter, or PIL) and a parent Frequently Asked Questions brochure (parent FAQ) one week prior to the mailing to their student. (Please refer to the Telephone Counseling Student Consent Procedure below.) Likewise, for the parents of participants who turned 18 after their parents had received the request for active parental consent (but who failed to reply after 30 days), we mailed a modified PIL.

We sought active parental consent via mail, with telephone follow-up as needed, a procedure that was used successfully with trial participants in all experimental schools. The mailing schedule was as follows: The initial packet (letter, parent FAQ, and Reply Card) went out on Day 0 (with a first class commemorative stamp on the outer envelope), followed by a reminder/thank you card on Day 5. Then, on Day 16 non-responder calls started. All correspondence with parents was designed to be informative, friendly and conversational.

Documentation of active parental consent among those parents who provided consent via the mail was the parent’s indication and signature on the Reply Card. Documentation of active parental consent among those parents contacted via a non-responder telephone call was a database record of the date and time of the consent, the name of the person providing the consent, and a summary of the consent conversation.

b) Student Consent Procedure

Soon after receiving parental consent (for students under age 18) or after mailing the parent information letter (students at least age 18), the study mailed an informational letter and a student Frequently Asked Questions brochure about the telephone counseling activity to students. The first counseling call was placed a week or more after the student informational letter mailing. In the first call, the counselor mentioned the letter, summarized the letter (as needed), reminded the student that the counseling conversations were confidential and that participation was voluntary and asked for the student’s consent to start. If any student wanted time to think about it, the counselor asked for a good time to call back.

2. Telephone Counseling Intervention Implementation

a) Overview

The intervention was implemented in experimental arm high schools during the participants' senior year. It consisted of individually tailored cessation counseling delivered via counselor-initiated telephone calls, supported by selected self-help materials and a cessation Web site. Intervention goals were to (1) obtain the participant's frank and personal insights about smoking and non-smoking, (2) motivate 12th grade smokers to want to quit smoking, to make a quit attempt, and to support them in their quit attempt, and (3) reinforce nonsmokers' choice not to smoke and provide them with the motivation and skills to help their smoking peers to quit.

Telephone counselors followed a structured protocol that (1) Delineated the goals and minimum content for each counseling session, set guidelines for responses to the teen's individual needs and statements, and outlined goals for each session and a rationale and a clinical approach (e.g., establish rapport, assess smoking status) for each goal. (2) Was sensitive to the teens' needs and individual circumstances while promoting teens' motivation to change and the belief that change is possible and desirable. (3) Helped teens develop the confidence and skills needed to successfully change their behavior. (4) Kept the telephone sessions brief and focused.

b) Counselor training and supervision

Counselors were trained in and used motivational interviewing (MI) techniques, which seek to help the client resolve ambivalence about behavior change and trigger a decision to change. Counselors followed a menu of broad MI strategies appropriate for exploring concerns about smoking and used specific interviewing skills (e.g., reflective listening, open-ended questions, summarizing) to guide teens through the counseling session. These techniques combined individually tailored feedback regarding the effects of tobacco use with an empathetic, non-confrontational therapeutic style.

Intervention Manager Kathleen Kealey hired, trained, and supervised a staff of part-time counselors with master's degrees in clinical psychology, educational psychology, or social work. Evette Ludman, Ph.D., a licensed Clinical Psychologist with extensive training and experience using motivational interviewing in clinical settings, assisted with training and met regularly with counselors to discuss clinical issues. Counselors underwent 150 hours of rigorous training in (1) brief motivational interviewing, (2) treating tobacco use and nicotine dependence,(3) role-playing with constructive feedback, (4) reflective listening, (5) emergency procedures, (6) computer-assisted counseling and associated software, and (7) a variety of topics relevant to teen smoking cessation, e.g., the role of self-image in smoking initiation and cessation, relapse prevention, skills-building, and characteristics of late adolescence. Ms. Kealey implemented a monitoring system for quality assessments of counselor calls, including monitoring counselor's sessions with clients, and conducted monthly in-service training for continuing education and constructive feedback. The counselors were also guided by the IRB-approved Telephone Counseling Intervention Protocol.

c) Intervention Implementation for Smokers

All smokers in the trial cohort from experimental arm high schools (with parental consent, as applicable) who agreed to participate received a prescribed series of counselor-initiated calls, together with selected supplemental mailings, to (1) encourage quitting behavior and (2) support quit efforts, as detailed below. Counselors paid special attention to development of rapport and encouragement of the teen's continued participation.

Smokers were scheduled to receive up to three counselor-initiated motivation-enhancement calls designed to induce stage progression and ultimately trigger a decision and commitment to quit. Counselors' goals for the initial call were: (1) Obtain student consent for participation; (2) Establish rapport; (3) Perform brief assessments of the teen's smoking status and progression towards quitting in terms of his/her readiness to quit stage, smoking frequency, and nicotine dependence; (4) Assess personal and environmental factors related to smoking; (5) Motivate serious consideration of quitting, tailoring the message to the teen's feedback and staging; and (6) Request permission to make subsequent

calls and set up a plan for continued contact. If the smoker declined future calls, he/she was sent self-help materials and referred to the Web site.

When a teen smoker was ready to quit and set a quit date, he/she began a relapse-sensitive schedule of telephone counseling aimed at supporting cessation and preventing relapse. Participants received up to six cessation support calls along with follow-up counselor mailings, which delivered cessation support and advice. These calls were scheduled to occur over 60 days, beginning on the quit date, scheduled for Days 0, 3, 7, 14, 30 and 60 (and adjusted, as needed, to the participant's schedule/needs). This relapse-sensitive schedule was designed to support the teen during the period in which his/her withdrawal symptoms and cravings were strongest, and the probability of relapse was greatest, thus optimizing the effect of counseling.

d) Intervention Implementation for Non-smokers

Non-smokers in the trial cohort of experimental high schools (with parental consent, if needed) who agreed to participate received one or more telephone calls from a counselor who briefly reinforced the nonsmoker's abstinence, reviewed tips for helping friends quit smoking, and motivated and coached the nonsmoker in providing feedback, support, and advice to smoking friends. The counselor helped the teen practice such strategies and sent relevant self-help print materials.

3. Intervention Supporting Materials and Activities

a) Self-help Materials

To further reinforce the telephone counseling calls, support decisions to quit, and help smokers through the quitting process, counselors followed up each phone contact with a mailing to the teen (with the teen's approval and to an address of his/her choice). Content of each mailing was tailored to the needs, interests, and staging of the teens. Also, for nonsmokers, self-help materials (e.g., "Tips for Helping Friends to Quit") provided important background information (e.g., what smoking friends may be dealing with while trying to quit) and multiple helping strategies, similar to those communicated by the counselor.

b) Supportive Web Site

The intervention's cessation Web site provided material designed to reinforce counselors' messages to teens who participate in the telephone counseling. Features included assessment of risk factors that predispose teens to begin smoking or become dependent upon tobacco; succinctly presented information about the effects of smoking (e.g., graphic illustration and animation may be used); brief assessment (use of short forms when possible) and feedback about smoking level and motivation to quit; smoking cessation support and advice (i.e., considerations about motivation, and how to prepare for a quit attempt, identify difficult situations, build support, develop coping strategies, and utilize relapse prevention skills); and links to other teen-oriented smoking-reduction web sites. As a way of encouraging their use of the site, teens had opportunities to play games, view smoking related cartoons and explore topics of interest. There was no registration requirement for study participants who visited the study's Web site.

c) School-based Intervention Activities

The intervention included a school-based component, designed to use the school environment to create support for youth smoking cessation. This component included meetings with key stake-holders (i.e., faculty and student leaders) to inform them of the opportunities seniors have for help with quitting smoking, and mobilizing student leaders to hang posters in the school halls, pass out brochures ("Friends helping friends"), etc.

The telephone counseling intervention protocol was implemented successfully among 150 Pilot trial participants and 1422 experimental trial participants.

## F. Trial Participant Tracking and the Plus-1 (Age-19) Outcome Survey

### 1. Overview

Tracking and outcome survey procedures for the HS Study were modeled after the Hutchinson Smoking Prevention Program's "Plus 2" data collection, which successfully located and surveyed 94% of that study's 8,388 member young adult trial cohort at around age 20.

Outcome data on this study's young adult trial cohort was collected after high school graduation, approximately 6 months after the intervention period, when most trial participants were age 19. In accordance with principles of good trial design, all trial participants, including those who moved, dropped out, or otherwise left the high school after the baseline survey, were followed for the Plus-1 Outcome Survey. Prior to the survey, we used basic tracking information (names, addresses, and phone numbers) provided by the high school and by students who responded to those questions on the baseline questionnaire, to contact parents/guardians to request their (at least) 18-year-old study participants' current name, address, and telephone number. This preliminary information request was called the Primary Contact Address Request (PCAR) data collection.

The Plus-1 Outcome Survey procedures were designed to meet the following goals: (1) Be sensitive to the interests and needs of trial participants (TPs), parents and schools. For example: keep parents & participants well-informed, keep responses confidential, make response voluntary, make the survey easy to read and complete and as short as possible, the survey design allows respondents to skip many questions that are not applicable to them, the survey contains no questions about parents [this meant no questions about what parents do, think, or feel], the survey questions are not unnecessarily sensitive, data collection procedures don't unnecessarily infringe upon anyone, including schools, the survey includes a few open-ended items to allow self-expression by TPs. (2) Obtain accurate data by motivating participants via the survey and associated letters/scripts (a) to feel comfortable providing honest and accurate responses, (b) to want to provide honest and accurate responses, and (c) to be able to recall/report accurately. (3) Obtain complete data, both high participation rates and low item-non-response, by using procedures proven successful in previous studies, and by including innovative survey design features. (4) If at all possible while meeting goals #1 & #3, validate self reported smoking abstinence among TP Smokers. [This was accomplished via redundancies within the survey and additional survey questions.] (5) Continue the process of developing rapport with trial participants and their families. (6) Use identical procedures and surveys for trial participants in experimental and control schools.

The Plus-1 Outcome Survey questionnaire (the "Wrap-Up Survey") was designed to obtain the following data: (1) Outcome levels of primary outcome measures: quit History in last 12 months, length of current abstinence (7-day, 1-month, 3-month, and 6-month abstinence), frequency/level of current smoking, readiness to quit smoking cigarettes/stage of change. (2) Outcome levels of secondary outcome measures: nicotine dependence, reasons for not smoking, reasons for quitting (past or future), reasons for smoking, support for quit attempts of others in the last 12 months, lifetime cigarettes, current frequency of use of tobacco products other than cigarettes, last use of any tobacco products other than cigarettes, intentions to smoke cigarettes in the future, perception that stopping smoking is easy, level of smoking in the social environment. (3) Outcome levels of mediating variables: whether thought about stopping in last 12 months, importance of quitting in last 12 months, confidence for quitting in last 12 months, details about attempts to stop smoking completely in last 12 months, importance of stopping, confidence for stopping, commitment to stopping, consideration of quitting, skills/self-efficacy for stopping, social support for stopping, ambivalence about smoking, importance of being a non-smoker for rest of life, level of commitment to being a non-smoker for rest of life, confidence for being a non-smoker for rest of life, skills/self-efficacy for being a non-smoker for rest of life, willingness to help others to quit smoking, Importance of helping someone quit, confidence for helping someone quit, stage of change for helping others quit, Skills for helping others quit,

beliefs about smoking/quitting, norms, social support, stress, self-efficacy for life. (4) Demographic information needed to describe the study population at outcome, such as highest education level attained to date. (5) Items that will increase the importance and salience of the survey.

2. Procedures for Trial Participant Tracking and Location

   a) Directory Information Updates via the National Change of Address Service

      In order to maximize the likelihood that the mailed Primary Contact Address Requests and Wrap-Up Surveys reached their intended addressee, and that any follow-up telephone contacts were made to the correct number, we conducted a Directory Information Update activity one month prior to the start of the Tracking and Plus-1 Wrap-Up Survey. To accomplish this, the names, addresses, and phone numbers of trial participants and their primary contacts (typically parents), provided to us by collaborating high schools at the outset of the study (many of which have since been updated by either the United States Postal Service (USPS), the trial participants, and/or their primary contacts) were submitted to Lorton Data's National Change of Address service and Telephone Append and Verify service. This input file submission and receipt of output was via secure ftp. Lorton Data certified that data provided to them was treated as confidential.

   b) Primary Contact Address Request (PCAR)

      i. Mailed Day 0 packet to parent/guardian of each trial participant (first class commemorative stamp): Day 0 letter, Address Update card, Reply envelope and a $2 cash incentive/thank you.
      ii. Mailed Day 5 post card mailing to parent/guardian of each trial participant: Day 5 Thank you/Reminder Post Card
      iii. Mailed Day 16 non-responder #1 packet to parents/guardians who have not yet replied (first class commemorative stamp): Day 16 non-responder letter, Address Update card, Reply envelope
      iv. Mailed Day 30 non-responder #2 packet to parents/guardians who have not yet replied (first class commemorative stamp): Day 30 non-responder letter, Address Update card, Reply envelope
      v. Day 47+: Made Non-responder phone calls to parents/guardians who had not yet replied

   c) "Lost" Parents/Guardians

      If a PCAR packet was returned by the post office without a forwarding address, the study attempted telephone contact using all possible phone numbers and contacts provided by the school, parent, other contact or student. If that failed, an attempt was made to obtain a current phone number for the parent/guardian or TP by using various directory assistance services, or by contacting the collaborating school. Once reached by phone, study staff used the PCAR Non-responder phone script to request that the parent/guardian provide the TPs current locator information. If the parent/guardian requested it, a PCAR packet was mailed.

      If the study contacted the TP in the process of tracking a lost parent/guardian, and if the TP was at least 18 years old, the study used the TP Non-responder phone script to request that the TP complete the Plus-1 Outcome Survey over the phone. If the TP requested it, a survey packet was mailed. If the TP was less than 18 years old, the study staff continued to attempt to contact the parent/guardian.

3. Procedures for the Plus-1 Outcome Survey ("Wrap-Up Survey")

   a) Mailed Day 0 survey packet to Trial Participants (TPs): Day 0 Letter, Wrap Up Survey, FHCRC Pencil, $10 cash, Reply Envelope
   b) Mailed Day 5 post card to TPs included in Day 0 mailing: Thank you/Reminder Post Card
   c) Mailed Day 16 non-responder packet #1 to TPs who had not yet responded: Day 16 Non-responder letter, Wrap-Up Survey, Pencil, Reply Envelope

d) Mailed Day 30 non-responder packet #2 to TPs who had not yet responded: Day 30 Non-responder letter (promising $20 upon receipt of completed survey), Wrap-Up Survey, Pencil, Reply Envelope
e) Using a colorful envelope, mailed Day 47 non-responder packet #3 to TPs who had not yet responded: Day 47 Non-responder letter (promising $20 upon receipt of completed survey), Wrap-Up Survey, Pencil, Reply Envelope
f) Day 60+ Made Non-responder phone calls to TPs who had not yet replied
g) Upon receipt of surveys completed after the Day 30 mailing, or completed over the telephone during tracking, mailed $20 cash to the TP along with a thank you note.

[Note: Importantly, the PCAR and the Wrap-Up Survey procedures included monetary incentives in a multiple-contact survey plan in which a $1 cash prepaid incentive was included in the initial PCAR mailing, and a $10 cash prepaid incentive was included in the initial survey packet. A promise of $20 cash upon receipt of a completed survey was included in all communication among non-responders after 30 days. The purpose of incentives for follow-up surveys was to obtain the needed response rate (over 90%). Research has shown that (1) a modest prepaid monetary incentive (e.g., $10 for a survey, or $1 for the minimal PCAR request) works well for encouraging response from many survey participants, yielding nearly double the initial response over a survey conducted without a prepaid incentive, and (2) a promised incentive of $20 can often double the response to a second survey mailing to initial non-responders. This method was used in our previous longitudinal study (the Hutchinson Smoking Prevention Project). That study obtained a 94% survey participation rate, thus achieving the very high scientific rigor required for the study. The procedures worked well, and we received no complaints from the study's 8,388 young adult participants. In the unlikely event that we received a call from an early-responding participant requesting additional payment, our plan was to send him/her $20 (after explaining the purpose of the incentives and the two-tier system).]

These procedures were used successfully during the Plus-1 Outcome Survey. We achieved a survey location and participation rate of 89.1% among the 2,863 eligible trial participants.

## G. School Environment Survey (Survey of High School Principals)

Data on school structure, policies, resources and communications were collected from high school principals at collaborating high schools once at the outset of each school's collaboration and again at the end of the intervention period. The survey was conducted as a sequenced mailed survey with telephone follow-up of non-responders, the same as the Plus-1 Outcome Survey procedure but without the monetary incentives. The cover letter for the initial survey packet explained the purpose of the survey, assured potential participants that the data collected would be kept confidential, and told them that participation was voluntary.

The baseline Survey of High School Principals was successfully conducted with the principals of all collaborating high schools, with 100% participation. The outcome survey was successfully administered to all collaborating high school principals.

## H. Trial Participant Tracking and the Plus-7 (Age-25) Outcome Survey

### 1. Overview

The Plus-7 follow-up and survey targeted the 2,151 members of the HS Study's original *smoker* cohort when most were age 25. Excluded were 46 original participants who were either deceased or declined future participation prior to the follow-up, leaving 2,105 original smokers eligible for the Plus-7 follow-up.

Procedures for locating and re-surveying the HS smoker cohort were similar to those used successfully for the previous Plus-1 Outcome Survey (as outlined in section F above).

2. Procedures for Trial Participant Tracking and Location

   a) Submission of names, addresses and telephone numbers in the HS database, collected at baseline for the purpose of future follow-up, to the National Change of Address Service (NCOA) via Lorton Data, who certifies that data provided to them will be kept confidential.

   b) A “soft tracking” mailing to participants and/or their primary contacts (typically a parent) in order to utilize the United States Postal Service’s (USPS) Address Service Request.

   c) Use of Lexus Nexus’ Accurint person search service, with IRB-approved procedures, for obtaining contact information for lost study participants.

   d) A mailing to the participant’s primary contact, requesting updated contact information for the participant, with mail and telephone follow-up of non-responders. The mail/phone follow-up sequence for the Primary Contact Address Request (PCAR) was as follows.

      i. Day 0 Initial mailing to all eligible participants
      ii. Day 6 Thank you/Reminder postcard to all eligible participants
      iii. Day 16 Non-responder mailing #1
      iv. Day 30 Non-responder mailing #2
      v. Day 35 Begin non-responder telephone calls
      vi. Day 77 End non-responder telephone calls

3. Procedures for the First Plus-7 Outcome Survey (“TOPS”)

   a) Mailing the Plus-7 TOPS Survey to participants, with mail, telephone and online follow-up of non-responders. The sequence of activities for TOPS was as follows.

      i. Day -7 Advance letter to all eligible participants, signed by the Principal Investigator
      ii. Day 0 Initial mailing with survey to all eligible participants
      iii. Day 6 Thank you/Reminder postcard to all eligible participants
      iv. Day 16 Non-responder mailing #1
      v. Day 30 Non-responder mailing #2, including an online survey option for TOPS
      vi. Day 48 Non-responder mailing #3, including an online survey option for TOPS
      vii. Day 62 Begin non-responder telephone calls (there is no fixed end date for these calls)

   b) Use of prepaid and promised monetary incentives to increase participation: Specifically, a $1 cash prepaid incentive was included in the initial PCAR mailing, a $10 cash prepaid incentive was included in the initial TOPS Survey packet, and a promise of $20 cash upon receipt of a completed TOPS Survey was included in all communications with non-responding participants after 30 days. This incentive method had already been used with good results for the HS Primary Contact Address Request, the HS Plus-1 Outcome Survey and both the Hutchinson Smoking Prevention Program age 20 and age 28 surveys.

4. Rationale and Procedures for the Second Plus-7 Outcome Survey (“SNAP”)

   Early in 2013, after monitoring TOPS survey response rates through part of our final wave of participants, we concluded that we were falling well short of our minimum goal of 80% response over the whole study cohort. We re-evaluated our instrument and procedures and, with IRB approval, designed a final data collection effort that targeted all nonresponders to the TOPS survey. The special survey for TOPS nonresponders was named the Smoking and Nonsmoking Attitudes and Practices Survey (SNAP). The SNAP survey effort employed a number of modifications to TOPS procedures, with the aim of reaching and surveying nonresponders. These included:

a) Prefacing the survey effort with intensive tracking of previously unreached study participants.

b) Modifying the survey instrument to (1) reduce survey length and complexity, while retaining the items that support our most important research aims; (2) improve survey saliency; and (3) improve survey appearance with glossy front and back covers with appealing photographic images.

c) Using a modified version of the standard mail/phone/online follow-up procedures. The sequence of activities for SNAP was as follows.

   i. Day 0 Initial survey mailing to all eligible participants
   ii. Day 6 Thank you/Reminder postcard to all eligible participants
   iii. Day 16 Non-responder survey mailing #1, including an online survey option for SNAP
   iv. Day 30 Non-responder survey mailing #2, including an online survey option for SNAP
   v. Day 48 Non-responder survey mailing #3, including an online survey option for SNAP
   vi. Day 62 Non-responder telephone calls begin
   vii. Day 100 Final survey mailing to all remaining non-responders, including an online survey option

d) Using enhanced, inflation-adjusted incentives of $20 prepaid, in the initial mailed survey packet, and, starting with the second nonresponder mailing, a promise of $40 for a returned survey.

The SNAP survey achieved its participation goal, giving us a final Plus-7 Outcome Survey data collection response rate of 81.5%

Data from the Plus-7 Outcome Survey will be used along with the Baseline and Plus-1 Outcome Survey data, and intervention implementation data, to address the research objectives outlined in the study's grant proposal.
