# Supplementary material for: Does Effectiveness of Adolescent Smoking-Cessation Intervention Endure Into Young Adulthood? 7-Year Follow-Up Results from a Group-Randomized Trial
Source: PLoS One. 2016 Feb 1;11(2):e0146459. doi: 10.1371/journal.pone.0146459 (PMC4734743; doi:10.1371/journal.pone.0146459)
Supplement: S3 File — (PDF) [file pone.0146459.s003.pdf]

FRED HUTCHINSON CANCER RESEARCH CENTER

SURVEY OF HIGH SCHOOL JUNIORS

Place label like this

Form #BQ04-0303

INSTRUCTIONS

- **Please read and follow these directions.**
- **Please use the pencil we gave you.**
- **Answer each question as best you can. Carefully fill in the circle of the response that fits you. (Example: ●)**
- **If you need to erase, please erase completely.**
- **Raise your hand if you have any questions.**
- **All of the questions about smoking are about cigarettes.**

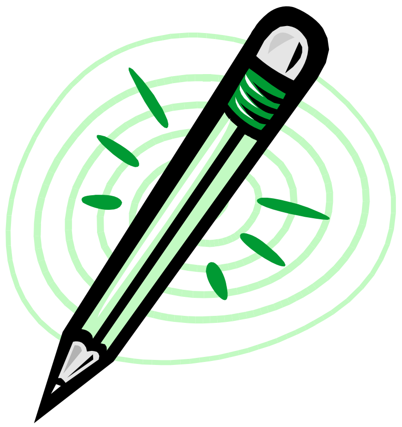

All of your answers on this survey will be kept confidential.

START HERE

First a few questions about you.

1. Are you male or female?

○ Female
○ Male

2. What is your birth date?

_______ / ______ /________
Month Day Year

3. What is your race/ethnicity?

(Please mark all that apply.)

○ American Indian or Alaska Native
○ Asian
○ Black or African American
○ Hispanic
○ Native Hawaiian or Other Pacific Islander
○ White
○ Other: ______________________________

____

These questions will help us understand the opinions of high school juniors.

4. What is your overall feeling about cigarette smoking?

○ Very pro-smoking
○ Somewhat pro-smoking
○ No strong feeling one way or the other
○ Somewhat anti-smoking
○ Very anti-smoking

5. Does cigarette smoke bother you?

○ No, not at all
○ Not much
○ Yes, a little
○ Yes, a lot

6. Do you think the health hazards of smoking are overstated?

○ No
○ Yes
○ No opinion/don't know

7. Do you think there are too many restrictions about where and when people may smoke?

○ No
○ Yes
○ No opinion/don't know

Next are some questions about cigarette smoking and non-smoking.

8. Have you ever smoked a cigarette?

- ○ No, never; not even one puff
- ○ Yes, but just one puff or a few puffs
- ○ Yes, but only one cigarette
- ○ Yes, more than one cigarette

9. Have you ever smoked regularly?

- ○ No, I've never smoked at all.
- ○ No, I've never smoked regularly.
- ○ Yes, I smoke regularly now.
- ○ Yes, I used to smoke regularly, but I quit completely. *Please answer the following question:*

↓

How many years, months, or days have you gone without a cigarette? *(Please write in a number.)*

________ **or** ________ **or** ________
Years Months Days

10. How many cigarettes have you smoked in your lifetime?

- ○ None, not even a puff
- ○ One cigarette or less
- ○ 2 – 20 cigarettes (up to one pack)
- ○ 21 – 100 cigarettes (2 – 5 packs)
- ○ 101 – 400 cigarettes (6 – 20 packs)
- ○ Over 400 cigarettes (more than 20 packs)

11. What was the last time you smoked, or even tried, a cigarette?

- ○ I have never smoked, or even tried, a cigarette.
- ○ Earlier today
- ○ 1 – 7 days ago
- ○ 8 – 30 days ago
- ○ Between 1 month and 3 months ago
- ○ Between 3 months and 6 months ago
- ○ Over 6 months ago

12. How often do you currently smoke cigarettes?

- ○ Not at all
- ○ Less than once a month
- ○ Once a month or more, but less than once a week
- ○ Once a week or more, but not daily

} GO TO Page 3.

- ○ At least daily – *Please answer questions (a) through (d) too.*

↓

a) How many cigarettes per day do you smoke?

- ○ 1 cigarette per day
- ○ 2 – 5 cigarettes per day
- ○ 6 – 10 cigarettes per day
- ○ 11 – 20 cigarettes per day
- ○ More than 20

b) How soon after waking up do you have the urge for a cigarette?

- ○ As soon as I wake up
- ○ Within about ½ hour of waking
- ○ An hour or two after I wake up
- ○ Over 2 hours after waking
- ○ Don't really have an urge to smoke

c) How soon after waking up do you smoke your first cigarette of the day?

- ○ As soon as I wake up
- ○ Within about ½ hour of waking
- ○ An hour or two after I wake up
- ○ Over 2 hours after waking

d) How soon after leaving home in the morning do you smoke your first cigarette?

- ○ I smoke my first cigarette before leaving home.
- ○ As soon as I leave home
- ○ Within about ½ hour of leaving home
- ○ An hour or two after leaving home
- ○ Over 2 hours after leaving home

13. Have you smoked one or more cigarettes in the last 30 days?

- ○ Yes → GO TO Page 5.
- ○ No

Continue with #14, below. below

14. What are your reasons for not smoking cigarettes? For each reason (a) through (s) listed below, please fill in the circle that describes how important the reason for not smoking is for you.

I don't smoke cigarettes . . .	Not at all important	Fairly important	Very important
a) because none of my friends smoke.	○	○	○
b) to avoid getting a serious illness or disease.	○	○	○
c) to prove to myself that I can keep from smoking.	○	○	○
d) because it smells bad.	○	○	○
e) because family or friends would hassle me about it.	○	○	○
f) to stay healthy for physical activities, such as sports or dance.	○	○	○
g) to stay healthy for musical activities, such as choir or band.	○	○	○
h) because I feel better about myself as a non-smoker.	○	○	○
i) because it's expensive.	○	○	○
j) because someone told me I absolutely could not smoke.	○	○	○
k) to feel in control of my life.	○	○	○
l) because I will receive a special gift or reward if I don't smoke.	○	○	○
m) because I have relatives or friends who died from smoking-related diseases.	○	○	○
n) because not smoking will prove I can accomplish things that are important to me.	○	○	○
o) because people I am close to will be upset with me if I smoke.	○	○	○
p) because I am concerned that smoking may cause me to die early.	○	○	○
q) to avoid addiction to cigarettes.	○	○	○
r) because I refuse to support the tobacco industry.	○	○	○
s) because I never even thought about smoking.	○	○	○

→

Questions for students who smoked no cigarettes in the last 30 days . . .

15. Do you feel comfortable not smoking even when you're with others who smoke?

- ○ Yes
- ○ No
- ○ Don't know

16. Do you know how to get out of situations where others might want you to smoke?

- ○ Yes, for sure
- ○ Yes, I think so
- ○ No
- ○ Don't know

17. How important is it to you to remain a non-smoker for the rest of your life?

- ○ Not at all important
- ○ A little important
- ○ Fairly important
- ○ Very important

18. How confident are you that you will remain a non-smoker for the rest of your life?

- ○ Not at all confident
- ○ A little confident
- ○ Fairly confident
- ○ Very confident

19. Is there anything else you'd like to say about your decision not to smoke?

- ○ No
- ○ Yes: ______________________________

____

____

____

____

20. Would you like to know more about the best ways to help friends or family members quit smoking?

- ○ Yes
- ○ No
- ○ Don't know

21. Would you ever be willing to help a friend or another student to quit smoking?

- ○ No, definitely not
- ○ No, probably not
- ○ Yes, probably
- ○ Yes, definitely
- ○ Don't know

22. How important is it to you to help your friends who want to quit smoking?

- ○ Not at all important
- ○ A little important
- ○ Fairly important
- ○ Very important

23. How confident are you that you would be able to help a friend to quit smoking?

- ○ Not at all confident
- ○ A little confident
- ○ Fairly confident
- ○ Very confident

24. In the last 12 months, have you encouraged any of your friends to quit smoking?

- ○ None of my friends smoke.
- ○ No
- ○ Yes →

How many?

- ○ One
- ○ Two
- ○ Three
- ○ Four
- ○ Five or more

GO TO Page 8.

Questions for students who smoked one or more cigarettes in the last 30 days . . .

25. On how many days in the last 30 days have you smoked at least one cigarette?

- Every day
- 20 – 29 days
- 10 – 19 days
- 5 – 9 days
- 2 – 4 days
- 1 day

26. What are your most important reasons for smoking cigarettes, even if you smoke only once a month?

(Please mark all that apply.)

- I smoke to change the way I feel; for example, to relax or reduce stress.
- I smoke for pleasure.
- I smoke because my friends or other people I'm around smoke.
- I smoke because I'm addicted or have the smoking habit.
- I smoke because I like the way it looks.
- I smoke because it tastes good.
- I smoke to keep my weight down.
- I smoke to keep from being bored.
- I smoke just because I want to.
- I smoke because it's my choice/right to smoke if I want.
- I don't know why I smoke.
- Other reasons: ____________________

____

____

27. How old were you when you . . .

a) smoked your very first cigarette?

- Age 8 or before
- 9 – 10
- 11 – 12
- 13 – 14
- 15 – 16
- Over age 16

b) first smoked at least once a week?

- Age 8 or before
- 9 – 10
- 11 – 12
- 13 – 14
- 15 – 16
- Over age 16
- Never smoked once a week

c) first smoked every day?

- Age 8 or before
- 9 – 10
- 11 – 12
- 13 – 14
- 15 – 16
- Over age 16
- Never smoked every day

28. Think back over the last 12 months. Did you try to stop smoking completely anytime in the last 12 months?

- No, I did not try to stop smoking completely anytime in the last 12 months.
- Yes, I did try to stop smoking completely in the last 12 months. *Please answer questions (a) and (b) too.*

↓

a) How many times did you try to stop smoking completely in the last 12 months?

________ Times

b) Of these times, how many months or days was the longest time you went without smoking?

__________ Months **or** __________ Days

29. How much have you thought about the pros and cons of cigarette smoking?

- Not at all
- Only a little
- Quite a bit
- A lot

30. Do you have mixed feelings about your smoking?

- Yes
- No
- Don't know

31. Do you like the idea of being a smoker?

- Yes
- No
- Don't know

32. Do you think your smoking could get in the way of important future goals?

- Yes
- No
- Don't know

→

Questions for students who smoked one or more cigarettes in the last 30 days . . .

Now we'd like to know why some students want to stop smoking completely and others don't.

33. People who smoke can be at different levels of thinking about stopping. Fill in one circle that indicates where you are now.

- ○ I am not thinking of stopping.
- ○ I think I need to consider stopping someday.
- ○ I think I should stop, but I'm not quite ready.
- ○ I'm starting to think about how to change my smoking patterns.
- ○ I'm taking action now to stop smoking (for example, cutting down, starting a program).

34. Would you cut down or stop smoking entirely if you knew an easy way to do so?

- ○ No, I wouldn't cut down or stop, even if I knew an easy way.
- ○ Yes, I'd cut down or stop if I knew an easy way.
- ○ Don't know
- ○ Other: ______________________________

35. Do you plan to stop smoking entirely someday?

- ○ No
- ○ Yes, in the next 30 days
- ○ Yes, between 1 and 6 months from now
- ○ Yes, sometime in the future, but not in the next 6 months
- ○ Don't know

36. Do you think that if you put your mind to it, you could stop smoking?

- ○ Yes
- ○ No
- ○ Don't know

37. How <u>important</u> is it for you to stop smoking completely?

- ○ Not at all important
- ○ A little important
- ○ Fairly important
- ○ Very important

38. If you were to try to stop smoking, how <u>confident</u> are you that you could keep from smoking for at least 6 months?

- ○ Not at all confident
- ○ A little confident
- ○ Fairly confident
- ○ Very confident

39. How <u>committed</u> are you to becoming a non-smoker someday?

- ○ Not at all committed
- ○ A little committed
- ○ Fairly committed
- ○ Very committed

40. How do you think most of your close friends would feel about it if you were to stop smoking?

- ○ They would definitely want me to stop.
- ○ They might want me to stop.
- ○ They wouldn't care whether or not I stop.
- ○ They might <u>not</u> want me to stop.
- ○ They would definitely <u>not</u> want me to stop.
- ○ I don't know what they'd think.

41. Do your friends ever bring up the idea that you should stop smoking?

- ○ No, never
- ○ Once in a while
- ○ All the time

42. Would you like to know more about the pros and cons of smoking and quitting?

- ○ No
- ○ Yes
- ○ Don't know

43. Would you like to know more about how you could cut down or stop smoking?

- ○ No
- ○ Yes
- ○ Don't know

44. Would you like to have someone help you stop smoking?

- ○ No
- ○ Yes
- ○ Don't know

Questions for students who smoked one or more cigarettes in the last 30 days . . .

45. For each reason (a) through (s) listed below, please fill in the circle that describes how important the reason for stopping smoking *might* be for you.

I *might* want to stop smoking cigarettes (now or someday) . . .	Not at all important	Fairly important	Very important
a) because none of my friends smoke.	○	○	○
b) to avoid getting a serious illness or disease.	○	○	○
c) to prove to myself that I can stop smoking if I really want to.	○	○	○
d) so I'll smell better.	○	○	○
e) to stop friends or family from hassling me about it.	○	○	○
f) because I can already tell that smoking is hurting my health.	○	○	○
g) to do better at physical activities, such as sports or dance.	○	○	○
h) to do better at musical activities, such as choir or band.	○	○	○
i) because I would feel better about myself if I were a non-smoker.	○	○	○
j) because it's expensive.	○	○	○
k) because someone told me I absolutely must stop smoking.	○	○	○
l) to feel in control of my life.	○	○	○
m) because I will receive a special gift or reward if I stop smoking.	○	○	○
n) because I have relatives or friends who died from smoking-related diseases.	○	○	○
o) because stopping smoking will prove I can accomplish things that are important to me.	○	○	○
p) because people I am close to will be upset with me if I keep smoking.	○	○	○
q) because I am concerned that smoking may cause me to die early.	○	○	○
r) to prove to myself that I am not addicted to cigarettes.	○	○	○
s) to stop supporting the tobacco industry.	○	○	○

46. Is there anything else you'd like to say about your reasons for smoking or for wanting to stop smoking?

○ No
○ Yes: ____

____

The rest of the questions are for EVERYONE.

47. How often do you currently smoke or use . . .	Not at all	Less than once a month	Once a month or more, but not once a week	Once a week or more, but not daily	At least daily
a) chewing tobacco or snuff?	○	○	○	○	○
b) cigars?	○	○	○	○	○
c) bidis or clove cigarettes?	○	○	○	○	○
d) pipe tobacco?	○	○	○	○	○

48. When was the last time you used any tobacco product other than cigarettes (e.g., chewing tobacco, snuff, cigars, bidis, clove cigarettes, or pipe tobacco)?

- ○ I have never used, or even tried, other tobacco products.
- ○ Earlier today
- ○ 1 – 7 days ago
- ○ 8 – 30 days ago
- ○ Between 1 month and 3 months ago
- ○ Between 3 months and 6 months ago
- ○ Over 6 months ago

What do you think about these things that have been said about cigarette smoking?

49. Do you believe . . .	Yes	No	Don't know
a) that people have to smoke a long time before it will hurt their health?	○	○	○
b) that smoking can help people when they are bored?	○	○	○
c) that there is any harm in having an occasional cigarette?	○	○	○
d) that smoking can help people relax?	○	○	○
e) that smoking can help people keep their weight down?	○	○	○
f) that almost all doctors think their patients should not smoke?	○	○	○

Your friends . . .

50. How many of your five closest friends . . .	None	One	Two	Three	Four	Five
a) smoke cigarettes?	○	○	○	○	○	○
b) use chewing tobacco or snuff?	○	○	○	○	○	○

51. **<u>In the last 12 months</u>, have you seen posters at school, either about stopping smoking or about a Web site that can help students stop smoking?**

- ○ No
- ○ Yes
- ○ Don't know

52. **What happens to students when they are caught smoking at school?**

(Please mark <u>all</u> that apply.)

- ○ Nothing
- ○ Sent to the office
- ○ Stay after school
- ○ Parents are notified
- ○ Required to go to a special class
- ○ Suspended from sports and activities
- ○ Suspended from school
- ○ Arrested
- ○ Expelled from school
- ○ There's no rule against smoking at my school.
- ○ Don't know
- ○ Other: ____________________________

____

53. **How often are you around <u>people over age 25</u> who smoke cigarettes?**

- ○ A lot
- ○ Sometimes
- ○ Hardly ever
- ○ Never

54. **How often are you around <u>high school students</u> who smoke cigarettes?**

- ○ A lot
- ○ Sometimes
- ○ Hardly ever
- ○ Never

55. **Do you think you will smoke cigarettes in the future?**

- ○ Yes, definitely
- ○ Yes, I think so
- ○ No, I don't think so
- ○ No, definitely not
- ○ Don't know

The next items ask how much you AGREE or DISAGREE with each statement.

56. **Most students at my high school smoke.**

- ○ Strongly agree
- ○ Agree
- ○ Disagree
- ○ Strongly disagree
- ○ Don't know

57. **Most students at my school think that smoking cigarettes is a bad idea.**

- ○ Strongly agree
- ○ Agree
- ○ Disagree
- ○ Strongly disagree
- ○ Don't know

58. **I think smoking cigarettes is a bad idea.**

- ○ Strongly agree
- ○ Agree
- ○ Disagree
- ○ Strongly disagree
- ○ Don't know

59. **Most high school students who smoke wish they could stop smoking.**

- ○ Strongly agree
- ○ Agree
- ○ Disagree
- ○ Strongly disagree
- ○ Don't know

60. **Smoking is addictive.**

- ○ Strongly agree
- ○ Agree
- ○ Disagree
- ○ Strongly disagree
- ○ Don't know

61. **Stopping smoking is often very hard to do.**

- ○ Strongly agree
- ○ Agree
- ○ Disagree
- ○ Strongly disagree
- ○ Don't know

62. Stopping smoking often leads to gaining weight.

- ○ Strongly agree
- ○ Agree
- ○ Disagree
- ○ Strongly disagree
- ○ Don't know

63. Smoking is considered cool by most people at my school.

- ○ Strongly agree
- ○ Agree
- ○ Disagree
- ○ Strongly disagree
- ○ Don't know

64. There is help at my school for students who want to stop smoking.

- ○ Strongly agree
- ○ Agree
- ○ Disagree
- ○ Strongly disagree
- ○ Don't know

65. Most adults smoke.

- ○ Strongly agree
- ○ Agree
- ○ Disagree
- ○ Strongly disagree
- ○ Don't know

66. Most adult smokers wish they could stop smoking.

- ○ Strongly agree
- ○ Agree
- ○ Disagree
- ○ Strongly disagree
- ○ Don't know

67. I have good grades in most of my classes this year.

- ○ Strongly agree
- ○ Agree
- ○ Disagree
- ○ Strongly disagree
- ○ Don't know

Please read each sentence, then choose the answer that best describes you.

68. I am basically satisfied with my life.

- ○ Just like me
- ○ Somewhat like me
- ○ Only a little like me
- ○ Not like me

69. During the past year, I've had a lot of stress in my life.

- ○ Just like me
- ○ Somewhat like me
- ○ Only a little like me
- ○ Not like me

70. When I need help with problems I'm having, my friends try to understand and give me the support I need.

- ○ Just like me
- ○ Somewhat like me
- ○ Only a little like me
- ○ Not like me

71. Most of the time, when my friends want me to do something, I go along with it.

- ○ Just like me
- ○ Somewhat like me
- ○ Only a little like me
- ○ Not like me

72. Doing the best I can in school is very important to me.

- ○ Just like me
- ○ Somewhat like me
- ○ Only a little like me
- ○ Not like me

73. I am willing to work hard to achieve success in life.

- ○ Just like me
- ○ Somewhat like me
- ○ Only a little like me
- ○ Not like me

74. I have family or friends to turn to when I need help.

- ○ Just like me
- ○ Somewhat like me
- ○ Only a little like me
- ○ Not like me

75. I can handle anything that comes my way.

- ○ Just like me
- ○ Somewhat like me
- ○ Only a little like me
- ○ Not like me

76. I can do anything I put my mind to.

- ○ Just like me
- ○ Somewhat like me
- ○ Only a little like me
- ○ Not like me

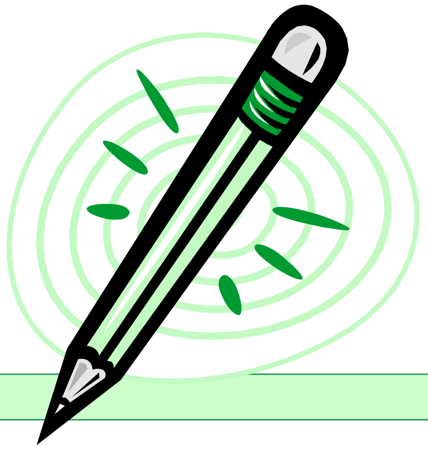

Now we'd like your feedback about the survey.

1. Did you know we were coming *today?*

- ○ Yes → When did you hear we were coming? ________________________________
- ○ No

2. Did you answer the survey questions as accurately as you could?

- ○ Yes, I answered all of the questions accurately.
- ○ Yes, I answered most of the questions accurately.
- ○ I only answered some of the questions accurately.
- ○ Other: ________________________________

Please write down anything else we can do to improve this survey and its questions:

____
____
____
____
____
____
____

→

Now, please complete Page 13.

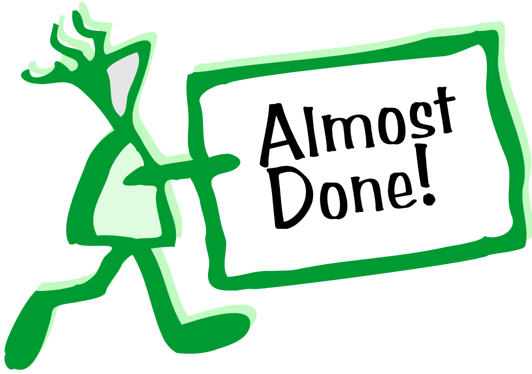

HELPFUL CONTACT INFORMATION

In the future, we may invite some of you to take part in another activity. We'll use the information below to contact you to see if you want to take part.

This information, like all of your survey answers, will be kept confidential.

1. **What might be some good ways to reach you next fall?**

By telephone:

Family home phone number: (__ __ __) ___ ___ ___ - ___ ___ ___ ___

Personal home phone: (__ __ __) ___ ___ ___ - ___ ___ ___ ___

Personal cell phone: (__ __ __) ___ ___ ___ - ___ ___ ___ ___

Other phone: (__ __ __) ___ ___ ___ - ___ ___ ___ ___

By e-mail:

Personal e-mail: ____

Family e-mail: ____

Other: ____

2. **After graduation, who might know how to reach you (other than your parents)?**

(Partial information is fine. Even just a name and city/state would help us.)

**First and Last Name** *(for example, your grandmother, aunt, uncle, friend)*		
**Address**		
**City**	**State**	**Zip**

Telephone: (__ __ __) ___ ___ ___ - ___ ___ ___ ___

Relationship to you: ____________________________________

Thank You for completing the Survey of High School Juniors.

Please raise your hand and we'll pick up your survey.
(The pencil is yours to keep.)
