# Supplementary material for: Does Effectiveness of Adolescent Smoking-Cessation Intervention Endure Into Young Adulthood? 7-Year Follow-Up Results from a Group-Randomized Trial
Source: PLoS One. 2016 Feb 1;11(2):e0146459. doi: 10.1371/journal.pone.0146459 (PMC4734743; doi:10.1371/journal.pone.0146459)
Supplement: S4 File — (PDF) [file pone.0146459.s004.pdf]

Hutchinson Study of High School Smoking (HS Study)

Matchbreaker

Telephone Intervention Protocol

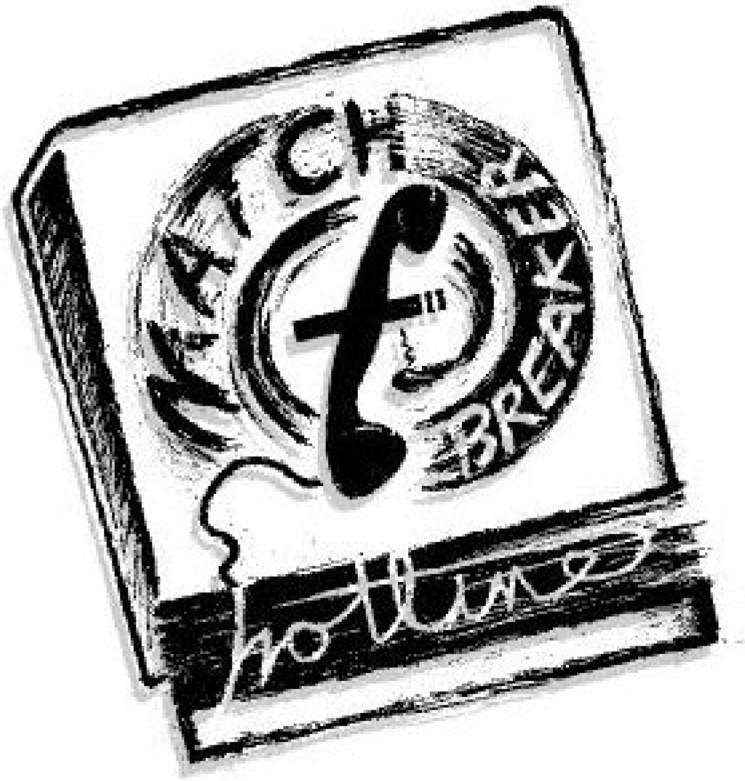

August 28, 2001

Revised June 25, 2002, September 5, 2002, July 23, 2003

Adapted for dissemination June 6, 2011

© Fred Hutchinson Cancer Research Center, 2001

MATCHBREAKER Telephone Intervention Protocol

TABLE OF CONTENTS

1. BACKGROUND ... 1

1.1 Overview: The Hutchinson Study of High School Smoking (HS Study) ... 1
1.2 Intervention Premises ... 2
1.3 Intervention Goals ... 3
1.4 Challenges ... 3
1.4.1 Reaching/making (initial and follow-up) contact with older teens ... 3
1.4.2 Making the goals of our trial salient to trial participants/Obtaining consent ... 3
1.4.3 Tailoring the intervention to each individual within our target population ... 4
1.4.4 Scheduling "Quit Preparation" with participants ... 4
1.4.5 Reducing relapse ... 4
1.4.6 Counseling participants who use other forms of tobacco (e.g., snuff, chew, cigars) ... 4
1.4.7 Counseling participants who do not smoke ... 4
1.5 Intervention Strategies ... 4
1.5.1 Tailor the intervention to the individual smoker ... 4
1.5.2 Use the telephone to reach smokers ... 5
1.5.3 Use proven intervention strategies ... 5
1.5.4 Provide useful self-help materials ... 6
1.5.5 Provide access to cessation materials and encouragement via a Web site ... 6
1.5.6 Be (pleasantly) persistent in contacting new and continuing participants ... 6
1.6 Approaches and Models Used in the Intervention ... 6
1.6.1 Motivational Interviewing ... 7
1.6.2 Cognitive-Behavioral Therapy ... 7
1.6.3 Integrating MI and CBT ... 8
1.6.4 Transtheoretical Model of Behavior Change ... 8
1.6.5 Matching the Intervention to the Stage of Change ... 11

2. THE PROACTIVE TELEPHONE COUNSELING INTERVENTION ... 15

2.1 Smoking Acquisition Among High School Students ... 15
2.2 Overview of the Proactive Telephone Counseling Intervention ... 16

3. SUCCESSFULLY CONTACTING TRIAL PARTICIPANTS ... 19

3.1 Challenges ... 19
3.2 Contact Goals ... 19
3.2.1 Trial goals for parent and student agreement to participate ... 19
3.2.2 Goals for contacting trial participants ... 19
3.3 Contact Strategies for Initial Calls ... 19
3.4 Contact Strategies for Follow-up Calls ... 20
3.4.1 TP misses call appointment ... 21
3.4.2 Leaving voicemail messages ... 21
3.4.3 Trying different phone numbers ... 22
3.5 Call Timing for Motivation Enhancement Calls ... 22

3.5.1 Target windows for motivation enhancement calls 22
3.6 Call Timing for Cessation Support Calls 23
3.6.1 Timing for participants who stopped since the last call 24
3.7 Tracking Counselor's Calls 24
3.8 Flexible Counselor Work Schedule for Calling 25
3.9 General Tips for Telephone Contacts 25
3.10 Participants Who Are Drunk or High 26
3.11 Calling a participant whose name is difficult to pronounce 26

4. COUNSELOR MAILINGS TO PARTICIPANTS 27

4.1 Strategies for Using the Mailings 27
4.2 Contents of Counselor Mailings to Participants 28
4.2.1 Personal notes after every call 28
4.2.2 Motivation Enhancement Packet 28
4.2.3 Stop Day Mailer 28

5. BUILD INTEREST, RAPPORT; ENCOURAGE FUTURE CALLS 29

5.1. Indications of Interest/Rapport 29
5.2 Responding to Indications of Lack of Interest or Rapport 30
5.3 Additional strategies for building interest and rapport 31
5.4 Collaborative Agenda Setting 33
5.5 Motivation for Future Calls 33

6. INITIAL CALL 35

6.1 Introductions 35
6.2 Conduct Informed Consent 36
6.3 Confirm that Participant Has Privacy for the Call 38
6.4 Seek Personal Opinions and Insights 38
6.5 Build Interest and Rapport; Build Motivation for Future Calls 38
6.6 Assess Current Smoking Status and Readiness to Stop 39
6.6.1 Querying for current smoking status 39
6.6.2 Querying for readiness to stop 40
6.6.3 Additional Queries 40
6.7 Process Data Items: Current Smoking and Readiness to Quit 41
6.8 Using process data to assign intervention components 42

7. MOTIVATION ENHANCEMENT (ME) CALLS 45

7.1 Introduction 46
7.2 Build Interest and Rapport; Encourage Future Calls 46
7.3 Revisit Consent / Privacy 46
7.4 Seek Personal Opinions and Insights 47
7.5 Assess the Participant's Unique Smoking Situation 47
7.6 Build Importance 49
7.6.1 Additional Strategies for Infrequent Smoking 52
7.7 Build Confidence 55
7.8 Recognizing Readiness to Change 56

7.9 Initiating Phase 2: Strengthening Commitment to Change ... 57
7.9.1 Recapitulation ... 57
7.9.2 Key Questions ... 58
7.9.3 Information and Advice ... 59
7.10 Negotiate an Action Plan ... 59
7.10.1 Set Goals ... 61
7.10.2 Assess Challenges ... 62
7.10.3 Plan for Stopping Smoking ... 62
7.10.4 Arrive at a Plan ... 66
7.10.5 Elicit Commitment ... 67
7.10.6 Setting Short-Term Goals In Preparation For Stopping For Good ... 68
7.10.7 Set a Stop Date ... 68
7.11 Wrap-up the Call ... 69

8. CESSATION SUPPORT: PREPARING TO STOP ... 71

8.1 Assess the Participant's Unique Smoking Situation ... 71
8.2 Initiating Phase 2: Strengthening Commitment to Change ... 73
8.2.1 Recapitulation ... 73
8.2.2 Key Questions ... 74
8.2.3 Information and Advice ... 74
8.3 Negotiate an Action Plan ... 75
8.3.1 Set Goals ... 76
8.3.2 Assess Challenges ... 77
8.3.3 Plan for Stopping Smoking ... 78
8.3.4 Arrive at a Plan ... 82
8.3.5 Elicit Commitment ... 82
8.3.6 Setting Short-Term Goals In Preparation For Stopping For Good ... 83
8.3.7 Set a Stop Date ... 83
8.4 Wrap-up the Call ... 84

9. CESSATION SUPPORT: RELAPSE PREVENTION ... 85

9.1 Introduction ... 86
9.2 Build Interest and Rapport; Encourage Future Calls ... 86
9.3 Revisit Consent / Privacy ... 86
9.4 Seek Personal Opinions and Insights ... 87
9.5 Assess the Participant's Unique Smoking Situation ... 87
9.6 Assess Quit Status / Progress ... 88
9.7 Discuss and Normalize Withdrawal Symptoms ... 89
9.8 Evaluate the Effectiveness of Coping Strategies ... 90
9.9 Examine Slip/Relapse Situations ... 90
9.10 Revise Action Plan as Needed ... 91
9.11 Revisit self-efficacy, motivation, confidence, social support, benefits/costs, self-image.. 92
9.12 Wrap-up the Call ... 93
9.13 Non-attempts ... 94

10. INTERVENTION FOR NON-SMOKERS ... 95

10.1 Revisit Consent / Privacy 96
10.2 Assess Unique Situation 96
10.3 Support the Participant's Choice to be Smoke-free 97
10.4 Enhance Motivation to Help Smoking Peers to Stop 97
10.5 Develop Skills for Helping Smoking Peers to Stop 98
10.6 Wrap-up the Call 99

11. PROTECTION OF HUMAN SUBJECTS 101

11.1 Overview: Human Subjects Protection and Informed Consent 101
11.2 Training in Human Subjects Protection 102
11.3 Potential Benefits of Participation in Matchbreaker Telephone Counseling 102
11.4 Potential Risks of Participation in Matchbreaker Telephone Counseling 102
11.5 HS Study Informed Consent Procedures 103
11.5.1 Goals of Informed Consent Process 103
11.5.2 Parental Consent Procedure 103
11.5.3 Student Consent Procedure 104
11.6 Confidentiality 104
11.6.1 Limits of Confidentiality 105

12. RESPONSE TO IMMINENT AND NON-IMMINENT RISK SITUATIONS 107

12.1 Encountering Participants with Psychological, Mental Health, or Other Problems 107
12.2 Definition of Imminent Risk 107
12.3 Procedures for Handling Imminent Risk Situations 107
12.3.1 Counselor's Response to Indications of Imminent Risk Situations 109
12.3.1.1 Participant requires immediate emergency intervention 109
12.3.1.2 LSP is *NOT* available to take over the imminent risk call 109
12.3.2 SLP's Handling of Imminent Risk Call 109
12.4 Procedures for Handling Non-Imminent Risk Situations 112
12.4.1 Initial Screening of (non- imminent) Homicide/Suicide Risk 112
12.4.2 Counselor Response to Initial Screening for Non-Imminent Risk 113
12.4.2.1 Discovery of imminent risk to self/others 113
12.4.2.2 Indication of non-imminent risk to self/others 113
12.4.2.3 Indication of no risk of harm to self/others 114
12.4.3.1 Participant has recently discussed situation and has follow-up appointment . 114
12.4.3.2 Participant has discussed situation with provider, but not recently 114
12.4.3.3 Participant has not discussed problems with provider & will not agree to 115
12.4.4 Counselor Response to Follow-up Screening for Non-Imminent Risk 115
12.4.4.1 Discovery of imminent risk to self/others 115
12.4.4.2 Indication of non-imminent risk to self/others 115
12.4.4.3 Indication of no risk of harm to self/others 116
12.5 Imminent Risk Documentation and Reporting 116
12.5.1 Refer Suspected Instances of a Reportable Offense to Supervisor 116
12.5.2 Intervention Director's Assessment and Reporting of Reportable Offenses 116
12.5.3 Project Documentation 117

REFERENCES 119

APPENDICES **121**

1. BACKGROUND

1.1 Overview: The Hutchinson Study of High School Smoking (HS Study)

Conducted between 2000 and 2009, the Hutchinson Study of High School Smoking (HS Study) was a randomized controlled trial involving 50 Washington high schools with the high school as the experimental unit. Goals of the trial were to (1) implement the best possible adolescent smoking cessation intervention consisting of individually-tailored telephone cessation counseling, supported by a complementary interactive Web site, self-help materials, and school-based promotion of non-smoking/smoking cessation (e.g., school posters, newspaper ads, student and faculty informational presentations), and (2) use the randomized controlled trial features of the study to rigorously evaluate the effectiveness of this intervention.

Twenty-five of the participating 50 high schools were randomized to the intervention arm of the trial (and participants in those experimental high schools received the intervention during their senior year of high school). The remaining 25 high schools served as no-intervention controls.

The study population consisted of 2,151 smokers and 743 nonsmokers among high school juniors enrolled in the 50 participating high schools. The 50 collaborating schools were randomly selected from 168 Washington State public high schools that met the study's eligibility criteria: they were within 200 miles of Fred Hutchinson Cancer Research Center (Hutchinson Center) and had an enrollment of 100-500 seniors. Nine of the selected eligible schools declined participation because of their current involvements in other similar studies (3 schools), their student information privacy policies (3 schools), or lack of time/energy (3 schools). To maximize the diversity of sampled schools, only one high school per school district was randomly selected.

An in-class survey, administered to students in the Spring of their junior year of high school was used to proactively identify participants. Targeted were all smokers and a selected sample of nonsmokers, including both former smokers and never smokers, who indicated on their baseline surveys an interest in helping their friends who smoke to stop. Nonsmokers were included in this adolescent smoking cessation trial for three important reasons: (1) to help ensure confidentiality of the participants' smoking/nonsmoking status, (2) to reduce the potential of smokers feeling singled out or stigmatized by participating, and (3) to enlist the support of nonsmokers in encouraging and supporting smoking peers who want to stop smoking.

In these 50 high schools, 12,141 high school juniors participated in the baseline survey (93.1% of 13,042 eligible juniors in the 50 collaborating high schools). Ineligible for the survey were 1,188 11th graders who were foreign exchange students, enrolled only in off-campus classes, or unable to read/understand English. Based on their baseline survey responses, 2,894 trial participants (2,151 smokers and 743 nonsmokers) were proactively identified. Excluded as trial participants were 24 smokers and 2 nonsmokers who, in the

baseline survey, declined further contact.

Participants were followed up with 88.8% participation, approximately three months after graduation, to assess their cessation status, quit attempts, reduction in level of smoking, and stage of change.

It is clear from previous studies that a majority of teen smokers want to stop and try to do so. Unfortunately, very few meet with success. Similarly, many nonsmoking teens report that they want to help their friends who smoke to stop. The primary goals of this randomized trial, then, are to rigorously develop and implement (with the dedication and resourcefulness of outstanding telephone counselors) an innovative smoking cessation intervention that will help students who smoke to succeed in stopping, and help nonsmokers support their smoking peers' efforts to stop, and then rigorously evaluate the effectiveness of the intervention in effecting smoking cessation. A positive finding would have significant implications for reducing youth smoking and, ultimately, for improving the nation's health.

This document describes in detail the telephone counseling intervention that was implemented by a team of highly motivated, well-trained counselors with participants from experimental high schools and was evaluated by the trial.

1.2 Intervention Premises

- Smoking is a learned behavior; new learning can lead to new [non-smoking] behavior.
- Smoking acquisition is a continuum of multiple stages from trying to becoming an established smoker.
- Responsibility and capability for stopping smoking lie with the smoker.
- Adolescent smokers are heterogeneous with respect to frequency of smoking, dependence on nicotine, their reasons for smoking, and readiness to stop, etc.
- More beneficial results can be achieved if cessation treatment is tailored to the individual smoker's needs, characteristics, and living/social situations.
- To stop, the smoker must have sufficient motivation to change, and confidence in his/her ability to do so, and must take an active role in changing his/her behavior.
- Because adolescents are not adults, they may face situations that they are unable to change or have great difficulty changing (e.g., living, social, or family situations). These circumstances may significantly impact their motivation and/or their ability to stop smoking. The counselor should be aware of and recognize these when tailoring the intervention to the individual participant.
- Behavior change is a process; successful interventions effect transitions in readiness for change and actions.
- Counseling can help a smoker stop by increasing motivation and commitment to change and by helping the smoker develop necessary skills and confidence for stopping.

1.3 Intervention Goals

1.3.1 Identify for intervention all smokers and a sample of non-smokers among the high school senior class.

1.3.2 Demonstrate an interest in each participant and develop counselor-participant rapport.

1.3.3 Better understand each participant's motivations for/characteristics of smoking and non-smoking, by seeking each participant's frank and personal opinions and insights about smoking and non-smoking, as well as details of his/her unique situations with regard to smoking.

1.3.4 Promote motivation to change and strengthen commitment to stop smoking for each participant who smokes.

1.3.5 Help each participant (who smokes and wants to stop) to increase knowledge and build skills for smoking cessation.

1.3.6 Help each participant (who smokes and is trying to stop) to maintain action/prevent relapse.

1.3.7 Reinforce abstinence from smoking among each nonsmoking participant.

1.3.8 Help each nonsmoking participant build motivation to help smoking peers who want to stop.

1.3.9 Help each interested nonsmoking participant build skills for helping others who want to stop smoking.

1.4 Challenges

Multiple challenges have been identified that must be addressed in any protocol, for a proactive smoking cessation intervention to be effective:

1.4.1 Reaching/making (initial and follow-up) contact with older teens

Older teens are hard to reach, even when an appointment has been scheduled to call at a given time/place. Most are very independent, and often have cars and the freedom that comes with them. Many have jobs and other outside the home activities and interests. As a result, teen cessation help-lines have reported missed appointment rates as high as 50%. Our own pilot experience demonstrates it takes persistent, multiple, and frequent contact attempts to successfully reach trial participants.

1.4.2 Making the goals of our trial salient to trial participants/Obtaining consent

It is anticipated that a substantial portion of the eligible trial participants will be smokers who've not seriously considered stopping, or who may not view their

smoking as a problem behavior that should be changed. Further, many teenagers smoke only infrequently, and do not regard themselves as "smokers." For these participants, the idea of "stopping smoking" has no relevance and they, thus, may have less natural inclination to consent to participate in this study.

1.4.3 Tailoring the intervention to each individual within our target population

Each individual within our target population of 17-20 year olds is unique. The intervention must address each individual's developmental and social needs, and his/her specific situation with regard to smoking, so that each participant will view the program as relevant to his/her specific goals and circumstances.

1.4.4 Scheduling "Quit Preparation" with participants

Pilot experience has demonstrated (and the literature confirms) that adolescents can be quite impulsive with regards to quit attempts, often making the decision to stop smoking quickly with little or no advance preparation. In the pilot study, it was not uncommon for precontemplative smokers to stop smoking between motivation enhancement calls, which resulted in them getting no assistance with planning and preparing for a successful quit attempt via our "preparation" strategies.

1.4.5 Reducing relapse

Relapse rates among adolescent smokers are very high: half of all quitters relapse within one day of quitting. Identifying and addressing the most common relapse situations for this age group, and supporting each participant in the days immediately following his/her quit when the potential for relapse is highest, are of paramount importance.

1.4.6 Counseling participants who use other forms of tobacco (e.g., snuff, chew, cigars)

It is not uncommon for adolescent smokers to also use another form of tobacco, e.g., snuff, chew, cigars, bidis, or clove cigarettes. Also, occasionally smokers will elect to use smokeless tobacco in an effort to stop smoking cigarettes (or vice versa). Substituting one tobacco product for another puts users at increased risk for increasing their smoking.

1.4.7 Counseling participants who do not smoke

Pilot experience demonstrated that nonsmoking teens are reluctant to initiate conversations with friends about their smoking because "smoking is their choice."

1.5 Intervention Strategies

1.5.1 Tailor the intervention to the individual smoker

A premise of the study is that cessation is more likely if the cessation treatment is tailored to the individual smoker's needs and characteristics. Accordingly, counselors will tailor the intervention to the individual smoker's level of motivation to change, as well as on other characteristics of the individual identified in the literature as critical to youth smoking cessation (e.g., frequency/level of smoking, level of nicotine

dependence, experience of any physiological or psychological symptoms of withdrawal from nicotine, concerns about physical changes or other barriers associated with stopping, like weight gain or reduced energy levels, social or peer influences, and self-image as a smoker).

1.5.2 Use the telephone to reach smokers

Past school-based smoking cessation research has identified participant recruitment and retention as difficulties that must be overcome to successfully intervene with adolescent smokers. To address this recruitment challenge, the HS Study proactively identifies the trial population via a school-based baseline survey, and then (after obtaining permission from parents of minors) proactively contacts eligible trial participants via counselor-initiated telephone calls. While this innovative proactive approach has the advantage of reaching many more students than would otherwise seek treatment, it also creates unique challenges: Students who have not sought treatment on their own may not view smoking as a problem, or may not immediately appreciate the value of the proactive call. To meet such challenges, the intervention emphasizes rapport-building between counselor-client, and use of motivational interviewing strategies (both described in detail elsewhere; see sections 5.1 and 7, respectively).

1.5.3 Use proven intervention strategies

The intervention integrates aspects of two proven therapeutic approaches – Motivational Interviewing (MI) and Cognitive-Behavioral Therapy – and uses the stages of change as described in Prochaska and DiClemente’s Transtheoretical Model of Behavior Change (1984, 1986), as a basis for tailoring the intervention to the individual. Each of these approaches, described in more detail in section 1.6, has been used successfully in other settings or with other populations, to achieve behavior change.

1.5.4 Provide useful self-help materials

Self-help materials are an important bridge between clinical and public health approaches to smoking cessation. Using self-help materials offers several advantages: The majority of smokers prefer less intensive, self-help approaches (Fiore et al, 1990). By packaging the components of intensive clinical programs into self-administered forms, the best of the collective intervention expertise can be delivered to millions of smokers at relatively low cost. Printed self-help materials can be customized for different target groups (e.g., for our 17-20 year olds). Importantly, written self-help materials also allow smokers to tailor programs to their specific needs; it gives them control over what they'll do and when they'll do it. Finally, use of self-help printed materials provides participants with something they can keep and refer back to, especially important for those who fail to stop on a single quit attempt.

Of course, self-help materials can be effective only if they are used. It is the role of the telephone counselors to help maximize trial participants' use of relevant self-help materials.

1.5.5 Provide access to cessation materials and encouragement via a Web site

Providing access to a Web site with cessation materials and messages of encouragement extends the reach of the cessation intervention to students who may not be easily accessed by the telephone, or who do not have parental consent to participate in the telephone intervention. The Web site also provides students with 24-hour access to cessation help.

1.5.6 Be (pleasantly) persistent in contacting new and continuing participants

As previously noted, older teens can be notoriously hard to reach by telephone. They lead busy lives, and participation in Matchbreaker is not their top priority. Consequently, we must and will do everything possible (e.g., make multiple and frequent call attempts, blanket all days of the week and times of day with call attempts) to reach participants for the initial and scheduled follow-up calls. Sometimes the most challenging aspect of delivering a skilled telephone intervention is simply reaching the participants on the phone.

1.6 Approaches and Models Used in the Intervention

The HS Study intervention is built on the premise that the responsibility and capability for stopping smoking lie with the smoker. The intervention's tasks, then, are to (1) create a set of conditions that will enhance the participant's own motivation for and commitment to change, and (2) help the participant develop skills and a plan for stopping that will enable him/her to succeed with stopping and maintaining smoking abstinence for the long-term. To accomplish these tasks, the intervention integrates aspects of two successful therapeutic approaches – Motivational Interviewing and Cognitive-Behavioral Therapy – and uses the stages of change as described in Prochaska and DiClemente's Transtheoretical Model of Behavior Change (1984, 1986), as a basis for tailoring the intervention to the individual.

1.6.1 Motivational Interviewing

Motivational interviewing (MI), developed and most comprehensively articulated by Miller and Rollnick (1991, 2002), is defined as "a client-centered directive method for **enhancing intrinsic motivation to change by exploring and resolving ambivalence**." MI seeks to (1) mobilize the client's inner resources to help him/her resolve ambivalence about behavior change and trigger a decision to change (Phase 1), and (2) empathically and strategically support and strengthen the client's decision to change (Phase 2). Miller and Rollnick (1991) propose that the *style* of counselor-client interactions is critical. Hence, MI emphasizes **empathic listening**. Use of MI rules out judgmental, critical, or confrontative communications; these communication styles are thought to increase client defensive reactions, making it less likely that clients will attempt behavior changes.

MI has been shown to increase motivation and reduce problem drinking and smoking among adults, and also has been tested with some success with adolescents in limited settings. For example, telephone cessation counseling incorporating MI goals and strategies have been proven successful for both contemplative adults (Zhu et al, 1996) and precontemplative adults (Curry et al, 1995), and show promise for contemplative teens (Zhu, personal communication).

The HS Study intervention uses a menu of broad MI strategies appropriate for exploring concerns about smoking and specific interviewing strategies (e.g., reflective listening, open-ended questions, summarizing) to guide participants through the counseling session. MI strategies are employed to avoid conflict with clients, support self-efficacy, minimize resistance, and explore cognitive discrepancies. [See also Appendix 26, Motivational Interviewing.] Motivational interviewing is ideal for this project because of its emphasis on promotion of the client's motivation to change and its usefulness for brief telephone counseling. Further, **MI's minimally prescriptive style makes it ideal for use during the teenage years, a developmental stage in which the importance of making one's own decisions is paramount.** By strengthening motivation and commitment to change, MI facilitates each individual's progression through the stages of change and helps the individual maintain abstinence following cessation.

1.6.2 Cognitive-Behavioral Therapy

The cognitive-behavioral approach to smoking cessation focuses on restructuring the client's beliefs about smoking and stopping, and emphasizes the development and implementation of coping strategies and skills (Marlatt & Gordon, 1985). Skills training forms the core of most cognitive-behavioral therapy (CBT). CBT *assumes* client motivation and emphasizes the development, modeling, and practice of new skills for coping with life demands without using addictive substances (Baer et al, 1999). CBT strategies are integrated into the HS Study intervention to help students develop plans to stop, initiate quit attempts, maintain abstinence, and respond promptly and effectively to relapse situations. When motivation lags, MI strategies are revisited in the context of CBT.

1.6.3 Integrating MI and CBT

The HS Study intervention integrates MI and CBT. The two therapeutic approaches complement one another, and share several assumptions about addictive behavior change. First, motivation is critical for achieving behavior change, both prior to and accompanying that change. Whether considered as motivation, readiness, or reinforcement, individuals must have some reason to make and maintain behavior changes. Second, some basic or minimal level of skills is necessary for successful behavior change. Finally, both approaches acknowledge that individuals' motivation and environmental risk will vary over time. Motivation to make and maintain changes is not static, but will ebb and flow with major life experiences as well as the stresses of day-to-day living and other environmental demands.

Counselors will move between motivational and skills-oriented cognitive-behavioral strategies, based on the needs of the individual client, as follows. If a participant is insufficiently motivated to stop smoking, MI strategies are emphasized. This approach is consistent both with MI and with Prochaska & DiClemente's recommendations that interventions be matched to the stage of change of the individual (Prochaska, 1996; Prochaska et al 1992, 1993). Then, once the participant is motivated to change, CBT is provided in response to his/her existing skills deficits and associated need for skills development. The majority of participants will need help developing general skills for stopping, as well as coping skills. Individual students may need help with additional skills, e.g., relaxation techniques, assertiveness, or obtaining support for stopping. It is important to note that CBT does not have to be directive, confrontive, or didactic in manner. Instead, CBT can draw from the student's experience and, therefore, be very consistent with MI's strategies and empathic therapeutic style. The counselor will elicit the student's existing coping skills, help develop his/her natural coping resources, and suggest subtle shifts or adjustments where indicated. Further, counselors will query for student's cognitions and softly challenge maladaptive ones, often by reference to his/her own, other cognitions (Baer, 1999). Finally, counselors will support the initiation and maintenance of motivation by re-visiting and reinforcing motivation with the student throughout the intervention period.

1.6.4 Transtheoretical Model of Behavior Change

The Transtheoretical Model of Behavior Change (so-called because it uses stages of change to integrate processes and principles of change from across major theories of intervention) conceives behavior change as a process involving progress through a series of five **stages**. The stages are described below [Prochaska, 1997].

(1) Pre-contemplation is the stage in which people have *no intention to change* in the foreseeable future, usually measured in the next 6 months. People may be in this stage because they are uninformed or under-informed about the consequences of their behavior, or they may have tried and failed to change and have become demoralized about their abilities to do so. Precontemplators tend to avoid reading, talking, or thinking about their

high-risk behaviors, and are often characterized in other theories as resistant, unmotivated, or unready for therapy.

(2) Contemplation is the stage in which people *intend to change within the next 6 months*. They are aware of the advantages of changing but are also acutely aware of the disadvantages. This balance between the costs and benefits of changing can produce profound ambivalence that can keep people stuck in contemplation for long periods of time. These people are not ready for action-oriented programs.

(3) Preparation is the stage in which people *intend to take action to change in the immediate future*, usually measured as in the next 30 days. They typically have already taken some significant action in the past year. These individuals are ready for a more action-oriented intervention than are contemplators and precontemplators. For example, smokers will be ready to develop their plan for stopping and set a date to stop. Many smokers in this stage may even set short-term goals for themselves, such as stopping for 24 hours, in preparation for stopping for good.

(4) Action is the stage in which people have *made specific overt modifications in their lifestyle within the last 6 months*. Because action is observable, behavioral change has often been equated with action. But in the Transtheoretical Model, action is only one of six stages. Not all modifications count as action in this model. The criterion for action is making a change that scientists and professionals agree is sufficient to reduce risk of disease. Traditionally, total abstinence from smoking has counted as action [Prochaska et al, 1997]. As noted, this stage lasts approximately six months.

(5) Maintenance is the stage in which people have *made a sustained change and are working to prevent relapse*. Maintaining their behavior change comes more easily in this stage; they do not apply change processes as frequently as do people in action. Smokers in this stage are less tempted to relapse and increasingly more confident that they can continue as nonsmokers than they were in the action stage. Based on their research, Prochaska & DiClemente estimate that maintenance lasts from six months to about five years [Prochaska, 1997].

(Termination is a sixth stage that applies to some behaviors, especially addictions. Termination is the stage in which individuals have no temptation and 100% self-efficacy. No matter whether they are depressed, anxious, bored, lonely, stressed, or angry, they will not return to their old, unhealthy behavior as a way of coping.)

The Transtheoretical Model emerged from a comparative analysis of leading theories in psychotherapy and behavior change. This analysis, conducted by Prochaska and DiClemente, identified ten processes of change among the theories. These included consciousness raising from the psycho-analytic tradition, contingency management from the behavioral tradition, and helping relationships from the humanistic tradition. In an empirical analysis comparing self-changers and smokers taking professional treatments for cessation, Prochaska and DiClemente assessed how frequently each group used each of the ten processes [DiClemente & Prochaska, 1982]. Participants said that they *used different processes at different times* in their struggles with smoking, revealing that behavior change unfolds through a series of stages [Prochaska & DiClemente, 1983].

Subsequent work by Prochaska and DiClemente [1984, 1986] has supported the notion that behavior change is a process that involves distinct stage progressions. From these initial studies of smoking, the stage model has expanded in scope to include investigations and applications of a broad range of health and mental health behaviors, including alcohol and substance abuse, anxiety and panic disorders, and cancer screening.

This previous work is important to this current study because it has demonstrated that, to be effective, interventions must be matched, or tailored, to the individual's stage. For example, providing a person who is ready to quit with specific strategies for how to quit can be very effective. However, the same advice will have little effect on the person who has given very little thought to quitting smoking. Therefore, determining the individual's stage of readiness to quit, and tailoring cessation strategies to that stage, will be an important task of the counselor. Delving deeper into Prochaska and DiClemente's work with the Transtheoretical Model, to study **processes of change**, provides some guidance as to *how* the intervention should be tailored to stage.

Processes of change are the overt and covert activities that people use to progress through the stages of change. The processes of change provide important guides for intervention programs because they are methods that effect movement from stage to stage. The ten processes identified by Prochaska & DiClemente as receiving the most empirical support for leading to change are briefly outlined in the table below.

Transtheoretical Model of Behavior Change: Processes of Change

Process	Definition
Consciousness raising	Finding and learning new facts, ideas, and tips that support the healthy behavioral change
Dramatic relief	Experiencing the negative emotions (fear, anxiety, worry) that go along with unhealthy behavioral risks
Self-reevaluation	Realizing that the behavioral change is an important part of one's identity as a person
Environmental reevaluation	Realizing the negative impact of the unhealthy behavior, or the positive impact of the healthy behavior, on one's proximal social and physical environment
Self-liberation	Making a firm commitment to change
Helping relationships	Seeking and using social support for the healthy behavioral change
Counter-conditioning	Substituting healthier alternative behaviors and cognitions for the unhealthy behaviors
Contingency management	Increasing the rewards for the positive behavioral change and decreasing the rewards of the unhealthy behavior
Stimulus control	Removing reminders or cues to engage in the unhealthy behavior and adding cues or reminders to engage in the healthy behavior
Social liberation	Realizing that the social norms are changing in the direction of supporting the healthy behavioral change

Both MI and CBT make use of these processes as strategies for helping individuals progress from stage-to-stage, and all are used in the Matchbreaker intervention.

1.6.5 Matching the Intervention to the Stage of Change

Stage of change is an important consideration when tailoring the cessation intervention to the individual smoker. Action-oriented cessation help, such as how to identify cues to smoke and developing a list of alternatives to smoking in those instances, would be inappropriate and unhelpful to a smoker who has not thought about stopping. Similarly, a smoker ready to set a quit date is not helped by a discussion that explores his/her feelings about smoking. **Individuals can be helped best when the intervention is tailored to meet their individual needs and circumstances.** Thus, identifying the smoker's stage of change, and then matching cessation strategies to the identified stage, are the first steps in tailoring the intervention to the individual. The table below provides a list of stage-matched MI and CBT strategies. **Note how many of these strategies incorporate the processes of change**.

Matching the Intervention to the Stages of Change

Stage	Stage-matched Processes of Change	Stage-matched MI and CBT Strategies
Pre-contemplation	Consciousness Raising<br>Dramatic Relief<br>Environmental Re-evaluation	Motivation-enhancement: **Introduce ambivalence**<br>Eliminate pressure, emphasize choice<br>Explore self-image, values, goals<br>Explore/build importance, explore feelings and opinions about smoking/stopping, discuss pros & cons of smoking/stopping (to identify areas of discrepancy between behavior and self-image, values & goals)<br>Provide normative feedback (e.g., for "chippers," effects of infrequent smoking)<br><br>Avoid: Assuming participant is against change
Contemplation	Self Re-evaluation<br>Social Liberation	Motivation-enhancement: **Resolve ambivalence in favor of change**<br>Develop discrepancy, discuss pros & cons of smoking/stopping<br>Reflect smoker's ambivalence and help "tip the scale"<br>Elaborate smoker's reasons for stopping and desire for self-control to increase salience of intrinsic motivation<br>Build confidence<br>Evoke self-motivational statements<br>Strengthen commitment to change<br><br>Avoid: Assuming health will motivate change, Accepting ambivalence
Preparation	Self-liberation<br>Helping Relationship	Preparation: **Prepare for change**<br>Determine/reinforce reasons for stopping<br>Develop plan for stopping<br>Identify potential challenges and coping strategies<br>Develop strategies for overcoming barriers<br>Identify social support<br>Prepare and practice strategies/self-talk<br>Set a stop date<br><br>Avoid: Assuming participant needs little help

Stage	Stage-matched Processes of Change	Stage-matched MI and CBT Strategies
Action	Contingency Management<br>Helping Relationship<br>Counter-conditioning<br>Stimulus Control	Cessation support: **Prevent relapse**<br>Ask when student last smoked<br>Discuss immediate benefits of stopping<br>Assess success coping with early physical and social stimuli<br>Debrief smoking situation / Normalize any slips<br>Discuss alternative activities<br>Identify rewards<br>Discuss difficult situations, revise plans for coping as needed<br>Develop identity as a nonsmoker<br><br>Avoid: Assuming participant is stable too early
Maintenance	Contingency Management<br>Helping Relationship<br>Counter-conditioning<br>Stimulus Control	Cessation support: Validate change, promote continued growth<br>Ask how things are going<br>Debrief smoking situation / Normalize any slips<br>Normalize relapse (e.g., emphasize several tries are common, learning from this try, and importance of "keep trying")<br>Teach skills in seeing learning potential in setbacks<br>Maximize environmental support<br>Ask for pointers for other smokers<br>Discuss general well-being, pleasurable activities<br>Provide motivations, skills for helping others

2. THE PROACTIVE TELEPHONE COUNSELING INTERVENTION

2.1 Smoking Acquisition Among High School Students

Just as quitting smoking is a process involving transitions through stages, so has smoking onset been defined. Understanding the smoking onset process is relevant to the HS Study because many participants who smoke will still be in the process of becoming a regular smoker. Indeed, in the HS Study, counselors will encounter participants at every stage in the smoking onset process. The table (below) describes the stages of smoking onset (Mayhew, Flay & Mott, 2000). Smokers start transitioning through these stages at different ages: some try their first cigarette at 8 or 9, while others may not smoke their first few puffs until high school. But nearly all (>90%) begin smoking before age 18.

The time interval from the initial try to regular smoking takes an average of two to three years, with considerable interval variation among individuals (Leventhal, Fleming, Glynn 1988). McNeill (1991) found in a prospective study that of those experimenting with cigarettes, approximately half were smoking on a daily basis within a year. Similarly, the prospective HSPP trial found that of those smoking monthly or less as high school seniors, 40% were smoking daily two years later.

In the HS Study, our participants who smoke infrequently are likely to be "triers" and "experimenters," and the participants who smoke regularly are the "regular" and "established" smokers, as defined below.

Stages of Smoking Onset

Stages of Smoking Onset	Definition	Possible HS Measurement
Non-smoking – precontemplation stage	Non-smoker and does not intend to smoke	"susceptible to smoking" (Pierce et al, 1996) "Will you smoke in the future?" (definitely not)
Non-smoking – preparatory stage	Nonsmoker and intends to smoke. Is developing attitudes and beliefs about smoking; susceptible to peer pressure.	"susceptible to smoking" (Pierce et al, 1996) "Will you smoke in the future?" (any but definitely not)
Trier	Stage when adolescents try their first cigarettes. May say they are nonsmokers or tried but quit	# cigarettes smoked in lifetime<br>(few puffs to 20 cigs)
Experimenter	Stage when adolescents gradually increase the frequency of smoking and the variety of situations in which they smoke. Adolescents at this stage emphasize the positive aspects of smoking and few negative aspects. They're not totally committed to smoking in the future and are still deciding whether its for them.	Smoking frequency or<br># days in the last month you smoked >/= 1 cigarette? (<10)<br>and<br># cigarettes smoked in lifetime<br>(21-100 cigs)

Stages of Smoking Onset	Definition	Possible HS Measurement
Regular smoker	Adolescents progress beyond sporadic smoking to smoking on a regular, though still infrequent basis. Some will smoke every weekend at parties or most week days on the way to/from school. These youth are not smoking every day and are not smoking at very high rates.	Smoking frequency or<br># days in the last month you smoked >/= 1 cigarette? (10-19)<br>and<br># cigarettes smoked in lifetime (>100)
Established (daily) smoker	Adolescents smoke daily or nearly every day, may experience dependence and find it difficult to quit. In this stage, both psychological and biological factors influence maintenance of smoking behavior.	Smoking frequency or<br># days in the last month you smoked >/= 1 cigarette? (20-29, every day)<br>and<br># cigarettes smoked in lifetime (>100)

2.2 Overview of the Proactive Telephone Counseling Intervention

All trial participants (TPs) in the study will receive a protocol-directed series of counselor-initiated calls (from 1 to 10, depending on participant's interest), supplemented with selected and personalized mailings. Figure 1 outlines the proactive telephone counseling sequence for trial participants. Through conversation and strategic questioning, the counselor will determine the participant's smoking status and, if he/she smokes, stage of change. The counselor uses these pieces of information to match the intervention to the individual by stage, as shown in Figure 1 and described below:

All participants, regardless of their smoking status and readiness to quit, will be invited to share their frank and personal opinions and insights about smoking and non-smoking, as well as details of their unique situations with regard to smoking. (Please refer to section 6.4 for details.)

Participants who smoke (i.e., smoke >/= monthly) **but are not currently thinking about stopping** (i.e., are in precontemplation or contemplation stages) will receive up to three Motivation Enhancement calls, depending on their interest, designed to help them build motivation and commitment for change. Counselors will use motivational interviewing strategies, tailored to the individual participant, to help the participant explore and resolve his/her ambivalence about stopping. When it is established that the smoker is ready to stop (preparation stage), the counselor's focus shifts from motivation to preparation and transitions to cessation support. (Please refer to section 7 for details.)

Participants who smoke and want to stop (preparation stage) will receive help with meeting this goal. The counselor will use cognitive behavioral therapy, with an MI communication style, to help the participant prepare for stopping and to take action to do so. Specifically, the counselor will help the participant (1) develop a plan for stopping, (2) identify potentially difficult situations or smoking triggers and suitable coping skills, and (3) set a stop date. On the day that the participant stops smoking (action stage), the counselor begins a series of up to six **Cessation Support** calls (depending on participant needs and interest) aimed at supporting the participant's efforts not to smoke and to prevent relapse. (Please refer to section 8 for details.)

Participants who do not smoke (i.e., never smokers and former smokers who have not smoked in the past 3 months) will receive a one-call **nonsmoker intervention** (with the option of a second call at their request) aimed at (1) reinforcing their choice not to smoke, (2) motivating them to help their smoking peers who want to stop, and, if they are so interested, (3) helping them develop skills for doing so. (Please refer to section 9 for details.)

In all telephone calls and written communications, counselors will pay special attention to development of rapport, building trust and a helping relationship, and encouragement of the student's continued participation in the Matchbreaker calls. See additional information about the importance of building rapport and a helping relationship, and strategies for doing so, in section 5.

Figure 1. Proactive Telephone Counseling Sequence

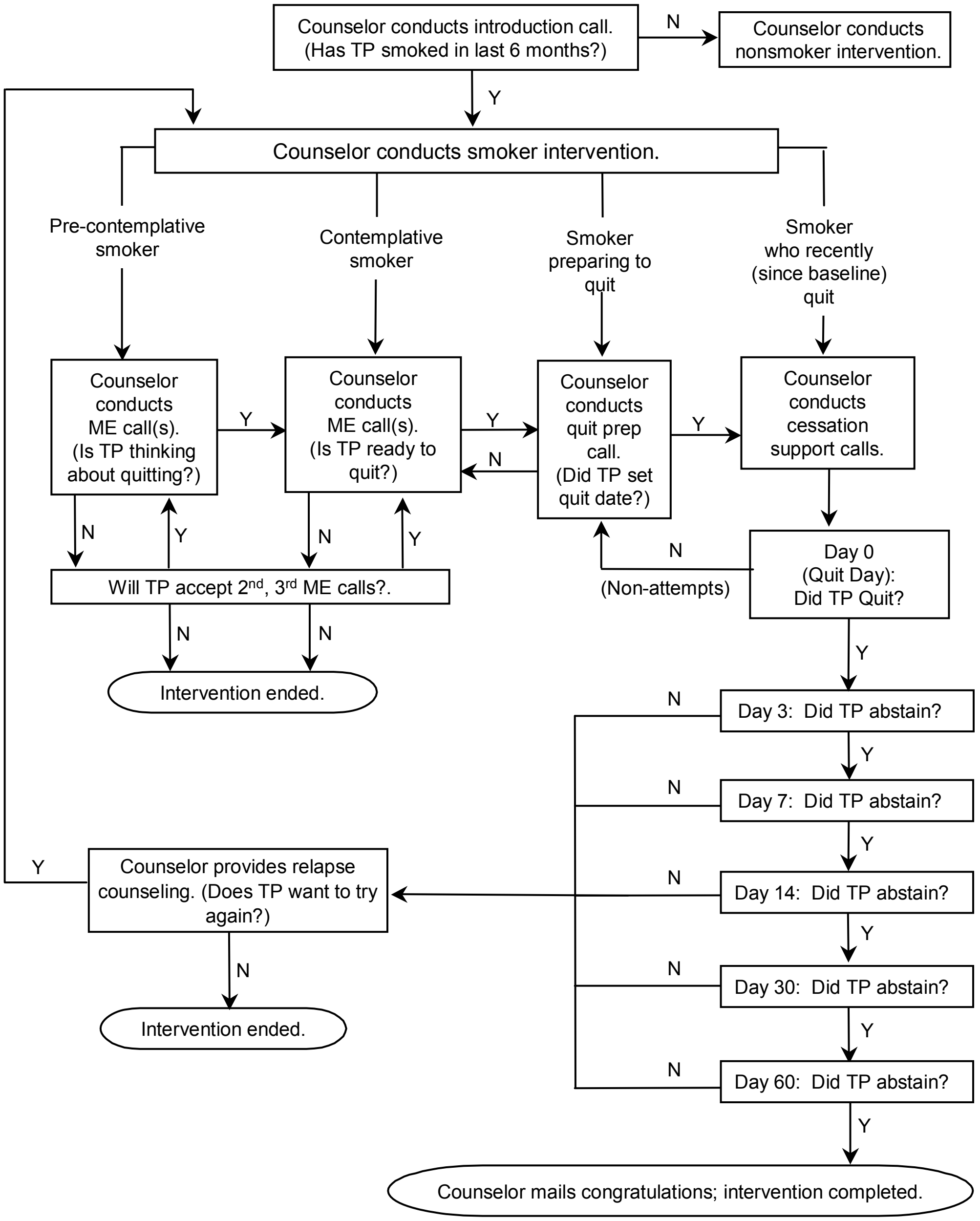

3. SUCCESSFULLY CONTACTING TRIAL PARTICIPANTS

3.1 Challenges

Trial participants can be difficult to reach by telephone, even when an appointment has been scheduled to call at a given time/place. Older teens are very independent; they often have their own cars and the freedom that comes with them. They have jobs and many school- and non-school-related outside-the-home activities and interests. Some high school seniors also may only attend school for part of a day, going in late or leaving early, because they don't need a full day schedule to graduate. Others may take all or part of their senior year classes at a community college. Still others will have dropped out of school entirely; many in this group are faced with some major life challenges. As a result of older teens' full and sometimes erratic schedules, other teen cessation help-lines have reported missed appointment rates as high as 50%. Our own pilot experience demonstrates it takes persistent, multiple, and frequent contact attempts to successfully reach trial participants.

3.2 Contact Goals

3.2.1 Trial goals for parent and student agreement to participate

90% of eligible trial participants will be over 18 or have parent permission to participate in the HS Study.
96% of those participants will be reached by telephone for the initial call.
99% of those reached by phone will accept the initial counseling call.
87% of those accepting the initial counseling call will agree to receive subsequent intervention calls.

3.2.2 Goals for contacting trial participants

On average, it takes approximately 10 call attempts (0.62 minutes per attempt) to successfully reach and complete a call with a participant. Completing a participant call takes an average of 16 minutes per completed call. Counselors need to use their time efficiently, to maximize their number of call attempts. For management purposes, all eligible participants are randomly assigned to one of six groups. Data Operations staff will obtain parental consent, if required, before groups are released to counselors. On the first Tuesday of each month, "Round 1" of each group (all eligible participants with consent) will be assigned to counselors (Round 1 = approximately 20 eligible participants). Approximately six weeks later, "Round 2" will be assigned to counselors (Round 2 = approximately 8 eligible participants). This schedule is designed to enable counselors to work most productively, always having students to call. It is anticipated that in an hour, counselors can make at least 10 call attempts, and successfully complete a call, including writing call notes and the student's follow-up letter.

3.3 Contact Strategies for Initial Calls

In all calls, the counselor's aim is to call at the time when the participant is most likely to be available and receptive to a call. Most of the participants will be in school during weekday mornings and afternoons, making late afternoons and evenings the best time for participants.

To contact a difficult-to-reach TP, it may be necessary to try calling during multiple different times of the day and night, both on weekdays and weekends. When attempting to reach a TP for the first time, counselors will make at least two contact attempts during each of the following call periods: (1) weekdays, after school (mid to late afternoon, e.g., 2:00 – 4:30 p.m.), (2) weekdays, early evening (e.g., 4:30 – 7:00 p.m.), (3) weekdays, late evening (e.g., 7:00-9:00 p.m., or later with parental permission), (4) Sunday evenings (e.g., 5:00 – 9:00 p.m.), (5) weekday mornings or early afternoons (to catch those participants not in school during regular school hours), (6) Sunday afternoons (e.g., 12:00 – 5:00 p.m.), and (7) Saturday mornings (e.g., 10:00 – noon),. No outer limit will be placed on the number of call attempts to reach a participant.

Counselors should start with the days/times that have been most productive in terms of reaching TPs, e.g., weekday afternoons and evenings. It will only be necessary to blanket for hard-to-reach TPs.

> *Tip:* "Make friends" with parents. Ask them about best times to contact their son/daughter. Good relationships with parents will pay off not only when attempting to contact the participant for counseling, but also later when locating him/her for outcome data collection.

If an initial contact can not be made after blanketing all time periods on weekdays and weekends with at least two contact attempts per call period for an entire week, then it can be helpful to move on to other new contacts and wait two weeks before re-attempting contact with the hard-to-reach TP. In the intervening 14 days, it is possible that something will change in the TP's life/situation that will make him/her easier to contact at next attempt (e.g., sports season has concluded, no more drama or marching band practice, different work hours, etc.). Alternately, easily reaching a TP on the initial call is no guarantee that he/she will be easy to reach for follow-up because something may change that makes contact more challenging, e.g., a new job, extra rehearsals for an upcoming performance, start of a sports season, etc.

> *Tip:* When all avenues for contacting a participant have been exhausted, writing him/her a letter, inviting him/her to participate in the study by calling our 800 number, can sometimes generate a response. The letter should be inviting, convey no blame for the TP not being reached, and should say who we are and what we have to offer.

3.4 Contact Strategies for Follow-up Calls

Whenever possible, follow-up calls are made by appointment. If calling participants during the prescribed target window of 10 days to 3 weeks later (which will work for a majority of participants), appointments are helpful for reminding both the counselor and the participant. The counselor will make every attempt to call the TP on the date/time scheduled for the follow-up call. ***Goal:* We always do what we said we would do. We keep the appointments we make.**

> *Note:* Appointments are recommended but are not required when making scheduling follow-up calls more than 8 weeks out, e.g., calls to resistant or unengaged participants who would benefit from and prefer a longer period between calls (although the counselor should make an appointment with him-/herself for the call back).

Furthermore, when making appointments, or when TP misses his/her appointment, counselors will ask about and note "best times" to call back and then always try to call within the "best time" parameters provided by TPs or parents.

When making an appointment for the next call, the counselor will prompt the participant to think about any work, sports, or rehearsal schedules; this might help increase the likelihood of setting a time for an appointment that will be met by the participant.

3.4.1 TP misses call appointment

If TP misses his/her call appointment, counselor will call back during a "best time to be reached."

If we have no recorded "best time to be reached," the counselor will start blanketing all time periods on weekdays and weekends with at least two contact attempts per call period for one week, in order to reach the participant.

If the participant is in a quit attempt and receiving cessation support, call attempts will be even more aggressive in order to stick more closely to the relapse sensitive call schedule.

> *Participants hard to reach? Try calling at all these different times.*
>
> **Call Periods:**
> (1) Weekdays, after school (mid to late afternoon, e.g., 2:00 – 4:30 p.m.)
> (2) Weekdays, early evening (e.g., 4:30 – 7:00 p.m.)
> (3) Weekdays, late evening (e.g., 7:00 – 9:30 p.m. or later with parental permission)
> (4) Sunday evenings (e.g., 5:00 – 9:00 p.m.)
> (5) Weekday mornings or early afternoons (e.g., 9:00 a.m. – 2:00 p.m.)
> (6) Sunday afternoons (e.g., 12:00 – 5:00 p.m.)
> (7) Saturday mornings (e.g., 10:00 a.m. – noon)

3.4.2 Leaving voicemail messages

(add guidelines for leaving voicemail messages, e.g., without breaching confidentiality, how many to leave, what if voicemail message doesn't identify the identity of the family?)

3.4.3 Trying different phone numbers

The HS Study Telephone Intervention (TI) Master Form will include a list of all contact numbers for each participant and his/her family known to the Study (as supplied by the participant on the baselines survey's contact information sheet). Associated with each phone number is a statement about where the number came from, e.g., Family phone. Counselors are free to ask participants if there is another number they prefer we call on, e.g., private line in their room or their own cell phone. If repeated attempts on the participant's preferred number go unanswered, try calling one or more of the other numbers.

If none of the telephone numbers appear to be valid, flag the TP for tracking and the Data Operations Staff will track the TP and obtain a new phone number.

3.5 Call Timing for Motivation Enhancement Calls

When calling to build motivation for change, the timing between calls must balance between the urge to "strike while the iron is hot," i.e., get all the calls in while the participant seems to be engaged and interested, and the desire to not make the participant feel we are pushing our agenda on him/her. Scheduling follow-up calls requires counselor judgment. When deciding on best timing for a follow-up call, then, counselors should consider:

(1) The participant' s level of receptivity and rapport
(2) Degree of progress being made by the participant
(3) Needs of the participant

3.5.1 Target windows for motivation enhancement calls

Ideally, most participants will receive their **next ME call between 10 days and 3 weeks following the call just concluding**. For those participants who are really resistant and unable or unwilling to benefit from a call during that target window, consider not calling that student back for 8-12 weeks. Waiting allows time for things to change for the participant, either with regard to his/her situation, smoking behavior, or motivation to fully participate in the telephone conversations.

When scheduling the call, counselor's will consider the criteria above, as well as participant preference. Examples:

(1) Participant is engaged and seems into the calls, doing some serious thinking about his/her smoking. In this case the counselor would want to try to schedule the next call early in the target window.

> *"It sounds like you're interested in [motivation]. I'd like to look up more information on that. How about if I do that and call you back week after next? Would that be helpful to you? [pause for reply] Great! Is this same time good? Okay, I'll plan to call you on Wednesday, Oct. 23 at 7."*

(2) Participant is engaged and receptive to additional calls, but seems to be strongly precontemplative; this participant might benefit from a little extra time between calls. Try to call him/her back late in the target window, or even a week or so later.

"If it's okay with you, I'd like to send you a booklet that has some info on just what we talked about today--I'll note the pages for you. Perhaps I can call you again next month and see what you think? Would that work for you?

(3) Participant is neither receptive or resistant, but has expressed concern about a particular smoking situation. Offer to call back at a time most helpful to him/her.

"I can see that is a concern for you. If it would be helpful, I can call you back next week and we can talk about options for relieving stress. Would you like that?"

(4) Participant has agreed to participate, but doesn't seem engaged and is resistant to moves directed towards discussing his/her future tobacco use. Consider calling this person back during the late window, in 8-12 weeks. Give this participant a window; don't attempt to make an appointment. (This gives the counselor the flexibility to start calling a little early, in order to reach the participant sometime during the agreed upon follow-up window.)

"Sounds like you're just not that interested in talking about this right now. How about if I call you back in a couple of months or so?"

> *Note:* Never push a next call on a participant. Give the participant space to make his/her own decision; always try to leave the door open for future contact. Participants may decline calls whenever they wish.

3.6 Call Timing for Cessation Support Calls

Sixty percent of smokers relapse within the first week of quitting [Zhu & Pierce, 1995]. Cravings and withdrawal symptoms are typically most intense in the two weeks following the last cigarette; thereafter, the cravings are weaker, and withdrawal symptoms are mild or even non-existent. This schedule may be helpful not only to individuals who are nicotine dependent, but also for those smoking has become a behavioral habit, a social ritual, or provides psychological relief.

To support participants during the crucial period immediately following their stopping smoking and beyond, on the participant's stop date he/she will begin an intensive, relapse-sensitive schedule of telephone counseling. These Cessation Support calls are aimed at supporting the participant's efforts to remain abstinent and prevent relapse. Following this schedule, participants may receive *up to six* cessation support calls, along with follow-up counselor mailings, **scheduled over 60 days beginning on their stop date and scheduled for Days 0, 3, 7, 14, 30, and 60.** This relapse-sensitive schedule is designed to support participants during the period in which [physical and psychological] withdrawal symptoms and cravings are strongest, and the probability of relapse is greatest, thus optimizing the effect of counseling.

Calls may be adjusted to the participant's schedule as needed – for example, participants with high risk situations coming up, e.g., weekend parties, may want a call just before that event, or participants may miss appointments.

If they wish, TPs with e-mail will also have the option to also receive messages of encouragement and support on Days 1, 2, 5, 10, 12, and 20.

3.6.1 Timing for participants who stopped since the last call

The literature on adolescent smoking cessation shows that adolescents in the early stages of change seem to move prematurely into action (Pallonen, 1998). This was also our experience in the pilot study: it was not uncommon to complete an ME call with a precontemplative smoker and call back three weeks later to discover that the participant had stopped smoking shortly after the ME call. Pallonen found that precontemplative and contemplative adolescents who quit impulsively relied more on behavioral processes in the precontemplation to contemplation and contemplation to preparation stage transitions, rather than applying experiential processes, which more appropriately (in the early stages of change) help prepare for a change.

If the counselor discovers the participant stopped smoking since the last Matchbreaker call, the counselor's goal should be to "map" the participant's next scheduled calls onto the relapse sensitive call schedule. Examples:

(1) Participant stopped smoking just in the past week. Because the participant stopped so recently, the counselor should schedule the next call in a week, mapping onto day 7 in the schedule, and follow the schedule out from there.

(2) If the participant stopped smoking two weeks or more before the current call, map onto day 14 and follow the schedule from there, so that the participant can receive some cessation support.

Pallonen's work (described above) can help guide the counselor in deciding how best to help those participants who stopped several weeks prior to our follow-up call without quit preparation assistance. When applying the mapping strategy (above), counselors are urged to consider other factors important to the participant, in addition to length of the quit attempt, when deciding how to provide optimum support to the TP. Considerations: types of cessation strategies the TP has used to stay quit; types of challenging situations or smoking triggers he/she has encountered, and how his/her coping strategies have worked in these situations; his/her reasons for stopping; and his/her strength of commitment for stopping for good. In other words, try to identify whether the participant's commitment to change is strong, and to build/strengthen (later better than never) his/her skills for stopping smoking/staying stopped.

3.7 Tracking Counselor's Calls

The Intervention Manager is responsible for monitoring counselors' work, including weekly monitoring of calls/call attempts, to insure that the intervention is being implemented as per protocol.

3.8 Flexible Counselor Work Schedule for Calling

Counselors are allowed to work flexible schedules to be free to call participants when they are available to receive intervention calls. Therefore, counselors can set their own daily work schedules (with supervisor approval). For example, a counselor may have some participants who prefer being called on Saturday afternoons. The counselor can choose to work a Saturday afternoon shift in lieu of a regular weekday shift in order to reach those participants at the time they most prefer.

3.9 General Tips for Telephone Contacts

Whoever it is you are calling, they have something you want. So, **participants are your customers and all rules about treating customers well apply**.

(1) Treat everyone with sincerity, respect, and appreciation.

(2) Always be polite, professional, honest and friendly

(3) Match your style of speech to the person with whom you are talking (e.g., if the TP is a very quick, fast speaker, match as much of it as you can; alternately, if the TP has a very slow, articulate, sensible voice, match that as best you can. Do not use the slow, articulate voice to speak to the quick, fast person.)

(4) While the person you're talking with is sizing you up in the first 30 seconds on the phone, you need to also size up him/her. You want to do a quick "read" on the type of person you're talking to so you can meet his/her needs accordingly. You won't always read the situation right, but you'll also be surprised at how often you do.

(5) Be human, not robotic. Realize that each person you speak with has a life, probably a dramatic life, and your call may not be a priority. What you want to do is find commonality so that even though they don't know you, there is something familiar about you. That familiarity may be as simple as your tone of voice (back to matching style) or the TP being familiar with the Hutch, or remembering the Survey of High School Juniors (e.g., students may remember the saliva sample/cotton dental roll, the mint, free Fred Hutch pencil).

(6) If the TP you are calling wants to tell you about something, related to your mission or not, **listen to him/her and be kind and non-judgmental in your response**. After listening and responding, bring the conversation back to your reason for calling.

(7) Always approach each contact knowing there's a strong possibility that you will call that person again. Having this mind-set is very powerful because it guides you to always try to have a pleasant conversation and makes you want to leave the call on good terms.

(8) Always remember that people are generally nice. If they sound curt, rushed, rude, etc., assume you caught them at a down moment – do not put it on yourself. Realize that, at the moment we are calling, we may be the least of their priorities. Be sensitive to this and try to accommodate them before the door gets slammed.

(9) Realize that even well before counselors make their first calls, a lot of effort has gone into establishing rapport, e.g., through the baseline survey activities and the design and implementation of all consent contacts.

3.10 Participants Who Are Drunk or High

Counselors should not attempt to deliver the intervention to participants who are drunk or high when reached on the telephone. In such instances, it is better to quickly and politely end the call and try to call back at another time.

> *"Thanks for taking these few minutes to talk with me today; I think I have all I need for now. I'll try and call you back in a few weeks, if that's okay with you."*

3.11 Calling a participant whose name is difficult to pronounce

When calling participants, it is not uncommon to come across a name that may be difficult to pronounce. Many of the high schools in the study serve multiple immigrant populations, so counselors will be challenged to pronounce unfamiliar names. Counselors are encouraged to check available Internet resources for pronunciation keys (e.g., http://www.csupomona.edu/~faculty_computing/lab/Pronunciations/Pronunciation/; and http://www.geocities.com/Athens/Aegean/2444/pronounce.html). Other than that, they are to proceed with calls, approaching difficult to pronounce names using the "brave, friendly, give-it-your-best-shot, apologizing-if-you-get-it-wrong" approach. Specifically, counselors should express humility as they try to pronounce the name and express interest in learning from the participant the correct pronunciation of his/her name. Counselors are to record how to correctly pronounce the name in the "pronunciation field" of the participant's call record and use the correct pronunciation in all future contacts with the participant.

Our experience, and the experience of multiple other PHS projects, is that participants are generally very kind about helping the telephone staff learn to pronounce the participants' names correctly and are pleased that telephone staff want to do so. It creates an overall positive interaction between the participant and the project/FHCRC.

4. COUNSELOR MAILINGS TO PARTICIPANTS

Counselors will follow-up each call to a participant with a mailing to him/her (with the participant's approval and to an address of his/her choice). Content of each mailing will be tailored to the needs and interests of the participants. All will include a personal note from the counselor to the participant. Other self-help materials may be mailed following specific calls in the protocol sequence, or as needed by individual participants.

Self-help materials are an important bridge between clinical and public health approaches to smoking cessation. The majority of smokers prefer less intensive, self-help approaches (Fiore et al, 1990). By packaging the components of intensive clinical programs into self-administered forms, the best of the collective intervention expertise can be delivered to millions of smokers at relatively low cost. Another advantage of printed self-help materials is that they can be customized for different target groups (e.g., for our 17-20 year olds). Written self-help materials also allow smokers to tailor programs to their specific needs; it gives them control over what they'll do and when they'll do it. Finally, use of self-help printed materials provides participants with something they can keep and refer back to, especially important for those who fail to stop on a single quit attempt. Of course, self-help materials can be effective only if they are used. It is the role of the telephone counselors to help maximize their use by trial participants.

4.1 Strategies for Using the Mailings

Counselors should tie their conversations to the "tools" provided in the mailings whenever possible. It is very helpful to include a "post-it" note on the front of the ButtsOut books, directing the participant to information the counselor knows will be particularly relevant. Example:

> *"Joe: Take a look at "strategies cards" on the page I flagged for you. You might want to keep the "Stress" card in your wallet!"*

Using the "ButtsOut" books as opening strategies during subsequent calls can be very helpful for both generating conversation and reminding the participant of the great resource he/she may not have used yet. Example:

> *"Last time we talked you mentioned you'd like to try other things besides smoking to relieve stress. Did you get a chance to try the stress-relievers listed in "ButtsOut"? How did those suggestions work for you?"*

Counselors can gently promote and encourage use of the materials. We know these can be helpful to participants. Example:

> *"I think this would be a helpful tool for you. I'd like to know what you think."*

4.2 Contents of Counselor Mailings to Participants

4.2.1 Personal notes after every call

Counselor's will mail a personal note to participants following every call. At a minimum, the post-call mailings to participants will contain: (1) a short, friendly note summarizing (a) the topics discussed in the call, (b) additional motivational or confidence-enhancing messages, (c) the participant's planned next steps, and (d) the date and time of the next call appointment; and (2) a wallet card with the counseling 800# and the Matchbreaker Web site address.

If a participant completes three ME calls and still does not want to stop smoking, the counselor has a final opportunity to provide a strong, personal message in the TP's follow-up letter. Using the "MI in a box" strategy, these final follow-up notes should convey the following messages:

(1) Care for the participant and concern regarding continuing smoking
(2) Respect and acknowledgment of the participant's independence, autonomy (i.e., right to make his/her own decision)
(3) An offer of further help. Example:

"Dear John, I've really enjoyed our conversations these last few weeks. You've done a lot of careful thinking about your smoking, and given me some great feedback for our research. I guess you know that I'd like to see you just walk away from smoking, but it's not my decision. It's up to you. But, if you decide later on this year that stopping smoking is something you want to do, I'd love to talk to you again, and offer any help or assistance that you'd like. All the best to you, John."

4.2.2 Motivation Enhancement Packet

Following the first motivation-enhancement counseling call, mailings to participants will also include (1) a cessation-motivation booklet ("ButtsOut," Vol. 1) that supplements and reinforces counselor- and Web-supplied information, and (2) a Matchbreaker matchbook notepad.

4.2.3 Stop Day Mailer

Smokers making an attempt to stop smoking will be sent a "Stop Day Mailer" containing (1) a personal note from their counselor containing (a) messages of encouragement and support, (b) a brief outline of their "plan," (c) the date and time of the next call appointment; (2) a smoking cessation self-help booklet ("ButtsOut," Vol. 2); (c) a wallet card with the counseling 800# and the Matchbreaker Web site address; and (d) a pack of chewing gum, a small rubber squeeze ball, a water bottle, and a frisbee.

5. BUILD INTEREST, RAPPORT; ENCOURAGE FUTURE CALLS

We list “interest/rapport/encouragement” as a goal for every call, but they are much more: Interest, rapport, and encouragement of future calls are essential considerations throughout *every* call and with *every* contact with a participant. Building rapport and interest starts with every *“Hello,”* – **it is the most important thing a counselor can develop at the outset of his/her contact with a participant**. The counselor’s ability to quickly establish interest and rapport with participants will be key to keeping participants on the line and getting them to accept future calls from our study.

As with all counselor-client settings, it is important to build the client-counselor relationship, establish trust, and open the lines of communication. But this goal is particularly important to the HS Study *because of its proactive approach to contacting high school students* (i.e., initiating contact with eligible participants). Establishing the student’s interest in participating and developing a rapport between the TP and the HS Study began with (1) the data collectors’ interactions with students during baseline data collection, and (2) carefully chosen wording of the informed consent mailings to potential TPs. Every counselor-client contact will build on those initial contacts.

Rapport sets the stage for everything that comes after by helping establish a feeling of respect, safety, interest and warmth between the counselor and the participant. Once a participant feels such rapport, he/she is much more likely to agree to participate in the intervention and do so in an active and collaborative fashion. Thus, establishing rapport helps both to recruit more participants, and to work more productively with each of participant over the course of the intervention.

In order to facilitate these goals, counselors must assess the level of interest and rapport in the first few moments of contact during the initial call. This assessment is a qualitative decision that must be considered in light of the participant’s immediate reaction to the counselor’s first words of introduction and conversation, and the subsequent responses that immediately. Within this time frame, counselors should evaluate the participant’s verbal tone, and the quality of any spontaneous comments or moments of silence.

5.1. Indications of Interest/Rapport

If a participant’s verbal tone, words, or silence is characterized by any of the following adjectives, there is probably adequate initial interest/rapport: bright, cheerful, excited, curious, thoughtful, reflective, considerate, and open.

Initial interest/rapport may be lacking if a participant’s verbal tone, words, or silence can be described by any of the following adjectives: wary, skeptical, impatient, irritated, frustrated, bored, worried, or angry.

Often participants will not provide obvious indications of their interest or feelings of rapport. At these times, counselors must rely upon their own gut instincts or intuition to guide their next steps.

5.2 Responding to Indications of Lack of Interest or Rapport

If a participant appears negative or “neutral to negative,” the counselor should consider trying to quickly build rapport with the participant before proceeding further. Sometimes the solution is as simple as taking a few extra minutes to chat with the participant. Showing a sincere interest in the participant and his/her interests can be a good place to start.

It also can be helpful to take a few minutes to more completely describe the goals of the study, particularly those related to the goal of better understand the motivations for/characteristics of high school smoking and non-smoking, by seeking participants' frank and personal opinions and insights about smoking and non-smoking. The pilot study demonstrated that students like to know that they’re helping with research. Emphasizing how they can help the research can help break down defenses and overcome skepticism.

The counselor’s wording and sincere tone can also help put the participant at ease and let the participant know that his/her goals, and not the counselor’s, will direct what is discussed in the calls.

Other rapport-building techniques include being respectful of the participant, demonstrating empathy, actively listening, and demonstrating acceptance of the client’s current stage of motivation to stop. If the participant was a smoker at baseline who expressed interest in having help with stopping, or if he/she was a nonsmoker or former smoker who expressed interest in helping others to stop, use this information to build a connection with the participant.

If a student seems reluctant to commit to further participation, the counselor won’t push. The goal is to eliminate pressure and emphasize choice so that an opportunity for a second call is maintained. Example:

> *“Sounds like talking about smoking isn’t something that interests you right now. How about if I send you some information about what we talked about today, and I touch base with you in a few weeks to see what you think? When would be a good time for me to call?”*

Keep in mind that some student’s reluctance or disengagement may not be a reflection of their feelings about participant in Matchbreaker, but rather may be due to other circumstances in their lives. Giving them some time before calling back may make a big difference. Before assuming a student’s disengagement is due to lack of interest, give him/her a chance for a later call back. When doing so, be sure not to put pressure on the student to accept another call.

> *“I’m thinking that I may have caught you at a bad time. Instead of taking up your time now, how about if I try calling you back in a couple of months?”*

If the student declines any further telephone contact during the first call, or a subsequent call, the counselor may offer to send self-help cessation materials (to the address of the participant's choice), refer the student to the Web site, and invite the student to call back any time during the senior year if he/she changes his/her mind about telephone counseling. **The strategy here is to leave many doors open for students who may later decide to learn more about stopping smoking.**

5.3 Additional strategies for building interest and rapport

The strategies and examples outlined below cover several possible interest- and rapport-building opportunities that may be encountered in the introductory call, or in subsequent calls.

If rapport is not improved, the counselor should continue to be positive. Some students will not immediately respond to the rapport-building initiatives, but will still be willing to participate in the intervention. The counselors' efforts – and patience – may pay dividends later in the intervention, once the participant has had more time to recognize the counselor's authentic and sincere interest in him/her as an individual.

Building interest and rapport

Strategies:	***Examples:***
During the introductory call to a student, the goal is to engage students quickly and without pressuring them immediately to consider their own smoking behavior. If a student is hesitant, try talking about a topic that will help bring him/her along.	*"Have you seen the TV ads about tobacco, like the one with the little kid named Jake who's dad died of lung cancer, or the one with the woman who smokes through a hole in her neck? Those ads were written by high school students. What do you think about those ads . . .?"*<br>*"Your opinions about those ads, and about everything we talk about, are very important to us . . ."*<br>or<br>*"So, you're a senior, planning to graduate in a few more months. What are your plans for after?"*<br>or<br>*"You're in Running Start? A lot of students I talk with are doing that, too. How do you like it?"*<br>or<br>*"Senior year – long haul's almost over. What kind of classes are you taking this semester?"*

Strategies:	***Examples:***
If a student seems interested about the fact he/she has been called or selected by the study, try to capitalize on this interest; be direct.	For Smokers:<br>*"Do you remember the survey about smoking you completed at the end of your junior year? We really appreciated your participation! As a follow-up activity to that survey, I've been asked to call you, to learn more about you and about your frank and personal opinions about smoking/not smoking, how smoking does or doesn't fit into your life, and about your choices regarding tobacco."*<br><br>For nonsmokers:<br>*"Do you remember the survey . . . ? I'm calling because you indicated on that survey that you'd be interested in learning some tips for helping your friends who smoke and want to stop. Can you tell me more about that?"*
If participant is a <u>**former smoker**</u>, talk about that accomplishment.	*"You were a smoker but quit. That's an accomplishment that many of your peers and even adults can't claim. What worked for you? What helped you make the decision to quit?"*
If a student seems reluctant to talk, start with topics that might seem more relevant, less intrusive, to him/her. Show interest in the participant by asking about broader interests, values and goals. Doing so demonstrates interest in the participant as a person.	*"First, I'd like to find out more about you, your interests, your daily routine. I know that last year you were a junior at ____ High School. Do you still go to school there? What else keeps you busy? . . . What about a job – are you working? . . . Are you involved in any sports? . . ."*<br><br>*"First, it would really help me to know more about you – it's important to our research to know more about people as individuals."*
During subsequent calls, use a motivational opening strategy to re-establish and build on rapport begun in the last call. Show interest in the TP by referring to something discussed at last contact, either something personal, or something said with regard to the participant's smoking situation.	*"What's been happening with you since the last time we talked? You were [personal reference here, i.e., in drama rehearsals]. How'd that go?"*<br><br>*"Last time we talked about general lifestyle stuff, daily stresses, and how smoking fit in. Has anything changed for you?"*
During conversations about smoking or stopping, express acceptance, regardless of whether the participant is ready to think about stopping, or even talk about his/her smoking.	*"It sounds like you're not prepared to (talk about smoking/non-smoking) right now . . ."*<br>or<br>*"It sounds like you don't feel talking to me now about this would be helpful to you."*

Strategies:	***Examples:***
Keep building that trusting relationship, even when concluding calls. When concluding calls with precontemplative & contemplative smokers, leave the door open for further contact without pressuring them to stop. Participants' positive experience with the counselor in the current call will make him/her more receptive to receiving a future call	*"I've enjoyed talking with you today. I'd like to mail you some information about some of the stuff we talked about today, and then give you a call back in a couple weeks and see what you're thinking. Should I mail it to your home address, or another? (It will be in a plain envelope.) When's the best time for me to reach you again?"*

5.4 Collaborative Agenda Setting

One method to build strong counselor-participant relationships is to use collaborative agenda setting. Collaborative agenda setting involves the counselor and participant working together to set the agenda for the call. It lets the participant know up front what he/she and the counselor will be talking about during the call and helps move the call forward.

Establishing the call's agenda with the participant sets up realistic expectations (and removes the fear of not knowing what this call will be all about), provides structure to the call, and shows respect and distributes responsibility for the discussion equally to the counselor and participant (providing accountability to both). Collaborative agenda setting can also be used to set approximate times for discussions during the call, and to bring the conversation back to the agenda if it strays – after all, the agenda is one that the participant agreed to.

An easy way to do this is to propose a tentative agenda to the participant, and ask him/her if that sounds okay, and is there anything else he/she'd like to add. Example:

> *"Today, I'd like to get your views on smoking. So, I thought we'd talk about your opinions of smoking and not smoking, why you think some people smoke and others don't, and anything else you'd like to talk about. Does that sound okay to you?"*

5.5 Motivation for Future Calls

The strategies and examples outlined below cover several possible interest- and rapport-building opportunities that may be encountered in the introductory call, or in subsequent calls.

The strongest motivation for accepting a "next" call is the great call that is just being completed. Participants are more likely to want to continue participation and accept additional calls if their current (and all previous) calls leave them feeling that we are genuinely interested in them as individuals; that we respect them, particularly their right to make decisions about smoking for themselves; that we are appreciative of their help; and that they have something important to contribute to our research. Counselors in the pilot study reported "it is such a hook for participants to know they're helping with research. It breaks down their defenses and makes their attitude about accepting a second call almost universally fine."

Leaving the participant with motivation for the next follow-up call can also be helpful. Examples:

"I'd like to call back to:

. . . provide you with some feedback on what we talked about today."

. . . just to check in and see where you are with all this smoking stuff."

. . . just to check in (no reason)."

. . . get your feedback on some materials I'd like to send you."

. . . learn more from you – including lots of different people and getting lots of their personal perspectives really helps with our research. We really appreciate your time and help."

"I'd like to have a chance to do some research for you, to better answer the questions you had about [topic]. How about if I do that, and then call back to let you know what I find out?"

"You've talked a lot today about how smoking helps you feel less stressed, but that there are lots of things about it you don't like so well, like how much the smell gets in your hair and clothes so that other people can smell it on you, and you don't like the price increase. Maybe you can think about what other things you currently do, or could do, to help you with your stress, and I'll check and see what other people I talk to are doing and then I could call you back and we can compare notes. How does that sound to you?"

"I want to be a resource for you in any way that seems helpful to you. Other students have used these calls to . . . .

. . . decide what they might do in a situation where they are tempted to smoke but really don't want to."

. . . learn how to feel better and less stressed out without smoking.

"We know that often people's ideas and thoughts about smoking can shift over time. I'd like to check on new thoughts or decisions that you may have in the next few weeks/months."

6. INITIAL CALL

Initial calls to trial participants accomplish several important goals. First, this call is the counselor's first verbal contact with the trial participant (TP), so making a positive first impression is essential. Also, it is in the initial call that the counselor conducts informed consent to ensure that the TP understands his/her choice to participate in the research study and his/her rights as a research participant. Finally, it is in the initial call that the counselor elicits the TP's frank and personal opinions about smoking and nonsmoking, and assesses his/her current smoking status, lifetime smoking history, and readiness to quit. The information gained enables the counselor to seamlessly segue during the initial call into the appropriate stage-matched smoking or nonsmoking intervention.

Call Elements

- Introduction
- Conduct Informed consent
- Confirm Privacy
- Seek Personal Opinions and Insights
- Build Interest / Rapport / Motivate Future Calls
- Assess Current Smoking Status and Readiness to Quit

6.1 Introductions

As part of the initial call, counselors will introduce themselves as calling from the HS Study and confirm the identity of the TP. Following introductions, it's essential to start building rapport – engage the TP in some brief conversational exchange so that he/she will be ready to receive the important informed consent information that follows.

"Hi. May I speak with [participant's full name]?

"Hi, [participant's name], this is [counselor's full name] with the Fred Hutchinson Cancer Research Center's Study of High School Smoking.

At this point, take a moment to chat with the student to begin building rapport and help engage his/her interest. Examples of ways to do this (pick one or more, depending on the needs and interests of the TP, that is natural for you):

(weather) *"Wow – I'm glad I caught you on the phone since it's such a gorgeous day out. I hope you've had a chance to get out and enjoy some of that sunshine. Isn't it great?" or "It's just pouring buckets here; how is it out your way?"*

(student's busy schedule) *"Wow – I finally caught you at home. Your schedule must be busy! You must have lots of stuff going on. Are you in sports or a band or some other activity?"*

(the Hutch) *"Before I say anything more, I should ask – are you familiar with the Fred Hutch?* (If yes, *"Then you know we're a major cancer research center and our motto is 'Advancing Knowledge, Saving Lives' . . ."* / If no, *"Well, just briefly then, we're a major cancer research . . . ."*)

(last Spring's data collection) *"First, I want to thank you for completing the survey that we did among high school juniors at your school last spring. Do you remember that survey with the cotton dental roll?"*

After engaging the student, proceed with explaining the purpose of your call and conduct the informed consent.

Note: Don't be afraid to follow the lead of the student. For some students, "less is more." They want to get to the point of the call quickly and won't be open to rapport-building chat until they have heard more from you about why you are calling. You will be able to ascertain this from the student's responses to you on the phone. If you're talking to a "get-to-the point" kind of student, go ahead and proceed with the call without engaging in initial small talk.

6.2 Conduct Informed Consent

Conduct the review of informed consent, and obtain participant's consent to participate, as documented (below) and approved by FHCRC IRB.

Note: Conduct the informed consent in a conversational, natural way. You don't want to sound like you're reading it or reciting it rote from memory. Your tone should be friendly and convey your interest in being supportive and helpful. The TP may interrupt you with questions; answer the questions and proceed with the consent, modifying it as needed if the questions/answers pertained to information upcoming in the consent. Reading the consent verbatim is not required, but **counselors are required to cover all [<u>underlined</u>] essential elements of the informed consent**.

"We recently sent you some info about the Matchbreaker Program. Did you get it? [Pause. If student answers "no," respond *"That's okay; I can tell you a little about it."* If student answers "yes," respond *"Oh, good. I'm calling to tell you a little more about it."*

*"We're calling people – both smokers and non-smokers – who participated last year in our survey of high school juniors. Our goal is to talk to as many of you as possible, to learn from you as much as we can about what's going on with smoking these days. For example, we'd really like to know more about why some people decided against smoking, why some people want to keep smoking and others don't, why some can smoke just once in awhile, and how best to help the people who smoke and want to stop. Matchbreaker is **not** about pressuring people to stop. But, if someone we talk to decides to stop smoking, we will offer help.*

*"So, I'm calling <u>to invite **you** to participate in this research study activity</u>. We'd especially like to include **your** valuable thoughts and opinions in our research. And participation is easy – just talking on the phone.*

> *"Of course, Matchbreaker participation is voluntary! You may ask questions whenever you want, spend as much time talking as you want, or decline at any time.*
>
> *"I also want you to know what we discuss during any Matchbreaker call is confidential. I cannot and will not share our conversations with* ***anyone*** *outside our research office. An exception to this would be if I happened to learn through our conversation about a clear, immediate danger to you or others. Also, my supervisor may monitor or record a call to make sure that I'm doing my job well.*
>
> *"Does all that sound okay to you? Do you have any questions?"* [Answer all questions. If student objects to being recorded, note that and do not record the call. If student declines to participate, record that information and conclude the call by thanking the student for his/her time and consideration. When all questions have been answered to student's satisfaction, proceed.]
>
> *"Okay! How about if we get started?"*

[If student assents, record date and time on the Intro Call form and proceed with telephone counseling call protocol.]

Remember, consent is a process, not just a form or script. Informed consent requires two-way communication; it's more than the words on the page. Ask yourself, *"Have we communicated? Do I know what the participant wants to do?"*
If the student agrees to participate, but you think his/her consent is a little "mushy," empower the student by reminding him/her that he/she can stop at any time. For example,

If you can't tell what the student wants to do, rely on reflective listening. Reflect back what the student has said, and then ask him/her, *"Does that mean you want to participate? I want to be sure that I understand what you'd like to do."*

If the student declines to participate, record that information on the Intro Call form. Express appreciation to the student for reviewing our materials and taking the time to consider participation. Offer to leave our toll-free telephone number, in case he/she changes his/her mind about participating (which may happen after he/she talks to other classmates who are participating). End the conversation on a positive note by thanking the student for his/her time.

If the student agrees to participate, but now is not a good time for a call (e.g., it's dinnertime, or he/she has to leave for work, etc.), make arrangements to call the student back at a time that is convenient for him/her. Record the consent information and next call appointment on the Intro Call form, and record the call as "partially complete."

6.3 Confirm that Participant Has Privacy for the Call

To help protect participant confidentiality, confirm that the TP has sufficient privacy for the counseling session. For example:

> *"First, I want to be sure that you feel like you're in a comfortable, fairly private space for talking to me on the phone. Do you feel you have enough privacy?"*

Let the participant decide what is enough privacy. If needed, suggest measures the participant can take to avoid having others eavesdrop on the conversation (e.g., move to another room, close the door). If necessary, arrange to call back at another time/place.

6.4 Seek Personal Opinions and Insights

A major goal of the HS Study is to seek participants' frank and personal opinions and insights about smoking and non-smoking, as well as details of their unique situations with regard to smoking, in order to better understand the motivations for/characteristics of high school smoking and non-smoking. Often counselors will unconsciously begin speaking generally about tobacco and asking the participant's opinions early in the intervention as part of rapport-building. In this way, the goal of seeking participants' frank opinions and insights assists with building counselor-participant rapport and generating participant interest in the research. Examples of how to start this discussion:

> *"An important goal of this study is to hear from individual students about their thoughts about smoking and nonsmoking. What are your opinions, in general, about smoking?"* Or:
>
> *"One way we learn about what's going on with smoking is by doing pencil and paper surveys with high school students, like you. And often, the students write in that the surveys don't quite fit them, so we've decided to toss the pencil and paper for now, and just talk to students one-on-one to find out what they think about smoking, how it fits in their lives, or doesn't. So, I'd love to hear from you, about your thoughts and opinions of smoking."*

If the student has trouble with such a broad question, trying narrowing it down: *"Well, for example, there's a lot of pressure on people now not to smoke in public, not to expose other people to their cigarette smoke. What do you think about that?"*

6.5 Build Interest and Rapport; Build Motivation for Future Calls

In <u>every</u> call to participants, establishing and maintaining participant interest and counselor/participant rapport with the goal to maintain contact and keep the participants involved with the intervention is paramount. Express acceptance; don't pressure. Provide encouragement as appropriate. Build motivation for accepting future calls. For detail and strategies, see Section 5.

6.6 Assess Current Smoking Status and Readiness to Stop

The participant's smoking status and stage of change may have changed in the three to six months since he/she completed the baseline survey at the end of his/her junior year of high school. Therefore, it is essential to find out about the TP's current smoking/non-smoking practices. Counselors need this information to determine where the individual fits in the telephone counseling sequence and to decide how best to tailor the intervention to the individual.

Through conversation with the participant, elicit the answers to the question(s) below. These questions are designed to assess current smoking status and readiness to stop. Record the answers as process data.

> *Tips:* Be conversational and friendly in the way you ask the questions; don't make it sound like you're administering a survey. Don't offer answer choices; probe as necessary to obtain the staging information. Keep in mind that asking strategic questions can be a positive exchange: the counselor is learning about the participant, demonstrating interest in him/her, creating counselor-participant rapport, and getting information valuable to the research in return. For most participants, counselors can be fairly direct in their questions about smoking behavior, but should always try to weave the questions into the course of their conversations with the participants, so that the questions are relevant. Be aware of how the participant views themselves (e.g., as a smoker or nonsmoker) when choosing when/how to weave these queries into the conversation.

Sometimes when a participant is finally reached on the telephone, he/she may not have a lot of time because of other activities going on in the home (e.g., it's dinnertime) or other scheduled commitments (e.g., has to get ready and leave soon for work). Nonetheless, it's really helpful to be able to get through the assessments portion of the call, after receiving consent to participate, rather than waiting to do it in a next call. Therefore, try to motivate the TP to stay on the telephone a few more minutes, if just to complete the assessments. Example:

> *"Gosh, you're so busy and it's taken me forever to catch you. I have just a* ***few*** *questions to ask that will take just a few minutes longer. If we get those answered, I'll be able to give you some feedback in our next call. Would that be okay?"*

6.6.1 Querying for current smoking status

Assessing for current smoking status requires two pieces of information: (1) does the participant currently smoke? and (2) When did he/she smoke her last cigarette? The second question provides counselors with a hint of how much the participant smokes, which is useful when tailoring the intervention. It also helps the counselor identify those participants who smoke only occasionally and do not see themselves as smokers. For example, such a participant may tell the counselor that no, he/she

doesn't currently smoke, but his/her last cigarette was last weekend or last month.

Work these questions into your conversation with the student. Example:

"I can tell you have pretty strong feelings about smoking. Tell me, do you currently smoke cigarettes? No? I hear that. So, like, have you ever tried it? When did you last smoke a cigarette?"

6.6.2 Querying for readiness to stop

Knowing where the participant is in terms of his/her readiness to stop smoking is key to tailoring the intervention to the individual. Previous research has clearly demonstrated that matching the intervention to the client's readiness to change increases the chance that he/she will abstain from smoking.

When assessing the smoker's readiness to stop, ask open-ended questions in the course of your conversation. Doing so may help the participant to be more receptive to talking about stopping. For example, counselors may choose to frame the staging questions as part of the job of the counselor:

"People differ a lot in how ready they are to stop smoking. It would be helpful to me to understand where you are in wanting to stop – do you have any thoughts about just walking away from it?"

For participants who smoke only occasionally, or who do not see themselves as smokers, asking them about their plans to quit will hold no relevance for them. More skillful probing and listening skills may be needed to elicit the staging information from the participant:

"Has it [participant's smoking] always been that way? . . . What about the future – what might be different for you then?" . . . "So you think you'll stop. Do you think that's something you want to do soon?"

6.6.3 Additional Queries

Counselors have the flexibility to ask questions in the course of their conversation about other smoking-related issues that will help them better tailor the intervention to the individual. For example, it can be helpful to know whether the participant views him-/herself as a smoker, and to know the participant's lifetime smoking history.

6.7 Process Data Items: Current Smoking and Readiness to Quit

Q1. Do you currently smoke cigarettes?

a. No ***[go to Q2a]***
b. Yes ***[go to Q2a]***

Q2a. When did you last smoke a cigarette?

a. Never ***[proceed with NS call]***
b. Earlier today
c. 1-7 days ago
d. 8-30 days ago
e. Between 1-3 months ago
f. Between 3-6 months ago
g. Between 6-12 months ago ***[proceed with NS call]***
h. More than 12 months ago ***[proceed with NS call]***

Q2b. Query, as needed, to code TP's smoking pattern or "type," if information not already gleaned from counseling conversation.

a. Never smoker [autoset if Q2a=Never]
b. Regular smoker
c. Occasional smoker
d. Recently quit smoker (quit in last 3 mos.)
e. Quitter who slipped/relapsed
f. Former smoker (stopped > 3 mos. ago)

[If Q2b = regular or occasional smoker, proceed with staging Q3-5, and then select call type according to stage. For all other responses, use section 6.8 as a guideline to decide on how to proceed with call type.]

Q3. Have you ever thought about stopping?

a. Yes ***[go to Q4]***
b. No ***[stage = precontemplation]***
c. Don't know ***[stage = precontemplation***

Q4. Are you thinking about stopping in the next 6 months?

a. Yes ***[go to Q5]***
b. No ***[stage = precontemplation]***
c. Don't know ***[stage = precontemplation***

Q5. Do you think you'll be ready to stop in the next 30 days?

a. Yes ***[stage = preparation]***
b. No ***[stage = contemplation]***
c. Don't know ***[stage = contemplation]***

6.8 Using process data to assign intervention components

Participants' smoking status or stage of change may change in the months between baseline assessment and the initiation of Matchbreaker calls. Counselors will use participant's current status to determine what type of intervention call to do, e.g., nonsmoker, vs. motivation enhancement vs. cessation support. Assigning the appropriate intervention component to a participant involves the counselor using the following guidelines and, in some instances, his/her best judgment.

Baseline status: Smoking
Initial Call status: Smoking, stage = precontemplation or contemplation
Intervention: Proceed with Motivation Enhancement, section 7.

Baseline status: Smoking
Initial Call status: Smoking, stage = preparation
Intervention: Proceed with Preparation/Cessation Support, section 8.

Baseline status: Smoking
Initial Call status: Not Smoking (quit since baseline), stage = action
Intervention: Proceed with Cessation Support, section 9, mapping the relapse sensitive call schedule onto the participant's cessation attempt.

Baseline status: Smoking
Initial Call status: Hasn't smoked in the last month or more
Intervention: Proceed with Cessation Support, section 9, mapping the relapse-sensitive call schedule onto the participant's cessation attempt.
Exception: If TP claims he/she is a never smoker (contradicting baseline data), provide nonsmoker intervention. *Judgment call*.

Baseline status: Nonsmoking (Formerly smoked)
Initial Call status: Has smoked in the past 3 months
Intervention: Proceed with Smoking Cessation intervention, selecting ME vs. CS content based on participant's readiness to stop.

Baseline status: Nonsmoking (Never smoked)
Initial Call status: Has smoked in the past 3 months
Intervention: *Judgment call.*

(1) If assessment reveals TP was just trying smoking (e.g., </= 2 cigarettes), hasn't smoked since, views self as nonsmoker: Proceed with nonsmoker intervention.

(2) If assessment reveals TP has smoked > 2 cigarettes (i.e., more than just "trying"), proceed with Smoking Cessation intervention, selecting ME vs. CS content based on participant's readiness to stop.

(cont'd)

Baseline status: Nonsmoking
Initial Call status: Nonsmoking
Intervention: Proceed with Nonsmoker intervention, section 10.

Once the appropriate intervention component has been identified, the counselor should seamlessly segue into that call component, as time allows.

7. MOTIVATION ENHANCEMENT (ME) CALLS

Participants who smoke but are not ready to stop will be scheduled to receive up to three counselor-initiated motivation-enhancement (ME) calls, as shown in Figure 1 (section 2). ME calls are designed to induce motivational stage progression and ultimately trigger a decision and commitment to stop smoking. [Counselors have the discretion of making a fourth motivational enhancement call in the rare instances of having a participant very close to making a commitment to stop after the prescribed three calls.]

For precontemplative and contemplative participants, counselors will practice motivational interviewing strategies, focusing on building motivation for change. Strategies in these stages emphasize building the participant’s perception that stopping smoking is important and building the participant’s confidence in his/her own ability to make such a change. Counselors will work to facilitate discrepancy and elicit change talk. When, in the course of the M-E calls, the counselor recognizes the participant is ready to change, the counselor’s focus shifts from building motivation to strengthening commitment to change and preparing the student to stop smoking. Counseling strategies would focus on summarizing the participant’s current situation and feelings about change, helping the participant decide what changes he/she wants to make, and helping the participant develop an action plan for stopping smoking. Counselors will continue to adhere to MI principles in order to support the counselor-student relationship and reinforce students’ ownership of their decision to quit.

Reminder: There is no preset timeline for getting the participant to progress to the next stage. While the counselor will strategically lead discussions with the goal of moving the participant towards making a decision to change, the pace is always set by the participant. The decision to change is always made by the participant. The counselor can’t *make* anyone stop smoking.

Those participants who are not prepared to try to stop smoking following their final motivation-enhancement call will be encouraged to re-contact their counselor if they change their minds during the current [school] year, and to consult the Web site for additional information. Access to Web site features will remain available to all students, regardless of their decision to stop smoking or participate in the study.

M-E Call Elements

- Introduction
- Build Interest & Rapport / Motivate Future Calls
- Revisit Consent/Privacy
- Seek Personal Opinions and Insights
- Assess Unique Situation
- Build importance *(Precontemplation, Contemplation)*
- Build confidence *(Precontemplation, Contemplation)*
- Recognize Readiness to Change *(Preparation)*
- Strengthen Commitment to Change *(Preparation)*
- Negotiate an Action Plan *(Preparation)*
- Wrap-up the call

7.1 Introduction

Counselors will identify themselves (by full name and as calling from FHCRC) on all ME calls. It's helpful to greet the participant by name and, to get the ball rolling and re-establish rapport, remind him/her of something you learned from the last call. If the counselor promised some feedback or answer to a question, he/she should offer it early in the call. Examples:

> *"Hi, Nicole. This is ______ from the Matchbreaker Study at the Fred Hutch. How've you been? [response] I think last time we talked you were in rehearsals for the Spring musical. How was that? [response] It must feel great to have worked so hard and then to have had such great audience response – that's terrific! Well, today I thought we'd . . ."*

> *"Hey, John. This is ______ from the Matchbreaker Study at the Fred Hutch. How've you been? [response] Last time I talked to you, I promised to look up the answer to your question about light cigarettes. Do you remember that? [response] Well, what I found out for you was really interesting . . ."*

7.2 Build Interest and Rapport; Encourage Future Calls

In <u>every</u> call to participants, establishing and maintaining participant interest and counselor/participant rapport with the goal to maintain contact and keep the participants involved with the intervention is paramount. Express acceptance; don't pressure. Provide encouragement as appropriate. Build motivation for accepting subsequent calls. (See section 5.)

7.3 Revisit Consent / Privacy

Each time a participant is called, counselors must remind him/her that the call may be monitored or recorded. Example:

> *"My supervisor may monitor or record this call to make sure that I'm doing my job well. Is that okay with you?"*

Do not record a call if the participant indicates he/she does not wish to be recorded, and record in the TC Notes that participant asked not to be recorded.

Also, with each call, take a moment to be sure that this is a good time for the participant to talk and that he/she has enough privacy to participate in the call. Example:

> *"Is this a good time and place for you to talk to me on the phone?"*

Let the participant decide what is enough privacy. If this is not a good time/place for the call, make suggestions for enhancing privacy, or make an appointment to call the participant at another time/place or on another phone line.

7.4 Seek Personal Opinions and Insights

Continue to take advantage of natural opportunities that arise during conversations to learn participants' frank and personal opinions and insights about smoking and non-smoking, as well as details of their unique situations with regard to smoking. Doing so helps maintain rapport and provides counselors with tailoring the intervention to the individual. See sections 5 and 6.4.

7.5 Assess the Participant's Unique Smoking Situation

As we learn more about the participant, we'll want to also know more about the role smoking plays in his/her life. Assessing the participant's unique situation allows counselors to identify how to best proceed with the smoker intervention telephone calls; it provides useful information that the counselor can use when tailoring the intervention to the individual participant. The assessment also helps the counselor demonstrate that he/she wants to know what smoking means to the participant, it provides a common understanding shared by the counselor and the participant, both of which help build rapport, and it gets the participant to start thinking reflectively about his/her smoking.

In addition to readiness to stop smoking (already assessed), important aspects of the participant's unique situation are the importance he/she places on stopping, and his/her confidence in stopping. The participant's readiness to change may be influenced by his/her perceptions of the importance of stopping and confidence in his/her ability to do so. For example, a participant may be convinced of the personal value of stopping (*importance*), but not feel confident about mastering the skills necessary to stop (*confidence)*. Alternately, other participants may have mixed feelings about the importance of stopping (*importance*), but say that they could stop fairly easily (*confidence)* if they really wanted to. Learning the participant's perception of importance, confidence, and readiness helps the counselor to tailor the counseling session and match the call content to the individual. To further understand the participant's smoking, the intervention will also assess frequency of smoking, and level of nicotine dependence (this information may be drawn out through informal conversation during the call or via formal assessment).

Assess the smoker's unique situation

Strategies:	Examples:
Begin by asking the participant to describe how smoking fits into his/her lifestyle, or to describe a typical day.<br><br>If participant has indicated he/she has already decided to stop, modify statements accordingly, e.g., "*Before we talk about your decision to stop, I'd like to understand better how smoking fits into your life. Can you describe a typical situation when you smoke?"*)	*"I'd like to understand better how smoking fits in to your life. Can you describe a typical situation when you smoke?"*<br>Or<br>*"Let's spend the next few minutes going through a typical day for you. For example, describe today . . . what did you do today, how did you feel?"* [As participant brings up life situations, demonstrate interest.] *"So, you have to be at school by 7:30 a.m. for marching band practice. That's early! What instrument do you play?"* [As the opportunity arises, bring up smoking.] *"So, you're a trumpet player. Does your smoking ever impact your music, like affect how well you blow your horn?"* or be more general: *"So, how did smoking fit in to your day today?"*
For **infrequent smokers**, consider asking about specific situations when they may smoke, e.g., weekends or parties with friends.	*"Sounds like you only smoke once in a while, like when you're at a party. Tell me more about situations like that."*
Asking about a typical day provides a natural opportunity to follow-up with questions about level of smoking:<br>Process data item	*"Sounds like from your description that you're smoking (every day/weekly/monthly). Is that right? . . . About how many cigarettes would you say you smoke each (day/week/month)?"*
(If participant is in preparation stage) Explore the participant's motivations for stopping smoking.	*"Now, you mentioned that you're thinking about quitting smoking soon, like in the next 30 days or so. What has led you to that decision?"*
Assess importance of stopping smoking. Record the answer<br>Process data item<br><br>Note: If value placed on importance is low, work on building importance for stopping.	*"Right now, today, how important is it to you personally to stop smoking? If "0" was not important and "10" was very important, what number would you give yourself?"*<br><br>0 - 1 - 2 - 3 - 4 - 5 - 6 - 7 - 8 - 9 - 10<br><br>or, if participant is talking about stopping (i.e., in preparation),<br>*"It sounds like stopping smoking might be pretty important to you. Can you give me a feel about how important? Right now, today, how important is it to you personally to stop? If "0" was not important and "10" was very important, what number would you give yourself?"*

Strategies:	Examples:
Assess the participant's confidence in his/her ability to stop smoking. Record the answer. Process data item<br><br>Note: If confidence is low, work on building confidence.	*"If you decided right now to stop smoking, how confident are you that you would succeed? If "0" is not at all confident and "10" is very confident, what number would you give yourself?"*<br><br>0 - 1 - 2 - 3 - 4 - 5 - 6 - 7 - 8 - 9 - 10
Assess the participant's current dependence on nicotine. Record the answer.<br><br>Process data item	*"On a typical day, how soon after waking up do you have the urge to smoke your first cigarette?"*<br>a. As soon as I wake up<br>b. Within about ½ hour of waking<br>c. An hour or two after I wake up<br>d. Over 2 hours after waking

7.6 Build Importance

Once a participant places a high level of importance on stopping smoking, he/she will be more likely to be motivated to try to stop. Thus, building importance and motivation to stop are key goals when working with precontemplative and contemplative smokers. Strategies for building importance are provided in the next table. (Because building motivation to stop among participants who smoke only infrequently is especially challenging, *additional* strategies for participants who smoke only occasionally are outlined separately, in section 7.6.1.)

Build importance

Strategies:	Examples:
Create discrepancy (this strategy creates tension and the desire to resolve the tension). This can be done by having the participant talk about the good things and "less good" things about smoking. This strategy places no pressure on the participant with regard to stopping.	*"Let's talk about the good things and the not so good things about smoking. First, what would you say are the good things about smoking."*<br><br>*"Now, for you, what are the less good things?"*
Another strategy for creating discrepancy is to talk about the pros and cons of smoking and of stopping. "Pros & cons" is a more direct approach then talking about "good things/less good things" so should not be introduced with participants who don't want to talk about their feelings about stopping. Also, it is helpful to start with the pros of smoking if the smoker is in pre-contemplation or contemplation, or if he/she seems unclear about the issues, or seems to feel ashamed about smoking. Starting with the positive will help with rapport-building, and place the behavior in a normal context.	*"Sometimes it can help to talk about the pros and cons of smoking, and of stopping. Would you like to spend a few minutes doing this?"*<br>Or<br>*"When people are unsure about a change, there are pros and cons of staying the same, and pros and cons for changing. Have you ever thought about stopping smoking like this? Would you like to spend a few minutes talking about it?"*

Strategies:	Examples:
Explore participant's concerns about his/her smoking. (*Note*: Concerns are different from pros and cons. Concern has a stronger emotional connotation, which is avoided when asking about good things/less good things or pros and cons, a better strategy with participants not ready to change. Asking about concerns is useful if the participant is more ready to change. When asking about concerns, provide structure: listen carefully, and then summarize at the end. Let the participant explain how he/she really feels.)	*"What concerns do you have about your smoking?"*<br><br>Follow-up (ask about change in a neutral way):<br>*"Where does this leave you now?"*
If importance/motivation is low, explore ways to increase it. If the participant responds with impossible goal for increasing motivation, consider the strategy of using a reflective statement followed by agreement with a twist.	*"What would have to happen for it to become much more important for you to change?"* or *"What would have to happen for your importance score to move up from (x) to (y)?"*<br><br>TP: "If they found a spot on my lung or something."<br>*"It sounds like things would have to be pretty bad then, for you to consider making a change. It's too bad they don't have a test that would let people know if smoking was going to make them sick in the future."*
Another strategy to try in such a situation is amplified reflection. This strategy can get participant's to argue your point of view, reversing his/her position (i.e., saying they will quit).	*"So, you'll never quit, then."*
Explore motivating factors that the participant brings up, or if necessary, introduce possible motivators for discussion. Examples include social unacceptability of smoking, loss of personal control to cigarettes, financial costs of smoking, the impact of second hand smoke on others, hassles from family about smoking, trouble at school over smoking; physical consequences; the smell of cigarette smoke on hands, clothes, hair and the participant's feelings about others smelling smoke on him/her.	*"Have you ever felt uncomfortable smoking around friends, family or a girlfriend/boyfriend?"*<br><br>*"Do you ever feel like cigarettes control your life too much?"*<br><br>*"Cigarettes just took a huge jump in price. How has that affected you financially?"*<br><br>*"You say you used to run track but not anymore. Do you think your smoking had anything to do with that?"*<br><br>*"I think a neat thing about competitive swimming is that you can compete against your self – your own time – as well as against other swimmers. Do you find yourself doing that? I'm wondering, have you noticed if smoking has affected your times?*

Strategies:	Examples:
Another strategy for building motivation that uses motivating factors voiced by the participant is strategic reflection of feelings.<br><br>If you get a feeling for the participant's emotions that could put behavior change on your side, use it!	*"Being a bad example to you little sister really makes you feel guilty. That must be difficult for you."*<br><br>*"It's awful to think about your grandmother smelling the cigarette smoke on you. You must really care about her."*<br><br>*Other lines:*<br>*". . . and that makes you feel bad."*<br>*". . . I hear a lot of pain in your voice when you talk about that."*
When working with a precontemplative participant, try asking about hypothetical reasons he/she might want to stop. Doing so provides an opportunity to take such a response and use it to discuss the issues further.	*"Even though you're not interested in stopping smoking now, if there was a reason why you might stop in the future, just hypothetically, what would it be?"*<br><br>Some will answer, "If they found a spot on my lung or something." The counselor could reflect and add a whimsical aside to see where that might lead:<br>*"It sounds like things would have to be pretty bad then, for you to consider making a change. It's too bad they don't have a test that would let people know if smoking was going to make them sick in the future."*
If participant's perceived importance and interest in giving up smoking are markedly low, do little more; let the participant think about it.	*"Perhaps now is not the right time to talk to you about the possibility of stopping smoking. I'll touch base with you in a few weeks – what's a good time for me to reach you?"*<br><br>Or, *"You say you are unsure what to do. I don't want to push you into a decision; it's really up to you. Take your time to think about it. You will be the best judge of when it is the right time for you to stop. I'm here to support you, if and when you choose to stop."*
Use summarizing frequently and throughout the calls, and use "what else" to invite the participant to continue.	*"So, you say you like smoking with your friends, and you're really bothered by the smell it leaves on your hands and clothes, and don't want your teachers to smell it. What else?"*

Strategies:	Examples:
If rapport with the participant is not fragile, consider providing information / normative feedback.	*"Other participants have felt like you do. Would you like to hear what some of them did in similar situations?"*<br><br>*"Other students I talk to . . . ."*<br><br>When offering advice or information, always elicit feedback:<br>*". . .What do you make of that?"*<br>*". . . What does that mean to you?"*
Looking to the future (for those who appear interested in stopping).	*"How do you think things would be different for you in the future if you stopped smoking?"*
Use self-disclosure, i.e., use your reaction to what the participant is saying to get his/her attention.	*"What I hear . . ."*<br>*"Yeah, I agree that smoking's bad for your health, but I'm not telling you anything you don't already know."*

7.6.1 Additional Strategies for Infrequent Smoking

Building importance and motivation to stop smoking are no less important when working with a participant who only smokes occasionally. However, working with someone who smokes only infrequently can be particularly challenging because many of these people do not think of themselves as smokers and do not view their occasional smoking as a problem behavior that needs to be changed. Thus, stopping smoking is not a relevant goal. [The actual situation is more sobering: Data from the Hutchison Smoking Prevention Project (HSPP) and other national studies have demonstrated that infrequent smoking by teens leads to regular smoking. For example, 40% of the HSPP 12th grade infrequent smokers were smoking daily 2 years later. In some other national studies, the number is as high as 50%.] Therefore, when working with someone who smokes only occasionally, the counselor has the additional challenge of making stopping smoking a goal that is relevant and important.

A first step may be rephrasing the goal to make it more relevant: talk about *"giving up cigarettes for good," "just walking away from it,"* or *"not continuing."* Avoid using the word "quit." Many of the strategies in the preceding table are also very appropriate for building importance with participants who smoke infrequently: Using proximal risks and issues important to the participant to build discrepancy (e.g., their dislike of the way smoking makes them smell); using agreement with a twist and amplified reflection to increase motivation; exploring motivational factors raised by the participants; using strategic reflection of feelings; summarizing; and using self-disclosure.

Providing feedback also can be an important strategy in creating discrepancy in infrequent smokers. Additional examples are shown in the table below.

Additional strategies for building importance

Strategies:	Examples:
Emphasize how little the infrequent smoker is smoking; use that information to get the participant thinking critically about his/her own smoking. This is a "good for you!" strategy; it emphasizes the positive, not the negative. Teens will usually make negative statements about smoking – reasons they don't want to smoke more – and, using values clarification, you can use that information to build ambivalence.	*"When so many others smoke so much more, what keeps you smoking at your level?"*<br>*"So, by not smoking more you're doing something good for yourself. How could you do better?"*<br>*"Wow, you're only smoking one cigarette a day while all your friends smoke lots more. Why are you different from your friends?"*
Participants who smoke only a little may be surprised to learn that smoking even a single cigarette can cause negative physiological effects on the body. Offer this feedback to the participant.	*"Doctors have found that smoking even occasionally can have negative physical effects on the smoker. If you're interested, we can talk about this area of research and about the harm that even a single cigarette can do to the body."*<br>or (subsequent call)<br>*"Last time we talked about how even smoking one cigarette can hurt the body. Have you had any more thoughts about that?"*
When talking to participants still "experimenting" with smoking (i.e., </= monthly smoking, <100 lifetime cigarettes), try using the smoking literature to help shift the participant towards thinking of him-/herself as a smoker. For example, DiFranza & colleagues (2000) have identified signs of nicotine addiction in teens. Use these to help participants start thinking about their smoking as a problem. Be selective in what you ask from the examples list. Don't be dishonest, but use the literature to steer participants in the direction they need to go in order to think about stopping. (*Note:* This strategy may not have relevance for "triers," who've only smoked 1-2 cigarettes.)	*"Dependence on cigarettes can develop slowly and in a way that isn't that noticeable. Would it be okay if I asked you some questions that might help us see whether you are being affected like that?" . . .*<br>*. . . have you ever tried unsuccessfully to quit?"*<br>*. . . have you ever felt like you were addicted to tobacco?"*<br>*. . . have you ever felt like you really needed a cigarette?"*<br>*. . . when you haven't smoked for awhile, did you find it hard to concentrate because you couldn't smoke?"*<br>*. . . when you haven't smoked for awhile, did you feel more [irritable/nervous/restless/anxious/sad or blue] because you couldn't smoke?"*<br>If yes, *"In other studies where teens have reported the same thing, this characteristic has been identified as an early sign of addiction. It's interesting that you report the same characteristic. What do you make of that?"*
In subsequent calls, try asking about the same symptoms; if the TP is reporting more or different symptoms than previously, point this out.	*"Gosh. Last time we talked about this, you only told me about 2 symptoms. But today you say you've experienced 4. What's been different for you lately? What do you make of this change?"*

Strategies:	Examples:
If the TP seems confident he/she won't smoke in the future, offer normative feedback.	*"I can understand that you feel that way. In fact 95% of high school students who smoke have reported in surveys that they won't be smoking in 5 years. You're right on par with your peers. Students were resurveyed 8 years later about their smoking. Would you be interested in those results?"*<br>*If yes, "It turns out that out of every 10 of those high school smokers, only 2-3 were not smoking 8 years later. Almost all of them continued to smoke."* Wait for reaction. If none: *"Any thoughts?"*
Try exploring and reflecting values with the occasional smoker. This technique helps individualize the discussion to the participant, his/her values, his/her culture. Don't forget to key in on answers given to other questions; they can help you out here. Reflecting values also helps build rapport & a trusting relationship; the student knows he/she was heard.	*"Sounds like it's really important to you not to smoke around your younger brother. Tell me more about that."*<br><br>*"So, I hear you saying that, although you smoke sometimes, smoking is not really what you're about. It's not part of how you see yourself. Do I have that right?"*
Attempt to inject a little reality through self-discovery, around the occasional smoker's complacency about smoking only a few cigarettes and confidence that he/she can continue to do so in the future.	*"So you're saying that three cigarettes a day is okay for you and that you can continue that until that time in the future when you want to stop because you'll have kids. Tell me, how many adults do you know who only smoke a few cigarettes?"*<br>*"You do have a long-term goal to stop smoking. So, do you think it will be easier or harder to quit in the future?"*
Encouraging the occasional smoker to envision what his/her smoking may be like in the future can be helpful.	*"How do you see smoking fitting into your future? Will it increase or decrease?"*<br>*"Next year you'll be (in college, working, in the Marines, etc.). How will that change in your environment effect your smoking?"*<br>*"So, you only smoke when you're drinking, like at parties. Next year, you'll be at WSU. Do you think you'll be partying more in college? What will that mean to your smoking?"*
If counselor waited several months between calls, try exploring ways smoking behavior may have changed in the interim.<br>*Tip:* Be objective & dispassionate; let the data be confrontational.	*"So, you're smoking about 8 or 10 cigarettes every day now? When we talked last time, you were only smoking about 5 a day. So your rate of smoking has doubled. What do make of that?"*

7.7 Build Confidence

Raising an individual's motivation level may not be sufficient to make him/her think he/she is ready to prepare to attempt to stop smoking. A participant who places a high level of importance on stopping may nonetheless feel unable to do so. Low self-efficacy can sabotage quit attempts or even prevent the person from trying to stop. A lack of self-confidence in one's ability to maintain sustained abstinence has been identified as highly predictive of relapse [Baer, Holt, Lichtenstein, 1986; Condiottie & Lichtenstein, 1981].

Build confidence for stopping among regular smokers

Strategies:	***Examples:***
Explore/identify reasons for low self-efficacy. (This is easiest to do if the participant used a numerical scale for assessing confidence.) Counselors might ask, "Why so high?" or "How can you go higher?" See examples.	*"You said you were fairly confident you could stop – why do you say that? Why did you give yourself a 4 and not a 1?"*<br><br>*"You gave yourself a score of 4 when rating your confidence to stop. What do you think would help you become more confident, so that your score goes up to 5 or 6"?*<br>Or<br>*"What would help you to be more sure that you could stop if you tried?"*<br><br>*"What stops you moving up from 4 to 5 or 6?"*
Identify and challenge self-defeating thoughts about stopping smoking. These may arise from never having tried to stop, or from having stopped and relapsed several times. In this case, draw attention to successes of past efforts.	*"You mentioned that once before you stopped smoking for __ days/weeks. That's great. What helped you go so long that time?"*<br><br>*"It's great that you were able to stop before! Most smokers quit 3-4 times before they stop for good. Each time you stop, you learn a little more about what works and what doesn't. Let's talk about the last time you stopped smoking. Was there any part of that you found easy or encouraging?"*
Consider other ways to build participant's self-confidence; use examples from earlier in this or other call conversations to reflect/boost his/her strengths.	*"I know from talking to you that one of your strengths is your outgoing personality. What would you say your other strengths are?"*<br>*(Examples: Responsible, loyal, supportive, good self-control, caring, protective of self & others, trustworthy, assertive, friendly, determined, persistent, smart, athletic, creative, strong-willed, independent, etc.)*<br>*"In what ways could you use those strengths to help you next time you decide you want to stop smoking?"*
Draw attention to other obstacles the participant has overcome.	*"Has there been anything else in your life that was so hard you thought you couldn't do it, but you did? Tell me about that . . .."*

Strategies:	***Examples:***
Explore / challenge barriers to stopping smoking.<br><br>(Common teen barriers to stopping: triggers to smoke in social situations (e.g., at parties, when drinking), friends' reactions, fear of weight gain, fear of failure.)	*"What kinds of things would you be concerned about if (when) you stopped smoking? Have these ever happened to you before?"*<br><br>*"It sounds like you're concerned that if you stop, you'll gain weight and maybe be less popular. This is a concern I hear from other participants. What do you think you can do to avoid weight gain if you decide to stop?* (discuss*) Would you like to hear what other young women concerned about weight gain have found helpful?"*
Shift focus from confidence to motivation. One way to do this is offer the participant a scenario where he/she could easily see him-/herself being motivated not to smoke. This example helps the TP see how a strongly felt reason to stop (in this example, hypothetical $$) can overcome a lack of confidence.	*"Imagine that someone offered you $100 for every cigarette you did not smoke. Do you think you would take the $100 or the cigarette? . . . Why?"*
Brainstorm solutions; always encourage the participant to come up with a list of options and then discuss what might work best for him/her. Providing choices gives the participant control over his/her decision to stop smoking.	*"There is usually more than one way to solve this problem. I can tell you what's worked for other people, but you'll be the best judge of what will work for you. Let's go through some of the options together."*<br><br>*"If you were to decide to stop, what might your options be? Are there any ways you know about that have worked for other people?"*

7.8 Recognizing Readiness to Change

The first phase of MI involves *building* motivation for change. There comes a point when it is time to shift strategies from building motivation for change to *strengthening commitment* to change (Phase 2). At this point, the participant is ready to change but has not made a firm decision or commitment to do so. Miller & Rollnick (2002) note that there is often no exact, ideal moment to make this transition from Phase 1 to Phase 2, but do believe that, once a person has reached a point of readiness, there is a window of time during which change should be initiated. How long this window stays open will vary widely, but the recognition of an important discrepancy is just too uncomfortable for a participant to sustain forever. If change isn't initiated, the participant is likely to start using cognitive defensive strategies to decrease the discomfort (i.e., talk him/herself back into thinking there's not a problem with his/her smoking). Consequently, it is important to recognize and take advantage of the open window, otherwise the participant will slide backwards in the stage continuum and the counselor will have the challenge of once again bringing the participant to the point when he/she is ready to change.

There are no sure signs of an open window, but Miller & Rollnick have identified cues for identifying when to transition from Phase 1 to Phase 2. Signs of readiness to change [adapted from Miller & Rollnick, 1991, 2002] are listed below.

Decreased resistance. The participant stops arguing, interrupting, denying, or objecting.

Decreased questions about the problem. The participant seems to have enough information about smoking, and stops asking questions. There is a sense of being finished.

Resolve. The participant appears to have reached a resolution; he/she may seem more peaceful, relaxed, calm, unburdened, or settled.

Self-motivational statements (change talk). The client makes direct self-motivational statements; these may reflect a recognition that smoking is a problem, concern about continuing to smoke, an openness to change, or optimism about quitting.

Increased questions about change. The participant asks what he/she can do about his/her smoking, or about how other people have quit once they decided to do so.

Envisioning. The participant begins to talk about how life might be after quitting smoking, to anticipate difficulties if he/she were to quit, or to discuss advantages of quitting.

Experimenting. The participant may have begun experimenting with possible approaches to quitting (e.g., cutting back on cigarettes smoked, not smoking when in a typical smoking situation, or reading more self-help materials about quitting).

When there are such signs of readiness, it may be time to shift direction to the new goal of strengthening commitment. Typically, these signs will emerge gradually and subtly when the participant is in late contemplation.

7.9 Initiating Phase 2: Strengthening Commitment to Change

Note: **At this point, Counselor's shift from the ME form to Quit Prep in the TI Master for recording process data.**

7.9.1 Recapitulation

A first step in making the transition to Phase 2 is to summarize once again the participant's current situation, as reflected in your conversations to date. This is called recapitulation. The recapitulation should cover as many of the following elements as possible:

A summary of the participant's own perceptions of the problem, as reflected in his/her self-motivational statements.

A summing-up of the participant's ambivalence, including what remains positive or attractive about smoking.

A review of whatever objective evidence you have regarding the presence of risks and problems (e.g., slower time in the quarter mile, getting winded playing basketball).

A restatement of any indications the participant has offered of wanting, intending, or planning to change.

Your own assessment of the participant's situation, particularly at points where it converges with his/her own concerns.

The purpose of this summary is to draw together as many reasons for change as possible, while simultaneously acknowledging the participant's reluctance or ambivalence. The recapitulation is used as a final preparation for the transition to commitment, and leads directly into the key questions. This is usually done at a point when the participant is likely to be at a peak of problem awareness.

7.9.2 Key Questions

As in Phase 1, participants are not told what they have to do, but rather are asked what they want to do. The questions are open-ended for the purpose of getting the participant thinking and talking about stopping smoking. The participant's answers are met, typically, with reflection.

Possible key questions (Miller & Rollnick 1991, 2002)

What do you think you will do?
What does this mean about your smoking?
It must be uncomfortable for you now, seeing all this . . . . What's the next step?
What do you think has to change?
What could you do? What are your options? It sounds like things can't stay the way they are now. What are you going to do?
Of the things I've mentioned here, which for you are the most important reasons for a change? . . . How are you going to do that?
What's going to happen now? Where do we go from here?
How would you like things to turn out for you, ideally?

7.9.3 Information and Advice

Some participants may ask for information or ideas during Phase 2. It is appropriate to offer your own best advice in this circumstance, but it's best to try to get the participant to offer his/her own ideas first. Examples:

"I'll be happy to give you some ideas, but I don't want to get in the way of your own creative thinking – you're the expert on you."
"Of course, I can tell you what I think if you really want to know. But I don't want you to think that I'm trying to tell you what to do."

Another option is to qualify any suggestions you make. Present advice in a deliberately impersonal way, allowing the participant to decide whether or not it fits his/her situation. Always elicit the participant's response to the information or advice you are offering. Examples:

"I don't know if this would work for you or not, but I can tell you what has worked for other people."

"Some people have [suggestion]. I wonder whether that would work for you?"

Still another useful approach is to offer several ideas so that the participant can make a choice.

"Let me describe a number of options, and you tell me which of these makes the most sense for you."

The counselor can also elicit requests for advice from the participant. Example:

"We've talked about an awful lot today, and you seem to have been giving this a lot of thought. I wonder if there's anything you want to ask me, or if there's anything you've been wondering about."

7.10 Negotiate an Action Plan

"Action plan" refers to specific behavioral commitments made toward behavior change. Examples include setting goals, setting a stop date, identifying strategies for dealing with high-risk situations, and enlisting social support. From the participant's answers to key questions and responses to information and advice, the beginnings of an action plan may emerge.

Helping participants develop their action plan may be particularly important for smokers in our target age group. Longitudinal findings concerning the use of the processes of change provide some evidence that one reason for limited success among adolescent quitters is their poor preparedness for cessation. Adolescents in the early stages seem to move prematurely into action. This has been demonstrated in their reliance more on behavioral processes in the precontemplation to contemplation and contemplation to preparation stage transitions, rather than applying experiential processes, which more appropriately help prepare for a change (Pallonen, 1998).

Because of adolescents' inclination to move right into action, many won't immediately see a need to plan for quitting. Therefore, it may be necessary to first negotiate the need to have an action plan. Sometimes all that is needed is a simple, *"Would making a plan hurt?"* Using an easy to understand analogy about the benefits of planning may also help:

"When you go hiking in the mountains, you plan ahead – you make sure you have water, and the right shoes and clothing, and a map. And you let someone else know where you're going and when to expect you back. All those steps help ensure a safe and fun hike. Planning for stopping smoking is the same thing – you just want to know you have your bases covered. You know what to do in those situations where you usually smoke."

Some participants may feel like they want to stop but are not quite ready to commit to a stop date. They may feel more comfortable taking "intermediate steps" first, like cutting down on the number of cigarettes smoked, or even agreeing to read the self-help materials or visit the Web site. These steps may be regarded as "successive approximations" leading to stopping smoking. The counselor's goal in this case is to support progress and help the participant maintain focus.

Some participants may be feeling reluctant because they lack confidence in their ability to stop smoking (low cessation self-efficacy), or they flat-out just don't know how to go about quitting and that leaves them feeling a bit overwhelmed or hopeless, frozen in contemplation and unable to move forward. (P: *"I want to quit by the end of school. But it's hard."* C: *"How do you think you'll do that?"* P: *"I don't know. I don't know how to do it."*)

In such situations, the counselor should remember that the participant may not be aware of all the resources and assistance that the counselor and Matchbreaker can provide. Let the participant know that you can help him/her:

> *"Quitting is something you really want to do, but imagining how you would get through that difficult first week is holding you back. You know, I could help you with preparing for that if you'd like. I know a lot about how to help make quitting easier, and I have some stuff I can send you. Would you like that?"*

Developing an action plan is a process of negotiation between the participant and counselor that involves (1) setting goals, (2) assessing challenges, and (3) arriving at a plan. As a final step in negotiating an action plan, the counselor will want to (4) elicit commitment from the participant.

7.10.1 Set Goals

Motivation is, in part, a function of the discrepancy between a person's goals for his/her future and his/her present state. Setting clearly defined goals, then, is a critical step toward forward progress in the behavior change process ("envisioning the future"). Key questions include, *"How would you like for things to be different?"* or *"If you were completely successful in accomplishing what you want now, what would be changed?"*

Setting Goals

Strategies:	Examples:
The thought of never having another cigarette may seem overwhelming to some smokers, so it can be useful to convey the idea that stopping smoking is not a single task but a series of choices. Accordingly, it can be helpful to encourage the participant to set **proximate**, **attainable goals** for stopping, like getting through the urges of the first day without smoking.	*"Let's take things one step at a time. What do you think is the first step?"*
With or without previous experience with quitting, setting clearly defined proximate goals can boost the participant's self-efficacy. If he/she doesn't know where to begin, "brainstorm" or ask permission to provide some ideas for him/her to choose from.	*"How about if we take a few minutes now to list your goals? What would you like to accomplish first?*<br>or<br>*"It can be tough figuring out where to go from here. If you'd like, I can tell you what kinds of goals other people have found helpful."*
Help the participant evaluate his/her chosen goal by asking about consequences of taking this particular course of action; the participant may have some concerns that have not yet been expressed.	*"How would your life be different if you achieved this goal?"*<br>or<br>*"Do you think there are things that might go wrong with this plan?"*<br>Follow-up: *"If that happened, how might you handle it?"*
The participant's goals may not be explicitly about stopping smoking. Rather, they may be about achieving other ends which stopping can help with (e.g., "being more fit when I report to boot camp"). The counselor can use these goals to continue to motivate the participant towards the secondary goal of stopping.	*"You say that you want to be in good shape when you report to boot camp. I can see why that would motivate you to want to stop smoking now. So, let's talk about that . . . .*

7.10.2 Assess Challenges

Once goals have been clearly defined, the next step in developing an action plan is to consider what challenges the participant might face when trying to meet his/her goals.

Identifying challenges and triggers

Strategies:	Examples:
Ask participant to think about his/her triggers to smoke – in what situations will the urge to smoke be strongest? If he/she has a history of a past quit attempt, what situations posed challenges during those quit attempts? In this discussion, if the participant overlooks any relapse situations from the past, or any situations that he/she expressed concern about in the earlier sessions, the counselor asks if they should be added to the list.<br><br>[Challenges = Process Data]	*"Everyone who stops smoking faces challenges – those times when they really want a cigarette. Identifying and preparing for those challenges can really help you get through them. What do you think will be most challenging for you?"*<br><br>*"Think about when you're most likely to smoke -- Is there anything about those situations that trigger you to want a cigarette?"*<br><br>*"Once before you were able to stop smoking for 4 weeks. What was most challenging during that time – when did you most want a cigarette?" "How were you able to resist those temptations before – what got you through those 4 weeks?"*<br><br>*"Several times you've mentioned that it's really hard not to smoke when you're with certain friends. Should we put this on the list of stuff to plan for?"*
If participant has difficulty identifying challenges, ask him/her to keep track of his/her smoking for a week.	*"I can see you haven't thought much about this before. Since you really want to be prepared before you stop, you could try tracking your smoking for a week – write down when you smoke, who you're with, stuff like that. It's easy to do and folks are amazed at what they find out about themselves. I can send you a "Track your Triggers" worksheet after this call. Is that something you'd be interested in?"*

7.10.3 Plan for Stopping Smoking

Having identified what the most difficult situations are likely to be, the participant plans specific strategies for getting through each one without smoking. The counselor lets the participant propose his/her own ideas, and then the counselor brainstorms with the participant to come up with additional possibilities. The goal is to come up with a repertoire of strategies that are ***practical***, ***behavioral***, and ***specific***. Reflect the participant's ideas and ask permission to add to them, or to brainstorm more ideas. Older teens, like adults, respond well to a menu of choices; brainstorm and provide a set of possibilities from which to choose. The experience of making a personal choice often enhances motivation. Three areas to work on are:

(1) Restructure self-perceptions (self-talk),
(2) Develop strategies to cope with anticipated physical and emotional distress,
(3) Develop stopping strategies.

Examples of possible strategies are presented in the following table. When discussing strategies with participants, tell them what the strategy is intended to do, how it works, what is involved, and what to expect. Reserve choice and negotiation until after the menu of possible strategies has been reviewed. As needed, revisit confidence here, pointing out the personal strengths the participant can draw on when using his/her strategies to cope with challenges and difficult situations.

As possible courses of action are discussed, ask for the participant's best guess as to what might happen with each alternative. Examples:

> *"You say that you really need to say "NO" when your friends offer you a cigarette. How do you think your friends might react?"*

The goal here is to form as realistic a plan as possible. For some participants, your most effective strategy will be to focus on just one or two of his/her most challenging or high risk situations. Trying to cover all possible challenges will just overwhelm the participant; planning for just one or two situations may be all that the participant will allow. So, prioritize and respond to the participant's lead when planning for stopping. You can always go over more strategies, or different situations, in the first cessation support call.

> ***Tip:* Always ask permission. Don't provide advice too enthusiastically. Offer multiple options, and encourage personal choice. Always elicit the participant's reaction: *"What do you think of that idea? Is it likely to work for you?"***

Planning for stopping smoking

Strategy :	Examples:
Prepare "emotional self"	Make a list of reasons for quitting (problems with smoking/benefits of quitting).<br>Plan activities to keep busy, especially the first day.<br>Have worry stone or other objects to occupy your hands.<br>Argue against the cigarette ad.<br>Argue against any "just one cigarette won't hurt" thoughts.<br>Begin to visualize self as a non-smoker.
Prepare surroundings	Remove all smoking stuff from your room (e.g., ashtrays, lighters, matches, stashes); make it a smoke-free zone.<br>Clean out the car — clean the ashtray, vacuum inside, wipe down windows & dash.<br>Use room and car deodorizers if either still smell like smoke.

Strategy :	Examples:
Prepare "physical self"	Reduce caffeine intake (drink caffeine-free pop [e.g., 7-up, Sprite, caffeine-free Coke, etc.], coffee).<br>Get lots of rest.<br>Drink plenty of fluids (fruit juice, water).<br>Have substitutes on hand (e.g., carrots, pretzel sticks, straws).
Manage urges and cravings	*"Let's spend a minute thinking about ways to deal with urges to smoke. What do you see yourself doing when you want a cigarette?" "What do you think of these suggestions: some participants suck on lifesavers or drinking straws, so that they have something in their mouths when they get the urge to smoke."*<br>Learn the "Five D's":<br>— Deep breathing (practice deep breathing exercises)<br>— Drink water<br>— Distract (create distractions from smoking: call, e-mail, or IM a friend, go to a movie, play a video game, read a book)<br>— Distance (distance self from smoking situations by changing routines, e.g., showering as soon as one wakes up, or brushing teeth immediately after eating, or avoiding high-risk situations or people in the first few days/weeks following the quit date)<br>— Delay (Most urges and cravings pass in 3-5 minutes, so learn to "urge surf")<br>Learn/practice positive "self-talk" — recognize and counter potentially sabotaging thoughts *("You're going to go completely crazy if you go a whole day without cigarettes! If that were another person talking, what would you say to her?")*
Elicit social support – tell friends & family of intentions to quit and ask in advance for their help and patience.<br><br>If it's comfortable for the TP, he/she can ask smoking household members if they will help by smoking less/not at all in the home.	*"Have you told anyone about your plans for quitting smoking?" "How do your friends feel about you quitting smoking?" "What could others do that would be most helpful to you?"*<br><br>*"If you're comfortable talking to your parents about your smoking, tell them about your plans to quit. Let them know how they can help."*<br><br>*"Be assertive in asking for what you need, but ask respectfully, in a way that increases the likelihood that your request for help will be honored."*
Take a problem-solving approach: State the anticipated problem or challenge, and elicit several possible solutions<br>Help TP evaluate pros & cons of different approaches (also can use the 1-10 scale to evaluate confidence in particular situations)	*"So you think parties will be your biggest challenge because you like smoking when you're drinking, plus almost everybody smokes at parties. What would make it okay for you not to smoke at a party, if that's what you wanted to do?"*

Strategy :	Examples:
Every smoker gets something he/she thinks she needs from smoking. Explore with the participant what he/she gets from his/her smoking and come up with substitutes or alternatives.	Habit: *"Sounds like, for you, smoking may be automatic. Sometimes you may not realize you even have a cigarette in your hand. Your smoking may almost be ritual: mostly, you smoke at certain times or when doing certain things: while on the phone, when drinking or at parties, with a coffee. If this sounds like you, try to keep track of your smoking patterns for a week. That way, you can easily see when you smoke and what -- or who -- triggers your smoking. Once you identify your triggers, you'll be better able to come up with other ways to respond besides smoking."*<br><br>Pleasure: *"Sounds like you know the health problems associated with smoking, but you like to smoke. You associate smoking with pleasurable situations or events. What other pleasant activities could you substitute for smoking?" (suggestions: Treat yourself to a new soft drink or special tea (non-caffeine is best). Spend time with friends, do things you like to do.)*<br><br>Relaxation: *"Sounds like you smoke when you are worried, tense, or nervous -- smoking helps you deal with stress. What might work for you in these instances, when you don't want to smoke?" (ideas: deep breathing exercise, listen to music)*<br><br>Something to do with your hands: *"Sounds like smoking gives you something to do with your hands. You like to handle the cigarette, the mechanics of smoking. What else can you do to keep your hands bus?" (suggestions: Carry a worry stone or a marble, play with a coin, keep a plastic straw cut to cigarette size to play with, or keep a pad and pencil handy for doodling.)*<br><br>Stimulation: *Sounds like what you like about smoking is the stimulation you get from the nicotine. The problem is, nicotine is also a natural depressant; at the same time that it increases your heart rate and blood pressure, it decreases you neuron activity (that's your brain on nicotine!). There are easier, cheaper, and healthier way to boost your energy. What else can you do to get that feeling you like?" (suggestions: Try <u>exercise</u>; pick something you like to do. Try taking a brisk walk: the combination of the exercise and fresh air are an awesome pick-me-up.)*
Help the participant plan for slips. Talking about slips will normalize them. It helps the participant know he/she will face challenges, and to know that slipping up and having one cigarette does not mean he/she has to go back to smoking. It is not a failure, just a slip. Some participants, particularly those who've quit and failed before may be fearful of slips.	*"In talking to other people about their quitting, I hear it's not uncommon to slip up and have a cigarette. How do you think you'd feel if that happened to you?"*<br>*"What could you do to avoid that from happening?"*<br><br>*"What kind of situations were hard for you last time you quit?" What worked for you then?"*<br><br>*"How did you feel when you slipped up and smoked that cigarette?"*<br>*"What would you do differently next time?"*<br><br>*"Would you like to hear what's worked for other people?"*

Strategy :	Examples:
Present an optimistic view of slips to the participant; emphasize that he/she is in control.	*"You know, slips are an expected part of new learning, to help you know more about your needs and how to stay quit. For example, now you know that under certain circumstances, this particular situation (e.g., drinking at a party) can be a danger to you. So, what can you do next time you're in a similar situation but don't want to smoke?"*
Normalize withdrawal – Provide reassurance that withdrawal is temporary	*"Understanding the changes your body undergoes when you are quitting smoking is the first step towards dealing with them. Most of the time we refer to all of the discomforts as withdrawal symptoms, but some of the changes you notice are really symptoms of recovery. The good news is that withdrawal symptoms generally don't last long. Withdrawal is temporary!!"*<br><br>*"Most of the people I talk to don't even experience withdrawal – it's really pretty surprising."*<br><br>*"Is there any particular feeling or symptom that worries you?"*
Plan a self-reward on the first day of quitting	*"Rewarding yourself with a special treat or activity provides positive reinforcement and a counterbalance to the loss you may feel when you stop smoking. How would you reward yourself for stopping for a whole day?"*

7.10.4 Arrive at a Plan

On the basis of the negotiation discussions, summarize a plan that seems to fit with the participant's goals, needs, intentions, and beliefs. Match strategies to the participant's major challenges – trying to incorporate all of these strategies into a single quit plan might overwhelm the participant. [Coping strategies = Process Data]
Example:

"Let me see if I can summarize where you are, then. You wanted to know about different ways people could quit smoking and we've talked about several different possibilities. You're thinking that it's best if you just quit cold turkey. You plan to get your friends to help you stay away from cigarettes, and you're prepared to clean your room and car out, so you don't have a stash of cigarettes to tempt you. You're also planning to practice the 5-D's. You're still a little nervous about this plan, I think, but you think you really need to make a change now, before graduation, and this sounds like the one you've chosen. Have I missed anything?"

7.10.5 Elicit Commitment

The plan summary may bring the participant right to the point of commitment. Verbal agreement may be as simple as asking, *"Is this what you want to do?"* and getting a "Yes."

Commitment is also reflected in action. It may be possible to plan immediate steps that can be taken to implement the plan, i.e., set a date to stop smoking. (When talking to a participant who smokes only occasionally, consider rewording this goal in terms more meaningful to him/her, i.e., make a decision not to try smoking again from this point on.) If the participant still seems reluctant, don't push; explore any remaining ambivalence. Don't press for a commitment; instead, roll with the process. Example:

"If you're not ready yet, then it might be best to wait a bit before making this commitment. It's too important to rush. Take a week to think about all we've discussed and I'll call you in a week and we can talk more about it. What would be a good time for me to call?")

Some participants might firmly state they want to quit and quit soon, but they are vague about setting a firm date for stopping. They may feel like setting a quit date is boxing them into a corner, like you're nailing them down about stopping smoking. Try to explore these feelings with the participant. For example, if the participant says he'll quit in a month, reflect:

"Okay, so what you're telling me is that by November 30th, you want to be completely stopped."
(Pause. If participant remains silent, continue.*)*
"Okay, what's going to make it a good day to stop at the end of the month?"

The participant may have a sound reason, e.g., he's cutting back his work hours so won't be around as much smoking, or basketball practice starts and he needs to be in shape. In that case, proceed with setting a quit date. If the participant is vague about reasons why the end of the month is good, he/she may not be committed to stopping at the end of the month. Explore that possibility:
"Sounds like you're pretty committed to stopping smoking, but still might not be quite ready to set a date for stopping. What are your thoughts on that?"
"What's stopping you from setting a date for stopping smoking?"

Explore the participant's reluctance to set a quit date. Fall back on Motivational Interviewing as needed, to support the needs of the participant.

7.10.6 Setting Short-Term Goals In Preparation For Stopping For Good

If the participant is committed to stopping smoking but reluctant to set a quit date, consider asking him/her to set a short-term goal, e.g., quitting for 24 or 48 hours (if currently smoking daily), or going a weekend without smoking, even at parties (if a social or infrequent smoker). Short-term goals can help move the participant to set a stop date. These steps may be regarded as “successive approximations” leading to stopping smoking. The counselor’s goal is to support progress and help the participant maintain focus.

> *Tip:* Participants may have more confidence in their own ability to stop smoking, and have more confidence in their quit strategies, if they “try them out first.” Encourage the participant to try some of the strategies in his/her plan before your next call, just to try them out. With a little practice, the new strategy can become as second nature as smoking. *“Why don’t you just try out listening to music instead of smoking a cigarette when you’re feeling stressed at home? That way you can see how it works for you before you quit.”*

7.10.7 Set a Stop Date

When the participant feels that all questions have been answered and that a workable plan has been developed, then he/she and the counselor should agree on a date to implement the plan; this is called the “stop date.” (*“How would you feel about setting a date to stop smoking?”*) If the participant included anything in his/her action plan that is best done ahead of the stop date, e.g., cleaning his/her car or room, getting rid of cigarettes, the counselor will ask the participant about the best time for him/her to do these tasks (for example, the day before the stop date) to prompt the participant to include these steps in his/her planning.

The counselor then schedules an appointment to call back for cessation support on [preferred] or immediately after the stop date. (From that date forward, the counselor will schedule calls in accordance with cessation support call schedule.) The participant’s expectation of the Stop Day call provides a degree of accountability that may help to overcome any ambivalence he/she may feel about following through with stopping. It also lets the participant know that the counselor will be with him/her during this big step. By setting a stop date, the participant transitions with the counselor from Motivation Enhancement to Cessation Support.

> When working with participants who smoke only occasionally, remember to tailor goals and strategies accordingly.

Record the Stop Date. [Process data]

7.11 Wrap-up the Call

When wrapping up a call to a participant, the counselor will do the following

with participants in precontemplation, contemplation	with participants in preparation
(1) Summarize the discussion using "you" language. The counselor will use reflective statements to emphasize concerns and intentions to change that were voiced by the TP. The counselor will offer encouragement and support for these as appropriate.	(1) Summarize the discussion using "you" language. The counselor will use reflective statements to emphasize concerns, goals and specific plans for stopping smoking that were voiced by the TP. The counselor will offer encouragement and support for these as appropriate.
(2) Explore any actions taken in the call and discuss any "next steps" to be taken before the next counseling session.	(2) Explore any actions taken in the call and discuss any "next steps" (e.g., cleaning room, getting rid of cigarettes) to be taken before the next call.
(3) Offer to send a brief summary of the call, along with some self-help materials related to the day's discussion, to the address of his/her choice.	(3) Offer to send a brief summary of his/her action plan, along with some self-help materials ("ButtsOut #2") and a free "Quit Kit" (with helpful aids for stopping smoking such as gum, squeeze ball, water bottle, etc.), to the address of his/her choice.
(4) As appropriate, refer the participant to specific pages of the Web site, or to specific portions of self-help materials.	(4) As appropriate, refer the participant to specific pages of the Web site, or to specific portions of self-help materials.
(5) Particularly when talking to precontemplative and contemplative smokers, it is important for the counselor to always leave the door open for further contact, without pressuring the participant to stop smoking.	(5) Particularly when talking to precontemplative and contemplative smokers, it is important for the counselor to always leave the door open for further contact, without pressuring the participant to stop smoking.
(6) Set the date and time for the next call appointment. When making the next appointment, the counselor will prompt the participant to think about any work, sports, or rehearsal schedules; this might help increase the likelihood of setting a time for an appointment that will be met by the participant.	(6) Confirm that the counselor will be calling on the TP's Stop Day (the counselor will set date and time for call appointment) to check in and see how things are going. When making the next call appointment, the counselor will prompt the participant to think about any work, sports, or rehearsal schedules; this might help increase the likelihood of setting a time for an appointment that will be met by the participant.

8. CESSATION SUPPORT: PREPARING TO STOP

Note: If participant is transitioning from Initial Call directly to preparing to stop, follow these guidelines and shift from the Intro form to Quit Prep in the TI Master for recording process data.

Preparation Call Elements

(If Preparation is transition from Introduction Call)

- Assess unique situation
- Strengthen Commitment to Change *(Preparation)*
- Negotiate an Action Plan *(Preparation)*
- Wrap-up the call

8.1 Assess the Participant's Unique Smoking Situation

As we learn more about the participant, we'll want to also know more about the role smoking plays in his/her life. Assessing the participant's unique situation allows counselors to identify how to best proceed with the smoker intervention telephone calls; it provides useful information that the counselor can use when tailoring the intervention to the individual participant. The assessment also helps the counselor demonstrate that he/she wants to know what smoking means to the participant, it provides a common understanding shared by the counselor and the participant, both of which help build rapport, and it gets the participant to start thinking reflectively about his/her smoking.

In addition to readiness to stop smoking (already assessed), important aspects of the participant's unique situation are the importance he/she places on stopping, and his/her confidence in stopping. The participant's readiness to change may be influenced by his/her perceptions of the importance of stopping and confidence in his/her ability to do so. For example, a participant may be convinced of the personal value of stopping (*importance*), but not feel confident about mastering the skills necessary to stop (*confidence)*. Alternately, other participants may have mixed feelings about the importance of stopping (*importance*), but say that they could stop fairly easily (*confidence)* if they really wanted to. Learning the participant's perception of importance, confidence, and readiness helps the counselor to tailor the counseling session and match the call content to the individual. To further understand the participant's smoking, the intervention will also assess frequency of smoking, and level of nicotine dependence (this information may be drawn out through informal conversation during the call or via formal assessment).

Assess the smoker's unique situation

Strategies:	Examples:
Begin by asking the participant to describe how smoking fits into his/her lifestyle, or to describe a typical day.<br><br>If participant has indicated he/she has already decided to stop, modify statements accordingly, e.g., "*Before we talk about your decision to stop, I'd like to understand better how smoking fits into your life. Can you describe a typical situation when you smoke?*")	*"I'd like to understand better how smoking fits in to your life. Can you describe a typical situation when you smoke?"*<br>Or<br>*"Let's spend the next few minutes going through a typical day for you. For example, describe today . . . what did you do today, how did you feel?"* [As participant brings up life situations, demonstrate interest.] *"So, you have to be at school by 7:30 a.m. for marching band practice. That's early! What instrument do you play?"* [As the opportunity arises, bring up smoking.] *"So, you're a trumpet player. Does your smoking ever impact your music, like affect how well you blow your horn?"* or be more general: *"So, how did smoking fit in to your day today?"*
For **<u>infrequent smokers</u>**, consider asking about specific situations when they may smoke, e.g., weekends or parties with friends.	*"Sounds like you only smoke once in a while, like when you're at a party. Tell me more about situations like that."*
Asking about a typical day provides a natural opportunity to follow-up with questions about level of smoking:<br>Process data item	*"Sounds like from your description that you're smoking (every day/weekly/monthly). Is that right? . . . About how many cigarettes would you say you smoke each (day/week/month)?"*
(If participant is in preparation stage) Explore the participant's motivations for stopping smoking.	*"Now, you mentioned that you're thinking about quitting smoking soon, like in the next 30 days or so. What has led you to that decision?"*
Assess importance of stopping smoking. Record the answer<br>Process data item	*"Right now, today, how important is it to you personally to stop smoking? If "0" was not important and "10" was very important, what number would you give yourself?"*
Note: If value placed on importance is low, work on building importance for stopping.	0 - 1 - 2 - 3 - 4 - 5 - 6 - 7 - 8 - 9 - 10<br><br>or, if participant is talking about stopping (i.e., in preparation), *"It sounds like stopping smoking might be pretty important to you. Can you give me a feel about how important? Right now, today, how important is it to you personally to stop? If "0" was not important and "10" was very important, what number would you give yourself?"*
Assess the participant's confidence in his/her ability to stop smoking. Record the answer.<br>Process data item<br><br>Note: If confidence is low, work on building confidence.	*"If you decided right now to stop smoking, how confident are you that you would succeed? If "0" is not at all confident and "10" is very confident, what number would you give yourself?"*<br><br>0 - 1 - 2 - 3 - 4 - 5 - 6 - 7 - 8 - 9 - 10

Strategies:	Examples:
Assess the participant's current dependence on nicotine. Record the answer.<br><br>Process data item	*"On a typical day, how soon after waking up do you have the urge to smoke your first cigarette?"*<br>a. As soon as I wake up<br>b. Within about ½ hour of waking<br>c. An hour or two after I wake up<br>d. Over 2 hours after waking

8.2 Initiating Phase 2: Strengthening Commitment to Change

8.2.1 Recapitulation

A first step in making the transition to Phase 2 is to summarize once again the participant's current situation, as reflected in your conversations to date. This is called recapitulation. The recapitulation should cover as many of the following elements as possible:

A summary of the participant's own perceptions of the problem, as reflected in his/her self-motivational statements.

A summing-up of the participant's ambivalence, including what remains positive or attractive about smoking.

A review of whatever objective evidence you have regarding the presence of risks and problems (e.g., slower time in the quarter mile, getting winded playing basketball).

A restatement of any indications the participant has offered of wanting, intending, or planning to change.

Your own assessment of the participant's situation, particularly at points where it converges with his/her own concerns.

The purpose of this summary is to draw together as many reasons for change as possible, while simultaneously acknowledging the participant's reluctance or ambivalence. The recapitulation is used as a final preparation for the transition to commitment, and leads directly into the key questions. This is usually done at a point when the participant is likely to be at a peak of problem awareness.

8.2.2 Key Questions

As in Phase 1, participants are not told what they have to do, but rather are asked what they want to do. The questions are open-ended for the purpose of getting the participant thinking and talking about stopping smoking. The participant's answers are met, typically, with reflection.

<u>Possible key questions (Miller & Rollnick 1991, 2002)</u>

What do you think you will do?
What does this mean about your smoking?
It must be uncomfortable for you now, seeing all this . . . . What's the next step?
What do you think has to change?
What could you do? What are your options?
It sounds like things can't stay the way they are now. What are you going to do?
Of the things I've mentioned here, which for you are the most important reasons for a change? . . . How are you going to do that?
What's going to happen now? Where do we go from here?
How would you like things to turn out for you, ideally?

8.2.3 Information and Advice

Some participants may ask for information or ideas during Phase 2. It is appropriate to offer your own best advice in this circumstance, but it's best to try to get the participant to offer his/her own ideas first. Examples:

"I'll be happy to give you some ideas, but I don't want to get in the way of our own creative thinking – <u>you're</u> the expert on you."
"Of course, I can tell you what I think if you really want to know. But I don't want you to think that I'm trying to tell you what to do."

Another option is to qualify any suggestions you make. Present advice in a deliberately impersonal way, allowing the participant to decide whether or not it fits his/her situation. Always elicit the participant's response to the information or advice you are offering. Examples:

"I don't know if this would work for you or not, but I can tell you what has worked for other people."
"Some people have [suggestion]. I wonder whether that would work for you?"

Still another useful approach is to offer several ideas so that the participant can make a choice.

"Let me describe a number of options, and you tell me which of these makes the most sense for you."

The counselor can also elicit requests for advice from the participant. Example:

"We've talked about an awful lot today, and you seem to have been giving this a lot of thought. I wonder if there's anything you want to ask me, or if there's anything you've been wondering about."

8.3 Negotiate an Action Plan

"Action plan" refers to specific behavioral commitments made toward behavior change. Examples include setting goals, setting a stop date, identifying strategies for dealing with high-risk situations, and enlisting social support. From the participant's answers to key questions and responses to information and advice, the beginnings of an action plan may emerge.

Helping participants develop their action plan may be particularly important for smokers in our target age group. Longitudinal findings concerning the use of the processes of change provide some evidence that one reason for limited success among adolescent quitters is their poor preparedness for cessation. Adolescents in the early stages seem to move prematurely into action. This has been demonstrated in their reliance more on behavioral processes in the precontemplation to contemplation and contemplation to preparation stage transitions, rather than applying experiential processes, which more appropriately help prepare for a change (Pallonen, 1998).

Because of adolescents' inclination to move right into action, many won't immediately see a need to plan for quitting. Therefore, it may be necessary to first negotiate the need to have an action plan. Using an easy to understand analogy about the benefits of planning may help:

"When you go hiking in the mountains, you plan ahead – you make sure you have water, and the right shoes and clothing, and a map. And you let someone else know where you're going and when to expect you back. All those steps help ensure a safe and fun hike. Planning for stopping smoking is the same thing – you just want to know you have your bases covered. You know what to do in those situations where you usually smoke."

Some participants may feel like they want to stop but are not quite ready to commit to a stop date. They may feel more comfortable taking "intermediate steps" first, like cutting down on the number of cigarettes smoked, switching brands, or even agreeing to read the self-help materials or visit the Web site. These steps may be regarded as "successive approximations" leading to stopping smoking. The counselor's goal in this case is to support progress and help the participant maintain focus.

Some participants may be feeling reluctant because they lack confidence in their ability to stop smoking (low cessation self-efficacy), or they flat-out just don't know how to go about

quitting and that leaves them feeling a bit overwhelmed or hopeless, frozen in contemplation and unable to move forward. (P: *"I want to quit by the end of school. But it's hard."* C: *"How do you think you'll do that?"* P: *"I don't know. I don't know how to do it."*)

In such situations, the counselor should remember that the participant may not be aware of all the resources and assistance that the counselor and Matchbreaker can provide. Let the participant know that you can help him/her:

> *"Quitting is something you really want to do, but imagining how you would get through that difficult first week is holding you back. You know, I could help you with preparing for that if you'd like. I know a lot about how to help make quitting easier, and I have some stuff I can send you. Would you like that?"*

Developing an action plan is a process of negotiation between the participant and counselor that involves (1) setting goals, (2) assessing challenges, and (3) arriving at a plan. As a final step in negotiating an action plan, the counselor will want to (4) elicit commitment from the participant.

8.3.1 Set Goals

Motivation is, in part, a function of the discrepancy between a person's goals for his/her future and his/her present state. Setting clearly defined goals, then, is a critical step toward forward progress in the behavior change process ("envisioning the future"). Key questions include, *"How would you like for things to be different?"* or *"If you were completely successful in accomplishing what you want now, what would be changed?"*

Setting Goals

Strategies:	Examples:
The thought of never having another cigarette may seem overwhelming to some smokers, so it can be useful to convey the idea that stopping smoking is not a single task but a series of choices. Accordingly, it can be helpful to encourage the participant to set **proximate**, **attainable goals** for stopping, like getting through the urges of the first day without smoking.	*"Let's take things one step at a time. What do you think is the first step?"*
With or without previous experience with quitting, setting clearly defined proximate goals can boost the participant's self-efficacy. If he/she doesn't know where to begin, "brainstorm" or ask permission to provide some ideas for him/her to choose from.	*"How about if we take a few minutes now to list your goals? What would you like to accomplish first?*<br>or<br>*"It can be tough figuring out where to go from here. If you'd like, I can tell you what kinds of goals other people have found helpful."*

Strategies:	Examples:
Help the participant evaluate his/her chosen goal by asking about consequences of taking this particular course of action; the participant may have some concerns that have not yet been expressed.	*"How would your life be different if you achieved this goal?"* or *"Do you think there are things that might go wrong with this plan?"* Follow-up*: "If that happened, how might you handle it?"*
The participant's goals may not be explicitly about stopping smoking. Rather, they may be about achieving other ends which stopping can help with (e.g., "being more fit when I report to boot camp"). The counselor can use these goals to continue to motivate the participant towards the secondary goal of stopping.	*"You say that you want to be in good shape when you report to boot camp. I can see why that would motivate you to want to stop smoking now. So, let's talk about that . . . .*

8.3.2 Assess Challenges

Once goals have been clearly defined, the next step in developing an action plan is to consider what challenges the participant might face when trying to meet his/her goals.

Identifying challenges and triggers

Strategies:	Examples:
Ask participant to think about his/her triggers to smoke – in what situations will the urge to smoke be strongest? If he/she has a history of a past quit attempt, what situations posed challenges during those quit attempts? In this discussion, if the participant overlooks any relapse situations from the past, or any situations that he/she expressed concern about in the earlier sessions, the counselor asks if they should be added to the list.<br><br>[Challenges = Process Data]	*"Everyone who stops smoking faces challenges – those times when they really want a cigarette. Identifying and preparing for those challenges can really help you get through them. What do you think will be most challenging for you?"*<br><br>*"Think for a minute about when you are most likely to smoke. Is there anything about those times or situations that trigger you to want a cigarette?"*<br><br>*"Once before you were able to stop smoking for 4 weeks. What was most challenging during that time – when did you most want a cigarette?" "How were you able to resist those temptations before – what got you through those 4 weeks?"*<br><br>*"Several times you've mentioned that it's really hard not to smoke when you're with certain friends. Should we put this on the list of stuff to plan for?"*
If participant has difficulty identifying challenges, ask him/her to keep track of his/her smoking for a week.	*"I can see you haven't thought much about this before. Since you really want to be prepared before you stop, you could try tracking your smoking for a week – write down when you smoke, who you're with, stuff like that. It's an easy exercise and folks are amazed at what they find out about themselves. I can send you a "Track your Triggers" worksheet after this call, or if you want, you can print one off of our Web site. Is that something you'd be interested in?"*

8.3.3 Plan for Stopping Smoking

Having identified what the most difficult situations are likely to be, the participant plans specific strategies for getting through each one without smoking. The counselor lets the participant propose his/her own ideas, and then the counselor brainstorms with the participant to come up with additional possibilities. The goal is to come up with a repertoire of strategies that are ***practical***, ***behavioral***, and ***specific***. Reflect the participant's ideas and ask permission to add to them, or to brainstorm more ideas. Older teens, like adults, respond well to a menu of choices; brainstorm and provide a set of possibilities from which to choose. The experience of making a personal choice often enhances motivation. Three areas to work on are:

(1) Restructure self-perceptions (self-talk),
(2) Develop strategies to cope with anticipated physical and emotional distress,
(3) Develop stopping strategies.

Examples of possible strategies are presented in the following table. When discussing strategies with participants, tell them what the strategy is intended to do, how it works, what is involved, and what to expect. Reserve choice and negotiation until after the menu of possible strategies has been reviewed. As needed, revisit confidence here, pointing out the personal strengths the participant can draw on when using his/her strategies to cope with challenges and difficult situations.

As possible courses of action are discussed, ask for the participant's best guess as to what might happen with each alternative. Examples:

> *"You say that you really need to say "NO" when your friends offer you a cigarette. How do you think your friends might react?"*

The goal here is to form as realistic a plan as possible. For some participants, your most effective strategy will be to focus on just one or two of his/her most challenging or high risk situations. Trying to cover all possible challenges will just overwhelm the participant; planning for just one or two situations may be all that the participant will allow. So, prioritize and respond to the participant's lead when planning for stopping. You can always go over more strategies, or different situations, in the first cessation support call.

> ***Tip:*** **Always ask permission. Don't provide advice too enthusiastically. Offer multiple options, and encourage personal choice. Always elicit the participant's reaction: *"What do you think of that idea? Is it likely to work for you?"***

Planning for stopping smoking

Strategy :	Examples:
Prepare “emotional self”	Make a list of reasons for quitting (problems with smoking/benefits of quitting).<br>Plan activities to keep busy, especially the first day.<br>Have worry stone or other objects to occupy your hands.<br>Argue against the cigarette ad.<br>Argue against any “just one cigarette won’t hurt” thoughts.<br>Begin to visualize self as a non-smoker.
Prepare surroundings	Remove all smoking stuff from your room (e.g., ashtrays, lighters, matches, stashes); make it a smoke-free zone.<br>Clean out the car — clean the ashtray, vacuum inside, wipe down windows & dash.<br>Use room and car deodorizers if either still smell like smoke.
Prepare “physical self”	Reduce caffeine intake (drink caffeine-free pop [e.g., 7-up, Sprite, caffeine-free Coke, etc.], coffee).<br>Get lots of rest.<br>Drink plenty of fluids (fruit juice, water).<br>Have substitutes on hand (e.g., carrots, pretzel sticks, straws).
Manage urges and cravings	*“Let’s spend a minute thinking about ways to deal with urges to smoke. What do you see yourself doing when you want a cigarette?” “What do you think of these suggestions: some participants suck on lifesavers or drinking straws, so that they have something in their mouths when they get the urge to smoke.”*<br>Learn the “Five D’s”:<br>— Deep breathing (practice deep breathing exercises)<br>— Drink water<br>— Distract (create distractions from smoking: call, e-mail, or IM a friend, go to a movie, play a video game, read a book)<br>— Distance (distance self from smoking situations by changing routines, e.g., showering as soon as one wakes up, or brushing teeth immediately after eating, or avoiding high-risk situations or people in the first few days/weeks following the quit date)<br>— Delay (Most urges and cravings pass in 3-5 minutes, so learn to “urge surf”)<br>Learn/practice positive “self-talk” — recognize and counter potentially sabotaging thoughts *(“You’re going to go completely crazy if you go a whole day without cigarettes! If that were another person talking, what would you say to her?”)*

Strategy :	Examples:
Elicit social support – tell friends & family of intentions to quit and ask in advance for their help and patience.	*“Have you told anyone about your plans for quitting smoking?” “How do your friends feel about you quitting smoking?” “What could others do that would be most helpful to you?”*
If it’s comfortable for the TP, he/she can ask smoking household members if they will help by smoking less/not at all in the home.	*“If you’re comfortable talking to your parents about your smoking, tell them about your plans to quit. Let them know how they can help.”*<br><br>*“Be assertive in asking for what you need, but ask respectfully, in a way that increases the likelihood that your request for help will be honored.”*
Take a problem-solving approach: State the anticipated problem or challenge, and elicit several possible solutions<br>Help TP evaluate pros & cons of different approaches (also can use the 1-10 scale to evaluate confidence in particular situations)	*“So you think parties will be your biggest challenge because you like smoking when you’re drinking, plus almost everybody smokes at parties. What would make it okay for you not to smoke at a party, if that’s what you wanted to do?”*
Every smoker gets something he/she thinks she needs from smoking. Explore with the participant what he/she gets from his/her smoking and come up with substitutes or alternatives.	Habit: *“Sounds like, for you, smoking may be automatic. Sometimes you may not realize you even have a cigarette in your hand. Your smoking may almost be ritual: mostly, you smoke at certain times or when doing certain things: while on the phone, when drinking or at parties, with a coffee. If this sounds like you, try to keep track of your smoking patterns for a week. That way, you can easily see when you smoke and what -- or who -- triggers your smoking. Once you identify your triggers, you’ll be better able to come up with other ways to respond besides smoking.”*<br><br>Pleasure: *“Sounds like you know the health problems associated with smoking, but you like to smoke. You associate smoking with pleasurable situations or events. What other pleasant activities could you substitute for smoking?” (suggestions: Treat yourself to a new soft drink or special tea (non-caffeine is best). Spend time with friends, do things you like to do.)*<br><br>Relaxation: *“Sounds like you smoke when you are worried, tense, or nervous -- smoking helps you deal with stress. What might work for you i these instances, when you don’t want to smoke?” (ideas: deep breathing exercise, listen to music)*<br><br>Something to do with your hands: *“Sounds like smoking gives you something to do with your hands. You like to handle the cigarette, the mechanics of smoking. What else can you do to keep your hands bus?” (suggestions: Carry a worry stone or a marble, play with a coin, keep a plastic straw cut to cigarette size to play with, or keep a pad and pencil handy for doodling.)*

Strategy :	Examples:
	Stimulation: *Sounds like what you like about smoking is the stimulation you get from the nicotine. Smoking helps you wake up and keeps you alert. The problem is, nicotine is also a natural depressant; at the same time that it increases your heart rate and blood pressure, it decreases you neuron activity (that's your brain on nicotine!). There are easier, cheape and healthier way to boost your energy. What else can you do to get that feeling you like?" (suggestions: Try exercise; pick something you like to do. Try taking a brisk walk: the combination of the exercise and fresh ai are an awesome pick-me-up.)*
Help the participant plan for slips. Talking about slips will normalize them. It helps the participant know he/she will face challenges, and to know that slipping up and having one cigarette does not mean he/she has to go back to smoking. It is not a failure, just a slip. Some participants, particularly those who've quit and failed before may be fearful of slips.	*"In talking to other people about their quitting, I hear it's not uncommon to slip up and have a cigarette? How do you think you'd feel if that happened to you?"*<br>*"What could you do to avoid that from happening?"*<br><br>*"What kind of situations were hard for you last time you quit?" What worked for you then?"*<br><br>*"How did you feel when you slipped up and smoked that cigarette?"*<br>*"What would you do differently next time?"*<br><br>*"Would you like to hear what's worked for other people?"*
Present an optimistic view of slips to the participant; emphasize that he/she is in control.	*"You know, slips are an expected part of new learning, to help you know more about your needs and how to stay quit. For example, now you know that under certain circumstances, this particular situation (e.g., drinking at a party) can be a danger to you. So, what can you do next time you're in a similar situation but don't want to smoke?"*
Normalize withdrawal – Provide reassurance that withdrawal is temporary	*"Understanding the changes your body undergoes when you are quitting smoking is the first step towards dealing with them. Most of the time we refer to all of the discomforts as withdrawal symptoms, but some of the changes you notice are really symptoms of recovery. The good news is that withdrawal symptoms generally don't last long. Withdrawal is temporary!!"*<br><br>*"Most of the people I talk to don't even experience withdrawal – it's really pretty surprising."*<br><br>*"Is there any particular feeling or symptom that worries you?"*
Plan a self-reward on the first day of quitting	*"Rewarding yourself with a special treat or activity provides positive reinforcement and a counterbalance to the loss you may feel when you stop smoking. How would you reward yourself for stopping for a whole day?"*

8.3.4 Arrive at a Plan

On the basis of the negotiation discussions, summarize a plan that seems to fit with the participant's goals, needs, intentions, and beliefs. Match strategies to the participant's major challenges – trying to incorporate all of these strategies into a single quit plan might overwhelm the participant. [Coping strategies = Process Data] Example:

"Let me see if I can summarize where you are, then. You wanted to know about different ways people could quit smoking and we've talked about several different possibilities. You're thinking that it's best if you just quit cold turkey. You plan to get your friends to help you stay away from cigarettes, and you're prepared to clean your room and car out, so you don't have a stash of cigarettes to tempt you. You're also planning to practice the 5-D's. You're still a little nervous about this plan, I think, but you think you really need to make a change now, before graduation, and this sounds like the one you've chosen. Have I missed anything?"

8.3.5 Elicit Commitment

The plan summary may bring the participant right to the point of commitment. Verbal agreement may be as simple as asking, *"Is this what you want to do?"* and getting a "Yes."

Commitment is also reflected in action. It may be possible to plan immediate steps that can be taken to implement the plan, i.e., set a quit date. If the participant still seems reluctant, don't push; explore any remaining ambivalence. Don't press for a commitment; instead, roll with the process. Example:

"If you're not ready yet, then it might be best to wait a bit before making this commitment. It's too important to rush. Take a week to think about all we've discussed and I'll call you in a week and we can talk more about it. What would be a good time for me to call?")

Some participants might firmly state they want to quit and quit soon, but they are vague about setting a firm date for stopping. They may feel like setting a quit date is boxing them into a corner, like you're nailing them down about stopping smoking. Try to explore these feelings with the participant. For example, if the participant says he'll quit in a month, reflect:

"Okay, so what you're telling me is that by November 30th, you want to be completely stopped."
(Pause. If participant remains silent, continue.*)*
"Okay, what makes it November 30th a good day to stop – what will be different at the end of the month?"

The participant may have a sound reason, e.g., he's cutting back his work hours so won't be around as much smoking, or basketball practice starts and he needs to be in shape. In that case, proceed with setting a quit date. If the participant is vague about reasons why the end of the month is good, he/she may not be committed to stopping at the end of the month. Explore that possibility:
"Sounds like you're pretty committed to stopping smoking, but still might not be quite ready to set a date for stopping. What are your thoughts on that?"
"What's stopping you from setting a date for stopping smoking?"

Explore the participant's reluctance to set a quit date. Fall back on Motivational Interviewing as needed, to support the needs of the participant.

8.3.6 Setting Short-Term Goals In Preparation For Stopping For Good

If the participant is committed to stopping smoking but reluctant to set a quit date, consider asking him/her to set a short-term goal, e.g., quitting for 24 or 48 hours (if currently smoking daily), or going a weekend without smoking, even at parties (if a social or infrequent smoker). Short-term goals can help move the participant to set a stop date. These steps may be regarded as "successive approximations" leading to stopping smoking. The counselor's goal is to support progress and help the participant maintain focus.

> *Tip:* Participants may have more confidence in their own ability to stop smoking, and have more confidence in their quit strategies, if they "try them out first." Encourage the participant to try some of the strategies in his/her plan before your next call, just to try them out. With a little practice, the new strategy can become as second nature as smoking. *"Why don't you just try out listening to music instead of smoking a cigarette when you're feeling stressed at home? That way you can see how it works for you before you quit."*

8.3.7 Set a Stop Date

When the participant feels that all questions have been answered and that a workable plan has been developed, then he/she and the counselor should agree on a date to implement the plan; this is called the "stop date." (*"How would you feel about setting a date to stop smoking?"*) If the participant included anything in his/her action plan that is best done ahead of the stop date, e.g., cleaning his/her car or room, getting rid of cigarettes, the counselor will ask the participant about the best time for him/her to do these tasks (for example, the day before the stop date) to prompt the participant to include these steps in his/her planning.

The counselor then schedules an appointment to call back for cessation support on [preferred] or immediately after the stop date. (From that date forward, the counselor will schedule calls in accordance with cessation support call schedule.) The participant's expectation of the Stop Day call provides a degree of accountability that may help to overcome any ambivalence he/she may feel about following through with stopping. It also lets the participant know that the counselor will be with him/her during this big step. By setting a stop date, the participant transitions with the counselor from Motivation Enhancement to Cessation Support.

Record the Stop Date. [Process data]

8.4 Wrap-up the Call

When wrapping up a call to a participant in preparation, the counselor will do the following:

(1) Summarize the discussion using "you" language. The counselor will use reflective statements to emphasize concerns, goals and specific plans for stopping smoking that were voiced by the TP. The counselor will offer encouragement and support for these as appropriate.

(2) Explore any actions taken in the call and discuss any "next steps" (e.g., cleaning room, getting rid of cigarettes) to be taken before the next call.

(3) Offer to send a brief summary of his/her action plan, along with some self-help materials ("ButtsOut #2") and a free "Quit Kit" (with helpful aids for stopping smoking such as gum, squeeze ball, water bottle, etc.), to the address of his/her choice.

(4) As appropriate, refer the participant to specific pages of the Web site, or to specific portions of self-help materials.

(5) Particularly when talking to precontemplative and contemplative smokers, it is important for the counselor to always leave the door open for further contact, without pressuring the participant to stop smoking.

(6) Confirm that the counselor will be calling on the TP's Stop Day (the counselor will set date and time for call appointment) to check in and see how things are going. When making the next call appointment, the counselor will prompt the participant to think about any work, sports, or rehearsal schedules; this might help increase the likelihood of setting a time for an appointment that will be met by the participant.

9. CESSATION SUPPORT: RELAPSE PREVENTION

If, in the course of the initial call or the M-E calls, it is established that the participant is ready to stop smoking on a specified stop date, or has already taken action to stop smoking, the counselor's focus shifts from motivation to cessation support, i.e., emphasizing implementation of coping strategies and skills for stopping smoking and relapse prevention. Thus, the counselor's primary therapeutic modality shifts from MI to cognitive behavioral therapy. The counselor will continue to use MI as a communication style in order to support the counselor-participant relationship and reinforce participants' ownership of their decision to stop and, as needed, will fall back on MI strategies when motivational issues arise.

Sixty percent of smokers relapse within the first week of quitting [Zhu & Pierce, 1995]. Cravings and withdrawal symptoms are typically most intense in the two weeks following the last cigarette; thereafter, the cravings are weaker, and withdrawal symptoms are mild or even non-existent. This schedule may be helpful not only to individuals who are nicotine dependent, but also for those whose smoking has become a behavioral habit, a social ritual, or provides psychological relief.

To support participants during the crucial period immediately following their stopping smoking and beyond, on the participant's stop date he/she will begin an intensive, relapse-sensitive schedule of telephone counseling. These Cessation Support calls are aimed at supporting the participant's efforts to remain abstinent and prevent relapse. Following this schedule, participants may receive *up to six* cessation support calls, along with follow-up counselor mailings, **scheduled over 60 days beginning on their stop date and scheduled for Days 0, 3, 7, 14, 30, and 60.** This relapse-sensitive schedule is designed to support participants during the period in which [physical and psychological] withdrawal symptoms and cravings are strongest, and the probability of relapse is greatest, thus optimizing the effect of counseling.

Calls may be adjusted to the participant's schedule as needed – for example, participants with high risk situations coming up, e.g., weekend parties, may want a call just before that event, or participants may miss appointments.

If they wish, TPs with e-mail will also have the option to also receive messages of encouragement and support on Days 1, 2, 5, 10, 12, and 20.

In every support session, the counselor encourages the participant to report the extent to which he/she has used the action plan, evaluate its effectiveness, and modify the plan accordingly, taking into account any new situations or obstacles coming up. By leading the participant through several repetitions of this cycle, the counselor provides the training necessary for the participant to continue the process independently, after the counseling has ended. With time, most of the situations in which it was initially difficult to refrain from smoking become easier to manage due to the waning of withdrawal symptoms, and because the participant has learned to refrain from smoking in these situations. **The counselor will ensure that the participant recognizes these successes and attributes them to his/her own actions.**

Cessation Support (Relapse Prevention)
Call Elements

- Introduction
- Build interest and rapport; Encourage future calls
- Revisit Consent / Privacy
- Seek Personal Opinions and Insights

(If Cessation Support is transition from Introduction Call)

- Assess unique situation
- Assess Quit Status/Progress
- Discuss & Normalize Withdrawal Symptoms
- Evaluate Coping Strategies
- Examine Slip/Relapse Situations
- Revise Action Plan as Needed
- Revisit Self-Efficacy, Motivation, Confidence, Support, Benefits/Costs, Self-image as Needed
- Wrap-up the Call

9.1 Introduction

Counselors will identify themselves on all follow-up CS calls. It's helpful to greet the participant by name and, to get the ball rolling and re-establish rapport, remind him/her of something you learned from the last call, or something related to the participant's attempt to stop smoking.

9.2 Build Interest and Rapport; Encourage Future Calls

In <u>every</u> call to participants, establishing and maintaining participant interest and counselor/participant rapport with the goal to maintain contact and keep the participants involved with the intervention is paramount. Express acceptance; don't pressure. Provide encouragement as appropriate. Build motivation for accepting subsequent calls. (See section 5.)

9.3 Revisit Consent / Privacy

Each time a participant is called, counselors must remind him/her that the call may be monitored or recorded. Example:

> *"My supervisor may monitor or record this call to make sure that I'm doing my job well. Is that okay with you?"*

Do not record a call if the participant indicates he/she does not wish to be recorded, and record in the TC Notes that participant asked not to be recorded.

Also, with each call, take a moment to be sure that this is a good time for the participant to talk and that he/she has enough privacy to participate in the call. Example:

> *"Is this a good time and place for you to talk to me on the phone?"*

Let the participant decide what is enough privacy. If this is not a good time/place for the call, make suggestions for enhancing privacy, or make an appointment to call the participant at another time/place or on another phone line.

9.4 Seek Personal Opinions and Insights

Continue to take advantage of natural opportunities that arise during conversations to learn participants' frank and personal opinions and insights about smoking and non-smoking, as well as details of their unique situations with regard to smoking. Doing so helps maintain rapport and provides counselors with tailoring the intervention to the individual. See section 5.

(If Cessation Support is transition from Introduction Call proceed with 9.5.)

9.5 Assess the Participant's Unique Smoking Situation

As we learn more about the participant, we'll want to also know more about the role smoking plays in his/her life. Assessing the participant's unique situation allows counselors to identify how to best proceed with the smoker intervention telephone calls; it provides useful information that the counselor can use when tailoring the intervention to the individual participant. The assessment also helps the counselor demonstrate that he/she wants to know what smoking means to the participant, it provides a common understanding shared by the counselor and the participant, both of which help build rapport, and it gets the participant to start thinking reflectively about his/her smoking.

Assess the smoker's unique situation

Strategies:	Examples:
For participants who've taken action to stop, modify your usual assessment statements accordingly.	*"I'd like to understand better how smoking fit in to your life. Can you describe a typical situation when you were smoking?"*<br>Or<br>*"Before we talk about your decision to stop, I'd like to understand better how smoking fit into your life. Can you describe a typical situation when you smoked?"*<br>Or<br>*"Let's spend the next few minutes going through a typical day for you.* [As participant brings up life situations, demonstrate interest.] *"So, you have to be at school by 7:30 a.m. for marching band practice. That's early! What instrument do you play?"* [As the opportunity arises, bring up smoking.] *"So, you're a trumpet player. Did your smoking ever impact your music, like affect how well you blow your horn?"*
For **infrequent smokers**, consider asking about specific situations when they may smoke, e.g., weekends or parties with friends.	*"Sounds like you only smoked once in a while, like when you were at parties. Tell me more about situations like that."*

Strategies:	Examples:
Asking about a typical day provides a natural opportunity to follow-up with questions about level of smoking: Process data item	*"Sounds like from your description that you were smoking (every day/weekly/monthly). Is that right? . . . About how many cigarettes would you say you smoked each (day/week/month)?"*
(If participant is in preparation stage) Explore the participant's motivations for stopping smoking.	*"Now, you mentioned that you recently quit. What led you to that decision?"*
Assess importance of stopping smoking. Record the answer<br>Process data item<br><br>Note: If value placed on importance is low (e.g., quitting because boy-/girlfriend wants him/her to), work on building importance for stopping.	*"It sounds like stopping smoking might be pretty important to you. Can you give me a feel about how important? Right now, today, how important is it to you personally to stop? If "0" was not important and "10" was very important, what number would you give yourself?"*<br><br>0 - 1 - 2 - 3 - 4 - 5 - 6 - 7 - 8 - 9 - 10
Assess the participant's confidence in his/her ability to stop smoking. Record the answer.<br>Process data item<br><br>Note: If confidence is low, work on building confidence.	*"So, you've decided to stop smoking. How confident are you that you will succeed this time? If "0" is not at all confident and "10" is very confident, what number would you give yourself?"*<br><br>0 - 1 - 2 - 3 - 4 - 5 - 6 - 7 - 8 - 9 - 10
Assess the participant's current dependence on nicotine. Record the answer.<br><br>Process data item	*"When you were smoking, on a typical day, how soon after waking up did you have the urge to smoke your first cigarette?"*<br>a. As soon as I wake up<br>b. Within about ½ hour of waking<br>c. An hour or two after I wake up<br>d. Over 2 hours after waking

9.6 Assess Quit Status / Progress

Assess the participant's progress with staying smoke-free. If this is a Stop Day call, confirm (using a conversational style) whether the participant stopped smoking as planned, and record the date in the TI Master form. [Process data] Example:

"Hey, today was your big day. How'd it go?" "What's it been like for you today. Did anything about today surprise you?" "So, how are you feeling about stopping, now that your first day of not smoking is almost over?"

If participant failed to stop as planned, find out why (e.g., didn't receive Quit Kit, major life event, other problem). Example:

"Gosh, I thought you were all set. What happened?"
"How are you feeling about the fact that you're still smoking?"

If this is a post-Stop-Date-Call, find out what's happened since the last call. Example:

"How's it going? What's been happening with you since the last time we spoke?"

9.7 Discuss and Normalize Withdrawal Symptoms

Use open-ended questions and record symptoms on the checklist. Help the participant understand the physiological processes behind the symptoms, and how long each might be expected to last. Provide positive reinforcement for the participant having withstood the symptoms. This feedback helps him/her understand that the discomfort of withdrawal is normal, harmless, and passing, and it helps improve self-confidence.

Strategy	*Examples*:
Discuss and normalize withdrawal symptoms.<br><br>R**ecord all withdrawal symptoms reported by the participant on the TI Call Form.**.	*"How have you been feeling, physically and emotionally since you quit? Have you noticed anything different?"* [Process Data]<br>*"Do you have any concerns you'd like to discuss?"*<br>*"You know, these are signs your body is recovering, and they'll go away in a few more days/weeks."*

Potential withdrawal symptoms participants may experience are listed below.

> *Tip:* **Do not ask participants specifically about each symptom; ask open-ended questions and let the participant tell you what he/she is experiencing.**

- ☐ Coughing, throat/nasal problems
- ☐ Craving
- ☐ Depression / Mood swings
- ☐ Dizziness
- ☐ Fatigue
- ☐ GI symptoms
- ☐ Headaches
- ☐ Hunger
- ☐ Insomnia
- ☐ Irritability
- ☐ Inability to concentrate
- ☐ Nervousness / Anxiety
- ☐ Shaking
- ☐ Sweating
- ☐ Other ______________________
- ☐ Other ______________________
- ☐ No withdrawal & on pharmacoRx
- ☐ No withdrawal (no pharmacoRx)

9.8 Evaluate the Effectiveness of Coping Strategies

Find out how the participant's planned coping strategies for handling challenging situations have worked. Discuss both coping strategies for external circumstances (triggers to smoke) and for physical withdrawal symptoms, as needed.

**Strategy**	***Examples***
Evaluate the effectiveness of coping strategies for external circumstances (triggers to smoke). If needed, also evaluate effectiveness of strategies for handling physical withdrawal symptoms.	*"Let's talk about difficult situations you've been in, and how you've handled them." "How did [strategy] work for you?" " Is there anything you think you'd do differently in the future?"*
Address refusal skills as needed.	*"Sounds like you have a tough time staying quit around your friends. What do you think would make that easier?"*
Support internal strengths and resources that have helped the participant to be successful.	*"Wow, I can see that once you put your mind to something, you follow through. That's great. What besides your persistence has helped you be so successful?"*

9.9 Examine Slip/Relapse Situations

Without conveying disapproval, the counselor discusses the lapse with the participant, with a goal to ascertaining whether the lapse was due to the difficulty of the situation or failure to use a planned strategy, an omission that can be remedied the next time the situation arises. An important goal of the counselor is to help normalize the slip, and to point out that the quit attempt is not ruined by a slip and that failure is not the inevitable outcome. The participant who has slipped has a unique opportunity for success by refusing to give up on quitting! [Process data items]

Strategies	*Examples*
Examine slip or relapse situations (if necessary). If the participant slipped, find out about the slip in a conversational, nonjudgmental way.<br><br>If no slips, proceed.	*"Have you smoked at all – even a puff – since you quit?"*<br><br>[if yes] *"What happened – what was going on when you had that cigarette?"*<br>*"How did you feel about it?"*<br>*"When did you smoke that first cigarette?"*<br>*"How many cigarettes did you smoke that day?" "Have you smoked at all since then? . . . How much?"*
If the participant slipped, support internal & stable strengths that got him/her back on track.	*"So you just smoked a few cigarettes at that one party but haven't smoked in the last two days. That's great. Why do you think you've been able to get back on track with quitting?"*

Strategies	*Examples*
Talking about slips will normalize them. It helps the participant know he/she will face challenges, and to know that slipping up and having one cigarette does not mean he/she has to go back to smoking. It is not a failure, just a slip. Some participants, particularly those who've quit and failed before may be fearful of slips.<br><br>Present an optimistic view of slips to the participant; emphasize that he/she is in control.	*"How did you feel when you slipped up and smoked that cigarette?" "What would you do differently next time?"*<br><br>*"Would you like to hear what's worked for other people?"*<br><br>*"You know, slips are an expected part of new learning, to help you know more about your needs and how to stay quit. For example, now you know that under certain circumstances, this particular situation (e.g., drinking at a party) can be a danger to you. So, what can you do next time you're in a similar situation but don't want to smoke?"*
If the participant relapsed, attribute behavior to lack of planning rather than a deficiency in internal resources. Address abstinence violation effect as needed.	*"Well, it looks like the plan was not what it needed to be to help you in this situation. What do you think you could include in your plan to help you cope next time you're in this situation?"*

9.10 Revise Action Plan as Needed

The counselor asks the participant to think of any new situations or challenges that may arise before the next follow-up session, and helps brainstorm coping strategies with the participant.

**Strategies**	***Examples***
Find out about high-risk situations coming up in the near future that the participant may be concerned about. Help him/her brainstorm coping strategies for those.	*"Is there anything difficult coming up in the next few days that you want to plan for?"*<br><br>*"What do you think you could do in that situation, to feel good and still not smoke?"*

9.11 Revisit self-efficacy, motivation, confidence, social support, benefits/costs, self-image

During cessation support calls, revisit these issues as needed in response to feedback from the participant, to boost participant's self-esteem and resolve any lingering ambivalence about quitting.

Strategies	*Examples*
Revisit / boost motivation as needed	*"I can understand that you feel pretty discouraged right now; you tried to stop but now you're smoking again. It's tough when you try something but don't get as far as you wanted to. But don't overlook the fact that you did succeed for XX days – that's a great accomplishment. What do you think worked for you on those days?"*<br><br>*"Are you still thinking about your reasons for quitting?"*<br><br>*"Some people find that their motivation goes up and down . . . is that true for you? How do you experience that?"*
Revisit issues of social support, as needed. If participant does not have a social support network for quitting, explore how he/she rewards him-/herself for quitting.	*"So, who's been supportive? . . . What kind of support have they given you (e.g., emotional, practical)?"*<br><br>"Do you feel like you're getting the support you need?"<br><br>"Has anyone surprised you with support?"<br><br>**If no support:** *"How have you been taking care of yourself?"*
Explore and boost confidence, self-efficacy.	*"Why do you think you've been successful so far?"*<br><br>*"What strengths do you see in yourself?"*<br><br>*"How do you feel about what you've done?"*<br><br>*"On a scale of 0-10, how confident are you that you can go another month without smoking?"*
Revisit benefits and costs of quitting, as needed. Work to focus participant slightly more on the benefits side of the equation.	*"Do you miss smoking? What kinds of things do you miss about it? Can you get those things another way?"*<br><br>*"What good things have you noticed since you quit? What else has changed for you?"*

**Strategies**	***Examples***
In post-Quit-Day calls, focus participant on developing a self-image as a nonsmoker. Help participants over any awkwardness they feel as nonsmokers by positively reinforcing their new behavior, giving reassurance that any awkward feeling will fade, and emphasizing that being smoke-free is the participant's natural state.	*"Have you begun to feel like a nonsmoker?"*<br><br>*" How do you think this is different from feeling like a smoker who is currently not smoking?"*<br><br>*"How do you feel about accomplishing something – quitting smoking – that not many people your age have been able to do?"* *"Remember, you were a nonsmoker once before in your life."*

9.12 Wrap-up the Call

When wrapping up a cessation support call to a participant, the counselor will do the following:

(1) Summarize the discussion using "you" language. The counselor will use reflective statements to emphasize goals and specific plans voiced by the participant. The counselor will end with supportive, positive comments. *"How are you feeling now?"*

(2) For participants doing well following their cessation plans, the counselor will briefly review any "next steps" to be taken before the next session. For a participant who has experienced a slip or relapse situation, the counselor will review the participant's revised cessation plan or other planned next steps. With these participants, the counselor will emphasize what has been learned from the slip experience, and the time that the TP successfully remained quit.

(3) As appropriate, the counselor will refer the participant to sections of "ButtsOut" and/or the web site.

(4) Ask TP if we may send a brief summary of what the TP talked with his/her counselor about today.

(5) **Set date and time for next appointment.** When making the next call appointment, the counselor will prompt the participant to think about any work, sports, or rehearsal schedules; this might help increase the likelihood of setting a time for an appointment that will be met by the participant. Also, the counselor will ask about any upcoming challenging situations that might influence the timing of your next call.

(6) For participants who relapsed, the counselor will note their new quit date, or date set for other new goal: [Process data]

(7) **For FINAL CALL:** The counselor will reflect with participant on the enormous progress he/she has made. Examples: *"Let's look back on what you have accomplished!" "What advice would you give to a friend who was quitting?" "What do you like most about having quit?" "What will you remember in order to stay quit for good?" "What have you learned about yourself?"*)

9.13 Non-attempts

Despite their best intentions and the accountability engendered in the counseling relationship, some participants who set stop dates will not attempt to stop on their planned date. When this occurs, the counselor will help the participant examine his/her reasons for the change of plans. The participant may have encountered (1) unforeseen difficulties, (2) waning of his/her motivation, or (3) increasing ambivalence about making difficult behavioral changes. In such situations, the counselor will try to explore reasons for the non-attempt, and normalize ambivalence or concerns (e.g., *"What you're feeling is not at all unusual. It's really quite common, especially in these early stages of quitting. After all, you're thinking about changing a pattern that has developed over several years. Give yourself some time."*). Counselors will also reinforce any self-motivational statements and indications of willingness to quit, and reassure the participant that other participants have been successful in quitting smoking. Rapport may be fragile in these cases, therefore, the counselor must be careful to be nonjudgmental when the participant has not made his/her quit attempt.

The participant will be given the opportunity to set a new stop date and the counselor will help to refine strategy, boost motivations, and overcome the ambivalence about implementing the plan. A new stop date and appointment is set, and the cessation support call schedule starts over.

If a participant lets a second stop date pass without making an attempt, the counselor again assesses the reasons and helps the participant understand the obstacles to stopping smoking. To avoid risk of harming the participant's self-efficacy with a third non-attempt, the Counselor will talk to the participant about setting a more short-term, achievable goal. For example, rather than set a date to quit altogether, the counselor and participant may instead target a specific day for a 24-hour quit attempt. After going for a day without smoking, the participant's motivation and self-efficacy may be increased so that he/she will be ready to proceed with a full quit attempt.

10. INTERVENTION FOR NON-SMOKERS

As previously described, nonsmokers have a significant role in the proposed trial. Accordingly, telephone counseling, the Web site, and supplemental self-help materials for nonsmokers are designed to (1) support their smoking abstinence, and (2) enhance their motivation and skills for influencing their smoking peers to quit. Like the smoker intervention, the nonsmoker intervention incorporates aspects of Motivational Interviewing (MI) and Cognitive Behavioral Therapy (CBT): Use of MI strategies are aimed at helping motivate nonsmokers and move them toward action with regard to helping their smoking peers to quit. CBT will help nonsmokers build the skills needed to make those actions more effective.

Each nonsmoker trial participant will receive a telephone call from a counselor who will briefly reinforce the nonsmoker's abstinence, review tips for helping friends quit smoking, and motivate and coach the nonsmoker in providing feedback, support, and advice to smoking friends. The counselor will help the participant practice such strategies when appropriate. The self-help materials for nonsmokers will provide these TPs with important background information (e.g., what smoking friends may be dealing with while trying to quit) and multiple helping strategies, like those practiced with the counselor. Likewise, individual elements (e.g., posters, school newspaper ads) of the environmental component will be directed towards nonsmokers and the promotion of smoke-free norms.

By listening to these participants well, and letting them know we are listening, they may be empowered to feel good about themselves.

Participants who receive the nonsmoker intervention are those that answered "no" to the question *"Do you currently smoke cigarettes?"* <u>and</u> answered "never" or "more than 3 months ago" to the question *"When did you smoke your last cigarette?"* *Exception:* The participant who was a smoker at baseline but at initial contact says he/she has never smoked and hasn't smoked in the past 3 months.

Nonsmoker Call Elements

(Reminder: these calls will have transitioned from Initial Call)

- Revisit Consent / Privacy
- Assess Unique Situation
- Support the Nonsmoker's Choice to be Smoke-free
- Enhance Motivation to Help Smoking Peers to Stop
- Develop Skills for Helping Smoking Peers to Stop
- Wrap-up the Call

10.1 Revisit Consent / Privacy

If the counselor is doing a second nonsmoker call, or has to call the participant a second time to complete the non-smoker intervention (e.g., the participant didn't have time during the introductory call):

Counselors must remind participants that the call may be monitored or recorded. Example:

> *"My supervisor may monitor or record this call to make sure that I'm doing my job well. Is that okay with you?"*

Do not record a call if the participant indicates he/she does not wish to be recorded, and record in the TC Notes that participant asked not to be recorded**.**

Also, with each call, take a moment to be sure that this is a good time for the participant to talk and that he/she has enough privacy to participate in the call. Example:

> *"Is this a good time and place for you to talk to me on the phone?"*

Let the participant decide what is enough privacy. If this is not a good time/place for the call, make suggestions for enhancing privacy, or make an appointment to call the participant at another time/place or on another phone line.

(If Cessation Support is transition from Introduction Call proceed with 10.2)

10.2 Assess Unique Situation

Strategies:	***Examples:***
Many of the nonsmokers in the study may be former smokers. Ask more detailed questions about smoking history to assess the participant's unique situation.	*"How long has it been since you stopped smoking?" "What were your reasons for stopping?" "What quitting strategies were particularly helpful to you?"*
If participant is a never smoker, show interest in that choice, too.	*"I'm interested in knowing more about your experience as a never smoker."* (pause) *"What has that been like for you? What helped you make that choice?"*

10.3 Support the Participant's Choice to be Smoke-free

Opportunities to support the non-smoker's choice to be smoke-free may naturally occur in your discussion of the TP's experience as a non-smoker, as you assess his/her unique situation.

Strategies:	***Examples:***
Acknowledge the participant's choice not to smoke; if the participant relates that he/she used to smoke, acknowledge what a significant accomplishment quitting is. Show interest in this accomplishment.	*"You've made the choice not to smoke. That's really great. What helped you make that choice?"* (if participant relates that he/she used to smoke, continue) *"Wow – congratulations on quitting smoking! That's a major accomplishment. What kind of changes have you noticed since you quit?"*

10.4 Enhance Motivation to Help Smoking Peers to Stop

Despite being selected based on their survey responses indicating a desire to help others to stop smoking, assume less interest and motivation than more. Use MI strategies to get the nonsmoker trial participants more interested in actually taking helping action.

Strategies:	***Examples:***
Use the participant's success in becoming/remaining a nonsmoker to <u>enhance his/her motivation to help smoking peers to quit</u>. Emphasize the importance of social support to smokers who are trying to quit.	*"A lot of people find it easier to quit smoking when they have support from friends. Was that your experience? . . ."*<br><br>*"Based on your experience, it sounds like you have a lot to offer your friends who smoke and want to stop. Is that something you'd be interested in doing?"*<br><br>*"How do you think you could be most helpful? When you were quitting, what kind of help would you have liked?"*
Many participants feel uncomfortable with the idea of offering help to a friend; they feel it's obtrusive. Their belief that smoking is a personal choice is so strongly held that it precludes offering assistance.<br><br>Try using this argument as a reason to help. Remind the participant how much he/she has to offer.	*"For your friends, smoking is a personal choice, and so is quitting. But that doesn't mean that you can't help. In fact, some may welcome your help."*<br><br>*"Other students tell us that when they're ready to stop, they want their friends to help. Many don't think the adults in their lives can help them with stopping, but their friends can."*<br><br>*"Sometimes it's hard to talk to friends who smoke about stopping. You don't want to interfere with their right to choose for themselves. Some of your friends might not be interested in stopping now. But when the time is right, you can help a lot."*<br><br>*"Some people, when they want to stop, they don't even try because they don't think they can or they don't know how. With your experience, you can show them first hand that if stopping is what they want, they can do it!"*

10.5 Develop Skills for Helping Smoking Peers to Stop

Strategies:	***Examples:***
Help nonsmoker develop skills for helping smoking peers to quit. Brainstorm tips for helping others. Follow-up on these and solicit additional ideas. Emphasize that when brainstorming, there are no bad ideas. Offer to practice, or role play, some of these strategies over the phone. (If teen has no ideas, or has suggested ideas that may not be helpful, ask permission to provide some helping tips. Use the MI strategy of "agreement with a twist" to turn a less fruitful idea into a more constructive one.)	*"Let's talk about things you might do to help others to quit – we can brainstorm about things that might help. What ideas come to mind?"*<br><br>*"What worked for you when you were trying to quit?"*<br>Constructive ideas:<br>*DO express your help in terms of your own concern for your friend (e.g., "I'm worried about . . .")*<br><br>*DO acknowledge that your friend may get something he/she likes from smoking, and may find it difficult to quit.*<br><br>*DO express your confidence in your friend's ability to quit for good; be encouraging. ("I know quitting is tough, but so are you. You can do this if you really put your mind to it. And I'll help any way I can.")*<br><br>*DO suggest specific action. For example, if your friend always smokes at parties, he/she can try holding something to eat or drink in the hand he/she usually uses to hold a cigarette. Or, help your friend make a list of all the places and times when he/she doesn't smoke. Point out, first, what a long list it is! Then, try to pick out things to do, places to go from the list where your friend won't smoke. For example, go shoot hoops or roller-blading in the park.*<br><br>*DON'T criticize, nag, or bring up past failures. Now is the time to be positive! "Catch" your friend not smoking and congratulate him/her.*
Another option is to teach the participant some easy MI strategies to use when talking to friends who smoke:<br>(1) Express care for the participant and concern regarding continuing smoking<br>(2) Express respect and acknowledgment of the participant's independence, autonomy (i.e., right to make his/her own decision)<br>(3) Make an offer of further help.	*"You're my friend; you know how I feel about you. And you know that I worry that you may be smoking too much. But it's not my decision to make. It's up to you. But, if you ever do decide that stopping smoking is something you want to do, I'm here to help you in any way I can."*

10.6 Wrap-up the Call

When wrapping up a call to a nonsmoking participant, the counselor will do the following:

(1) Summarize the discussion using "you" language. The counselor will use reflective statements to emphasize goals and specific plans for helping smoking peers to stop that were voiced by the TP. The counselor will offer encouragement and support for these as appropriate.

(2) Ask TP if we may send a brief summary of what the TP/counselor discussed today, along with some print materials that provide "helping tips."

(3) If TP thinks a second call would be helpful, the counselor will schedule a second call. Example:

> *"It's completely up to you whether you'd like another call from me. These calls are meant as a resource for you. [Some students who want to help a friend or loved one quit have found a second call helpful.] Would that be helpful to you?"*

11. PROTECTION OF HUMAN SUBJECTS

11.1 Overview: Human Subjects Protection and Informed Consent

Dramatic advances in prevention and treatment of disease have been achieved through research carried out by universities, the private sector and the government. A crucial part of this research involves the voluntary participation of human subjects in clinical trials to test new therapies. Federal policy has sought to preserve the benefits of this research by establishing detailed regulations and guidelines for protecting research participants from any abuse or harm. In particular, a regulation implemented by 17 federal agencies, known as the Common Rule, seeks to guarantee review of research for projects and assure willing consent, including a proper understanding of risks involved, for those participating in clinical trials.

As required by federal regulation and FHCRC policy, the FHCRC has established an Institutional Review Board (IRB). The IRB is responsible for reviewing all Center research activities involving human subjects to ensure that ethical standards for the care and protection of human subjects have been established and are in compliance with all pertinent (federal, state, and local) regulations and with FHCRC policy. The IRB approves research based on their determination that the following requirements have been satisfied:

(1) Risks to subjects are minimized by using procedures consistent with sound research design and which do not unnecessarily expose subjects to risk.
(2) Risks to subjects are reasonable in relation to anticipated benefits, if any, to subjects, and the importance of knowledge that may reasonably be expected to result.
(3) Selection of subjects is equitable.
(4) Informed consent will be sought from each prospective subject, or the subject's legally authorized representative. [The "informed consent" process requires that potential participants be given an explanation of purposes of the research, the expected duration of the subject's participation, a description of the procedures to be followed and their potential risks and benefits, and identification of any procedures that are experimental.]
(5) Informed consent will be appropriately documented.
(6) Where appropriate, the research plan makes adequate provision for monitoring the data collected to ensure the safety of subjects.
(7) Where appropriate, there are adequate provisions to protect the privacy of subjects and to maintain the confidentiality of the data.

The FHCRC IRB approved the HS Study on March 1, 2001. Subsequently, the IRB has reviewed and approved modifications to specific procedures and documents. The HS Study will undergo annual IRB review.

11.2 Training in Human Subjects Protection

Training in the principals for the ethical conduct of human subjects research is required for all key personnel of studies awarded to or sponsored through the Fred Hutchinson Cancer Research Center. "Key personnel" includes all persons – faculty and staff – responsible for the design and conduct of research involving human subjects. Funded NIH awards specifically require principal investigators to confirm that all key personnel listed in the grant have completed the training. FHCRC has extended this requirement to ALL human subjects research, regardless of funding source.

FHCRC recognizes either completion of the web-based human subject ethics/education training program or attendance at an in-person training session offered by the University of Washington or FHCRC.

All HS Study counselors are required to complete human subjects training prior to beginning calls to trial participants.

11.3 Potential Benefits of Participation in Matchbreaker Telephone Counseling

The greatest potential direct benefits of participation in the study's telephone counseling intervention are the tremendous long-term and short-term health benefits to participants who stop smoking or to non-smokers who remain smoke-free. These include a greatly reduced risk of developing lung cancer and other cancers, heart disease, stroke, and high-blood pressure, and freedom from nicotine addiction.

There are many other potential benefits to participants who take part in the intervention. For example, participants may appreciate the opportunity to talk to an impartial, caring adult on the phone (instead of face-to-face) about their smoking and interest in stopping. Participants interested in stopping smoking will receive assistance with stopping. Participants who stop smoking may save money otherwise spent on cigarettes. In addition to improved health, participants who stop smoking may realize improved self-esteem, self-image, and/or self-confidence. They also greatly reduce the risk that their own [future] children will smoke. Helping friends or family who smoke to stop may be a source of personal satisfaction to participants. Friends or relatives of participants may stop smoking and experience health benefits. Parents may appreciate someone helping their teen who smokes to stop.

11.4 Potential Risks of Participation in Matchbreaker Telephone Counseling

Smoking cessation is a difficult task that may create psychological and physical stress and discomfort, especially during its early phases. Although many smokers aged 17-19 are not physically dependent upon nicotine, those that are will experience at least mild physical withdrawal symptoms during the first few days of quitting. These withdrawal symptoms can include headaches, GI upset, loss of concentration, nervousness, insomnia, irritability, and moodiness. People also frequently use smoking as a way to cope with psychological concerns such as stress, anxiety, depression, anger, and body image issues. Smoking cessation may exacerbate these symptoms and necessitate that participants develop alternative coping strategies. Additionally, participants in the research study may experience increased conflict with smokers in either their peer groups or families. Research involvement

may also arouse a parent's suspicion that his/her teen is a smoker. In rare instances, a participant's confidentiality may be violated if a parent overhears or eavesdrops upon conversations between the participant and Hutchinson Staff during an intervention call. At the onset of calls participants will be asked if they are in a private place and will be advised about measures they can take to avoid having others eavesdrop on their conversation.

11.5 HS Study Informed Consent Procedures

A multi-step process is used to obtain parental (of minor children) and participant consent for participation in the Matchbreaker Telephone Counseling Intervention.

11.5.1 Goals of Informed Consent Process

Consent procedures for the intervention were developed to meet the following criteria:

(1) Honor the rights of parents and the study cohort member.
(2) Be sensitive to the needs and interest of parents, students, and schools.
(3) Provide an accurate description of the intervention.
(4) Use procedures designed to maximize participation (a) by parents in the active consent process and (b) by study cohort members in the intervention.
(5) Develop rapport with parents, motivating them to cooperate with the study, and in particular, to want their study cohort member to participate in the intervention.
(6) In all consent communications (letters and telephone scripts), use simple, clear language that is easy to read and understand, and that is clean, pleasant looking, and simple to complete and send back.
(7) Make the agreement to participation as easy as possible to understand, to fill out, and to do.

11.5.2 Parental Consent Procedure

The HS Study seeks active parental consent via mail, with mail and telephone follow-up, as needed, of non-responders. All correspondence with parents is designed to be informative, friendly, conversational. (Parent active consent letters, the Reply Card, the parent Frequently Asked Questions brochure, and the non-responder telephone script may be found in Appendix 1.)

Documentation of active parental consent among those parents who provide consent via the mail are be the parent's indication and signature on the Reply Card. Documentation of active parental consent among those parents contacted via a non-responder telephone call are paper and database records of the date and time of the consent, the name of the person providing the consent, and a summary of the consent conversation.

Active parental consent is required for those students under 18 years of age. An exception is those study participants under age 18 identified by their schools as "emancipated minors." Because 18 year olds are considered adults in the state of

Washington, parental consent is not technically required. However, as a courtesy to parents, the HS Study mails an informational letter to parents of 18 year old students in the active consent procedure, to let them know that we will be contacting their 18-yr-old to invite him/her to participate in the Matchbreaker program. Likewise, for the parents of potential participants who turn 18 after their parents have received the request for active parental consent (but who failed to reply after 30 days), we mail a modified informational letter.

11.5.3 Student Consent Procedure

The HS Study mails an informational letter and a student Frequently Asked Questions brochure (Appendix 1) about the telephone counseling activity to minor students whose parents have consented, and to students who are age 18 or older. The first counseling call occurs approximately 2-3 weeks after the informational letter mailing. In the first call, the counselor mentions the letter, offers to summarize or resend the letter if student didn't receive it, reminds the student that the counseling conversations are confidential and that participation is voluntary, and asks for the student's consent to participate (see section 6.2). If any student wants time to think about it, the counselor will ask for a good time to call back.

11.6 Confidentiality

As per FHCRC Personnel Policies and Procedures, section 8-5, preserving confidential information, including but not limited to medical information of patients or information relating to confidential Center-related work, is the responsibility of all employees. In accordance with the FHCRC policy, counselors must protect the privacy of trial participants and ensure that there will be no unauthorized disclosures of information given in confidence. Specifically, counselors will:

(1) not disclose outside the Project any identifiable information communicated to them from participants.

(2) share information about participants only within the Project and only for professional purposes, even when the information does not include identifying information.

(3) not use identifying information when consulting about participants with other project counselors unless there is a clear and imminent danger to the participant or others, as defined in section 12.2.

(4) safeguard all materials with identifying information, such as lists, letters, and files. Identifiable materials must never be left out on desks or taken out of the office. Within the project, materials with identifying information must be stored, when not in use, in locked file drawers in locked offices. Also, counselors will shred all confidential materials before disposing of them. All identifiable computerized records will be kept secure and password-protected, as per study security and confidentiality procedures.

11.6.1 Limits of Confidentiality

As described to participants as part of the informed consent process, all information obtained from participants can be considered confidential except (1) where there is clear and imminent danger to the client or someone else, or (2) in the case of a reportable offense as stipulated by Washington State law. Specifically, Matchbreaker staff are legally and ethically obligated to breach confidentiality in the following situations, as stipulated by the Revised Code of Washington State (RCW) 18.19.180 (Confidential Communications); 26.44.030 (abuse of children and adult dependent or developmentally disabled); 71.05.150 (Mental Illness); and 74.34.030 (abuse of vulnerable adults):

- The participant is at imminent risk to suicide.
- The participant presents an imminent threat of homicide to identified person.
- The participant is gravely disabled: A mental disorder renders an individual incapable of maintaining personal safety or providing for basic human needs.
- The participant makes a first person report of child abuse.
- The participant makes a first person report of elder abuse.
- The participant makes a first person report of abuse of a developmentally disabled individual.

12. RESPONSE TO IMMINENT AND NON-IMMINENT RISK SITUATIONS

12.1 Encountering Participants with Psychological, Mental Health, or Other Problems

As providers of a targeted public health smoking cessation research intervention, counselors should generally avoid addressing a mental health issue unless a participant reports imminent risk of harm to self (e.g., suicide; child abuse, gravely disabled) or others (e.g., homicide, child abuse, elder abuse, abuse of a developmentally disabled individual).

If the mental health problem is relatively minor, e.g., academic or other school problems, emotional upset, or family discord, the counselor should provide a short, supportive response and then tactfully attempt to direct the conversation back to smoking cessation related issues. Typically, there will be no need for additional discussion of minor issues.

If the participant persists in discussing a non-emergent psychological or mental health issue, the counselor should gently explain that he/she is not qualified to advise or counsel in this area. The counselor will then encourage the participant to seek assistance from a more qualified professional and attempt to provide the participant with an appropriate referral source.

12.2 Definition of Imminent Risk

Imminent risk is defined as the immediate likelihood of serious harm to the participant or someone else. Examples of imminent risk:

1. A participant spontaneously tells you that he has a weapon and intends to use it soon.
2. A participant spontaneously tells you she has been hoarding medication in order to make a suicide attempt. She insinuates that she might swallow the medication at any time.
3. An intoxicated participant tells you she intends to end her life and has a gun nearby.
4. A participant tells you that he has been cutting on himself and inflicting serious damage.
5. A participant tells you that she believes her father will assault her when he returns home.

12.3 Procedures for Handling Imminent Risk Situations

HS Study Counselors and supervising psychologists must be prepared to respond to participant reports of imminent risk to self or others. Although such reports are encountered rarely in a smoking cessation intervention setting, the following procedures, nonetheless, address legal and ethical obligations for taking appropriate action in response to a range of emergent situations. As a research project, our challenge is to ensure the confidentiality of participants while taking appropriate measures to protect participants' safety, adhere to legal statute, and follow clinical practice guidelines.

Figure 2, Flow of Emergency Response Actions, shows how counselors and the licensed supervising psychologist/Intervention Manager will respond to situations of imminent risk of harm to participants and others, and suggestions of imminent risk. Procedural details are documented in the sections that follow.

Figure 2: FLOW OF EMERGENCY RESPONSE ACTIONS

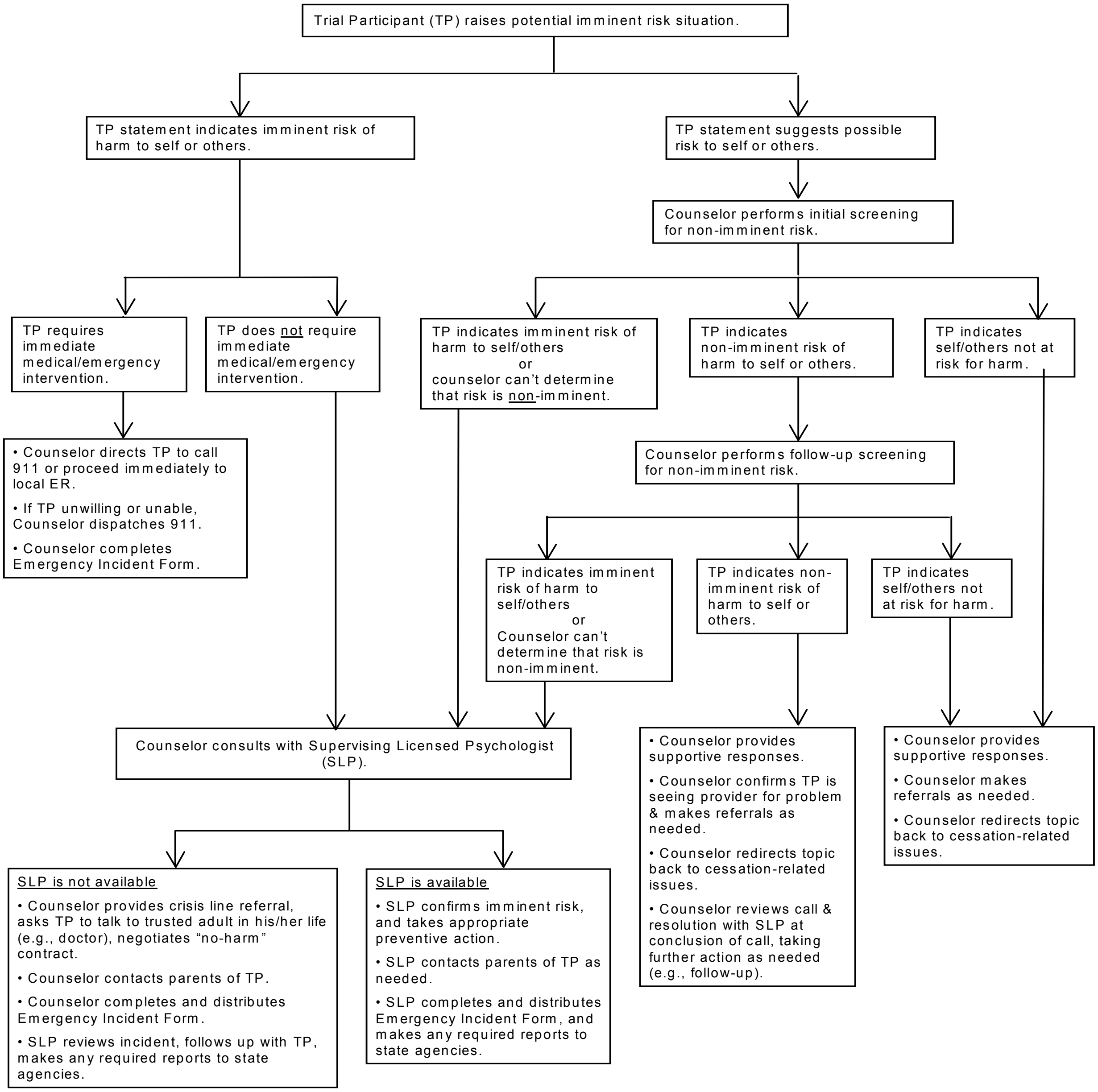

Acronyms
TP – Trial Participant (participating in telephone counseling intervention of the Hutchinson Study of High School Smoking)
SLP –Supervising Licensed Psychologist (provides clinical supervision to Study Counselors)

12.3.1 Counselor's Response to Indications of Imminent Risk Situations

In the rare circumstance where a counselor believes that a situation requires urgent assessment due to an imminent risk (that does not require emergency 911 intervention), he/she should immediately consult with the Licensed Supervising Psychologist (LSP). In order to facilitate such consultation, the counselor will elicit the help of a co-counselor as follows: While staying on the phone with the participant, the counselor will inform a co-counselor that the participant is at imminent risk. The co-counselor will then *immediately* notify the LSP, either in person or by phone/pager, while the primary counselor maintains telephone contact with the participant with the goal of having the LSP take over counseling of the imminent risk participant.

12.3.1.1 Participant requires immediate emergency intervention

If, at any point, immediate medical intervention is needed, the counselor will respond immediately by instructing the participant to call 911 or to safely proceed to the nearest emergency room. If the participant is unwilling or unable to seek immediate medical attention, the counselor will try to stay on the line and alert a co-counselor to call 911 and dispatch emergency assistance to the participant.

12.3.1.2 LSP is *NOT* available to take over the imminent risk call

If the counselor believes that a participant or another person is at imminent risk and is not able to make contact with the LSP, he/she will provide the following assistance to the participant:

- Provide a list of emergency phone numbers; offer to directly connect the participant. (See Director of On-Call and Emergency Referral Telephone Numbers.)
- Ask the participant if there is a trusted adult in his/her life (e.g., doctor, counselor) he/she can talk to and, if so, encourage him/her to do so.
- Ask the participant to "agree to no harm" until such time as he/she can check in with a responsible health care provider (physician, psychiatrist, psychologist, etc.).

12.3.2 SLP's Handling of Imminent Risk Call

If immediately available, the SLP will take over the phone call.

If out of the office and available by land or cell phone, the primary counselor will "patch" the participant through to the counselor's phone using the "conference call" feature on his/her telephone, and the LSP will take over the call.

<u>Using the Conference Call feature</u>
<u>to connect the participant to the LSP</u>

(1) With the participant on the line, push the 'Conference' button (this puts the first call on hold and you hear a dial tone).
(2) Dial 9 followed by the SLP’s telephone number, push 'Conference' (this puts the counselor in direct communication with the SLP while the participant is on hold).
(3) When the LSP answers, give him/her the TP’s phone number (in case of disconnection) and any pertinent case information.
(4) Push 'Conference' again and all three parties will be connected – the LSP, the participant, <u>and</u> the counselor are on the line together. Let the TP and the LSP know that they are connected.
(5) If the LSP wants to speak privately to the TP, push ‘Conference’ one more time. The LSP and TP will be connected and your phone will beep. You may hang up the phone, but your line will still be connected to the call until the LSP and TP end their call (you will see a blinking arrow by your phone line for as long as the LSP and TP are on the phone together). ***Another call cannot be made on this line while the connected call is still in progress.***
Notes: (a) A Hutch phone must make the call to the second, third, etc., person and (b) with our phone system, there is no way for an incoming call from the LSP to be patched in to an existing phone conversation with a participant.

When reached to handle an imminent risk call, the LSP will briefly consult counseling staff. Unless the situation is no longer emergent, the counselor will tell the participant that his/her supervisor is present and introduce the participant to the LSP as he/she joins the call. The LSP will then proceed with a formal risk assessment and disposition planning (see box below).

If the call was discontinued prior to patching through the LSP, he/she will immediately call the participant back to conduct the risk assessment interview.

Upon completion of the imminent risk call, the LSP will contact the parent(s) of the participant as needed. He/she will then complete and distribute an Emergency Incident Form, and make any and all required reports to state agencies (see section 12.5.2).

Supervising Psychologist's Formal Assessment of Imminent Risk of Homicide/Suicide/Abuse

Suicidal, homicidal and abuse risk assessment is a skilled clinical practice that relies upon the mental health professional's accurate evaluation of both acute and chronic risk factors. These risk factors include acute emotional upset, the availability of a lethal means, psychiatric and substance abuse problems, school and work difficulties, family discord, and a stated intention to harm self or others. Additionally, any antipathy by the client toward the mental health professional suggests additional risk.

The supervising psychologist will evaluate each of these factors in the course of the homicide/suicide or abuse risk assessment. If determination of imminent risk is confirmed at any point, preventative actions will be immediately undertaken. Preventative actions may include any or all of the following: supportive communication, the establishment of a no-harm contract and an agreement to seek further crisis intervention, and the disclosure of the participant's situation and state of mind to appropriate members of the community as stipulated by the Revised Code of Washington State (RCW) 26.44.30, 71.05.150 and 74.34.030. Additionally, if a participant makes a credible threat of homicide against an identifiable individual the supervising psychologist will notify legal authorities and attempt to directly warn the threatened individual as determined by the case law (Tarosof, Duty To Warn). Following such action, the LSP will complete an Emergency Incident Form (Appendix 3), documenting the incident, action(s) taken, outcome(s), and follow-up required. Copies will be forwarded to the Data Operations Manager, Sue Mann, and to the Principle Investigator, Art Peterson. Ms. Mann will notify IRB and the NIH, as required.

Safety Planning for the Imminent Threat of a Reportable Offense

If a participant discloses vulnerability to any reportable offense (RCW 26.44.30 or 74.34.030) and is imminently at risk, the LSP will immediately provide crisis intervention counseling. Such counseling is solely intended to support the immediate safety and welfare of the participant by devising a safety plan. The LSP will also complete an Emergency Incident Form, as per protocol, and make necessary contact with appropriate community resources (see Director of On-Call and Emergency Referral Telephone Numbers).

12.4 Procedures for Handling Non-Imminent Risk Situations

On rare occasions, participants may spontaneously initiate a discussion that will suggest a possible risk to self or other. Examples of spontaneous statements by participants that indicate possible danger but have not reached the threshold for imminent risk are:

1. *"I can't take it anymore…I'm going to end it all."*
2. *"Nobody cares. Everybody would be better off if I was gone."*
3. *"I'm going to get even and then they'll be sorry."*

12.4.1 Initial Screening of (non- imminent) Homicide/Suicide Risk

Following veiled or ambiguous comments of this kind, the counselor should sensitively ask an open-ended question in order to help clarify the significance of such a statement. For example, the counselor might ask, *"Can you tell me a little bit more about what you meant by that?"*

If the participant provides further information that indicates he/she is at risk of (non imminent) harm to self or others, or the counselor believes that such a risk exists, he/she should proceed as follows:

(1) Ask the TP, *"Have you had thoughts that you would be better off dead or thoughts of hurting yourself or another person in some way?"*

**If "no:"**	If "yes:"
Use reflective comments that are supportive and then steer discussion to prior cessation related topic. Participants asking for or appearing to require more support should be referred to an appropriate crisis help line	Proceed with asking question (2).

(2) *"Have these included thoughts of ending your own life or the life of another person?"*

**If "no:"**	If "yes:"
Use reflective comments that are supportive and then steer discussion to prior cessation related topic. Participants asking for or appearing to require more support should be referred to an appropriate crisis help line	Proceed with asking question (3).

(3) *"How often have you had these kinds of thoughts?*

**If "often" or "very often or provides <u>an equivalent account of frequency</u>:** Proceed immediately to section 12.4.3.	<u>If "not very often:"</u> Proceed with asking question (4).

(4) *"Have you felt really tempted to carry out such thoughts?"*

**<u>If "no</u>:"** Use reflective comments that are supportive and then steer discussion to prior cessation related topic. Participants asking for or appearing to require more support should be referred to an appropriate crisis help line	<u>If "yes:"</u> Proceed with asking question (5).

(5) *"Do you think you can keep from acting on them?"*

**<u>If "no</u>:"** Proceed immediately to section 12.4.3.	<u>If "yes</u>:" Proceed with asking question (6).

(6) *"Just how strong are your impulses to act on these feelings?"*

**<u>If "not very strong</u>:"** Use reflective comments that are supportive and then steer discussion to prior cessation related topic. Participants asking for or appearing to require more support should be referred to an appropriate crisis help line	<u>If "strong" or "very strong</u>:" Proceed immediately to section 12.4.3.<br><br>**<u>If participant reports the intention to end a life (but not imminently)</u>:** Proceed immediately to section 12.4.3.

12.4.2 Counselor Response to Initial Screening for Non-Imminent Risk

12.4.2.1 Discovery of imminent risk to self/others

If participant's responses lead counselor to believe that participant is at imminent risk, or if counselor is unable to determine that risk is non-imminent, discontinue screening process and follow procedures in section 12.3, Imminent Risk. (omitted because contacting the SLP is part of the procedure in sx 12.3. This section also explains what the counselor should do if the SLP is not immediately available.

12.4.2.2 Indication of non-imminent risk to self/others

If participant's responses lead counselor to believe that participant/others are at non-imminent risk for harm, the counselor will perform follow-up screening for non-imminent risk (section 12.4.3).

In the rare event that a participant spontaneously provides a first person account of a reportable offense that is not imminent**,** the counselor will continue the call but will inform the participant that the information he/she has shared is of such a serious nature that the counseling supervisor will also need to talk to the participant before the conclusion of the call. A co-counselor will immediately notify the supervising psychologist, either in person or by phone (see section 12.4.4.).

If the LSP is out of contact at the time of the call, the counselor will inform the participant that a supervisor will call him/her within the next 24 hours. If contact is not made, the LSP will report the incident to Child Protective Services or Adult Protective Services in accordance with legal statute. (The Intervention Director or other clinical supervisor will make an immediate verbal and subsequent written report to the appropriate social service agency based upon the counselor's documented account as indicated above.)

12.4.2.3 Indication of no risk of harm to self/others

If participant's responses lead counselor to believe that participant/others are not at risk for harm, the counselor will proceed as follows:

(1) Provide a short, supportive response
(2) If needed, encourage the participant to seek assistance from a more qualified professional and provide an appropriate referral source
(3) Tactfully attempt to direct the conversation back to smoking cessation related issues.

12.4.3 Follow-Up Screening of (Non-imminent) Homicide/Suicide Risk

Ask the participant if he/she has recently (in the past 2 weeks) discussed this situation with his/her doctor or some other responsible mental health provider and has a follow-up appointment with this provider.

12.4.3.1 Participant has recently discussed situation and has follow-up appointment

If a responsible provider is aware of the immediate situation, ask the participant if he/she would also be willing to receive a follow-up call from your supervisor. Follow the participant's response with reflective comments that are supportive and then steer discussion to prior cessation related topic. (If a participant asks for or appears to require more support, he/she should be referred to the responsible mental health provider unless there is evidence of an imminent threat.) Consult with supervisor at the conclusion of the call. No further action by the counselor is necessary at this time.

12.4.3.2 Participant has discussed situation with provider, but not recently

If the participant has discussed these problems with a responsible provider, but it was 2 weeks ago or more, then suggest that he/she discuss these thoughts with his/her provider again. If participant agrees to do so, ask the participant if he/she is also willing to receive a follow up call from your supervisor. Follow

the participant's response with reflective comments that are supportive and then steer discussion to prior cessation related topic. Consult with your supervisor at the conclusion of the call. No other action is required at this time.

12.4.3.3 Participant has not discussed problems with provider & will not agree to

If the participant has not discussed these problems with a responsible provider, and does not intend to contact a provider about these problems, then the counselor should do the following:

- Advise the participant of emergency resources in the participant's community
- Provide a list of emergency phone numbers and offer to directly connect the participant to help
- Inform the participant that you will have to make a referral to your supervisor, who will contact him/her. Consult with supervisor during or at the conclusion of the call.

12.4.4 Counselor Response to Follow-up Screening for Non-Imminent Risk

12.4.4.1 Discovery of imminent risk to self/others

If participant's responses lead counselor to believe that participant is at imminent risk, or if counselor is unable to determine that risk is non-imminent, discontinue screening process, consult the LSP immediately, and follow procedures in section 12.3, Imminent Risk.

> Counselors must follow these procedures, based upon a participant's answers to the interview questions, and should not attempt to perform any independent assessment of homicide/suicide risk, unless the counselor has the appropriate clinical training for such an assessment and related intervention (e.g. licensed psychologist, certified social worker, etc.), and has received prior approval from the Intervention Director to perform such assessments.

12.4.4.2 Indication of non-imminent risk to self/others

If participant's responses lead counselor to believe that participant/others are at non-imminent risk for harm, the counselor will proceed as follows:

(1) Provide a short, supportive response
(2) Confirm participant is seeing a provider for the problem and make referrals (to mental health clinics) as needed
(3) Tactfully re-direct the conversation back to smoking cessation related issues
(4) Review call and its resolution with LSP at conclusion of call, taking follow-up action as needed/recommended by the LSP

12.4.4.3 Indication of no risk of harm to self/others

If participant's responses lead counselor to believe that participant/others are not at risk for harm, the counselor will proceed as follows:

(1) Provide a short, supportive response
(2) If needed, encourage the participant to seek assistance from a more qualified professional and provide an appropriate referral source
(3) Tactfully re-direct the conversation back to smoking cessation related issues.

12.5 Imminent Risk Documentation and Reporting

According to Washington State statute, mental health practitioners are required to report incidents of child abuse, elder abuse, or abuse of the developmentally disabled to the Department of Social and Human Resources (DSHS) as stipulated by the Revised Code of Washington (RCW) 26.44 and 74.34 (Appendix 2B).

12.5.1 Refer Suspected Instances of a Reportable Offense to Supervisor

In the rare event that a participant spontaneously provides a first person account of a reportable offense <u>that is not imminent</u>**,** the counselor will continue the call but will inform the participant that the information he/she has shared is of such a serious nature that the counseling supervisor will also need to talk to the participant before the conclusion of the call. A co-counselor will immediately notify the supervising psychologist, either in person or by phone. (*Note*: Imminent offenses are immediately referred to the LSP, see section 12.3.)

If the LSP is out of contact at the time of the call, the counselor will inform the participant that a supervisor will call him/her within the next 24 hours.

12.5.2 Intervention Director's Assessment and Reporting of Reportable Offenses

The LSP will conduct a clinical assessment at first available opportunity. If the LSP determines a reportable offense has occurred, he/she will make immediate verbal (1-866-ENDHARM) and subsequent written notification to the appropriate division of the Department of Social Human Services (DSHS). (If unable to reach the participant to make a clinical assessment, the LSP or Intervention Manager will report the incident to Child Protective Services or Adult Protective Services in accordance with legal statute. The Intervention Director or other clinical supervisor will make an immediate verbal and subsequent written report to the appropriate social service agency based upon the counselor's documented account as indicated above.)

Verbal and written reports will include as much of the following information as available: (1) identification of the vulnerable individual; (2) the nature and extent of the suspected abuse or neglect; (3) evidence of previous abuse or neglect; (4) the name, address and institutional affiliation of the Intervention Director.

12.5.3 Project Documentation

Counselors will complete and sign an Emergency Incident Form (Appendix 2), documenting any and all incidents involving reportable offenses and participants at imminent risk, action(s) taken, outcome(s), and follow-up required. The counselor will give copies to the Intervention Manager, the Principal Investigator, and to the Data Operations Manager. The Data Operations Manager will notify IRB and the NIH, as required.

REFERENCES

Baer JS, Kivlahan DR, Donovan DM. Integrating skills training and motivational therapies. Implications for treatment of substance dependence. Journal of Substance Abuse Treatment, 1999;17:15-23.

California Smokers' Helpline. BUTTsOUT #1 and #2. San Diego, CA: California Department of Health Services. 1998.

Center for Health Studies, GHC. Fresh Start Pediatric Smoking Cessation Study Materials Notebook. Seattle, WA: Center for Heath Studies. Group Health Cooperative. July 1997.

Curry SJ. Self-help interventions for smoking cessation. Journal of Consulting and Clinical Psychology, 1993;61(5):790-803.

Curry SJ, McBride C, Grothous LC, Louie D. A randomized trial of self-help materials, personalized feedback and telephone counseling with nonvolunteer smokers. Journal of Consulting and Clinical Psychology, 1995;63(6):1005-1014.

DiFranza JR, Rigotti NA, McNeill AD, Ockene JK, Savageau JA, St. Cyr, D, Colemen M. Initial symptoms of nicotine dependence in adolescents. Tobacco Control, 2000;9:313-319.

Fiore MC, Bailey WC, Cohen SF, et al. Treating Tobacco Use and Dependence. Clinical Practice Guidelines. Rockville, MD: U.S. Department of Health and Human Services. Public Health Service. June 2000.

Fiore MC, Novotny TE, Pierce JP, Giovino GA, Hatziandreu EJ, Newcomb PA, Surawicz TS, Davis RM. Methods used to quit smoking in the United States. Journal of the American Medical Association, 1990;263:2760-2765.

Hutchinson Smoking Prevention Project. Background Information for Teachers. Seattle, WA: Fred Hutchinson Cancer Research Center. Rev. 1990.

Leventhal H, Fleming R, Glynn K. A cognitive-developmental approach to smoking intervention. In: Maes S, Spielberger CD, Defares PB, Sarason IG, editors. Topics in health psychology: proceedings of the first expert conference in health psychology. New York: John Wiley & Sons, Inc., 1998.

Mayhew KP, Flay BR, Mott JA. Review: Stages in the development of adolescent smoking. Drug and Alcohol Dependence, 2000;59(Suppl 1):S61-S81.

McNeill AD. The development of dependence on smoking in children. British Journal of the Addictions, 1991;86(5):589-592.

Miller WR, Rollnick S. Motivational Interviewing: Preparing People for Change. Second Edition. New York, NY: The Guilford Press, 2002.

Miller WR, Rollnick S. Motivational Interviewing. Preparing People to Change Addictive Behavior. New York, NY: The Guilford Press, 1991.

Miller WR, Zweben A, DiClemente CC, Rychtarik RG. Motivational Enhancement Therapy Manual. Rockville, MD: Department of Health and Human Services. Public Health Service. National Institute on Alcohol Abuse and Alcoholism, NIH Publication No. 94-3723, 1995.

Pallonen UE. Transtheoretical measures for adolescent and adult smokers: similarities and differences. Preventive Medicine, 1998;27:A29-A38.

Prochaska JO. A stage paradigm for integrating clinical and public health approaches to smoking cessation. Addictive Behaviors, 1996;21(6):721-732.

Prochaska JO, DiClemente CC, Norcross JC. In search of how people change: applications to addictive behaviors. American Psychologist, 1992;47(9):1102-1114.

Prochaska JO, DiClemente CC, Velicer WF, Rossi JS. Standardized, individualized, interactive and personalized self-help programs for smoking cessation. Health Psychology, 1993;12(5):399-405.

Prochaska JO, Redding CA, Evers KE. The Transtheoretical Model and stages of change. In: Glanz K, Lewis FM, Rimer BK. Health Behavior and Health Education: Theory, Research, and Practice. 2nd Edition. San Francisco, Calif: Jossey-Bass, 1997.

Ravicz L, Metzger F. Smoking Cessation Clinic Manual. Seattle, WA: Addictions Treatment Center. Addictions Treatment Center, Veterans Affairs Puget Sound Health Care System, Seattle Division. Rev. 6/2000.

Rollnick S, Mason P, Butler C. Health Behavior Change. A Guide for Practitioners. London: Churchill Livingstone, 1999.

Zhu S-H, Pierce JP. A new scheduling method for time-limited counseling. Professional Psychology: Research and Practice, 1995;26(6):624-625.

Zhu S-H, Stretch V, Balabanis M., Rosbrook B. Sadler G, Pierce JP. Telephone counseling for smoking cessation : effects of single-session and multiple-session interventions. Journal of Consulting and Clinical Psychology, 1996;64(1):202-211.

Zhu S-H, Tedeschi GJ, Anderson CM, Pierce JP. Telephone counseling for smoking cessation: What’s in a call? Journal of Counseling & Development, 1998;75:93-102.

APPENDICES

APPENDIX 1: CONSENT DOCUMENTS
Student Information Letter to be printed on colored stationery with Matchbreaker logo
7/31/01 Revised 6/24/02

Address

Dear [student first name]:

Remember us? Last spring, as a junior at [name of high school], you participated in the Survey of High School Juniors. Thank you! That survey was conducted in 14 Washington high schools as part of the Fred Hutchinson Cancer Research Center's Study of High School Smoking.

We learned a lot of interesting things from all of you – for example, when it comes to smoking, you and your classmates are a very diverse bunch. Now we're gearing up for the next exciting phase of our research: the Matchbreaker Program. Matchbreaker is a unique, cutting-edge program with two goals:

(1) To **learn** even more about your insights and individual choices with regard to smoking by hearing **directly from you**. Only you can tell us your frank and personal opinions about smoking and not smoking, and we want to hear what you have to say.

(2) To provide you, in return, with amazing facts, access to a new, exclusive Web site (www.matchbreaker.org), and other great stuff. For those who want it, we can also help with stopping smoking, or provide tips and advice for helping a friend or loved one to quit.

But we want you to know that Matchbreaker is NOT about pressuring smokers to quit. Our goal is to listen and learn from participants, and provide whatever help or support they want, if any.

Your voice counts and is important to us. Whether you're a smoker, a non-smoker, or something in-between, you have unique and valuable insights to contribute to our cancer prevention efforts. And participation is **easy** – *just talking on the phone!* The enclosed Matchbreaker brochure is for you; it will tell you much more about Matchbreaker and the benefits of participation.

Very soon, a Matchbreaker staff person will call to tell you more and answer your questions. You'll be invited to be part of this exciting **research**. Of course, that phone call, like all the contact we have with you, will be strictly **confidential**. And, as always, participation is **voluntary** – you may ask questions, or decline any Matchbreaker phone call whenever you wish. If you have questions that are not answered by the brochure or this letter, please feel free to call me toll-free at 1-866-524-0235.

Thank you so much for your support of the school survey last spring. We look forward to calling and including your ideas and insights in this important research program.

Sincerely,

Kathleen A. Kealey
Manager, Matchbreaker Program
Hutchinson Study of High School Smoking

APPENDIX 1: CONSENT DOCUMENTS (cont'd)
Student Frequently Asked Questions Brochure Revised 06/24/02

All about Matchbreaker

Answers to frequently asked questions about
Matchbreaker & the Hutchinson Study of High School Smoking

What is Matchbreaker?
Matchbreaker is a unique opportunity for *both smokers and nonsmokers* to share their frank and personal **opinions about smoking** during one or more **brief** telephone **calls**. In return, Matchbreaker offers participants amazing facts, access to a fun and informative Web site (www.matchbreaker.org), and other great stuff. For those who want it, we can also help with stopping smoking, or provide tips and advice for helping a friend or loved one to stop. By taking part in Matchbreaker, you will be helping the Fred Hutchinson Cancer Research Center with important cancer prevention research.

What if I don't smoke?
Since Matchbreaker is for both smokers and nonsmokers, your views as a nonsmoker are important to include in our research. We're interested in knowing more about your choice not to smoke; **your experience** may offer insights that will prove **helpful** to others.

I hardly ever smoke — just once in awhile, like now and then at a party. Do you want me to participate?
Absolutely, yes, we'd like your help. Many people smoke very occasionally or infrequently. We'd like to know more about that.

What if I smoke and don't want to stop?
That's **your decision**; we respect it. We know that stopping smoking is a personal choice. We value your participation so that we can better understand the thoughts and ideas of people who smoke and don't want to quit.

Is participation in Matchbreaker voluntary?
Yes, participation in Matchbreaker is **voluntary**. You may **ask questions or decline** any Matchbreaker phone call whenever you wish.

Helping with research is good, but how does participating in the Matchbreaker Program help me?
All participants stand to benefit from the opportunity to frankly and confidentially share and discuss their ideas and thoughts about smoking and not smoking with a caring and impartial Matchbreaker staff member. As a participant, **your voice counts** and is valued.

Matchbreaker also has some great stuff that we can send you, if you're interested. And, if you have Internet access, you will be invited to visit our Web site (www.matchbreaker.org) – it has original comics, amazing facts, and an opportunity for one-on-one, confidential chat with a Matchbreaker staffer.

In addition, participants who remain smoke-free or who stop smoking may experience tremendous long- and short-term benefits, which may include:

- Greatly **reduced risks** of
  - lung **cancer** and other cancers
  - heart disease, stroke, and high blood pressure
  - smoking by your own [future] children
- **Freedom** from nicotine addiction
- Increased stamina and lung capacity when **playing sports** (if you smoke and stop)

- **Savings of money** otherwise spent on cigarettes (if you smoke and stop)
- Cleaner, healthier skin and hair, whiter teeth, fresher breath – the list goes on and on!

So, what's the downside, if any?
The downsides to participation are very few and unlikely:

- Matchbreaker telephone calls may be overheard by others in the room with the participant, causing them to wonder if the participant smokes.
- Some smokers who quit may have **short-lived withdrawal** symptoms, such as nicotine cravings, depressed mood, insomnia, or anxiety. Matchbreaker staff will help smokers who stop to anticipate and cope with any withdrawal symptoms.

What if my parents or others were ever to ask you what we talk about? Will you tell them?
No, we're not allowed to do that. As part of a research study, the Matchbreaker conversations are **confidential** and cannot be shared with anyone outside the study. [The only exception would be if you share information indicating that you or others are at immediate risk of being harmed. In that rare event, Matchbreaker staff would follow the program's procedures to get needed help to whomever needs it – even if that would mean breaking a confidence.]

Who will call me?
Calls are made by Matchbreaker staff at the Fred Hutchinson Cancer Research Center; all have training, experience, and interest in talking with people about smoking-related issues. (And, they really like talking on the phone!)

How many Matchbreaker phone calls will I receive?
It depends. Every participant will receive at least **one call**. Beyond that, the number of calls will depend on **your own interest**. We are able to offer up to 10 total calls, if you wish.

I no longer go to high school or have transferred. Am I still in the study?
Yes. You may still take part even if you've moved, transferred, or otherwise left your school. Having participants in various life situations actually helps the research.

What do other people say about participating in Matchbreaker?
A lot of our participants say that they **like** the opportunity of **helping with research**; some even comment that it's "fun." Many let us know that they like our respectfulness and appreciation of them as participants. And some of our participants even stopped smoking with help from the Matchbreaker staff. When one participant told us, "*You helped me,*" it really made our day! We are hoping that what we've learned from these participants, and may learn from you and others, will help many people with their own future decisions about smoking.

Did we answer all YOUR questions?
If not, please call us toll-free; we're happy to talk to you.
For questions about the Matchbreaker program or telephone calls, call Kathleen Kealey or Li Ravicz toll-free at 1-866-524-0235.
For specific questions about study participants' rights in research studies, contact Sue Mann toll-free at 1-800-245-8790.

Hutch Logo (add: Advancing Knowledge, Saving Lives)

Supported by a grant from the National Cancer Institute, R01-CA82569.

APPENDIX 1: CONSENT DOCUMENTS (cont'd)
Rapport-Building Postcard
6/24/02

RECIPIENTS: To the Parents of all SPs who completed a baseline survey.

FHCRC LOGO + graphic that says 'THANK YOU!' Printed on an interesting-colored card.

We have some exciting news to share with you. The Survey of High School Juniors, in which your son or daughter participated this past spring, had a participation rate of over 90%!

This high level of survey participation means that the views of a diverse group of high school juniors were included. The Hutchinson Study of High School Smoking is grateful for the outstanding support by parents, students, and the 14 collaborating high schools that resulted in such high participation. You have contributed to a great start to our important cancer prevention research. Thank you!

Please always feel welcome to call us toll-free, at 1-800-245-8790, with any questions you may have. Enjoy your summer!

Sincerely,

Sue Mann
Data Operations Manager

APPENDIX 1: CONSENT DOCUMENTS (cont'd)
Active Parental Consent Letter (Day 0) – for parents of < 18 year old potential participants
6/24/02 *[printed on Matchbreaker letterhead, with Matchbreaker logo]*

Inside address

Dear [honorific, last name of Parent or Guardian]:

I'm writing to tell you about some thought-provoking results from last spring's successful Survey of High School Juniors, conducted in 14 diverse Washington state high schools. I also want to introduce the next exciting phase of the Hutchinson Study of High School Smoking – the Matchbreaker program.

What we learned:
Over 90% of the Class of 2003 took part in the survey! This means that the voices of a diverse group of high school juniors were heard – an excellent result in and of itself. Among other things, we learned that:

- Most do not smoke, and many would like to know how to help friends or family who want to quit smoking.
- Some smoke a cigarette only now and then, and don't think it's any big deal.
- Others smoke on a regular basis; of these, some don't want to quit at all, some want to quit in the distant future, and some want to quit soon.

What's next?
Next we'd like to learn even more about the Class of 2003's insights about smoking and non-smoking. We designed the Matchbreaker program to help us discover, for example:

• Why people decided against smoking<br>• Why some smoke only now and then	• Why some people might want to keep smoking<br>• How best to help people who want to quit

How will Matchbreaker do this?
Matchbreaker staff will telephone potential participants and ask them to share their frank and personal opinions about smoking and non-smoking. In return, Matchbreaker will offer participants amazing facts, an assortment of interesting materials, and access to our fun and informative Website (www.matchbreaker.org). (And if, at any time, a smoker happens to be interested in quitting, Matchbreaker's state-of-the-art program will offer help.)

Please take some time now to read the enclosed Frequently Asked Questions (FAQ) brochure. If you have questions not answered by the FAQ, please feel free to call me toll-free at 1-866-524-0235.

With your permission, we'll mail [TP's first name] some advance information about Matchbreaker and then telephone [him/her] to answer any questions and ask if [he/she] wants to take part. **To let us know that we have your permission to contact [TP's first name] so that [he/she] can make a personal decision about participation, please complete the enclosed Matchbreaker Reply Card, and return it in the envelope provided.**

We believe Matchbreaker is a unique opportunity for both smokers and non-smokers to contribute information that will be priceless in planning our future cancer prevention efforts. Thank you for your support.

Sincerely,

Kathleen A. Kealey
Manager, Matchbreaker Program

APPENDIX 1: CONSENT DOCUMENTS (cont'd)
Parent Information Letter – for parent of 18 year old potential participants
6/24/02 *[printed on Matchbreaker letterhead, with Matchbreaker logo]*

Inside address

Dear [honorific, last name of Parent or Guardian]:

I'm writing to tell you about some thought-provoking results from last spring's successful Survey of High School Juniors, conducted in 14 diverse Washington state high schools. I also want to introduce the next exciting phase of the Hutchinson Study of High School Smoking – the Matchbreaker program.

What we learned:
Over 90% of the Class of 2003 took part in the survey! This means that the voices of a diverse group of high school juniors were heard – an excellent result in and of itself. Among other things, we learned that:

- Most do not smoke, and many would like to know how to help friends or family who want to quit smoking.
- Some smoke a cigarette only now and then, and don't think it's any big deal.
- Others smoke on a regular basis; of these, some don't want to quit at all, some want to quit in the distant future, and some want to quit soon.

What's next?
Next we'd like to learn even more about the Class of 2003's insights about smoking and non-smoking. We designed the Matchbreaker program to help us discover, for example:

• Why non-smokers decided against smoking<br>• Why some people smoke only now and then	• Why some people might want to keep smoking<br>• How best to help people who want to quit

How will Matchbreaker do this?
Matchbreaker staff will telephone potential participants and ask them to share their frank and personal opinions about smoking and non-smoking. In return, Matchbreaker will offer participants amazing facts, an assortment of interesting materials, and access to our fun and informative Website (www.matchbreaker.org). (And if, at any time, a smoker happens to be interested in quitting, Matchbreaker's state-of-the-art program will offer help.)

For your information, I've enclosed a Frequently Asked Questions (FAQs) brochure about Matchbreaker. (If you have questions not answered by the brochure, please feel free to call me toll-free at 1-866-524-0235.)

Within a few weeks, we=ll mail [name] some advance information about Matchbreaker. Then, a Matchbreaker staff member will call [him/her]. *Since [name] is at least 18 years old*, we will ask [him/her] directly about participation.

We believe Matchbreaker is a unique opportunity for both smokers and non-smokers to contribute information that will be priceless in planning our future cancer prevention efforts. Thank you for your support.

Sincerely,

Kathleen A. Kealey
Manager, Matchbreaker Program
Hutchinson Study of High School Smoking

APPENDIX 1: CONSENT DOCUMENTS (cont'd)
Matchbreaker Parent FAQ 6/24/02

Frequently Asked Questions from Parents
MATCHBREAKER Program
The Hutchinson Study of High School Smoking
1100 Fairview Ave. N, MP-603 Seattle, WA 98109
1-866-524-0235 (toll-free)

What is Matchbreaker?
Matchbreaker is a unique opportunity for *both smokers and non-smokers* to share their frank and personal opinions about smoking during one or more brief telephone calls. In return, Matchbreaker will offer participants amazing facts, an assortment of interesting materials, and access to our fun and informative Website (www.matchbreaker.org). (And, if at any time, a smoker happens to be interested in quitting, Matchbreaker will offer state-of-the-art help.) Taking part in Matchbreaker will also help the Center's important cancer prevention research.

What if my son/daughter doesn't smoke?
That's terrific! Since Matchbreaker is for both smokers and non-smokers, your son/daughter's views as a non-smoker will be very important to include in our research.

What if my son/daughter smokes and doesn't want to quit?
That's his/her decision; we respect it. We value your son/daughter's participation so we can better understand the insights of older teens who smoke and don't want to quit.

Is Matchbreaker participation voluntary?
Yes. Participation in Matchbreaker is voluntary. Also, participants may ask questions, or decline any Matchbreaker phone call whenever they wish.

What are the benefits to participation in Matchbreaker?
All participants stand to benefit from an opportunity to frankly and confidentially discuss their opinions about smoking and non-smoking with a caring and impartial Matchbreaker staff member. Each participant's voice counts, and is valued.

Are there other benefits to Matchbreaker participation?
Yes. Matchbreaker also has some innovative materials we will mail to interested participants. And, those with Internet access can go to our Matchbreaker Web Site (www.matchbreaker.org), which has great comics, amazing facts, and an opportunity for one-on-one confidential chat with a Matchbreaker staff member.

In addition, Matchbreaker participants who remain smoke-free or who quit smoking may experience tremendous long- and short-term benefits, which may include . . .

- Greatly reduced risks of:
  - lung cancer and other cancers
  - heart disease
  - stroke
  - high blood pressure
  - smoking by their [future] children
- Freedom from nicotine addiction
- Savings of money otherwise spent on cigarettes by participants who quit smoking

Are there any possible downsides to participation in Matchbreaker?
Very few, and very unlikely . . .

- The Matchbreaker conversations could possibly be overheard by others in the room with participants, causing them to wonder if the participant smokes.
- Smokers who quit may have short-lived withdrawal symptoms, such as cravings, depressed mood, insomnia, or anxiety. The Matchbreaker staff will help smokers who quit to handle these symptoms.

Can you tell me what my son/daughter talks about during the Matchbreaker calls?
Sorry, but we aren't allowed to do that. As part of a research study, the Matchbreaker conversations are confidential. As such, they cannot be shared with anyone outside the study. [The only exception to this rule would be if a Matchbreaker staff member happened to learn of a clear and immediate danger to your son/daughter or others. In such a rare event, Matchbreaker's procedures would get help to whomever needs it – even if it means breaking a confidence.]

Who will make the Matchbreaker calls?
Calls are made by Matchbreaker staff from the Fred Hutchinson Cancer Research Center. All staff members have training and experience in talking with older teens about smoking and non-smoking. They are supervised by a licensed Clinical Psychologist.

How many Matchbreaker phone calls will participants receive?
It depends. Every Matchbreaker participant will be called at least once. Beyond that, the number of calls will depend on the interest of each participant. We are able to offer up to ten calls.

My son/daughter no longer goes to high school or has transferred. Is he/she still in the study?
Yes. Your son/daughter is eligible to take part even if he/she has moved, transferred, or otherwise left school. Having participants in various life situations is a tremendous help to the research.

Will Matchbreaker give participants who smoke the Apatch@ or nicotine gum?@
No. We are not authorized to provide this service. Moreover, the scientific studies to show whether these products work for younger smokers, or less frequent smokers, are not yet complete.

Does participation in Matchbreaker cost anything?
No. The Matchbreaker calls and materials are offered free of charge to all participants.

Will you ask participants about their parents' smoking habits, or about drug or alcohol use?
No. We abide by a strict rule that we will not ask participants for any information about their parents, or about drug or alcohol use.

Will you give our name, address, or phone number to anyone else?
No. Like all information in this research study, this information is also confidential.

What do I do if I have more questions?
Please call us toll-free at one of the numbers below; we're happy to talk to you.

- Questions about Matchbreaker: Kathleen Kealey (Matchbreaker Manager) or Li Ravicz, PhD (Clinical Psychologist and Matchbreaker Director), toll-free at 1-866-524-0235.
- Questions about study participants= rights in research studies: Sue Mann (Data Operations Manager) toll-free at 1-800-245-8790.

Hutch logo + Advancing Knowledge, Saving Lives
Supported by a grant from the National Cancer Institute, R01-CA82569.

APPENDIX 1: CONSENT DOCUMENTS (cont'd)
Active Parental Consent Thank You/Reminder Card Day 5
6/24/02

[Include an eye-grabbing graphic – not FHCRC logo]

Dear Parent or Guardian:

A few days ago, I wrote you about the Hutchinson Center's Matchbreaker program – a unique opportunity for older teens from several diverse Washington state high schools. We are ready to contact your son/daughter about Matchbreaker so that he/she can ask questions and make a personal decision about participation. But first, we need a Reply Card from you.

If you=ve already mailed us your completed Reply Card, thank you!

If you have not yet mailed your Reply Card, please take a minute to fill it out and mail it today. If you need another Reply Card, please call me toll-free at 1-866-524-0235, and I=ll be happy to send you one. Also, please feel free to call me with any questions you may have. Thank you for your help.

Sincerely,

Kathleen A. Kealey
Manager, Matchbreaker Program

APPENDIX 1: CONSENT DOCUMENTS (cont'd)
Active Parental Consent Telephone Non-Responder Script Day 16+
6/24/02

<u>Introduction</u>:
*"Hello. May I please speak with [parent=s name]?
This is [telephone interviewer=s name] with the Fred Hutchinson Cancer Research Center. I'm calling about a research study program called Matchbreaker. We sent you some info about Matchbreaker a couple of weeks ago. I hope you had a chance to look it over…. "*

[If not, cut to <u>Matchbreaker Description</u>].
[If it is *clear* that the parent got & read the info, but just hasn't gotten around to completing/mailing a card, cut to <u>Reply Card Completion</u>.]
[If Parent says he/she just mailed their Reply Card, thank them and say you'll call in a couple of days if we don't receive it.]

<u>Reply Card Completion:</u>
"Great. The Matchbreaker calls are going very well. The older teen participants seem to like having a chance to talk frankly about tobacco issues, and we are learning so much. Do you have any questions about Matchbreaker?" [Pause. Respond appropriately.]

"Okay. Now, so you don't have to worry about finding your Reply Card, we can take care of it over the phone; it will just take a minute. Okay?" [Reply appropriately.]

"First, I want to remind you that [TP's name]'s participation in Matchbreaker is voluntary and that the content of the calls is confidential. With your permission, we'll mail [TP's name] an info packet and phone [him/her]. Then, [TP's name] can then make a personal decision about participating. Is it okay for Matchbreaker to contact [name of student]?"

[Complete Reply Card]

"Thank you so much."

<u>Matchbreaker Description</u>:
"Well, I'd be happy to tell you a bit about Matchbreaker if you've go t a couple of minutes. In a nutshell, Matchbreaker is part of the Hutchinson Study of High School Smoking. It was designed to offer smokers and non-smokers from the Class of 2003 a unique opportunity to share their frank and personal opinions about smoking during one or more brief telephone calls. In return, Matchbreaker will offer participants various innovative materials and access to our fun and informative Website. And, if a smoker happens to decide to quit, Matchbreaker will offer state-of-the-art help. Taking part in Matchbreaker will also help the Center's important cancer prevention research."

"Matchbreaker participation is voluntary and the content of the calls is confidential. With your permission, we'll mail [TP's name] an info packet and phone [him/her] so that [TP's name] can make a personal decision about participating. Do you have any questions about Matchbreaker?" [Respond]

"Is it okay for us to contact [TP's name]?"

[Complete Reply Card]

APPENDIX 2A: EMERGENCY INCIDENT FORM

HS Study Emergency Incident Form

TP Name: ____ Counselor: __________________________

Date / Time: ___________________ Contact type: |__| Phone |__| E-mail Contact initiated by: __________________________

Description of Problem/Situation:

Counselor action:

1. **Referral(s):** |__| None |__| Counseling supervisor, ____

|__| Family doctor |__| School nurse / Counselor, School: ____

|__| Community Helpline, Name/#: ____

|__| Local Crisis Line/Alcohol-Drug Helpline, Name/#: ____

|__| Emergency medical assistance (dial 9 - 9-1-1) |__| Washington DSHS

|__| Other, specify: ____

2. **Details:**

Follow-up:

Action Needed: |__| No |__| Yes, by whom: _______________________, When:______________________________________

Details: ____

____

Form Completed: (Date) _______________________________ Initials: ___________________________

Supervisor's Review:

____

____

(Date) _____________________________ Initials: ___________________________

Outcome (if known):

____

(Date) _____________________________ Initials: ___________________________

Copies to: |__| Intervention Manager |__| Data Operations Manager. |__| PI

APPENDIX 2B: Revised Code of Washington (RCW) 26.44 and 74.34

RCW 26.44.030
Reports -- Duty and authority to make -- Duty of receiving agency -- Duty to notify -- Case planning and consultation -- Penalty for unauthorized exchange of information -- Filing dependency petitions -- Interviews of children -- Records -- Risk assessment process--Reports to legislature.
(1)(a) When any practitioner, county coroner or medical examiner, law enforcement officer, professional school personnel, registered or licensed nurse, social service counselor, psychologist, pharmacist, licensed or certified child care providers or their employees, employee of the department, juvenile probation officer, placement and liaison specialist, responsible living skills program staff, HOPE center staff, or state family and children's ombudsman or any volunteer in the ombudsman's office has reasonable cause to believe that a child has suffered abuse or neglect, he or she shall report such incident, or cause a report to be made, to the proper law enforcement agency or to the department as provided in RCW 26.44.040.

(b) The reporting requirement also applies to department of corrections personnel who, in the course of their employment, observe offenders or the children with whom the offenders are in contact. If, as a result of observations or information received in the course of his or her employment, any department of corrections personnel has reasonable cause to believe that a child has suffered abuse or neglect, he or she shall report the incident, or cause a report to be made, to the proper law enforcement agency or to the department as provided in RCW 26.44.040.

(c) The reporting requirement shall also apply to any adult who has reasonable cause to believe that a child who resides with them, has suffered severe abuse, and is able or capable of making a report. For the purposes of this subsection, "severe abuse" means any of the following: Any single act of abuse that causes physical trauma of sufficient severity that, if left untreated, could cause death; any single act of sexual abuse that causes significant bleeding, deep bruising, or significant external or internal swelling; or more than one act of physical abuse, each of which causes bleeding, deep bruising, significant external or internal swelling, bone fracture, or unconsciousness.

(d) The report must be made at the first opportunity, but in no case longer than forty-eight hours after there is reasonable cause to believe that the child has suffered abuse or neglect. The report must include the identity of the accused if known.

(2) The reporting requirement of subsection (1) of this section does not apply to the discovery of abuse or neglect that occurred during childhood if it is discovered after the child has become an adult. However, if there is reasonable cause to believe other children are or may be at risk of abuse or neglect by the accused, the reporting requirement of subsection (1) of this section does apply.

(3) Any other person who has reasonable cause to believe that a child has suffered abuse or neglect may report such incident to the proper law enforcement agency or to the department of social and health services as provided in RCW 26.44.040.

(4) The department, upon receiving a report of an incident of alleged abuse or neglect pursuant to this chapter, involving a child who has died or has had physical injury or injuries inflicted upon him or her other than by accidental means or who has been subjected to alleged sexual abuse, shall report such incident to the proper law enforcement agency. In emergency cases, where the child's welfare is

endangered, the department shall notify the proper law enforcement agency within twenty-four hours after a report is received by the department. In all other cases, the department shall notify the law enforcement agency within seventy-two hours after a report is received by the department. If the department makes an oral report, a written report must also be made to the proper law enforcement agency within five days thereafter.

(5) Any law enforcement agency receiving a report of an incident of alleged abuse or neglect pursuant to this chapter, involving a child who has died or has had physical injury or injuries inflicted upon him or her other than by accidental means, or who has been subjected to alleged sexual abuse, shall report such incident in writing as provided in RCW 26.44.040 to the proper county prosecutor or city attorney for appropriate action whenever the law enforcement agency's investigation reveals that a crime may have been committed. The law enforcement agency shall also notify the department of all reports received and the law enforcement agency's disposition of them. In emergency cases, where the child's welfare is endangered, the law enforcement agency shall notify the department within twenty-four hours. In all other cases, the law enforcement agency shall notify the department within seventy-two hours after a report is received by the law enforcement agency.

(6) Any county prosecutor or city attorney receiving a report under subsection (5) of this section shall notify the victim, any persons the victim requests, and the local office of the department, of the decision to charge or decline to charge a crime, within five days of making the decision.

(7) The department may conduct ongoing case planning and consultation with those persons or agencies required to report under this section, with consultants designated by the department, and with designated representatives of Washington Indian tribes if the client information exchanged is pertinent to cases currently receiving child protective services. Upon request, the department shall conduct such planning and consultation with those persons required to report under this section if the department determines it is in the best interests of the child. Information considered privileged by statute and not directly related to reports required by this section must not be divulged without a valid written waiver of the privilege.

(8) Any case referred to the department by a physician licensed under chapter 18.57 or 18.71 RCW on the basis of an expert medical opinion that child abuse, neglect, or sexual assault has occurred and that the child's safety will be seriously endangered if returned home, the department shall file a dependency petition unless a second licensed physician of the parents' choice believes that such expert medical opinion is incorrect. If the parents fail to designate a second physician, the department may make the selection. If a physician finds that a child has suffered abuse or neglect but that such abuse or neglect does not constitute imminent danger to the child's health or safety, and the department agrees with the physician's assessment, the child may be left in the parents' home while the department proceeds with reasonable efforts to remedy parenting deficiencies.

(9) Persons or agencies exchanging information under subsection (7) of this section shall not further disseminate or release the information except as authorized by state or federal statute. Violation of this subsection is a misdemeanor.

(10) Upon receiving reports of alleged abuse or neglect, the department or law enforcement agency may interview children. The interviews may be conducted on school premises, at day-care facilities, at the child's home, or at other suitable locations outside of the presence of parents.

Parental notification of the interview must occur at the earliest possible point in the investigation that will not jeopardize the safety or protection of the child or the course of the investigation. Prior to commencing the interview the department or law enforcement agency shall determine whether the child wishes a third party to be present for the interview and, if so, shall make reasonable efforts to accommodate the child's wishes. Unless the child objects, the department or law enforcement agency shall make reasonable efforts to include a third party in any interview so long as the presence of the third party will not jeopardize the course of the investigation.

(11) Upon receiving a report of alleged child abuse and neglect, the department or investigating law enforcement agency shall have access to all relevant records of the child in the possession of mandated reporters and their employees.

(12) The department shall maintain investigation records and conduct timely and periodic reviews of all cases constituting abuse and neglect. The department shall maintain a log of screened-out nonabusive cases.

(13) The department shall use a risk assessment process when investigating alleged child abuse and neglect referrals. The department shall present the risk factors at all hearings in which the placement of a dependent child is an issue. Substance abuse must be a risk factor. The department shall, within funds appropriated for this purpose, offer enhanced community-based services to persons who are determined not to require further state intervention.

The department shall provide annual reports to the legislature on the effectiveness of the risk assessment process.

(14) Upon receipt of a report of alleged abuse or neglect the law enforcement agency may arrange to interview the person making the report and any collateral sources to determine if any malice is involved in the reporting.

(15) The department shall make reasonable efforts to learn the name, address, and telephone number of each person making a report of abuse or neglect under this section. The department shall provide assurances of appropriate confidentiality of the identification of persons reporting under this section. If the department is unable to learn the information required under this subsection, the department shall only investigate cases in which: (a) The department believes there is a serious threat of substantial harm to the child; (b) the report indicates conduct involving a criminal offense that has, or is about to occur, in which the child is the victim; or (c) the department has, after investigation, a report of abuse or neglect that has been founded with regard to a member of the household within three years of receipt of the referral.

[1999 c 267 § 20; 1999 c 176 § 30; 1998 c 328 § 5; 1997 c 386 § 25; 1996 c 278 § 2; 1995 c 311 § 17. Prior: 1993 c 412 § 13; 1993 c 237 § 1; 1991 c 111 § 1; 1989 c 22 § 1; prior: 1988 c 142 § 2; 1988 c 39 § 1; prior: 1987 c 524 § 10; 1987 c 512 § 23; 1987 c 206 § 3; 1986 c 145 § 1; 1985 c 259 § 2; 1984 c 97 § 3; 1982 c 129 § 7; 1981 c 164 § 2; 1977 ex.s. c 80 § 26; 1975 1st ex.s. c 217 § 3; 1971 ex.s. c 167 § 1; 1969 ex.s. c 35 § 3; 1965 c 13 § 3.]

NOTES:

Reviser's note: This section was amended by 1999 c 176 § 30 and by 1999 c 267 § 20, each without reference to the other. Both amendments are incorporated in the publication of this section under RCW 1.12.025(2). For rule of construction, see RCW 1.12.025(1).

Findings -- Intent -- Severability -- 1999 c 267: See notes following RCW 43.20A.790.

Short title -- Purpose -- Entitlement not granted -- Federal waivers -- 1999 c 267 §§ 10-26: See RCW 74.15.900 and 74.15.901.

Findings -- Purpose -- Severability -- Conflict with federal requirements -- 1999 c 176: See notes following RCW 74.34.005.

Application -- Effective date -- 1997 c 386: See notes following RCW 74.14D.010.

Finding -- Intent--1996 c 278: "The legislature finds that including certain department of corrections personnel among the professionals who are mandated to report suspected abuse or neglect of children, dependent adults, or people with developmental disabilities is an important step toward improving the protection of these vulnerable populations. The legislature intends, however, to limit the circumstances under which department of corrections personnel are mandated reporters of suspected abuse or neglect to only those circumstances when the information is obtained during the course of their employment. This act is not to be construed to alter the circumstances under which other professionals are mandated to report suspected abuse or neglect, nor is it the legislature's intent to alter current practices and procedures utilized by other professional organizations who are mandated reporters under RCW 26.44.030(1)(a)." [1996 c 278 § 1.]

Severability -- 1987 c 512: See RCW 18.19.901.

Legislative findings -- 1985 c 259: "The Washington state legislature finds and declares:

The children of the state of Washington are the state's greatest resource and the greatest source of wealth to the state of Washington. Children of all ages must be protected from child abuse. Governmental authorities must give the prevention, treatment, and punishment of child abuse the highest priority, and all instances of child abuse must be reported to the proper authorities who should diligently and expeditiously take appropriate action, and child abusers must be held accountable to the people of the state for their actions.

The legislature recognizes the current heavy caseload of governmental authorities responsible for the prevention, treatment, and punishment of child abuse. The information obtained by child abuse reporting requirements, in addition to its use as a law enforcement tool, will be used to determine the need for additional funding to ensure that resources for appropriate governmental response to child abuse are available." [1985 c 259 § 1.]

Severability -- 1984 c 97: See RCW 74.34.900.

Severability -- 1982 c 129: See note following RCW 9A.04.080.

Purpose -- Intent -- Severability -- 1977 ex.s. c 80: See notes following RCW 4.16.190.

RCW 74.34.021
Vulnerable adult -- Definition.
For the purposes of this chapter, the term "vulnerable adult" includes persons receiving services from any individual who for compensation serves as a personal aide to a person who self-directs his or her own care in his or her home under chapter 336, Laws of 1999.

[1999 c 336 § 6.]

NOTES:

Finding -- Intent -- 1999 c 336: See note following RCW 74.39.007.

RCW 74.34.035
Reports -- Mandated and permissive -- Contents -- Confidentiality.
(1) When there is reasonable cause to believe that abandonment, abuse, financial exploitation, or neglect of a vulnerable adult has occurred, mandated reporters shall immediately report to the department. If there is reason to suspect that sexual or physical assault has occurred, mandated reporters shall immediately report to the appropriate law enforcement agency and to the department.

(2) Permissive reporters may report to the department or a law enforcement agency when there is reasonable cause to believe that a vulnerable adult is being or has been abandoned, abused, financially exploited, or neglected.

(3) No facility, as defined by this chapter, agency licensed or required to be licensed under chapter 70.127 RCW, or facility or agency under contract with the department to provide care for vulnerable adults may develop policies or procedures that interfere with the reporting requirements of this chapter.

(4) Each report, oral or written, must contain as much as possible of the following information:

(a) The name and address of the person making the report;

(b) The name and address of the vulnerable adult and the name of the facility or agency providing care for the vulnerable adult;

(c) The name and address of the legal guardian or alternate decision maker;

(d) The nature and extent of the abandonment, abuse, financial exploitation, neglect, or self-neglect;

(e) Any history of previous abandonment, abuse, financial exploitation, neglect, or self-neglect;

(f) The identity of the alleged perpetrator, if known; and

(g) Other information that may be helpful in establishing the extent of abandonment, abuse, financial exploitation, neglect, or the cause of death of the deceased vulnerable adult.

(5) Unless there is a judicial proceeding or the person consents, the identity of the person making the report under this section is confidential.

[1999 c 176 § 5.]

NOTES:

Findings -- Purpose -- Severability -- Conflict with federal requirements -- 1999 c 176: See notes following RCW 74.34.005.

RCW 74.34.040
Reports -- Contents -- Identity confidential.
The reports made under *RCW 74.34.030 shall contain the following information if known:

(1) Identification of the vulnerable adult;

(2) The nature and extent of the suspected abuse, neglect, exploitation, or abandonment;

(3) Evidence of previous abuse, neglect, exploitation, or abandonment;

(4) The name and address of the person making the report; and

(5) Any other helpful information.

Unless there is a judicial proceeding or the person consents, the identity of the person making the report is confidential.

[1986 c 187 § 2; 1984 c 97 § 10.]

NOTES:

***Reviser's note:** RCW 74.34.030 was repealed by 1999 c 176 § 35.

RCW 74.34.053
Failure to report -- False reports -- Penalties.
(1) A person who is required to make a report under this chapter and who knowingly fails to make the report is guilty of a gross misdemeanor.

(2) A person who intentionally, maliciously, or in bad faith makes a false report of alleged abandonment, abuse, financial exploitation, or neglect of a vulnerable adult is guilty of a misdemeanor.

[1999 c 176 § 7.]

NOTES:

Findings -- Purpose -- Severability -- Conflict with federal requirements -- 1999 c 176: See notes following RCW 74.34.005.

APPENDIX 3: BASIC PRINCIPLES OF MOTIVATIONAL INTERVIEWING

Motivational Interviewing (MI) strategies enable counselors to (1) mobilize the smoker's inner resources to help him/her resolve ambivalence about behavior change and trigger a decision to change (i.e., quit smoking), and (2) empathically and strategically support the smoker's decision to quit. Miller and Rollnick (1991) have described five basic motivational principles underlying the MI approach:

Express empathy.

Empathy is commonly thought of as "feeling with" people, or having an immediate understanding of their situation. The counselor seeks to communicate respect for the client. Communications that imply a superior – inferior relationship between counselor and client are avoided. The counselor's role is a blend of supportive companion and knowledgeable consultant. The client's freedom of choice and self-direction are respected. Only the clients can decide to make a change in their smoking and carry out that choice. Counselors *listen* rather than *tell*. Persuasion is gentle, subtle, always with the assumption that change is up to the client. In therapeutic settings, the term empathy is often used to describe a specific skill and style of reflective listening (Rogers, 1957). In this style, the counselor listens carefully to what the client is saying, then reflects it back to the client, often in a slightly modified or reframed form.

Develop discrepancy.

Motivation for change occurs when people perceive a discrepancy between where they are and where they want to be. MI seeks to enhance and focus the client's attention on such discrepancies with regard to smoking behavior. In some cases – for example, with precontemplative smokers – it may be necessary first to *develop* such discrepancy by raising the client's awareness of the personal consequences of his/her smoking. Such information, properly presented, can precipitate a "crisis" (critical mass) of motivations for change. As a result, the client may be more willing to enter into a frank discussion of change options in order to reduce the perceived discrepancy and regain emotional equilibrium. Alternately, it may take less time and effort to move a client in the contemplation stage along to the point of making a decision to quit.

Avoid argumentation.

MI explicitly avoids direct argumentation, which tends to evoke resistance. Counselors do not seek to prove or convince by force of argument. Instead, the counselor uses other strategies to help the client see accurately the consequences of his/her smoking and devalue its perceived positive aspects. When MI is conducted properly, the client – <u>not</u> the counselor – voices the arguments for quitting.

Roll with resistance.

A defining characteristic of MI is the manner in which counselors handle client resistance. Counselors "roll with" resistance with a goal of shifting client perceptions in the process. New ways of thinking about smoking are invited but not imposed. Ambivalence is viewed as normal and healthy, and is explored openly. Solutions are usually evoked from the client rather than provided by the counselor.

Support self-efficacy.

Bandura (1982) has described self-efficacy as a critical determinant for behavior change. Self-efficacy is the belief that one can perform a particular behavior or accomplish a particular task. In this case, students must be persuaded that they have the ability to quit smoking and stay quit. Students who are persuaded that they have a problem with their smoking will still not move toward quitting unless there is hope for success.

APPENDIX 4: COMPARING/CONTRASTING MI WITH OTHER THERAPEUTIC APPROACHES

Cognitive-behavioral therapy: Cognitive behavioral approaches prescribe and attempt to teach clients specific coping skills without regard to motivation for change. MI assumes that the key element for lasting change is a motivational shift that instigates a decision and commitment to change.

Cognitive-Behavioral Therapy	Motivational Interviewing
Assumes that the client is motivated; no direct strategies for building motivation for change.	Employs specific principles and strategies for building motivation.
Seeks to identify and modify maladaptive cognitions.	Explores and reflects client perceptions without labeling or correcting them.
Prescribes specific coping strategies.	Elicits possible change strategies from the client.
Teaches coping behaviors through instruction, modeling, directed practice and feedback.	Leaves responsibility for change methods with the client; provides no training, modeling, or practice.
Teaches specific problem-solving strategies.	Elicits natural problem-solving processes from the client.

Confrontation-of-denial: MI differs dramatically from confrontational treatment strategies in which the therapist takes primary responsibility for "breaking down the client's denial." This is the conceptual opposite of MI in which the goal of the counselor is to evoke from the client statements of problem perception and a need for change.

Confrontation-of-Denial Approach	Motivational Interviewing
Emphasizes acceptance of diagnosis or label (with regard to problem behavior) as essential for change.	De-emphasizes labels; acceptance of diagnosis or label not seen as necessary for change to occur.
Emphasizes addiction, which reduces personal choice and control.	Emphasizes personal choice regarding future smoking.
Uses evidence of addiction or other problems with smoking in an attempt to convince client to quit.	Uses objective evaluation, but focuses on eliciting client's own concerns about smoking.
Sees resistance as "denial," which requires confrontation.	Sees resistance as an interpersonal behavior pattern influenced by the counselor's behavior.
Uses argumentation and correction to overcome resistance.	Uses reflection to "roll with" resistance.

Nondirective approach: Counselor does not direct treatment, but follows the client's direction wherever it may lead. In contrast, MI employs systematic strategies toward behavior change. MI is a directive and persuasive approach, not a nondirective, passive approach.

Nondirective Approach	Motivational Interviewing
Allows the client to determine the content and direction of counseling.	Systematically directs the client toward motivation for change.
Avoids injecting the counselor's own advice and feedback.	Offers the counselor's own advice and feedback where appropriate.
Uses empathic reflection noncontingently.	Uses empathic reflection selectively to reinforce certain points.
Explores the clients conflicts and emotions as they are currently.	Seeks to create and amplify the client's discrepancy in order to enhance motivation to change.

APPENDIX 5: CHALLENGING CONVERSATIONS WITH TEEN SMOKERS

1. Challenges During Initial Call

I don't understand why you are calling Me.

We selected both smokers and nonsmokers for the Matchbreaker program, from the juniors who completed our survey last Spring.

I didn't know that when I filled out that form you would be contacting my parents and calling me.

Sounds like its come as a surprise. Future steps in the study were mentioned at the time of the survey last Spring, but I can understand how you might have missed that information. When people under age 18 are involved in research, their parents have to be notified. Do you have any other questions about the Matchbreaker program – this step of the study?

II. Challenges from skeptical smokers

If you don't smoke yourself how can you understand what its like for me?

You think that because I don't smoke I can't understand.
Or
You think that because I'm not a smoker I can't help.

Well you probably know a lot about it from school but that is just talk.

You're right, I don't know what its like for you personally. I do know what other teens have said helped them to stop.

III. Challenges from infrequent smokers

I only smoke every so often. Just a couple of times a month when I'm hanging out with my friends. There is no point in talking about something I almost never do.

You're right, you hardly smoke at all you might never smoke more than that. It could seem pretty silly to talk about something you don't really do. Still, almost 40% of infrequent smokers eventually smoke on a daily basis. What do you think about that?

I guess they were just dumb. [or I don't believe that. / I know I can stop at any time.]

You figure that could never happen to you.

I'm not worried about it one way or the other.

Let me see if I follow you. Because you only smoke occasionally you're not really worried about any possible problems smoking could cause you. You know that many infrequent smokers eventually become heavy smokers but at least right now that isn't much of a concern. If you were to stop smoking now, how do you think you would feel about it?

I'm sure I wouldn't really miss it.

Sounds like you're not really thinking about stopping but if you were to quit it would be pretty easy for you. How do you think your friends would respond?

I don't think they would care. They don't really smoke that much either.

That's cool. You don't have to worry about getting flak for not smoking.

Hey, I think I've begun to get a good sense of how you feel about smoking. Would it be ok with you if I called you back next week and talked with you a little more about your views?

What's the point?

Well, I've been interested in what you have to say, and talking with you some more about it could definitely help me in my discussions with other students about smoking.

OK.

Great. What would be the best day and time to reach you?

IV. Challenges from Smokers who have cut down but not quit

I was smoking a pack a day. Now I'm just smoking a few cigarettes a day. What is the harm in that?

Y*ou figure since you've cut way back there is no harm in smoking every so often.*

I think its ok to smoke as long as it isn't every day.

Hmm, when we first talked you had said that you really wanted to quit entirely. Sounds like something has changed . . .

V. Challenges from smokers who don't think smoking in the short run will hurt them.

Smoking is only bad for you if you do it for a long time.

You feel that no harm can come from smoking now.

Right.

Certainly, the most serious effects of smoking tend to be long-term, but there are also short-term effects. Would you be willing to hear about some of these? (OK) *Well, there is the obvious like bad breath but people also report that they lose some of their sense of smell and taste. For many people premature wrinkling of their skin, decreased stamina and athletic performance and sexual side effects are even bigger problems. How do you feel about these issues?*

VI. Challenges from smokers who don't think they can quit.

I've tried to quit before and I never could. I don't see how talking with you about it will make any difference.

You feel pretty discouraged because of how hard it's been before.

Right!

It is tough when you try something but don't get very far. Tell me about your last quit attempt and what went wrong.

APPENDIX 6: HEALTH EFFECTS OF SMOKING AMONG YOUNG PEOPLE

Smoking has a tremendous negative impact upon health, causing more than one of every six deaths in the United States. Consider the risks of health effects specific to young adult men and women.

Cigarette smokers have a lower level of lung function than those persons who have never smoked.

Smoking reduces the rate of lung growth.

The younger people start smoking cigarettes, the more likely they are to become strongly addicted to nicotine.

Smoking hurts young people's physical fitness in terms of both performance and endurance – even among young people who train in competitive running.

The resting heart rates of young adult smokers are 2-3 beats per minute faster than the resting heart rates of nonsmokers.

Smoking at an early age increases risk of lung cancer. For most smoking-related cancers, the risk rises as the individual continues to smoke.

Teenage smokers suffer from shortness of breath almost three times as often as teens that don't smoke, and produce phlegm more than twice as often as teens that don't smoke.

Teenage smokers are 2.4 times more likely than nonsmokers to have poorer overall health, 2.5 times more likely to report cough with phlegm or blood, shortness of breath when not exercising, and wheezing or gasping. They are 3 times more likely to have seen a doctor or other health professional for an emotional or psychological complaint (e.g., depression) than nonsmokers.

Teens who smoke are more likely than nonsmokers to use alcohol, eight times more likely to use marijuana, and 22 times more likely to use cocaine. Smoking is associated with a host of other risky behaviors, including fighting and engaging in unprotected sex.

In adults, smoking causes heart disease and stroke. Studies have shown that early signs of the blood vessel damage present in these diseases can be found in adolescents who smoke.

Cigarette smoking causes premature wrinkling of the skin.

Smokers report more chronic bronchitis, emphysema, and chronic sinusitis than nonsmoker.

Smoking impairs the sense of smell and damages taste buds, damage that can linger for years.

Smoking causes impotence and decreased fertility in men.

Smoking interferes with the healing process following surgery – it delays wound healing and causes more pronounced scars.

Smoker's, even those with a lean body type, develop "smoker's paunch," an unhealthy distribution of fat around the midsection.

Women's Health

Death rates from heart disease, chronic lung disease, and lung cancer are higher for women who smoke, than for those who don't. Lung cancer is the number one cause of cancer deaths among women.

Use of oral contraceptives in addition to smoking substantially increases a woman's risk for strokes and heart disease.

Women who smoke are at increased risk for adverse pregnancy outcomes, including miscarriage, bleeding during pregnancy, premature labor, delivering a baby with low birth weight, stillbirth, and Sudden Infant Death Syndrome (SIDS).

Women smokers are at increased risk for pre-cancerous changes in the cervix, and cervical cancer.

Smoking may impair fertility.

Women who smoke experience menopause at younger ages than nonsmokers.

Women who smoke are at increased risk for osteoporosis.

Adapted from:
Hutchinson Smoking Prevention Project. Background Information for Teachers. Seattle, WA: Fred Hutchinson Cancer Research Center. Rev. 1990.
www.cdc.gov
Join Together. Quitting Guides. Trustees of Boston University. School of Public Health: Boston, MA. [On-line]. Available: www.quitnet.org; accessed 04/03/01.

APPENDIX 7: ENVIRONMENTAL TOBACCO SMOKE (ETS)

Involuntary smoke or environmental tobacco smoke (ETS) are the names commonly given to the smoke given off by a burning cigarette, cigar, or pipe. It has been called "the most dangerous air pollutant we face today;" it is dangerous to anyone who comes in contact with it. ETS consists of sidestream smoke (the unfiltered smoke from the burning end of the cigarette) and secondhand smoke (the smoke exhaled by the smoker which is then available to be inhaled by both smokers and nonsmokers). Everyone has a right to breathe clean, safe air. Next time you smoke around other people, consider these facts about ETS:

When in a crowded restaurant, ETS can produce six times the pollution of a busy highway.

A person who spends two hours in a room where someone is smoking, inhales the equivalent of four cigarettes, 200 poisons, and 43-cancer-causing agents.

ETS causes 30 times as many lung cancer deaths as all regulated pollutants combined.

ETS makes clothes and hair stink.

ETS causes wheezing, coughing, colds, earaches, and asthma attacks.

ETS causes up to 300,000 lung infections (such as bronchitis or pneumonia) in infants and young children each year. In fact, children who are around smokers are more likely than children from nonsmoking families to have more asthma and allergies, colds and coughs, colic, ear infections, and bronchitis and pneumonia.

ETS fills the air with many of the same poisons found in the air around toxic waste dumps (e.g., cyanide).

ETS causes reddening, itching, and watering of the eyes.

ETS wrecks the smell and taste of food.

Along with ETS deaths from lung cancer (about 3,700) and other lethal cancers and respiratory ailments (about 12,300), the total yearly death toll is 53,000, making ETS the third leading cause of death in the U.S. (behind direct smoking which kills about 435,000 people per year, and alcohol, which kills 100,000).

Nonsmokers who live with smokers have a 30% greater risk of developing lung cancer than do nonsmokers living with nonsmokers.

Even pets of smokers suffer. A Colorado State University study showed that dogs living in homes in which their owners smoked had about a 30% increase in risk of lung cancer.

<u>Adapted from</u>:

Hutchinson Smoking Prevention Project. Background Information for Teachers. Seattle, WA: Fred Hutchinson Cancer Research Center. Rev. 1990.
www.cdc.gov

APPENDIX 8: WHAT'S IN A CIGARETTE?

What's in a cigarette? Aside from tobacco, we're not exactly sure because cigarettes are one of the few products on the market that aren't regulated. Food manufacturers are required by law to provide ingredients on the labels of food products, clothing manufacturers tell you the fabric from which their products are made, and electronics manufacturers are required to demonstrate that electronic appliances and devices are UL-approved. But the cigarette industry is entirely unregulated. They are not required to provide information about what is in cigarettes to the public or any federal agency. Cigarette companies do sometimes release lists of additives, but refuse to provide details, saying the ingredients are "trade secrets."

Private and government agencies have analyzed particles found in the smoke that comes off of burning cigarettes. Of the more than 5,000 chemicals that the Federal Trade Commission (FTC) found in their analysis of cigarette smoke, over 40 are known human carcinogens — chemicals proven to have caused cancer not only in lab animals, but in people. Consider this list from the FTC:

Carbon monoxide (CO) is the major component of the gas phase of cigarette smoke, accounting for 4% of the smoke of an average cigarette. It is a colorless, poisonous gas that is also found in car exhaust. In high concentrations, CO is deadly; in lower doses it causes shortness of breath and increased heart rate. CO displaces oxygen from red blood cells so that blood cannot transport enough oxygen to the body. CO aggravates lung and respiratory diseases, promotes cholesterol deposits in arteries, and impairs vision and judgment.

Nicotine is an alkaloid poison found in nature only in tobacco, and was once the leading ingredient in most insecticides. It is the powerful stimulant that causes addiction. In cigarette smoke, nicotine is absorbed through the respiratory lining and is picked up by the blood in the capillaries of the lung. Within 7 seconds of inhaling on a cigarette, nicotine reaches the brain, causing release of chemicals that increase the heart rate and blood pressure by as many as 33 beats per minute, and decrease neuron activity. Nicotine also affects parts of the body other than the brain: it increases acid secretion in the stomach, decreases urine formation, releases epinephrine from the adrenal gland (causing further increase in the heart rate and blood pressure, dilation of the pupil of the eye, and dilation of the bronchioles), and a decrease in skin temperature due to narrowing of blood vessels in the fingers and toes, reducing blood circulation in the arms and legs. Nicotine causes narrowing, or constriction, of the blood vessels and dumping of blood platelets.

Nitrosamines are chemicals formed from nicotine when tobacco is cured (preserved) or burned, as when tobacco is smoked. A known carcinogen, nitrosamines cause malignant tumors of the lung, nasal cavity, esophagus, and trachea in animals and may cause cancer in humans. Nitrosamines are also present in smokeless tobacco and are believed to be the cause of the oral cancers associated with smokeless tobacco use.

Hydrogen cyanide is toxic to the lining of the respiratory tract.

Nitrogen oxide has been associated with emphysema and other chronic respiratory diseases common in smokers.

Tar is the sticky, oily brown substance that builds up in the air passages of smokers. It causes many of the cancers associated with smoking.

Polycyclic aromatic hydrocarbons attach to the DNA (genetic material in the cell nucleus), resulting in abnormal cell division, and probably cancer.

Benzene, a carcinogen is a colorless, toxic liquid aromatic hydrocarbon (C_6H_6) commonly used as a solvent and as a motor fuel.

Polonium-210 is a radioactive isotope, a product of radon decay and lead-210. These isotopes take up long-term residence in the lungs and produce measurable radioactive hot spots. When they finally depart the lungs, they travel through the blood and urine causing cancer in other parts of the body, most notably the bladder. The radiation exposure experienced by a pack-and-a-half-a-day smoker equals what a person would be exposed to in about 300 chest x-rays, a dose sufficient to cause cancer. In addition, at least 50% of the radioactive isotopes in cigarette smoke wind up in the air and are available to be inhaled by others.

Formaldehyde is a chemical preservative, the same preservative used on the dead frogs dissected in high school biology class.

Cyanide is an ingredient found both in tobacco smoke and rat poison.

Adapted from:
Hutchinson Smoking Prevention Project. Background Information for Teachers. Seattle, WA: Fred Hutchinson Cancer Research Center. Rev. 1990.

APPENDIX 9: DEPRESSION

Background: Smoking Cessation, Depression, and Teens.
Teens with major depression are significantly more likely to become smokers. Possible reasons: (a) self- medication hypothesis, and (b) a common etiology. Teen smokers are more likely to suffer from depression and anxiety. According to Patton et al. (1996), 38% of males and 59% of females fell into the "high morbidity" category. According to Brown et al (1996), 67% of teen smokers have a history of psychiatric disorder compared to 33% of non smokers.

Among 21-30 year olds, the greater the nicotine dependence the greater the positive association with depression and anxiety disorders. Non-dependent young adults had higher rates of substance dependency, but not of major depression or anxiety disorders (Breslau, 1991).

Adults that reported depressive issues (a) have greater withdrawal symptoms, (b) are less likely to quit, and (c) are more likely to have depressive episodes following cessation (Covey et. al, 1990 and 1997). These depressive symptoms also are likely to remit with the resumption of tobacco use (Borrelli, 1996).

Conclusions
(1) A significant proportion of teen smokers in the intervention will probably have a history of either clinical depression or sub-clinical depressive symptoms. Many of these will also have other psychiatric (ADHD, oppositional defiant/conduct disorder) and substance abuse problems. Smoking cessation may trigger some increased psychiatric difficulties both in the initial withdrawal and later maintenance stages.

(2) While it is not the counselor's job to diagnose or treat depression, counselors will be alert for symptoms that last longer than two weeks (many of which may mimic withdrawal symptoms):

- overwhelming sadness, feeling blue, or irritability
- hopelessness, guilt, or apathy
- sleep disturbances
- difficulty concentrating or remembering
- diminished interest or pleasure in activities
- recurrent thoughts of death

(3) Cessation support will need to provide teens with strategies for managing dysphoric mood states and for appropriate referral sources (to be developed). In the event of pronounced and prolonged symptoms, suggest the teen see his/her family doctor who can evaluate and offer treatment options (as per Emergency Guidelines, section __.

APPENDIX 10: COPING WITH NEGATIVE MOOD

To be submitted at a later date, prior to actual use.

Schedule pleasant activities

Schedule self-soothing practices (OBT Manual)

APPENDIX 11: STRESS AND ANXIETY

Stress is your body's reaction to a danger or a demand. Causes can be minor – a bad hair day, too much caffeine, or major – your third speeding ticket or even the death of a loved one. When stress builds up and exceeds your comfort zone, the body can react in all sorts of ways: muscle tension, headache, increased blood pressure, accelerated heart rate, losing your temper, stomach in knots.

Often smokers use their smoking to manage stress, or otherwise alter their moods. Smokers who use smoking to regulate how they feel will need to find replacements. Developing new ways to relax and deal with stress can help ensure a permanent quit.

Strategies for managing stress

- Use "relaxation through deep breathing" to relieve stress. This exercise can be done just about anywhere at anytime, so smokers can use it in high stress situations. But, to learn the exercise and start practicing it daily, choose a time of day when you're comfortable with turning off the cellular phone, TV, radio, etc. Try this exercise whenever you feel stressed, even if you only have one minute – relaxing for one minute is preferable to not relaxing at all!

1. Find a timer that can be set for at least five minutes; do so, and keep it next to the chair.
2. Sit comfortably upright (slouching increases tension on the body) with your hands in your lap; your eyes may be open or closed (whichever is more comfortable). Or, lay on your back on the floor with your legs on a chair. This posture is relaxing because it takes pressure off your spine.
3. Slowly INHALE deeply through your nose (you should feel your diaphragm expand in your abdomen) while counting to four; HOLD for four, and then, as you slowly EXHALE, say, "*Relax*."
4. As you practice this deep breathing, try to focus on each part of your body, from top to bottom or randomly, as you tell yourself to relax. You may actually feel your muscles letting go. Let your thoughts wander; any attempt to "clear your mind" will likely cause tension. *Relax*. You can bring yourself back to the moment at any time by simply focusing on your breath and the word, "*Relax*."
5. Let the timer worry about how long you've been relaxing. All you need to do is breathe in and out and say, "*Relax*." When your time is up, sit for another minute and check out how your body feels.

- Try exercise to relieve stress – get out and have some fun and fresh air. This strategy has the added benefits of helping with cravings and preventing weight gain associated with quitting. (See Appendix __, Exercise)

- Talk with others about your problems; don't keep them bottled up. Ask for help.

- Set some time aside for yourself to do something special you enjoy: take a 30-minute time-out to read a magazine, take a bubble bath, or call or e-mail/IM a friend. (See Appendix __, What do I do when I have the urge to smoke?).

Adapted from:
California Smokers' Helpline. BUTTsOUT #2. San Diego, CA: California Department of Health Services. 1998.
Center for Health Studies, GHC. Fresh Start Pediatric Smoking Cessation Study Materials Notebook. Seattle, WA: Center for Heath Studies. Group Health Cooperative. July 1997.
Join Together. Quitting Guides. Trustees of Boston University. School of Public Health: Boston, MA. [On-line]. Available: www.quitnet.org; accessed 04/03/01.

APPENDIX 12: SUICIDALITY

Research has shown that the teens at greatest risk for suicide are those who have attempted suicide previously, who are currently thinking about or discussing suicide, and who have depression or are abusing drugs and/or alcohol. Because so many individuals with suicidal ideation and many who have attempted suicide go unnoticed and therefore untreated, researchers have been working on accurate and effective screening programs. It is possible, but unlikely, that counselors will encounter a suicidal teen; however, if this rare event should occur, counselors should follow Emergency Guidelines (section 6.5).

General guidelines for participants expressing suicidal ideation.

- Reassure that person that he or she does have someone to turn to. Family, friends, school counselors, physicians or teachers are probably very willing to listen and help. Reassure the person that it can be hard to know how to tell others we want to talk about something as serious as our emotions.
- Don't lecture or point out all the reasons a person has to live. Instead, listen and reassure the individual that depression and suicidal tendencies can be treated. Depressive disorders respond readily to treatments such as psychotherapy or appropriate medication. Antidepressants can act within two to three weeks and often are used in addition to psychotherapy. Nearly 90 percent of all depressed people respond to these treatments.

APPENDIX 13: FEAR OF FAILURE

Many teens may be reluctant to try to quit smoking because they fear they will fail; some may have previously tried to quit and failed. When encountering fear of failure, frame fear of failure as an indication of commitment. For instance, a counselor might respond to a teen who says, *"I just know I can't do it. Every time I try I screw up and I don't want to screw it up again,"* by saying, *"It sounds like quitting is so important to you that you don't want to be disappointed by not succeeding."* Point out to the participant that ambivalence and fears are a natural part of making any big change.

Strategies for handling fear of failure

- Encourage teens to talk about their specific concerns so that they can start taking control of them by challenging their fears/concerns.

- Point out that it is true that you can't fail if you don't try – but it is also true that you can't succeed. Use the Michael Jordan example: *"You know, Michael Jordan didn't make his High School basketball team the first year he went out, but tried again the next year and made the team. The rest is history."*

- There's no such thing as failure when engaged in a process that occurs over time and involves "trial and error" learning.

Adapted from:
Center for Health Studies, GHC. Fresh Start Pediatric Smoking Cessation Study Materials Notebook. Seattle, WA: Center for Heath Studies. Group Health Cooperative. July 1997.

APPENDIX 14: BENEFITS OF QUITTING

The immediate benefits of quitting smoking are numerous and significant. Levels of nicotine in the body decrease so that blood pressure decreases to a normal level, pulse decreases to a normal rate, and skin temperature of hands and feet increase to normal level. Carbon monoxide levels decrease within eight hours of quitting smoking. As a result, oxygen levels rise and overall energy level and stamina increase.

There is immediate improvement in respiratory function. Cilia regrow, increasing the body's ability to remove mucus. This means that the body is better able to protect itself against infection. Consequently, there will be a decrease in excess mucus production, smoker's cough, shortness of breath, colds, and sinus congestion. The sense of taste and smell improve, as does oral health. "Smoker's breath" disappears and dental health is also improved.

The long-term benefits of smoking cessation are also significant: they include decreased risk of developing cancer. There is a gradual reduction in the risk of developing lung cancer that approaches the risk of a never-smoker after 10-15 years of being quit. There is a gradual reduction in risk for cancer of the larynx, which approaches that of a never-smoker after 10 years. The risk of developing bladder cancer also decreases, approaching that of a never-smoker after about seven years.

The risk of coronary artery disease sharply decreases after one year. After 10 years, an ex-smoker's risk of this disease is the same as a never-smoker. The risk of chronic bronchitis and emphysema is reduced. There is a decrease in mucus production within the first few weeks and gradual improvement in lung function.

Women who quit smoking before the fourth month of pregnancy eliminate the risks of low birth weigh and stillbirth.

The likelihood that peptic ulcers will heal is increased. Quitting smoking promotes wound healing, for example, after surgery.

Cosmetic improvements include clean-smelling clothes and hair, clean, bright smiles, and fewer wrinkles.

Quitting smoking has benefits outside the realm of improved health. For example, ex-smokers have more money to spend on other things that they like because they are no longer spending their money on cigarettes. Quitting smoking also has a positive impact on self-confidence, self-control, and self-esteem. Finally, ex-smokers no longer need to worry about where they can smoke, or whether their cigarette smoke is harming others.

Adapted from:
Hutchinson Smoking Prevention Project. Background Information for Teachers. Seattle, WA: Fred Hutchinson Cancer Research Center. Rev. 1990.
www.cdc.gov
Join Together. Quitting Guides. Trustees of Boston University. School of Public Health: Boston, MA. [On-line]. Available: www.quitnet.org; accessed 04/03/01.

APPENDIX 15: CHOOSING A QUIT METHOD

Menu of Quit Options

Method	How it works	Availability	Pros & Cons
Cold Turkey	Just do it – quit now.	Always an option; requires only your determination, motivation, and self-control	**Pros:** Result is immediate nonsmoker status, free, saves $$, immediate health benefits, smell good, confidence and sense of accomplishment.<br>**Cons:** Withdrawal symptoms, may have problems if no planning or preparation done
Cutting Down	Gradual reduction in the number of cigarettes smoked until you're smoke-free!	Same as above, only this method takes longer.	**Pros:** Gradual adjustment to nonsmoker status; increased confidence in ability to cut down and quit; sense of accomplishment.<br>**Cons:** Emotional attachment to last few cigarettes; Not nicotine free & still a smoker until totally quit; Chance of # of cigarettes creeping back up.
Buddy System	Identify a friend or relative who also wants to quit and work on this process together.	Find a quit buddy through your social circles – family, friends, church, school . . .	**Pros:** Shared problem-solving and someone to rely on<br>**Cons:** Possible conflict or sabotage, finding someone as ready as you are to quit
Counseling	Understand how smoking relates to other issues in your life; have support for quitting	Free from *Matchbreaker* on the phone; counseling may also be available through local hospitals, clinics, or churches	**Pros:** Individual, 1:1 attention, comprehensive; use this method with other strategies for greater chance of success<br>**Cons:** If counseling is done in person, it can be expensive and require considerable time commitment; Scheduling & keeping appointments
Medical Approach	Use of nicotine replacement therapy (e.g., nicotine gum or patch) or prescription medication (e.g., Zyban) to control withdrawal symptoms while quitting	From your doctor and local pharmacy	**Pros:** Effective in helping to control withdrawal symptoms<br>**Cons:** Only proven to work when used along with counseling; Not yet demonstrated to be effective in helping smokers under 18 yrs of age; Possible side effects; May require Doctor's visit and prescription.

Method		How it works	Availability	Pros & Cons
	Support groups or cessation classes	Educate you about health risks, other aspects of smoking and how to quit, provides shared discussion and support from others trying to quit	Find from the phone book; may be available through health dept, local hospitals, churches, schools, or American Lung, Heart and Cancer Societies	**Pros:** Education, support, fellowship, learning from others **Cons:** Not individual to suit your specific needs
Hypnosis		Reaching a deep state of relaxation and openness to suggestions about quitting	Requires licensed hypnotist	**Pros:** Relaxing, non-invasive **Cons:** Unproven, Giving up control; very expensive; need good hypnotist, return visits / scheduling/ transportation
Acupuncture		Fine needles are inserted into various body points to block receptors	Requires licensed acupuncturist	**Pros:** Natural, alternative medicine; **Cons:** Unproven; Uses needles; Very expensive; need good acupuncturist; Return visits/ scheduling / transportation

Adapted from:
California Smokers' Helpline. BUTTsOUT #1. San Diego, CA: California Department of Health Services. 1998.

APPENDIX 16: BOOSTING SELF-EFFICACY

To be submitted at a later date, prior to actual use.

APPENDIX 17: WHAT DO I DO WHEN I HAVE THE URGE TO SMOKE?

Alternatives to Smoking

Drink water
Walk the dog
Go skateboarding
Review your list of reasons for quitting
Call a friend
E-mail or IM a friend
Do deep breathing
“Urge surf”
Brush your teeth
Take a bubble bath
Rent a video
Suck on hard candy or cinnamon sticks
Chew gum
Take a walk
Read a magazine or book
Clean your room
Go to a movie
Shoot baskets
Ride your bike
Go shopping
Organize a potluck party
Practice the 5 D’s (delay, distance, deep breathe, drink water, distract)
Play video games
Listen to music
Write a poem, draw a picture, be creative!
Do your homework
Go someplace where you can’t smoke (e.g., the library, the movies)

APPENDIX 18: EXERCISE

Exercise can be a big help to people who want to quit smoking, relieve stress, and/or control their weight. It is also a natural anti-depressant because exercise releases endorphins in the brain that cause people to feel good. It need not be strenuous to be helpful, and any exercise beats no exercise at all. Pick something that is fun, that you enjoy doing, and you'll be more likely to stick with it. Having a friend or buddy to exercise with can also add to the fun and make it more likely you'll do it often.

Exercise Options

Take a walk
Put on some music and dance
Go skateboarding
Do resistance training (weight lifting)
Ride your bike
Work out with an exercise video or class
Walk the dog
Play Frisbee with a friend (2-legged or 4-legged)
Go swimming or take water aerobics
Play water polo
Play volleyball
Shoot hoops
Take an aerobics or exercise class
Jump rope
Play catch
Play soccer in the park
Learn Kung Fu
Do Kick-boxing
Go dancing – or dance in the living room to your favorite CD
Mow the lawn
Sweep the driveway
Wash the car
Use a stationary bike or treadmill
Play badminton
Take tennis lessons
Go jogging
Play baseball, softball
Play touch football
Always take the stairs
Go rollerblading
Ski – cross country or downhill
Go bowling
Speed walk
Learn to ride a unicycle

APPENDIX 19: HEALTHY SNACKS

When trying to quit smoking, it's often helpful to have snack substitutes for cigarettes, but don't replace smoking with pigging out! Instead, try some of these healthy strategies when quitting smoking.

• Eat regular meals; don't just eat whatever or whenever you feel like it. Keys are to eat a balanced diet and include lots of variety in your choices.

• Drink extra water, at meals and in-between. Also, cut out the caffeine; choose caffeine-free soda [Sprite, 7-Up] and coffee drinks. Also, if you like lattes or mochas, try ordering your drink decaf and fat-free or 2%.

• Choose low-fat snacks and sweets, those with 4 grams of fat or less per serving. Try putting individual-size portions of snacks aside each day in a plastic zip-lock bag. But, remember to be sensible – low-fat foods have calories, too.

When you want a snack, try some of these healthy (low fat) and yummy choices:

**Crispy, low fat snacks**:	**Fruits & Vegetables**:	**Sweets & Desserts**:
Pretzels	Raw or canned fruit	Fig bars, Raspberry bars
Light microwave popcorn	Fruit juices	Gingersnaps
Dry cereals	Vegetable juices	Vanilla wafers
Ak Mak crackers	Splash!	Animal crackers/cookies
Crackle or crispbread	Dried apricots or apples	Fat-free cookies and cakes
Melba toast	Raisins	Hard candy (lifesavers, sour balls)
Saltines	Carrot sticks or baby carrots	Jelly beans
Honey graham crackers	Celery sticks	Angel food cake
Cinnamon graham crackers	Green & red pepper strips	Licorice
Chocolate graham crackers<br>Doritos light corn chips	Fresh veggies with low-fat dip or fat-free dressing	Teddy Grahams<br>Gum drops
Low-fat Wheat Thins	Del Monte fruit cups	Low-fat pudding cups
Rice cakes	Frozen grapes or bananas	Fruit & Granola bars
Reduced-fat Ruffles	Applesauce	
**Breads:**	Salsa	**Frozen Desserts**:
Bagels	**Chocolate**:	Frozen yogurt
English muffin	Hot cocoa	Sherbet
French bread	Hershey's chocolate syrup	Sorbet
Pita bread	Tootsie Rolls	Light Ice Cream
Tortillas	Low-fat chocolate pudding	Fudgesicles
Bread sticks	Fudgesicles	Fruit juice bars
Whole wheat or rye bread	Chocolate wafers	Popsicles

(Snack ideas courtesy of the Women's Health Initiative (WHI), Fred Hutchinson Cancer Research Center)

APPENDIX 20: HANDLING CONCERNS ABOUT WEIGHT

Concerns about weight gain are not uncommon.
Many people, especially women, express concern about gaining weight when quitting smoking. This is not an unusual concern, given the societal messages women receive about being slender and attractive.

Unfortunately, weight concerns can lead to a lower commitment to quit smoking, but from a health perspective added weight is not as bad for you as smoking. In fact, a smoker would have to gain more than 85 pounds to offset the health advantages of quitting smoking a pack a day.

How much weight do smokers gain when quitting?
The truth is that weight gain while quitting smoking is not inevitable, but it is not unusual either. Most people who do gain weight gain 5-8 pounds. Women, African-Americans, heavy smokers (25+ cigarettes a day), and people under age 55 have a higher risk of gaining weight when quitting.

Also, you have to remember that there's this "urban myth" about smokers being thin. All smokers are NOT thin – just go to the mall if you want to see the evidence!

Why do people gain weight when quitting smoking?

Most weight gain results from increased snacking – not only does food seem more appealing due to the re-awakened sense of smell and taste, but snacking also provides an alternative to smoking. There is no reason to avoid snacking, just dump the Twinkies – be selective when choosing snacks. (See Appendix 17. Healthy Snacks.)

People also gain weight because of cigarettes' drug and chemical effects on the body. For instance, nicotine is a stimulant that speeds up a smoker's metabolism, causing the body to run faster and burn more calories than normal. When a smoker quits, nicotine intake is withdrawn and the smoker's metabolism returns to a normal rate. This can temporarily contribute to weight gain for some people. Also, the stimulant properties in nicotine's decreases the sensation of hunger while the carbon monoxide in the smoke interferes with the senses of smell and taste making food less enjoyable. All of these factors also make weight gain more likely when a person quits, but they are all manageable.

What's the best way to control weight while quitting smoking?

Exercise can offset the tendency to gain weight in several related ways. It naturally speeds up the bodies' metabolism and burns extra calories. These benefits continue for up to two hours after exercise is discontinued. At the same time, exercise decreases appetite resulting in fewer stored calories. In addition to exercise, drinking lots of water, and eating well-balanced meals and healthy snacks will also help with the goal of quitting smoking, as well as with controlling weight.

If you make quitting smoking your #1 priority now, you can start to take off any extra weight once you are a secure nonsmoker. A sure-fire way to make quitting smoking difficult is to try to diet at the same time. If you are hungry and on a diet, you are going to want a cigarette – food deprivation

leads to increased desire for nicotine. So, plan ahead and have snacks on hand. And if you are concerned about weight gain, eat sensibly but don't overdo it!

What other strategies can I use to control my weight while quitting smoking?

- **Don't go on a strict diet.** Research has shown that going on strict diets can undermine your attempts to quit smoking and usually fail to produce long-term weight reduction.

- **Monitor your weight.** Weigh yourself once a week – often enough to keep tabs on how you're doing but not so often that you become obsessed about your weight.

- **Know what you eat.** Avoid getting into the habit of managing urges and cravings with high calorie snacks. If you are having strong urges and cravings to have something in your hands or mouth, try the following: (1) Use sugarless gum, carrots, and celery; (2) Find things to keep your hands busy that do not involve food, e.g., carry tooth picks or paper clips to play with, cut straws into cigarette sized pieces, or carry a worry stone or small rubber squeeze ball.

- **Slow down your meals**. Many smokers have developed the habit of rushing through meals so they can smoke. After they have quit, they will still eat as fast and often use that "extra" time at the end of the meal to have seconds. Here are some possible solutions:
  - Have someone else portion your food.
  - Put away extras immediately
  - Slow your eating by cutting food into smaller pieces, putting your fork down after every bite, drinking water while you eat, get up from the table as soon as you are done, brush your teeth right after the end of the meal.

- **Do not skip meals**. Skipped meals are "made up for" by gorging.

- **Exercise regularly**. Remember, exercise doesn't have to be strenuous to be effective – go walk the dog! Exercise also helps relieve stress and reduces urges and cravings.

Adapted from:
Join Together. Quitting Guides. Trustees of Boston University. School of Public Health: Boston, MA. [On-line]. Available: www.quitnet.org; accessed 04/03/01.

APPENDIX 21: PREPARING TO QUIT

Being well prepared before you quit is crucial to your success. Often people give little consideration to what quitting will be like, so they are ill-prepared to handle the challenges associated with quitting smoking. You wouldn't go hiking without first preparing – e.g., wearing appropriate clothing and shoes, or bringing a full water bottle. Similarly, with a little preparation, you can make quitting smoking a lot less difficult.

Prepare Your Emotional Self

Make a list of YOUR reasons for quitting (problems with smoking/benefits of quitting)
Plan activities to keep busy, especially the first day
Have a worry stone, rubber squeeze ball, or other objects to occupy your hands
Argue against the cigarette ads
Argue against the "one cigarette won't hurt me" thoughts
Begin to visualize yourself as a non-smoker
Identify your smoking "triggers," i.e., things you do that always make you light up, and develop a list of strategies for managing them
Anticipate challenges to your quit attempt, particularly during the first two weeks
Re-read the self-help materials sent by the telephone counselor — know your plan!

Prepare Your Physical Self

Reduce your caffeine use
Get lots of rest
Drink plenty of fluids, especially cold water – it helps flush the nicotine from your system and helps ward off withdrawal symptoms including urges and cravings to smoke.
Have cigarette substitutes, a.k.a. "mouth toys." on hand (e.g., cinnamon sticks, lifesavers and other hard candies, chewing gum, carrot sticks, plastic drinking straws cut to the size of cigarettes)
Practice your deep breathing exercise
Learn about withdrawal and develop a list of strategies for coping with withdrawal symptoms
Learn skills for quitting, e.g., learn to urge surf; most urges and cravings pass in 3 to 5 minutes. Also, learn and practice the Five D's (i.e., deep breathing, drink water, distract, distance, delay).
Get a new haircut; you'll feel better!
Re-read the self-help materials sent by the telephone counselor

Prepare Your Surroundings

Make a clean start!
Throw away all your cigarettes – even those stashed away in coat pockets, backpacks, purses and car glove compartment boxes.
Remove all smoking stuff from your room/house, including ashtrays, lighters, and matches
Refuse to be a walking billboard for the tobacco companies — Put away all clothing, baseball caps, etc., with cigarette logos
Clean out your car — Empty and clean the ashtray, vacuum the carpets and seats, wipe down the windows and the dashboard. If it still smells like smoke, get a car deodorizer.
Get room deodorizer if your room smells like smoke
Get your clothes cleaned – don't forget your coat or jacket – so that they don't smell like smoke.

You may not be able to smell it now, but you will when you quit.
Avoid smoking in places where you spend a lot of time, particularly after you've cleaned them up in preparation for the quit attempt. Decide not to smoke indoors.
Get support from others for your quit attempt, particularly from smoking members of your household.

Adapted from:
Join Together. Quitting Guides. Trustees of Boston University. School of Public Health: Boston, MA. [On-line]. Available: www.quitnet.org; accessed 04/03/01.
Zhu S-H, Tedeschi GJ, Anderson CM, Pierce JP. Telephone counseling for smoking cessation: What's in a call? Journal of Counseling & Development, 1998;75:93-102.

APPENDIX 22: ASKING FOR AND GETTING SUPPORT FOR QUITTING

When making a big change such as quitting smoking, it can be helpful to have support from important others in your life. Other people will influence your quitting experience whether you like it or not, so why not take some control over their influence? Let them know what kind of help you'd like.

Think about the people closest to you – your friends, family, co-workers – identify who might be willing to help you in your efforts to quit. Enlist allies to help you. You'll probably get lots of support and positive reinforcement from your nonsmoking friends and family. A bigger challenge is family and friends who smoke – your quitting smoking may not be as important to them as it is to you. Also, they may not realize that their smoking in front of you can hurt your effort to quit. Planning ahead can help you get the support you'd like from others.

Strategies for obtaining support from friends and family, even those who smoke

Tell friends & family of your intentions to quit and ask in advance for their help and patience

Make agreements with friends and family who smoke not to smoke around you. Ask them not to give you a cigarette, even if you ask for one.

Ask smokers in your household if they will help by smoking less (or even not at all) in the home. If family members are reluctant stop smoking in the house accept their decision. Instead, create a smoke-free area at home for yourself – your bedroom, the bathroom, the TV room.

Stay away from smokers whenever possible, especially in the first few weeks after you quit.

Be assertive in asking for what you need. Prepare for some backlash after you quit. Stand by your decision not to smoke and give a reason *("I'm done with that." "No more smoking; I want to get in shape." "I've already made it three days, I don't want to blow it now.")*

Adapted from:
California Smokers' Helpline. BUTTsOUT #2. San Diego, CA: California Department of Health Services. 1998.
Center for Health Studies, GHC. Fresh Start Pediatric Smoking Cessation Study Materials Notebook. Seattle, WA: Center for Heath Studies. Group Health Cooperative. July 1997.

APPENDIX 23: NICOTINE REPLACEMENT THERAPY AND PHARMACOTHERAPY

General Approach:

Answer students' questions about nicotine replacement therapy (NRT) or other pharmacotherapy (e.g., Zyban) accurately. Point out that these therapies have been shown to be effective only with older adult smokers and only in conjunction with cessation counseling. Pharmacotherapies help relieve withdrawal symptoms; they are not smoking cessation's "magic bullet." Research has demonstrated that pharmacotherapies may be useful for smokers who are enrolled in a cessation program, smoke a pack a day or more, smoke within half an hour of waking up, and suffer withdrawal symptoms severe enough to interfere with quitting.

Smokers who choose to use the nicotine patch or gum MUST NOT CONTINUE TO SMOKE while doing so. Smoking while using NRT can lead to nicotine overdose and prevent successful quitting.

To date, only a few studies have tested the safety and efficacy of pharmacotherapy used with adolescent smokers. Those studies failed to demonstrate that pharmacotherapy is an effective treatment with teenaged smokers. The studies have demonstrated that NRT is safe for use with teens. Additional studies of pharmacotherapy for adolescent smoking cessation are currently underway.

Despite the very limited efficacy data on pharmacotherapy with adolescent smokers, teen smokers who are addicted and experience serious withdrawal symptoms may benefit from NRT in conjunction with the HS Study telephone counseling. If the smoker is assessed as dependent [using modified Fagerstrom items, i.e., student has the urge to smoke "upon waking" or "within ½ hour of waking"], smokes a pack a day or more, and has a history of previous quit attempts accompanied by withdrawal symptoms, counselors may elect to discuss NRT from the menu of quit options [Appendix __] with the smoker. *Note*: Nicotine patches do not require a prescription for adults age 18 years and older, therefore, study participants who are 18 or older may purchase the patch for themselves. Younger students will need to ask their parents to purchase the nicotine patch for them, or will need to talk to their physician and get a prescription for the patch. Students considering using the nicotine patch will be strongly advised against smoking while using the patch.

Frequently Asked Questions about Pharmacotherapy for Smoking Cessation

Who should receive pharmacotherapy for smoking cessation?

Current treatment guidelines recommend that all smokers trying to quit, except in the presence of special circumstances, receive pharmacotherapy. Several special populations are excluded from the recommendation: those with medical contraindications, those smoking fewer than 10 cigarettes/day, pregnant/breastfeeding women, and adolescent smokers.
Note: To date, clinical trials have not demonstrated that pharmacotherapy is effective for adolescent smokers.

What are the pharmacotherapies recommended in the current guidelines for treating tobacco use and dependence?

There are currently five FDA-approved pharmacotherapies for smoking cessation therapy recommended for use in treatment guidelines: sustained-release bupropion hydrochloride (“Zyban”), nicotine gum, nicotine inhaler, nicotine nasal spray, and the nicotine patch.

Are pharmacotherapies appropriate for lighter smokers (10-15 cigarettes/day)?

If pharmacotherapy is used with lighter smokers, clinicians should consider reducing the dose of first-line NRT therapies. No adjustments are necessary when using sustained-release bupropion hydrochloride.

Can taking Zyban help me quit smoking?

Zyban has been found helpful for long-term, heavy smokers when used in conjunction with counseling for smoking cessation. There is no evidence yet that it helps more recent younger smokers, so we can’t recommend that you try it. Because it’s a prescription medication, your doctor may be able to answer more questions about Zyban for you. However, if you take any kind of medication to help you quit smoking, you will probably be more successful if you also participate in the Matchbreaker program, than if you just take the medication by itself.

Can using the patch (nicotine gum) help me quit smoking?

Nicotine replacement products like the patch and gum have been found helpful for long-term, heavy smokers when used in conjunction with counseling for smoking cessation. There is no evidence yet that these aids help more recent, younger smokers who are less dependent on nicotine, so we can’t recommend that you try them. However, if you think these products might help you to quit, we recommend that you talk to your parents because the sale of nicotine-replacement products are limited to people who are 18 and older. If your parents know you are making a serious quit attempt, they may be willing to buy the patch or gum for you. Also, your doctor may be able to answer more questions about nicotine replacement therapy for you. If you do decide to take any kind of medication to help you quit smoking, remember that you will probably be more successful if you also participate in the Matchbreaker program, than if you just take the medication by itself.

Information about pharmacotherapies used for smoking cessation

Pharmacotherapy	Contraindications	Adverse Effects	Availability	Cost/day
Sustained-release bupropion hydrochloride ("Zyban")	Hx seizures<br>Hx eating disorders<br>Hx Bipolar Illness	Insomnia<br>Dry mouth	Prescription only	$3.33
Nicotine gum		Mouth soreness<br>Dyspepsia	Over-the-counter	$6.25/10 2mg pieces (1 ppd smoker requires up to 24 pieces/day)<br>$6.87/10 4mg pieces
Nicotine inhaler		Irritation of the mouth and throat	Prescription only	$10.94/10 cartridges (requires 6-16 cart. per day)
Nicotine nasal spray		Nasal irritation	Prescription only	$5.40 for 12 doses (requires 8-40 doses per day)
Nicotine patch		Local skin reaction, Insomnia	Prescription and over-the-counter	$4.50

<u>Adapted from</u>:
Clinical Practice Guideline for Treating Tobacco Use and Dependence, 2000.)

APPENDIX 24: WITHDRAWAL AND RECOVERY SYMPTOMS

Understanding the changes your body undergoes when you are quitting smoking is the first step towards dealing with them. Most of the time we refer to all of the discomforts as withdrawal symptoms, but some of the changes you notice are really symptoms of recovery. Identifying these symptoms can help you cope with them if and when they occur.

Withdrawal Symptoms
Withdrawal symptoms are feelings that are due to your body's need for nicotine and your mind's need for cigarettes. These feelings are natural and almost all smokers go through them. The good news is that withdrawal symptoms generally don't last long. Common withdrawal symptoms include:

Craving. This is the most common and most familiar withdrawal symptom. Craving is your body's physical addiction calling out. Just keep in mind that most cravings last less than 30 seconds. In general, most cravings stop altogether after just one week.

Difficulty concentrating. Nicotine is used by many people to help focus their attention. After awhile, nicotine becomes a crutch. Therefore, it can take smokers up to a couple of weeks to become accustomed to working without nicotine. Additionally, the increased (healthy) blood flow and oxygen levels can initially feel like a feeling of mental "fogginess." Nicotine actually seems to affect people's concentration by decreasing peripheral vision and hearing. If sounds seem louder, or you seem to notice people walking by while you do homework, it's probably just that you notice these things more now without the numbing effect of nicotine.

Fatigue. Nicotine increases a smoker's metabolism to an abnormally high rate. When you stop smoking, your metabolism drops back to normal and you may find that your energy level drops. This feeling of fatigue is normal, and it will go away as your body adjusts to "normal life."

Recovery Symptoms
Recovery symptoms are evidence that your body is healing itself from the damage done by the nicotine and tar in cigarettes. The overall feeling can be a bit like having a cold. These symptoms generally last only a few days, but don't worry – this is your body getting better! Common recovery symptoms are:

Headaches, dizziness, tingling in the hands and feet. When you quit, blood vessels all over your body open back up. Increased blood flow to the cerebellum (in your brain) can cause headaches. Increased blood flow to the fingers and toes can cause a tingling feeling (like after your foot falls asleep). Dizziness can result from both lower blood pressure and an increase in oxygen to the nerves and tissue as poisonous carbon monoxide is eliminated from your blood system and is replaced by oxygen.

Sore throat, coughing. Nicotine paralyzes the cilia responsible for keeping your respiratory system clean. Many smokers have a morning cough as these defenses come back "online" after not smoking all night. In the same way, when some people quit smoking, they will cough as their lungs begin to clean themselves out. A sore throat may result from development of new

tissue and the clearing of tar and nicotine from old tissue.

Hunger. To pay for all of this recovery, your body needs energy and materials to rebuild. Therefore, you may feel a craving for snacks, especially sweets. Sticking to fruits and fruit juices and other low-fat snacks is better for increasing your energy level and for avoiding weight gain.

Adapted from:
Join Together. Quitting Guides. Trustees of Boston University. School of Public Health: Boston, MA. [On-line]. Available: www.quitnet.org; accessed 04/03/01.

APPENDIX 25: TRIGGERS

Smoking is a behavior that is learned over a period of years. If you've been smoking for a few years, smoking has probably become associated with other things that you do, like drinking coffee, partying with friends, or driving in the car. These other events become "triggers," situations that prompt you to light up. Identifying your triggers, and ideas for coping with them, will become an essential part of your plan to quit.

To identify your triggers, keep track of your smoking for week. Every time you smoke a cigarette, write down the time (e.g., 11:30 a.m. or lunchtime), the situation (where you were and what you were doing – e.g., "sitting in the parking lot after lunch), your mood (e.g., "feeling relaxed"), and who was with you. At the end of the week, take a look at your list. What do the situations have in common? Are there certain times of day you smoke? Or do you smoke only when you're with certain people? On the same list, write down some things you could have done in each instance instead of smoking.

Look at the list again. Pick out and put a star by the three situations on your list that will be the most difficult to cope with after you quit. By keeping a record of your triggers and how you can deal with them, you identify possible difficult situations before they occur

Ideas for managing triggers

**Triggers**	**Avoid the situation**	**Change the situation**	**Choose a substitute**
Smoke while driving	Don't drive – catch a ride with a non-smoking friend or take the bus	Take the ashtray and lighter out of your car; don't let your friends smoke in your car	Sing along with the radio; Carry plastic straws cut to the length of cigarettes to occupy your hands and mouth
Smoke while talking on the phone	Make phone calls from places where you don't usually smoke	Try walking around while you talk; Hold the phone with your other (cigarette) hand	Keep something in your free hand when you're talking – a pen, a small squeeze ball, a paperclip; Doodle
Smoke when drinking coffee	If you're in a restaurant, sit in the nonsmoking section;<br>Instead of coffee, drink a soda or juice	Drink your coffee standing up; Hold your mug with your other (cigarette) hand; or try drinking it with a straw	Chew gum or hard candy, suck on a tootsie-pop, or munch on some carrot sticks

Remember, the best ideas for coping with triggers and urges are the ones you come up with yourself.

Adapted from:

California Smokers' Helpline. BUTTsOUT #2. San Diego, CA: California Department of Health Services. 1998.

www.quitnet.org

APPENDIX 26: RELAPSE PREVENTION

Many people quit smoking. A famous quote often (mis)attributed to Mark Twain got it right, *"Giving up smoking is easy. I've done it hundreds of times."* The hard part is staying quit. It is possible that you'll smoke after you quit. Therefore, it's smart to be prepared for this situation, whether you believe it will happen or not. Knowing how to keep a slip from turning into a relapse is like having a good emergency plan: You may never have to use it, but it's there if you need it.

So, what do you do if you slip up and have a cigarette?

Realize you messed up. It's okay to feel disappointed. But don't beat yourself up over it – this is just a slip-up, it's not the end of the world. Use this situation to your advantage and learn from it.

Look at the situation in which you smoked. Was the slip due to the difficulty of the situation and poor coping strategies? Or was low motivation to blame?

If poor coping was to blame, think about the situation. What could you do in the future to prevent a slip in a similar situation? Revise your quit plan accordingly. If low motivation was to blame, review your reasons to quit. Write down the most important reason and put it where you'll see it as a reminder.

Think of how long you'd already gone without smoking. Remind yourself that this is just a slip, and that it doesn't mean you've failed in your quit attempt. Tell yourself, *"It was just a slip and I learned something from it. Now I'm closer to my goal of quitting for good."*

One cigarette does not make you a smoker again. One cigarette didn't make you a smoker in the first place, and one slip now doesn't mean you aren't a non-smoker. Just because you had one cigarette doesn't mean you have to have another. A slip is not in any way an excuse to smoke again. Get right back into your quit plan. Don't let one slip derail you from reaching your goal.

What's the alternative?

Relapse. After smoking that cigarette, you could give in to negative self-talk. So, you felt bad after smoking that cigarette after going so long without. You start to blame yourself and tell yourself things like, *"I'm a failure. This shows I can't quit."*

This kind of thinking leads to giving up. You think, *"I've already blown it so I might as well smoke"* and before you know it, you're back to your old smoking pattern and feeling worse than you did when you smoked that one cigarette.

Be fair to yourself instead. Don't do something you really don't want to do, just because of one mistake! Don't give up on your plan to quit!

Lots of successful quitters suffered slips, even full-blown relapses, on their way to quitting for good. In fact, it takes the average smoker 7 times to quit for good. Try to identify what went wrong and give it another shot. You can do it. Believe in yourself. You're worth it!

Adapted from:

Zhu S-H, Tedeschi GJ, Anderson CM, Pierce JP. Telephone counseling for smoking cessation: What's in a call? Journal of Counseling & Development, 1998;75:93-102.

California Smokers' Helpline. BUTTsOUT #2. San Diego, CA: California Department of Health Services. 1998.

Join Together. Quitting Guides. Trustees of Boston University. School of Public Health: Boston, MA. [On-line]. Available: www.quitnet.org; accessed 04/03/01.

APPENDIX 27: SAMPLE FOLLOW-UP LETTERS FROM COUNSELORS TO PARTICIPANTS

Sample A: First letter in follow-up to initial call (to student who reported a self-initiated quit attempt)

Student Smoker (letter #1)
123 Camel St.
Anytown, WA 98110

Hi, Student:

Thanks for taking the time to talk the other night. I enjoyed talking with you, and hearing about your recent efforts to give up smoking.

I think it's great you've decided to do this for yourself, and to go on to college smoke-free.

You mentioned things that have helped you ride out the cravings so far - like giving your smokes away, and chewing sunflower seeds. Whatever works! Check out this magazine - it may have some other ideas that could help you out. I like the list in the "More How" section, about the 2Rs and the 4Ds – it's just another way to think about stuff you can do besides smoking.

I hope you get a chance to look it over, and see if any of it is useful for you. I'll be interested to hear any feedback you have. I'll call you on Wednesday, November 7, around 6:30pm. You can call me Monday-Thursday evenings at 1-866-524-0235.

Hang in there! Talk with you soon.

Sample B: Second letter to same student in follow-up to second counselor call

Student Smoker (letter #2)
123 Camel St.
Anytown, WA 98110

Hi, Student:

Thanks again for taking time to talk the other night.

Let me start off by saying – Way To Go! I am glad that quitting is going so well for you this time. Your strategy of distancing yourself from smoking situations is working well. And getting your friends to quit with you is a great idea – it gives you support and helps them too.

You have found some great ways to help yourself – nice work.

Hang in there. I'll call you in a couple weeks.

APPENDIX 28: SMOKELESS TOBACCO

Smokeless tobacco is the term applied to the various tobacco products that are not burned when used. The most popular types of smokeless tobacco are **moist snuff** and **chewing tobacco**. Both chewing tobacco and moist snuff may be sweetened or flavored. For example, many of the moist snuff products come in flavors like Wintergreen and Cinnamon. Users either swallow or spit out the additional saliva generated from sucking or chewing the tobacco "quid."

Moist snuff is ground, loose tobacco, packaged in small tins (e.g., Skoal, Copenhagen). It can also be purchased in sachet form, where the tobacco is pre-packaged in neat little bundles (e.g., Skoal Bandits) for use by beginners — also known as children. Moist snuff is "dipped" from the can and placed between the cheek and gums.

Chewing tobacco comes in ***plug***, ***twist***, and ***loose leaf*** forms and is placed between the cheek and teeth.

Another form of smokeless tobacco is d**ry snuff,** a finely powered form of tobacco that is inhaled through the nostrils. Dry snuff use in the U.S. is rare, but it is widely used in Great Britain and many Third World nations.

Who uses smokeless tobacco?
Smokeless tobacco was very popular in the early American settlements among both men and women. With the development of the germ theory in the 1880s, scientists realized that the germ that causes tuberculosis could be transmitted in the air by spitting. This knowledge, combined with the development of a new cigarette manufacturing machine, resulted in the increased use of cigarettes and a decline in the use of smokeless tobacco.

Use of smokeless tobacco has continued, primarily among men with occupations that prevented them from smoking on the job, e.g., logging, mining, farming. Also, in some very rural areas of the Southern U.S., it is not uncommon for both men and women to still use smokeless tobacco. Motivated by a potential new market – youth – tobacco companies increased their promotion of smokeless tobacco products in the 1980's. New major advertising campaigns, often directed at young people, were begun at that time; many of these implied that the use of smokeless tobacco is a safe alternative to smoking. (Currently, U.S. Tobacco, a major manufacturer of smokeless products, is petitioning the Federal Trade Commission for the right to claim that smokeless tobacco is safer than cigarettes because it does not put users at risk for lung cancer.)

As a result of these marketing efforts, the use of smokeless tobacco increased by 65 percent between 1978 and 1990. Total sales are over $1 billion a year, of which youths under 18 consume $3 million worth, or 26 million containers each year. A 2000 study revealed that 18 percent of high school students (29% of boys and 6.8% of girls) had used smokeless tobacco in the previous year. Researchers have found that the use of smokeless tobacco among young people is most likely to occur as a result of peer use rather than parental use.

Is smokeless tobacco a safe alternative to cigarettes?
No. While smokeless tobacco use does not present a risk of lung cancer, its use does have associated health hazards. In the short term, the effects are similar to those for smoking: an increased heartbeat, higher blood pressure, a drop in skin temperature, an increase of acid released into the stomach, a slowing of stomach emptying, and a decrease in urine formation. For the long term, the risks include cancers of the mouth, lip, throat, pharynx and esophagus.

What's in smokeless tobacco?
More than 2,500 chemicals have been identified in processed tobacco. Those important in producing disease include nicotine, nitrosamines (a known carcinogen), polycyclic aromatic hydrocarbons (which cause abnormal cell division and, possibly, cancer), and polonium-210 (a radio-active isotope and known carcinogen).

How does nicotine get into the blood of smokeless users?
Nicotine in smokeless tobacco is absorbed into the blood through the lining of the mouth, the oral mucosa. It is absorbed very rapidly and the blood level of nicotine in chewers and dippers is as high or higher than that of smokers. (*Note:* Tobacco companies purposely “doctor” their products to facilitate absorption of nicotine by the oral mucosa. Nicotine is a weak base. In its ionized form, as in the acidic environment of most cigarette smoke, nicotine crosses membranes poorly. As a consequence, there is almost no oral absorption of nicotine from cigarette smoke. In contrast, smokeless tobacco manufacturers buffer their products to an alkaline pH that facilitates absorption. This means that without this “product-doctoring” by the manufacturers, nicotine in smokeless tobacco would not be absorbed by the oral mucosa and smokeless tobacco would not be an addictive product.)

People who use oral smokeless tobacco, particularly those who chew tobacco, generate large amounts of saliva, some of which is swallowed. Nicotine in the swallowed saliva passes through the lining of the intestines and enters the blood.

How does nicotine affect smokeless tobacco users?
Nicotine is a highly addictive stimulant that makes it difficult to quit smokeless tobacco. Withdrawal from the nicotine in chewing tobacco produces the same symptoms as withdrawal from cigarettes, including irritability, anxiety, lethargy, increased appetite and craving for sweets, nausea, headache, constipation or diarrhea, aggressiveness and hostility. Craving for tobacco is the most common withdrawal symptom. Once in the blood, one-fourth of the nicotine goes directly to the brain, causing release of chemicals that increase the heart rate and blood pressure. The remainder is carried by the blood to the rest of the body where it produces effects including an increase in acid secretion in the stomach, a decrease in urine formation, a release of epinephrine from the adrenal gland (causing further increase in heart rate and blood pressure, dilation of the pupil of eye and dilation of bronchioles), and a decrease in skin temperature (due to narrowing of blood vessels in fingers and toes). In addition, nicotine causes narrowing (constriction) of arteries and clumping of blood platelets. Nicotine may cause damage to the lining of blood vessels allowing increased deposition of fats in the artery walls leading to atherosclerosis.

What are nitrosamines?
Nitrosamines are chemicals formed from nicotine when tobacco is processed and preserved. Additional nitrosamines are formed as saliva mixes with the nicotine in the tobacco as it is chewed.

Are nitrosamines harmful?
Very. Nitrosamines are the most prevalent carcinogens found in smokeless tobacco and are thought to be responsible for oral cancers associated with smokeless tobacco use. Also found in cigarette smoke, nitrosamines cause cancers of the lung, nasal cavity, esophagus, and trachea in animals.

Nitrosamine and nicotine levels in smokeless tobacco products vary by brand. Copenhagen (the most popular U.S. brand) and Red Man Moist Snuff have the highest nitrosamine and nicotine levels of all products distributed in the U.S.

Don't you have to use smokeless tobacco for twenty years to have any of these diseases?
Unfortunately, no. Many of the symptoms of harmful effects, like leukoplakia, or sores in the mouth can be detected in just the first few years of tobacco use. Smokeless tobacco use has been known to cause cancer, even fatal cancers, in young people. The most well-known example is the case of Sean Marsee. Sean was a 17-year-old high school track star who developed tongue cancer from using chew. Despite multiple (disfiguring) head and neck surgeries, and other aggressive therapy, Sean died of his cancer shortly after his 19th birthday. It is through the efforts of his family that warning labels, like those on cigarette packages, now appear on smokeless tobacco products.

Adapted from:
Hutchinson Smoking Prevention Project. Background Information for Teachers. Seattle, WA: Fred Hutchinson Cancer Research Center. Rev. 1990.
USDHHS, The Health Consequences of Using Smokeless Tobacco: A Report of the Advisory Committee to the Surgeon General, 1986. USDHHS, PHS, April, 1986, Bethesda, MD. NIH Pub. No. 86-2874.

APPENDIX 29 CLOVE CIGARETTES

Clove cigarettes, also known as “jarms” or “kreteks,” have been imported from Indonesia since 1968. Since about 1980, they have gained increasing popularity on the West Coast, the majority being purchased by persons between the ages of 17 and 30 years.

Are clove cigarettes a safe alternative to regular cigarettes?
No. Many people believe clove cigarettes to be a safe, low-tobacco alternative to regular cigarettes. In fact, clove cigarettes contain approximately 60 percent tobacco and 40 percent cloves. ***Clove cigarettes contain almost twice as much tar and nicotine as regular cigarettes***. This means that clove cigarette smokers are at increased risk of developing cancers of the lung, mouth, larynx, esophagus, pancreas, and bladder, as well as heart disease and emphysema.

Are the cloves in clove cigarettes harmful?
Yes. In addition to the harmful substances found in tobacco, clove cigarettes contain the chemical *eugenol* (from the cloves). Eugenol is a weak anesthetic and may make the initial stages of smoking easier by soothing the throat. Researchers believe that this effect may encourage smoking by persons who might otherwise be discouraged by the harshness of regular cigarettes.

A variety of scientific studies and medical cases indicate that when burned (such as when clove cigarettes are smoked), eugenol produces some unique, toxic effects which can be so severe as to be life-threatening: pulmonary edema (blood fluid in the lungs), bronchospasm (constriction of air passageways), coughing up blood, nausea and vomiting, increased evidence of respiratory tract infections, worsening of chronic bronchitis, and increased incidence and severity of asthma attacks. The highly-publicized death of 17-year-old Tim Cislaw of California has also been linked to clove cigarettes.

Eugenol is documented to be toxic to cells and pharmacologically active on the central nervous system, and can induce sensitization (develop allergies against itself). Eugenol may also aggravate an existing respiratory infection, so that the body cannot recover from the original infection. Burned eugenol, or a by-product, may also immobilize infection-fighting cells in the body, allowing bacteria to spread.

Who smokes clove cigarettes?
Children and teenagers smoke clove cigarettes; these products are rarely purchased by adults. A 2000 national survey of high school students reported that 12% of students reported having smoked clove cigarettes.

Adapted from:
Hutchinson Smoking Prevention Project. Background Information for Teachers. Seattle, WA: Fred Hutchinson Cancer Research Center. Rev. 1990.

APPENDIX 30: BIDIS

Bidis (pronounced “beedies”), an alternative type of cigarettes, are manufactured in India and exported worldwide. Bidis consist of finely ground, sun-dried tobacco rolled in a brown tendu leaf, often tied with a string. The tendu leaf is from a broad-leafed plant native to India. Bidi cigarettes are similar in appearance to hand-rolled marijuana cigarettes (joints). Most bidis are 60 mm in length; some are 100 mm. Some bidis have filters, but most are unfiltered. Highly flavored varieties of bidi cigarettes, including cherry, menthol, cinnamon, strawberry, vanilla, raspberry, and chocolate, are widely available in retail outlets and on the Internet. The flavor additives may partially account for the popularity of bidis among young teens. Other explanations for bidi popularity among teens are that they are less expensive than regular cigarettes, they are easily accessible, and they are trendy.

Bidis are hand-rolled by women and children. According to a 1996 report by Human Rights Watch of New York, ***Indian bidi manufacturers employ nearly 325,000 children as underpaid “bondage slaves.”***

Do you mean that bidis are made by child labor?
Yes. Bidis are only made by hand, and often by young children and teens in poor working conditions who are “bonded” or mortgaged to moneylenders, often for many years. They labor under harsh conditions for little more than $1.30 per week in bidi-making huts from 6 a.m. to 10 p.m., six days a week. Runaways are shackled with chains and made to work and sleep while chained. To earn their meager pay, the children must roll up to 1500 bidis each day.

Are bidis safer than regular cigarettes?
No. Some bidi smokers believe they are safer than regular cigarettes because they are rolled in a leaf, rather than paper. In fact, they may be more dangerous than commercial cigarettes. Although bidis contain less tobacco than American cigarettes, their tobacco contains significantly higher concentrations of nicotine than the tobacco in commercial cigarettes. Also, the tendu leaf that wraps the tobacco is very dense; users have to inhale harder and much more often to keep the bidi lit. Tests have shown that bidi smokers have to puff an average of 28 times per cigarette, compared to nine puffs for a regular cigarette. Because the smoker must inhale more deeply to keep the cigarette lit, he/she receives three times more carbon monoxide and nicotine, and five times more tar, than from regular cigarettes. The bidi smoker also receives higher doses of the other dangerous components of tobacco smoke – for example, phenol, hydrogen cyanide, ammonia, radioactive uranium, and benzopyrene.

Because these cigarettes are made in dark, dismal huts amid stench and litter instead of factories, and because they are made from the cheapest tobacco – the insect ridden, diseased flakes of the tobacco plant that no one else wants – ***each bidi could contain insects or animal feces***.

Should young smokers have special concerns about bidis?
Yes – when smoked in adolescence, bidis can actually stunt the growth of young smokers. They speed up the closing of growth plates in the long bones, effectively stopping them from ever reaching their full height potential. According to a study reported in the *Journal of Epidemiology and Community Health* (1980; 34:295-298), adolescents who smoked just two bidis daily for as little

as 2.5 years were significantly shorter than their nonsmoking peers.

Are bidis new?
No. Bidis have been imported to the United States for the last 40 years. Known as the “poor man’s cigarette” in India, they account for nearly 40% of tobacco consumption in that country. The candy-like flavors are added for export to the U.S.; few bidis in India are flavored. In India, a pack of 20 bidis costs eight cents. In the U.S., bidis sell for $2.50-3.00/pack.

Who smokes bidis?
In India, bidis are the poor man’s cigarette. They are smoked by adult males who can not afford to buy commercial cigarettes. In the United States, bidis are smoked mostly by people aged 12-21. The Center for Disease Control and Prevention reports that in a 2000 survey, 13% of high school students reported having smoked bidis.

Adapted from:
CDC. Bidi use among urban youth – Massachusetts, March-April, 1999. MMWR, 1999;48(36):796-799.
Warikoo N. In a fad, kids get burned. Detroit Free Press, 3/21/2000.
Malson JL,. Sims K, Murty R, Pickworth WB. Comparison of the nicotine content of tobacco used in bidis and conventional cigarettes. Tobacco Control, 2001;10(2):181-3.
Fisher L. Bidis--the latest trend in U.S. teen tobacco use. Cancer Causes & Control, 2000;11(6):577-8.

APPENDIX 31: ADDITIVE FREE CIGARETTES

Also called “all natural,” **additive-free cigarettes** are the tobacco industry’s attempt to take advantage of the public’s interest in all things natural and organic. They are cigarettes that have been made without the hundreds of ingredients normally added to cigarettes during manufacture by the five major cigarette companies. Hence, they are marketed as “additive free” or “natural.” Winston cigarettes are now marketed as additive free, as is the American Spirit brand.

If they are additive-free, are all-natural cigarettes safer than regular cigarettes?
No. There is no such thing as a safe or healthy cigarette. The only difference between all-natural cigarettes and conventional cigarettes is that the natural cigarettes contain only tobacco, no additives. The additive-free cigarettes still contain tar and nicotine, and give off carbon monoxide, nitrosamines, and thousands of other chemicals when burned. In fact, some of the all-natural cigarettes contain the highest amounts of tar and nicotine. For example, the Wall Street Journal (4/19/97, p. A-1) reported that American Spirit filter cigarettes, the most popular all-natural brand, contain 18.4 milligrams of tar and 1.87 milligrams of nicotine per cigarette, compared with 16 milligrams of tar and 1.1 milligrams of nicotine in one Marlboro cigarette.

Isn’t natural better?
There are plenty of poisons found in nature. Cyanide is naturally found in peach pits. That doesn’t mean people should ingest it. The same goes for tobacco. The bottom line is: there are no known health benefits of smoking additive-free cigarettes over other cigarettes.

Aren’t natural cigarettes a good alternative for smokers who don’t want to support the big tobacco companies?

Not really. Most of the all-natural cigarettes are manufactured or owned by the big tobacco companies. For example, American Spirit, the most popular all-natural brand, is owned by Philip Morris.

New promotion for a sagging cigarette brand.

Arnett, JJ. Winston’s “No Additives” campaign: “Straight Up”? “No Bull”? Public Health Reports, 1999; 114:522-527.

Synopsis: Once the most popular cigarette in the US, R.J. Reynolds’ Winston brand saw its market share decline in the 1970’s as Marlboro’s market share grew. This decline continued through the 1980’s and was not reversed until 1997. The source of this reversal was the “repositioning” of Winston through a new advertising campaign launched in summer, 1997. The campaign, still running today, presents Winston as a “Straight Up” brand, a cigarette with “100% Tobacco.” The ads promise “No additives” and “No Bull,” only “True Taste.” This advertising campaign has been controversial because of its implicit health claims. RJR has stated that the intention of the ads is simply to communicate that, unlike other brands, no non-tobacco ingredients have been added to the Winston tobacco blend during manufacture. Critics claim that the “no additives” slogan can be perceived as an implicit health claim because the statement “no additives or preservatives” is made in connection to foods being marketed with a health advantage. To determined how adolescents and adults perceived the “no additives” slogan, a study was conducted among 400 adolescents, aged 12-

17, and 203 adults, aged 30-50. Participants were shown four Winston ads and asked, "What do you think the Winston ads mean by saying that Winston's have 'no additives'? Circle ALL of the things you think the ads mean by saying 'no additives'." The five response options were: (1) "Winston cigarettes contain only tobacco," (2) "Winston cigarettes contain no added chemicals," (3) "Winston cigarettes are healthier than other cigarettes," (4) "Winston cigarettes are less likely than other cigarettes to harm your health," and (5) "Winston cigarettes are less likely than other cigarettes to be addictive."

As shown in Table 1, below, for adolescents and adults, the response chosen most often was one of the literal meanings ("no added chemicals"). However, a substantial proportion of the adolescents believed that "no additives" implied each of the three health claims. Adolescents were twice as likely as adults to believe that Winstons are "healthier than other cigarettes" and " less likely than other cigarettes to harm your health." The adolescents were three times more likely than adults to interpret "no additives" to mean that Winstons are "less likely than other cigarettes to be addictive." (These differences between adolescents and adults were statistically significant.) Overall, 67% of the adolescents believed that RJR was making at least one of the three health claims, compared with 27% of the adults. Results of this study suggest that Winston's "no additives" campaign is especially likely to deceive adolescents.

Table 1. Adolescents' and adults perceptions of the meaning of "No Additives" in advertisements for Winston cigarettes, 1998.

	**Percent who circled the indicated meaning**	
	**Adolescents**	**Adults**
Meaning: Winston cigarettes . . .	n=400	n=203
. . . contain only tobacco	42	44
. . . contain no added chemicals	61	72
. . . are healthier than other cigarettes	36	18
. . . are less likely than other cigarettes to harm your health	39	20
. . . are less likely than other cigarettes to be addictive	42	14

Adapted from:
Arnett JJ. Winston's "No Additives" campaign: "Straight Up"? "No Bull"? Public Health Reports, 1999; 114:522-527.
Kerber R. "Here's an oxymoron: All-natural smokes for health nuts," Wall Street Journal, 4/14/97, p. A1.

APPENDIX 32: CIGAR SMOKING

In the early to mid 1990’s, increased publicity of cigar use by celebrities, the introduction of "cigar bars," and the sub-culture of cigar paraphernalia such as humidors and clippers combined to make what was recently considered a dirty, smelly habit glamorous. Cigars seemed to be everywhere.

Since the banning of television ads for cigars in 1973, cigar consumption had steadily declined for almost two decades. That trend was reversed in 1992 when the cigar industry began marketing cigars as symbols of wealth and success, promoting big, glossy magazines such as *Cigar Aficionado*, and sponsoring events such as cigar nights at popular restaurants. As a result, cigar consumption increased by 50% in just five years. According to the U.S. Department of Agriculture Economic Research Service, cigar consumption hit an estimated 3.8 billion in 2001.

The recent increase in cigar consumption raises a number of important public health questions: What are the disease consequences of cigar smoking? What is the risk of addiction to nicotine from this form of tobacco use? Are the marketing practices that underlie the change in cigar consumption influencing adolescents to smoke cigars? What are the risks of environmental tobacco smoke exposure from cigar smoking?

What are the disease consequences of cigar smoking?
Cigar smoke is composed of the same toxic and carcinogenic constituents found in cigarette smoke and, because cigars have more tobacco per unit than cigarettes, they produce more carbon monoxide, nitrosamines, and other toxic chemicals when burned. Differences in disease risks produced by using cigarettes and cigars relates more to patterns of use and differences in inhalation, than to differences in smoke composition.

Cigar smoking can cause oral, esophageal, laryngeal, and lung cancers. The risk for esophageal and laryngeal cancers increases when cigar smoking is combined with heavy drinking. While lung cancer risk is lower for cigar smokers than cigarette smokers, the risk increases with more frequent cigar smoking and depth of inhalation. Regular cigar smokers who inhale, particularly those who smoke several cigars per day, have an increased risk of coronary heart disease and chronic obstructive pulmonary disease.

Little cigars are also very dangerous. Their physical properties are similar to those of cigarettes, and former cigarette smokers tend to use them as such. Most brands of little cigars have higher tar and nicotine levels than cigarettes.

What is the risk of addiction to nicotine from this form of tobacco use?
Cigars can deliver nicotine to the smoker in concentrations comparable to those delivered by cigarettes and smokeless tobacco. The alkaline pH of cigar smoke, and the tendency of cigar smokers not to inhale, results in nicotine absorption predominantly across the oral mucosa. Although not as fast a delivery system as the lungs, we know from studies of smokeless tobacco use that nicotine absorbed across the oral mucosa is capable of forming a powerful addiction.

Cigar smokers may spend up to an hour smoking a single large cigar that can contain as much tobacco as a pack of cigarettes. Thus, smoking even a few fat cigars could produce the same level of

nicotine exposure as that experienced by a pack-a-day cigarette smoker. The common practice of holding an unlit cigar in the mouth may also enable nicotine absorption.

Are the marketing practices that underlie the change in cigar consumption influencing adolescents to smoke cigars?

Yes. Prior to the current upswing in cigar use, most cigar smokers were middle-aged or older men, and they began smoking cigars as adults. Recent marketing efforts have promoted cigars as symbols of a luxuriant and successful lifestyle. Endorsements by celebrities, including athletes, elaborate cigar smoking events, and a resurgence of cigar smoking in the movies have all contributed to the increased visibility of cigar smoking in society and have helped lower barriers to cigar use in public. These same types of marketing efforts in the 1940's, 50's and 60's were highly successful at promoting cigarettes. Now, linking cigar smoking to an opulent and powerful lifestyle, and the featuring of highly visible men and women smoking cigars are core elements of cigar promotion. These promotions have been highly successful at increasing cigar smoking among men and *initiating* cigar smoking among women and teenagers.

Adolescent cigar use is occurring at a substantial level and is currently higher than that recorded in young adults prior to 1993. Currently, cigar use among adolescent males exceeds the use of smokeless tobacco in several states. The use of cigars is occurring among both males and females. In a 2001 CDC survey, 15.2 percent of U.S. high school students reported having smoked a cigar in the previous month. Male students (22.1 percent) were more likely than female students (8.5 percent) to smoke cigars.

In June of 2000, the Federal Trade Commission and seven of the USA's largest cigar makers made a deal to require warnings on cigar packages and in advertisements.

What are the risks of environmental tobacco smoke (ETS) exposure from cigar smoking?
Cigars are a major source of secondhand smoke that contains over 4,000 chemicals – 200 are poisons and 63 cause cancer. Tobacco smoke produced by cigars contains the same toxic and carcinogenic constituents found in cigarette smoke, and cigars generate similar amounts of ETS per minute, compared to cigarettes. Because it can take as long as 60-90 minutes to smoke a large cigar, the total amount of ETS generated by a single cigar is much greater than that from a single cigarette. Of particular concern is the amount of ETS generated in cigar bars or at cigar events where many people are smoking large cigars for extended periods. Measurements of smoke constituents at such events have shown carbon monoxide levels in the air similar to those on a crowded California freeway. Data such as these demonstrate that cigars can contribute substantial amounts of tobacco smoke to the indoor environment and, when large numbers of cigar smokers congregate together in a cigar bar or at a cigar smoking event, the amount of ETS produced is sufficient to be a health concern for individuals regularly required to work in those environments.

Adapted from:
Hutchinson Smoking Prevention Project. Background Information for Teachers. Seattle, WA: Fred Hutchinson Cancer Research Center. Rev. 1990.
USDHHS. Smoking and Tobacco Control, Monograph 9: Cigars: Health Effects and Trends, 1998. NIH, NCI, Bethesda, MD. NIH Pub. No. 98-4302.

APPENDIX 33: PIPE SMOKING

The prevalence of **pipe smoking** among men in the U.S. has declined substantially since 1965 and has continued to be very low among women. Reasons for the decreasing popularity of pipe smoking are not clear. Possible explanations include (1) public awareness of the dangers of tobacco use, including pipe smoking, (2) lack of appeal to large segments of the population, including women and adolescents, (3) clean indoor air policies that are more hostile to cigar and pipe smoking than even cigarette smoking, (4) the inconvenience and expense of purchasing pipes and other accessories (expenses not associated with other types of tobacco use), and (5) little advertising and promotion of pipe smoking (compared to cigarettes and cigars).

Pipe smoking is associated with several adverse health effects, including chronic obstructive pulmonary disease (COPD) and cancers of the oral cavity, lip, larynx, esophagus, stomach, and lung.

Most pipe smokers do not inhale tobacco smoke. Consequently, they have a lower risk of developing coronary heart disease and lung disease than do cigarette smokers. However, for those pipe smokers who *do* inhale, the risk of contracting heart or lung disease is greater than for cigarette smokers. Usually, cigarette smokers who switch to a pipe continue to inhale, even if unintentionally. This makes pipe smoking particularly hazardous to former cigarette smokers.

Pipe smokers who chronically chew the pipe stem can wear the enamel off their teeth, exposing the dentin, the inner, softer portion of the tooth. Tobacco residue can then penetrate the dentin, creating unsightly and irremovable stains. The position, shape and weight of the pipe stem can produce malocclusion, or crooked teeth.

Cancer is a risk for pipe smokers through the irritation of chemicals and the heat of pipe smoke. Small, elevated white bumps form on the soft palate around the red openings of partially blocked salivary gland ducts. This represents stomatitis nicotina, a pipe smokers' form of leukoplakia. Advanced cases show a thickened, cracked appearance.

Inhalation is not essential for exposure to the potentially harmful effects of pipe smoke. Contact with smoke dissolved in saliva or absorbed through mucous membranes of the mouth results in an increased risk of cancer of the mouth, throat, larynx, and stomach. Pipe smoking seems to be directly related to lip cancer.

Pipe smoke is more irritating to the eyes, nose, throat, and respiratory passages than cigarette smoke. The smoke of burning pipe tobacco contains many of the same toxic and carcinogenic constituents as cigarette smoke. Pipes, like cigars, contain higher levels of some toxic chemicals, such as benzopyrene, ammonia, and phenol, compared to cigarettes.

Adapted from:
Hutchinson Smoking Prevention Project. Background Information for Teachers. Seattle, WA: Fred Hutchinson Cancer Research Center. Rev. 1990.
Nelson DE, Davis RM, Chrismon JH, Giovion GA. Pipe smoking in the US, 1965-1991: Prevalence and attributable mortality. Preventive Medicine, 1996; 25(2):91-99.

APPENDIX 34: MARIJUANA

Marijuana (grass; pot; weed; joint = marijuana cigarette; hashish = stronger form of marijuana that contains more THC) is a drug made from the plant *Cannabis Sativa*. The drug contains more than 400 chemicals.

The mind-altering component of marijuana is THC (delta 9 tetrahydrocan-nabinol). The amount of THC in the marijuana determines the strength of its effects. Today's marijuana is about 10 times stronger than that smoked in the 1960s and 1970s. A potent and expensive form of marijuana called sinsemilla is up to 18 times more powerful than the marijuana of the 1960s.

There is great concern about the effects of marijuana on the lungs, particularly because it is inhaled deeply and held in the lungs to obtain the greatest "high." Although there is no tobacco in marijuana, marijuana smoke contains many of the same chemicals found in tobacco smoke, some in greater quantities, such as those linked to lung cancer (benzanthracene and benzopyrene) and chronic bronchitis (acetaldehyde). The concentration of tar is 20 times greater than in tobacco. It contains similar quantities of other irritants related to chronic bronchitis (hydro cyanine and acrolein) and emphysema (nitrogen oxides). Short-term effects of marijuana include decreased lung function, irritation of the lungs, and increased colds and lung infections.

Long-term use of marijuana can damage the immune system and affect the genetic structure of new cells. It can produce memory loss, interfere with body coordination, impair speech and vision, and cause a dramatic change in mood. It can also do serious damage to the respiratory system.

Researchers estimate that smoking three to five joints in a week has the same carcinogenic effect as smoking 16 tobacco cigarettes a day, seven days a week. Marijuana also affects the heart, raising the blood pressure and heart rate as much as 50 percent after only one joint.

Besides increased heart rate, the immediate effects of smoking marijuana include bloodshot eyes, and dry mouth and throat. Studies show that marijuana can alter sense of time, decrease ability to concentrate, and impair swift reactions and coordination needed to drive a car. People who have experienced marijuana's "acute panic anxiety reaction" describe it as extreme fear of "losing control," which leads to panic.

Marijuana may influence levels of sex hormones affecting menstrual cycles, ovulation, and milk production in females, and reducing testosterone levels in males. Infertility is a potential problem in both sexes. These effects are particularly important during the sexual development and maturation in teenagers.

Adapted from:
Hutchinson Smoking Prevention Project. Background Information for Teachers. Seattle, WA: Fred Hutchinson Cancer Research Center. Rev. 1990.
