# Supplementary material for: Does Effectiveness of Adolescent Smoking-Cessation Intervention Endure Into Young Adulthood? 7-Year Follow-Up Results from a Group-Randomized Trial
Source: PLoS One. 2016 Feb 1;11(2):e0146459. doi: 10.1371/journal.pone.0146459 (PMC4734743; doi:10.1371/journal.pone.0146459)
Supplement: S5 File — (PDF) [file pone.0146459.s005.pdf]

WRAP-UP SURVEY

Today's Date: ____________ ________ ___________
Month Day Year

Please give your accurate and honest answers, whatever they may be. We will use this information to help find new ways to prevent cancer.

Thank you for your help.

1. **How often do you see anti-smoking ads?**

   Every day.............................. ☐
   Several times each week .... ☐
   Only once in a while .... ☐
   Never .... ☐

2. **How much have your attitudes about cigarette smoking changed since your junior year of high school?**

   Not at all......... ☐
   Only a little...... ☐
   Quite a bit....... ☐
   A lot................ ☐

Is there anything you want to say about how your attitudes have changed? ____________

____

____

3. **How many cigarettes have you smoked in your entire life?**

None, not even a puff .... ❒
One cigarette or less .... ❒
2 – 20 cigarettes (up to one pack) .... ❒
21 – 100 cigarettes (2 – 5 packs) .... ❒
101 – 400 cigarettes (6 – 20 packs) .... ❒
Over 400 cigarettes (more than 20 packs)........... ❒

4. **Think back to September of your junior year of high school. Have you smoked *at all*, <u>even one cigarette</u>, between September of your junior year in high school and now?**

No .... ❒
Yes .... ❒

5. **How often do you currently smoke cigarettes?**

Not at all .... ❒
Less than once a month .... ❒
Once a month or more, but less than once a week ..... ❒
Once a week or more, but not daily........................... ❒

Go to #6 on page 3.

At least daily .... ❒

a. **How many cigarettes per day do you smoke?**

1 cigarette per day........................ ❒
2 – 5 cigarettes per day................. ❒
6 – 10 cigarettes per day............... ❒
11 – 20 cigarettes per day............. ❒
More than 20 .... ❒

b. **How soon <u>after waking up</u> do you have the <u>urge</u> for a cigarette?**

As soon as I wake up .... ❒
Within about 1/2 hour after I wake up .... ❒
An hour or two after I wake up .... ❒
Over 2 hours after I wake up .... ❒
Don't really have an urge to smoke .... ❒

c. **How soon <u>after waking up</u> do you <u>smoke</u> your first cigarette of the day?**

As soon as I wake up .... ❒
Within about 1/2 hour after I wake up .... ❒
An hour or two after I wake up .... ❒
Over 2 hours after I wake up .... ❒

6. **When was the last time you smoked, or even tried, a cigarette?**

   - I have never smoked, or even tried, a cigarette. ....❒
   - Earlier today ....❒
   - 1 – 7 days ago ....❒
   - 8 – 30 days ago ....❒
   - Between 1 month and 3 months ago ....❒
   - Between 3 months and 6 months ago ....❒
   - Over 6 months ago ....❒

7. **Do you think you will smoke cigarettes in the future?**

   - Yes, definitely .... ❒
   - Yes, I think so .... ❒
   - No, I don't think so .... ❒
   - No, definitely not .... ❒
   - Don't know .... ❒

8. **What's gone on with you *in the last 12 months* is very important for our study. Mark this period on the calendar below, as follows:**

   a. **Put an "X" on today's month.**

   b. **Count back 12 months and mark that month with another "X."**

2004				2005												2006						
SEP	OCT	NOV	DEC	JAN	FEB	MAR	APR	MAY	JUN	JUL	AUG	SEP	OCT	NOV	DEC	JAN	FEB	MAR	APR	MAY	JUN	JUL

Please use this 12-month period when answering the next questions.

First, some questions about your interactions with others…

9. **In the last 12 months (the period you marked in #8), were you ever *willing* to help someone stop smoking?**

   - No one I know smokes.......❒
   - No ....❒
   - Yes ....❒
   - Don't know ....❒

10. **In the last 12 months, how *important* was it to you to help someone stop smoking?**

    - Not at all important ....❒
    - Only a little important ....❒
    - Fairly important ....❒
    - Very important ....❒

11. **In the last 12 months, how *confident* were you that you could help someone stop smoking?**

- Not at all confident ❐
- Only a little confident ❐
- Fairly confident ❐
- Very confident ❐
- Not applicable ❐

12. **In the last 12 months, did you ever learn any tips or advice on how to help someone stop smoking?**

- No ❐
- Yes ❐
- Don't know ❐

13. **In the last 12 months, did you ever encourage anyone to stop smoking?**

- No one I know smokes ❐
- No ❐
- Yes ❐ → **How many?**
  - One ❐
  - Two ❐
  - Three ❐
  - Four ❐
  - Five or more ❐

Now about your own smoking or non-smoking in the last 12 months…

14. **In the last 12 months, what was the *longest period* of time you went <u>without</u> smoking (even a puff)?**

- I did <u>not smoke at all</u>, not even a puff ❐
- Less than 24 hours ❐
- 24 hours ❐
- 2 – 7 days ❐
- 8 – 30 days ❐
- Between 1 month and 3 months ❐
- Between 3 months and 6 months ❐
- Six months or more ❐

15. **In the last 12 months, have you smoked <u>at all</u>, even a puff?** *(Check one box.)*

- No, I have <u>not smoked at all</u> ❐ → **Go to #27 on page 8.**
- Yes, I have smoked one or more cigarettes ❐ → **Continue with #16.**

If you answered "Yes" to #15, please answer these questions as best you can, <u>even if they don't seem to apply to you</u>.

16. If you smoked at all in the last 12 months, *where* did you smoke your <u>last</u> cigarette?

- At home .... ❐
- At work................ ❐
- At school .... ❐
- At a party .... ❐
- At a restaurant ..... ❐
- At a concert.......... ❐
- In a car .... ❐
- Other: ______________________________

17. In the last 12 months, tell us *why* you might have smoked, or might currently smoke.

✓ ***<u>all</u> your reasons for smoking.***

- ❐ To change the way I feel; for example, to relax or reduce stress
- ❐ Because I enjoy it or it tastes good
- ❐ Because my friends or other people I'm around smoke
- ❐ Because it's a habit or I'm addicted
- ❐ Because I like the way it looks
- ❐ To keep my weight down
- ❐ To keep from being bored
- ❐ Just because I want to
- ❐ Because it's my choice/right to smoke if I want
- ❐ Because people don't want me to smoke
- ❐ It's something I do at parties or when I'm drinking
- ❐ It's a social thing for me
- ❐ Don't know
- ❐ Other reasons: ______________________________

18. In the last 12 months, how much did you <u>think</u> about stopping smoking entirely, or about cutting down?

- Not at all......... ❐
- Only a little...... ❐
- Quite a bit .... ❐
- A lot................ ❐

19. In the last 12 months, was it ever very *important* to you to stop smoking entirely?

- No ....❐
- Yes ....❐
- Don't know.......❐

20. If you smoked at all in the last 12 months, were you ever very *confident* that you could, if you wanted to, stop smoking entirely for at least 6 months?

No .... ❒
Yes .... ❒
Don't know....... ❒

21. In the last 12 months, did you ever *make plans and/or set a date* to stop smoking entirely?

No. I never even thought about stopping. .... ❒
No. I tried to stop, but never made plans or set a date to stop. .... ❒
Yes. I made plans or set a date to stop.. ❒

22. *What were, are, or might be* your reasons to stop smoking?

✓ ***all* of your reasons to stop smoking.**

❒ Most of the people I'm around don't smoke.
❒ I want to avoid getting a serious illness or disease.
❒ I want to prove to myself that I can stop smoking if I really want to.
❒ Smoking hurts my appearance and smells bad.
❒ I want friends or family to stop hassling me about it.
❒ I can already tell that smoking is hurting my health.
❒ I want to do better at physical activities.
❒ I want to do better at musical activities.
❒ I would feel better about myself if I were a non-smoker.
❒ It's expensive.
❒ Someone told me I absolutely must stop smoking.
❒ I want to feel in control of my life.
❒ I will get a special gift or reward if I stop smoking.
❒ I know people who died from smoking-related diseases.
❒ Stopping smoking will prove I can accomplish things that are important to me.
❒ People I am close to don't like it.
❒ I am concerned that smoking may cause me to die early.
❒ I want to prove to myself that I am not addicted to cigarettes.
❒ I want to stop supporting the tobacco industry.
❒ I had to keep from smoking on a family vacation.
❒ I don't want to smoke when I become a parent.
❒ I want to set a good example for my siblings or others.
❒ I've outgrown it.
❒ I'm not really a smoker anyway.
❒ Other: ____

23. **In the last 12 months, did you ever try to stop smoking entirely?** *(Check one box.)*

No. I never tried to stop smoking entirely. .... ❒ **Go to #27 on page 8.**

Yes. I tried to stop smoking entirely at least once..... ❒

Continue with #24

24. **How *determined* were you when you tried to stop smoking?**

- Not at all determined......... ❒
- Only a little determined...... ❒
- Fairly determined .... ❒
- Very determined .... ❒

25. **How many times in the last 12 months did you try to stop smoking entirely?**

__________ times (Your best estimate is fine.)

26. **If you have tried to stop smoking at least once in the last 12 months…**

a. **What thing or person helped you decide to try to stop smoking?**

✓ ***all** that apply.*

- ❒ Brochure. Describe: ____________________
- ❒ Telephone call(s) from the Fred Hutchinson Cancer Research Center
- ❒ Website. Which one: ____________________
- ❒ Boyfriend, girlfriend, spouse, or partner
- ❒ Teacher, school counselor, school nurse, or coach
- ❒ Poster. Describe: ____________________
- ❒ School detention
- ❒ TV or radio ads
- ❒ Family members
- ❒ Friend
- ❒ Price went up
- ❒ Rules against smoking
- ❒ Doctor told me to
- ❒ Just decided on my own
- ❒ Other: ____________________

b. **If you have tried to stop smoking in the last 12 months, what did you do to get ready before you tried to stop smoking?**

✓ ***all that apply.***

- ❐ Made a list of my reasons to stop
- ❐ Figured out my "high risk" situations and how to keep from smoking
- ❐ Threw away all my cigarettes
- ❐ Told friends/family I was stopping
- ❐ Made a plan for stopping
- ❐ Set a date to stop
- ❐ Got a friend to stop with me
- ❐ Quit drinking caffeine or alcohol
- ❐ Washed my clothes/room/car to get rid of smoke smell
- ❐ Got substitutes for cigarettes — gum, cinnamon sticks, straws, etc.
- ❐ Planned ways to overcome cravings
- ❐ Planned ways to reward myself for stopping
- ❐ Asked people not to smoke around me
- ❐ Planned activities to keep myself busy
- ❐ Got the "patch" or nicotine gum
- ❐ Cut down or quit — just to try it out
- ❐ Started drinking lots of water
- ❐ Learned other ways to control stress
- ❐ Other: ______________________________

c. **What was or is your biggest challenge for stopping smoking?**

____

Questions for everyone.

27. When was the last time you smoked, or even tried, a cigarette?

I have never smoked, or even tried a cigarette. ..... ❐

Approximate date of last cigarette: ________ Month ________ Day (if known) ________ Year

28. How often are you around people who smoke cigarettes?

A lot .... ❐
Sometimes ...... ❐
Hardly ever...... ❐
Never .... ❐

29. Is smoking allowed inside the place you currently live?

- No ......❒
- Yes ......❒

30. Think about the last 30 days. On how many of the last 30 days have you smoked one or more cigarettes? *(Check one box.)*

- Every day............❒
- 20 – 29 days ....❒
- 10 – 19 days ....❒
- 5 – 9 days ....❒
- 2 – 4 days ....❒
- 1 day...................❒

→ **Go to #35 on page 11.**

- 0 days ....❒

31. ***If you have <u>not smoked at all in the last 30 days</u>,*** **what are your reasons for not smoking?**

✓ ***all** of your reasons for not smoking.*

- ❒ Most of the people I'm around don't smoke.
- ❒ I want to avoid getting a serious illness or disease.
- ❒ I want to prove to myself that I can keep from smoking if I really want to.
- ❒ Smoking hurts my appearance and smells bad.
- ❒ Family or friends would hassle me about it.
- ❒ Someone told me I absolutely could not smoke.
- ❒ I want to feel in control of my life.
- ❒ I will get a special gift or reward if I don't smoke.
- ❒ I know people who died from smoking-related diseases.
- ❒ Not smoking will prove I can accomplish things that are important to me.
- ❒ People I am close to will be upset with me if I smoke.
- ❒ I am concerned that smoking may cause me to die early.
- ❒ I want to avoid addiction to cigarettes.
- ❒ I refuse to support the tobacco industry.
- ❒ I haven't been around the people I usually smoke with.
- ❒ I didn't have the money to buy cigarettes.
- ❒ I haven't been to a party in the last 30 days.
- ❒ I've been sick or had allergies and didn't feel like smoking.
- ❒ I never even thought about smoking.
- ❒ I quit or am trying to quit.
- ❒ Other: ____

32. **If you have not smoked at all in the last 30 days, how *important* is it for you to be a non-smoker for the rest of your life?**

- Not at all important .... ❒
- Only a little important .... ❒
- Fairly important .... ❒
- Very important.................... ❒

33. **How *confident* are you that you will be a non-smoker for the rest of your life?**

- Not at all confident .... ❒
- Only a little confident .... ❒
- Fairly confident .... ❒
- Very confident .... ❒

34. **How *committed* are you to being a non-smoker for the rest of your life?**

- Not at all committed...........❒
- Only a little committed........❒
- Fairly committed.................❒
- Very committed ....❒

→ **Go to #50 on page 13.**

These questions are for people who have smoked on one or more of the last 30 days.

Please answer all of these questions as best you can, even if they don't seem to apply to you.

35. **If you have smoked at all in the last 30 days, how would you describe your smoking pattern?**

    I smoke on a regular basis. .... ❐
    I smoke once in a while.......................... ❐
    I smoke only in social situations.............. ❐
    Other: ______________________________

36. **How much have you thought about the good and bad things about cigarette smoking?**

    Not at all.......... ❐
    Only a little....... ❐
    Quite a bit........ ❐
    A lot................ ❐

37. **People who smoke (even once in a while) can be at different levels of thinking about stopping for good.**

    Please check the one statement that indicates where you are now.

    I am not thinking of stopping. .... ❐
    I think I need to consider stopping someday.. ❐
    I think I should stop, but I'm not quite ready. .... ❐
    I'm starting to think about how to change my smoking patterns. .... ❐
    I'm taking action now to stop smoking (for example, cutting down, using the "patch").. ❐
    Other: ______________________________

38. **Do you have mixed feelings about your smoking?**

    No ....❐
    Yes ....❐
    Don't know......❐

39. **Do you like the idea of being someone who smokes?**

    No ....❐
    Yes ....❐
    Don't know......❐

40. If you have smoked at all in the last 30 days, do you think your smoking could get in the way of important future goals?

No ....❒
Yes ....❒
Don't know......❒

41. Do your friends ever bring up the idea that you should stop smoking?

No, never .... ❒
Once in a while ..... ❒
All the time........... ❒

42. Would you cut down or stop smoking entirely *if* you knew an easy way to do so?

No, I wouldn't cut down or stop, even if I knew an easy way. ..... ❒
Yes, I'd cut down or stop if I knew an easy way......................... ❒
Don't know.. ❒
Other: ______________________________

43. If you wanted to stop smoking, would you know *how* to do it?

No ....❒
Yes ....❒
Don't know......❒

44. Do you think that if you put your mind to it, you *could* stop smoking?

No ....❒
Yes ....❒
Don't know......❒

45. If you were to try to stop smoking, how *confident* are you that you could keep from smoking for at least 6 months?

Not at all confident .... ❒
Only a little confident .... ❒
Fairly confident .... ❒
Very confident .... ❒

46. How *important* is it for you to stop smoking entirely?

Not at all important .... ❒
Only a little important .... ❒
Fairly important .... ❒
Very important.................... ❒

47. How *committed* are you to becoming a non-smoker someday?

- Not at all committed ❐
- Only a little committed ❐
- Fairly committed ❐
- Very committed ❐

48. Do you *plan* to stop smoking entirely someday?

- No ❐
- Yes, in the next 30 days ❐
- Yes, between 1 and 6 months from now ❐
- Yes, sometime in the future, but not in the next 6 months ❐
- Don't know ❐

49. Do you think your friends would provide help or support if you tried to stop smoking?

- No ❐
- Yes ❐
- Don't know ❐

What else can you tell us that would help us understand your smoking?

____

____

Questions for everyone.

50. How often do you currently smoke or use:	Not at all	Less than once a month	Once a month or more, but not once a week	Once a week or more, but not daily	At least daily
a) chewing tobacco or snuff?	❑	❑	❑	❑	❑
b) tobacco pipes?	❑	❑	❑	❑	❑
c) cigars?	❑	❑	❑	❑	❑
d) clove cigarettes or bidis?	❑	❑	❑	❑	❑

51. When was the last time you used any tobacco product other than cigarettes (e.g., chewing tobacco, snuff, tobacco pipes, cigars, clove cigarettes, or bidis)?

- I have never used, or even tried, other tobacco products. ❐
- Earlier today ❐
- 1 – 7 days ago ❐
- 8 – 30 days ago ❐
- Between 1 month and 3 months ago ❐
- Between 3 months and 6 months ago ❐
- Over 6 months ago ❐

52. How many of your five closest friends…

	None	One	Two	Three	Four	Five
a) use chewing tobacco or snuff?	❑	❑	❑	❑	❑	❑
b) smoke cigarettes?	❑	❑	❑	❑	❑	❑

Next, we're interested in how much you agree or disagree with these statements.

53. Smoking can help people relax.

- Strongly agree .... ❑
- Agree .... ❑
- Disagree.................. ❑
- Strongly disagree ...... ❑
- Don't know .... ❑

54. There's no harm in having an occasional cigarette.

- Strongly agree .... ❑
- Agree .... ❑
- Disagree.................. ❑
- Strongly disagree ...... ❑
- Don't know .... ❑

55. People have to smoke a long time before it will hurt their health.

- Strongly agree .... ❑
- Agree .... ❑
- Disagree.................. ❑
- Strongly disagree ...... ❑
- Don't know .... ❑

56. Most people smoke.

- Strongly agree .... ❑
- Agree .... ❑
- Disagree.................. ❑
- Strongly disagree ...... ❑
- Don't know .... ❑

57. **Most people who smoke wish they could stop.**

Strongly agree .... ❒
Agree .... ❒
Disagree.................. ❒
Strongly disagree ...... ❒
Don't know .... ❒

58. **Smoking is addictive.**

Strongly agree .... ❒
Agree .... ❒
Disagree.................. ❒
Strongly disagree ...... ❒
Don't know .... ❒

59. **Stopping smoking often leads to weight gain.**

Strongly agree .... ❒
Agree .... ❒
Disagree.................. ❒
Strongly disagree ...... ❒
Don't know .... ❒

60. **Stopping smoking is hard to do.**

Strongly agree .... ❒
Agree .... ❒
Disagree.................. ❒
Strongly disagree ...... ❒
Don't know .... ❒

61. **Most people think smoking cigarettes is a bad idea.**

Strongly agree .... ❒
Agree .... ❒
Disagree.................. ❒
Strongly disagree ...... ❒
Don't know .... ❒

62. I think smoking cigarettes is a bad idea.

Strongly agree .... ❒
Agree .... ❒
Disagree.................. ❒
Strongly disagree ...... ❒
Don't know .... ❒

Read each sentence, then choose the answer that best describes you.

63. I feel comfortable not smoking even when I'm with others who are smoking.

Just like me .... ❒
Somewhat like me ..... ❒
Only a little like me .... ❒
Not like me............... ❒

64. I know how to keep from smoking in social situations (like parties) where I might feel tempted to smoke.

Just like me .... ❒
Somewhat like me ..... ❒
Only a little like me .... ❒
Not like me............... ❒

65. I feel I can ask someone for support to keep from smoking.

Just like me .... ❒
Somewhat like me ..... ❒
Only a little like me .... ❒
Not like me............... ❒

66. I know how to manage stress without smoking.

Just like me .... ❒
Somewhat like me ..... ❒
Only a little like me .... ❒
Not like me............... ❒

67. I have family or friends to turn to when I need help.

Just like me .... ❒
Somewhat like me ..... ❒
Only a little like me .... ❒
Not like me............... ❒

68. **When I need help with problems I'm having, friends try to understand and give me the support I need.**

Just like me .... ❒
Somewhat like me ..... ❒
Only a little like me .... ❒
Not like me................ ❒

69. **I am satisfied with my life.**

Just like me .... ❒
Somewhat like me ..... ❒
Only a little like me .... ❒
Not like me................ ❒

70. **The past year, I've had lots of stress in my life.**

Just like me .... ❒
Somewhat like me ..... ❒
Only a little like me .... ❒
Not like me................ ❒

71. **I can do anything I put my mind to.**

Just like me .... ❒
Somewhat like me ..... ❒
Only a little like me .... ❒
Not like me................ ❒

Just a few more questions…

72. Are you currently…

✓ ***all** that apply.*

❒ in the military?
❒ working?
❒ attending vocational school?
❒ attending college?
❒ staying home to raise a family?
❒ other: ______________________________

73. **Do you currently live with your parent or guardian?**

No ....❒
Yes ....❒

74. **What is your marital status?** *(Choose the one that best describes your current situation.)*

- Single, never married .... ☐
- Engaged .... ☐
- Married .... ☐
- Living together.................. ☐
- Separated......................... ☐
- Divorced .... ☐
- In serious relationship, not living together .... ☐
- Other: ____________________

75. **How many children do you have (who live with you at least part-time)?**

__________ children

76. **What is the *highest* level of high school education that you have completed?** *(Check one box.)*

- Completed 10th grade .... ☐
- Completed 11th grade .... ☐
- Started 12th grade, but did not graduate.......... ☐
- Graduated from high school............................ ☐
- Received GED.. ☐

77. **What is the *highest* level of post–high school education that you have completed?** *(Check one box.)*

- No post–high school education or training .... ☐
- Less than 1 year of college .... ☐
- 1 or more years of college, no degree................ ☐
- Associate degree .... ☐
- Bachelor's degree.. ☐
- Master's degree... ☐
- Post–high school training other than college ...... ☐

FEEDBACK QUESTIONS

Please help us improve our survey by answering these questions.

1. **Was the survey interesting to you?**

- Not at all interesting .... ☐
- Only a little interesting ...... ☐
- Fairly interesting .... ☐
- Very interesting .... ☐

2. **How long did it take you to complete the survey?**

15 minutes or less............❐
16–20 minutes ....❐
21–30 minutes ....❐
More than 30 minutes ......❐

3. **Did you give accurate answers to the survey questions?**

I answered all of the questions as accurately as I could .... ❐ → **Go to #4.**
I answered most of the questions as accurately as I could..... ❐
I answered some of the questions as accurately as I could.... ❐
Other: .... ❐

3a. Please give details, or say which questions you did not answer accurately.

____
____
____

4. **Is there anything you'd like to say about the survey questions?**

____
____

5. **Is there anything else you'd like to say about your smoking or non-smoking?**

____
____

That's it!

You have completed your

WRAP-UP SURVEY.

Your input may help us find new ways to prevent cancer.

THANK YOU SO MUCH!

NOW, PLEASE PUT IT IN THE ENVELOPE AND MAIL IT BACK TO US.

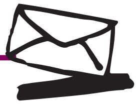

Form #OS04-0905
