# Supplementary material for: Does Effectiveness of Adolescent Smoking-Cessation Intervention Endure Into Young Adulthood? 7-Year Follow-Up Results from a Group-Randomized Trial
Source: PLoS One. 2016 Feb 1;11(2):e0146459. doi: 10.1371/journal.pone.0146459 (PMC4734743; doi:10.1371/journal.pone.0146459)
Supplement: S6 File — (PDF) [file pone.0146459.s006.pdf]

TOBACCO OPINIONS AND PRACTICES SURVEY
Changes from High School to Adulthood

We will use the information you provide to help find new ways to prevent cancer. Please give your accurate and honest answers, whatever they may be.

Thank you for your help!

1. **Today's Date:** ____________ ________ ___________
   Month Day Year

2. **How much have your attitudes about cigarette smoking *changed* between high school and now?**
   - Not at all......... ❐
   - Only a little...... ❐
   - Quite a bit....... ❐
   - A lot............... ❐

   2a. Is there anything you want to say about how your attitudes have changed? ______________________
   ____
   ____

3. **How often do you currently smoke cigarettes?**
   - Not at all...❐
   - Less than once a month...❐
   - Once a month or more, but less than once a week ....❐
   - Once a week or more, but not daily..................................❐
   - At least daily..❐

4. **When was the last time you smoked, or even tried, a cigarette?**
   - I have never smoked, or even tried, a cigarette ....❐
   - Over 6 years ago ....❐
   - Over 3 years ago ....❐
   - Over 12 months ago..❐
   - Between 6 months and 12 months ago ....❐
   - Between 3 months and 6 months ago ....❐
   - Between 1 month and 3 months ago....................................❐
   - 8 – 30 days ago...❐
   - 1 – 7 days ago...❐
   - Earlier today ....❐

FRED HUTCHINSON CANCER RESEARCH CENTER
A LIFE OF SCIENCE

1-888-270-0620

yasr@fhcrc.org

5. **How many cigarettes have you smoked in your entire life?**

None, not even a puff ❒ } **Go to #19 on page 6** →
One cigarette or less ❒ }
2 – 20 cigarettes (up to one pack) ❒ } **Go to #6 below**
21 – 100 cigarettes (2 – 5 packs) ❒ }
101 – 400 cigarettes (6 – 20 packs) ❒ }
Over 400 cigarettes (more than 20 packs) ❒ }

Now about your smoking or non-smoking in the last 6 years…

6. **In the last 6 years, have you smoked *at all*, even a puff?**
(check ✓ one box)

No. I have not smoked at all in the last 6 years. ❒ — **Go to #19 on page 6** →
Yes. I have smoked one or more cigarettes. ❒

7. **In the last 6 years, did you ever try to quit smoking entirely?**

No. I never tried to quit smoking entirely in the last 6 years. ❒ — **Go to #10** →
Yes. I tried to quit smoking entirely at least once in the last 6 years. ❒

8. **What helped you try to stop smoking?**

check ✓ all that apply

❒ Brochure or book: ____________________
❒ Telephone counseling. From what organization: ____________________
❒ Web or social networking site (Facebook, MySpace, etc.): ____________________
❒ Boyfriend, girlfriend, spouse, or partner
❒ Nicotine patch, gum, etc.
❒ Chantix, Zyban, etc.
❒ TV or radio ads. Describe: ____________________
❒ Friend
❒ Family members. What relationship(s): ____________________
❒ Pregnancy or parenthood
❒ Price went up
❒ Rules against smoking
❒ My doctor or personal health concerns
❒ Just decided on my own
❒ Other: ____________________

9. What did you do to get ready before you tried to stop smoking?

check ✓ all that apply

- ❒ Made a list of my reasons to stop
- ❒ Figured out my "high risk" situations and how to keep from smoking
- ❒ Threw away all my cigarettes
- ❒ Told friends/family I was stopping
- ❒ Made a plan for stopping
- ❒ Set a date to stop
- ❒ Got a friend to stop with me
- ❒ Quit drinking caffeine or alcohol
- ❒ Washed my clothes/room/car to get rid of smoke smell
- ❒ Got substitutes for cigarettes — gum, cinnamon sticks, straws, etc.
- ❒ Planned ways to overcome cravings
- ❒ Planned ways to reward myself for stopping
- ❒ Asked people not to smoke around me
- ❒ Planned activities to keep myself busy
- ❒ Got the "patch" or nicotine gum
- ❒ Cut down or quit — just to try it out
- ❒ Started drinking lots of water
- ❒ Learned other ways to control stress
- ❒ Other: ____________________________________

Now about your smoking or non-smoking in the last 12 months…

10. In the last 12 months, have you smoked at all, even a puff? (check ✓ one box)

No. I have not smoked at all in the last 12 months. ❒ — **Go to #18 on page 5** →

Yes. I have smoked one or more cigarettes. ❒

11. In the last 12 months, did you ever try to quit smoking *entirely*?

No. I never tried to quit entirely in the last 12 months. ❒ — **Go to #14 on page 4** →

Yes. I tried to quit at least once in the last 12 months. ❒

12. About how many times in the last 12 months did you try to quit smoking entirely?

_______ times *(Your best estimate is fine.)*

13. In the last 12 months, what was the longest period of time you went without smoking (even a puff)?

- Less than 24 hours ❐
- 24 hours ❐
- 2 – 7 days ❐
- 8 – 30 days ❐
- Between 1 month and 3 months ❐
- Between 3 months and 6 months ❐
- 6 months or more ❐

14. In the last 12 months, was it ever very *important* to you to quit smoking entirely?

- No ❐
- Yes ❐
- Don't know ❐

15. In the last 12 months, were you ever very *confident* that you could quit smoking entirely for at least 6 months?

- No ❐
- Yes ❐
- Don't know ❐

16. In the last 12 months, did you ever make plans and/or set a date to quit smoking entirely?

- No. I never even thought about quitting. ❐
- No. I tried to stop, but never made plans or set a date to quit smoking. ❐
- Yes. I made plans or set a date to quit smoking. ❐

17. Do you consider yourself to be a smoker?

- No ❐
- Yes ❐

Comment: ______________________________

Now, we're interested in your history of smoking and quitting…

18. *Think backwards* from 2012 to your junior year of high school. For each smoking experience below, tell us whether it occurred for you *at any time in each year*. *(It may help your memory to write important events under the year that they occurred, such as marriage, job change, college graduation, etc.)*

(a) **Was there a time between your junior year and 2012 that you smoked cigarettes every day?**

No ❒ — Go to **(b)** below.

Yes ❒ — Check the years below when, for at least part of the year, you smoked cigarettes every day. *(Your best estimate is fine.)*

2012 ❒	2011 ❒	2010 ❒	2009 ❒	2008 ❒	2007 ❒	2006 ❒	2005 ❒	2004 ❒

(b) **Was there a time between your junior year and 2012 that you smoked cigarettes occasionally, but not every day?**

No ❒ — Go to **(c)** below.

Yes ❒ — Check the years below when, for at least part of the year, you smoked cigarettes occasionally, but not every day. *(Your best estimate is fine.)*

2012 ❒	2011 ❒	2010 ❒	2009 ❒	2008 ❒	2007 ❒	2006 ❒	2005 ❒	2004 ❒

(c) **Was there a time between your junior year and 2012 that you did not smoke cigarettes at all for at least 6 months?**

No ❒ — Go to **#19** on page 6.

Yes ❒ — Check the years below when you did not smoke cigarettes at all for at least 6 months. *(Your best estimate is fine.)*

2012 ❒	2011 ❒	2010 ❒	2009 ❒	2008 ❒	2007 ❒	2006 ❒	2005 ❒	2004 ❒

QUESTIONS FOR EVERYONE.

19. How acceptable do you think it is for adults your age to smoke cigarettes?

- Not at all acceptable ❐
- Slightly acceptable ❐
- Moderately acceptable ❐
- Mostly acceptable ❐
- Completely acceptable ❐

20. How often are you around people who smoke cigarettes?

- A lot ❐
- Sometimes ❐
- Hardly ever ❐
- Never ❐

21. Is cigarette smoking allowed inside the place where you currently live?

- No ❐
- Yes ❐

22. Is cigarette smoking allowed inside the place where you currently work?

- No ❐
- Yes ❐
- Not applicable ❐

23. How many of your five closest friends smoke cigarettes?

- None ❐
- One ❐
- Two ❐
- Three ❐
- Four ❐
- Five ❐

24. Does your current spouse/romantic partner smoke cigarettes?

- ❐ No, never
- ❐ No, not since ___________/___________ *(approximate Month/Year)*
- ❐ Yes, but not every day
- ❐ Yes, daily
- ❐ I have no current spouse/romantic partner

Now, about your smoking or non-smoking in the last 30 days…

25. Think about the last 30 days. *On how many* of the last 30 days have you smoked one or more cigarettes?

- Every day .... ❐
- 20 – 29 days ..... ❐
- 10 – 19 days ..... ❐
- 5 – 9 days .... ❐
- 2 – 4 days .... ❐
- 1 day .... ❐

} **Continue with the next question, #26**

- 0 days .... ❐ → **Go to #43 on page 13** →

26. <u>On average</u>, on the days that you smoked in the past 30 days, how many cigarettes per day did you smoke?

_______ cigarettes per day

27. On the days that you smoke cigarettes, how soon <u>after waking up</u> do you <u>smoke</u> your first cigarette of the day?

- 0 – 5 minutes after waking up .... ❐
- 6 – 30 minutes after waking up .... ❐
- 31 – 60 minutes after waking up .... ❐
- More than 60 minutes after waking up .... ❐

28. Which of the following statements describe situations where you typically smoke?

check ✓ <u>all</u> that apply

- ❐ While socializing with friends
- ❐ At parties
- ❐ At clubs or bars
- ❐ While working or studying
- ❐ When taking a break at work or school
- ❐ Inside your home or apartment
- ❐ Outside in public spaces
- ❐ Driving in your car

29. Below is a list of statements about smoking. Please tell us what you expect or believe happens for you as a result of smoking cigarettes. For each statement, check the choice that best describes your expectation of how often it might happen for you.

I believe / expect that…

a. Smoking satisfies my nicotine cravings.	Never ❐	Rarely ❐	Sometimes ❐	Often ❐	Always ❐
b. I enjoy the flavor of a cigarette.	Never ❐	Rarely ❐	Sometimes ❐	Often ❐	Always ❐
c. Smoking a cigarette energizes me.	Never ❐	Rarely ❐	Sometimes ❐	Often ❐	Always ❐
d. Smoking keeps my weight down.	Never ❐	Rarely ❐	Sometimes ❐	Often ❐	Always ❐
e. When I am alone, a cigarette helps me pass the time.	Never ❐	Rarely ❐	Sometimes ❐	Often ❐	Always ❐
f. Nicotine "fits" can be controlled by smoking.	Never ❐	Rarely ❐	Sometimes ❐	Often ❐	Always ❐
g. When I smoke, the taste is pleasant.	Never ❐	Rarely ❐	Sometimes ❐	Often ❐	Always ❐
h. A cigarette gives me energy when I'm bored and tired.	Never ❐	Rarely ❐	Sometimes ❐	Often ❐	Always ❐
i. Smoking helps control my weight.	Never ❐	Rarely ❐	Sometimes ❐	Often ❐	Always ❐
j. If I have nothing to do, a cigarette helps me kill time.	Never ❐	Rarely ❐	Sometimes ❐	Often ❐	Always ❐
k. I enjoy the taste sensations while smoking.	Never ❐	Rarely ❐	Sometimes ❐	Often ❐	Always ❐
l. Cigarettes keep me from eating more than I should.	Never ❐	Rarely ❐	Sometimes ❐	Often ❐	Always ❐

30. People who smoke (even once in a while) can be at <u>different levels</u> of thinking about quitting for good.

Please check the <u>one</u> statement that indicates where you are <u>now</u>.

- I am not thinking of quitting. ❒
- I think I need to consider quitting someday. ❒
- I think I should quit, but I'm not quite ready. ❒
- I'm starting to think about how to change my smoking patterns. ❒
- I'm taking action now to quit smoking (for example, cutting down, using the "patch"). ❒

31. Would you cut down or quit smoking entirely *if* you knew an easy way to do so?

- No, I wouldn't cut down or quit, even if I knew an easy way. ❒
- Yes, I'd cut down or quit if I knew an easy way. ❒
- Don't know. ❒
- Other: ____________________

32. If you wanted to quit smoking entirely, would you know *how* to do it?

- No ❒
- Yes ❒
- Don't know ❒

33. Do you think that if you put your mind to it, you *could* quit smoking entirely?

- No ❒
- Yes ❒
- Don't know ❒

34. If you were to try to quit smoking, how *confident* are you that you could keep from smoking entirely for at least 6 months?

- Not at all confident ❒
- Only a little confident ❒
- Fairly confident ❒
- Very confident ❒

35. How *important* is it for you to quit smoking entirely?

- Not at all important .... ❒
- Only a little important .... ❒
- Fairly important .... ❒
- Very important .... ❒

36. Do you *plan* to quit smoking entirely someday?

- No .... ❒
- Yes, in the next 30 days... ❒
- Yes, between 1 and 6 months from now .... ❒
- Yes, sometime in the future, but not in the next 6 months........ ❒
- Don't know .... ❒

37. Do you think your family, friends or co-workers would provide help or support if you tried to quit smoking entirely?

- No .... ❒
- Yes .... ❒
- Don't know........... ❒

38. Do you have mixed feelings about your smoking?

- No .... ❒
- Yes .... ❒
- Don't know........... ❒

39. Do you like the idea of being someone who smokes?

- No .... ❒
- Yes .... ❒
- Don't know........... ❒

40. Do you think your smoking could get in the way of important future goals?

- No .... ❒
- Yes .... ❒
- Don't know........... ❒

41. For each statement below, tell us how strongly you agree or disagree that it will happen for you personally as a result of stopping smoking entirely.

check ✓ one box for each statement

**If I quit smoking completely, then...**	Strongly Disagree	Disagree	Neither Agree nor Disagree	Agree	Strongly Agree
a. I will be nervous and unbalanced.	❐	❐	❐	❐	❐
b. I will feel healthier.	❐	❐	❐	❐	❐
c. I will have more money to spend on other things.	❐	❐	❐	❐	❐
d. I will have more energy.	❐	❐	❐	❐	❐
e. I will be less likely to get sick.	❐	❐	❐	❐	❐
f. I will simply feel better physically.	❐	❐	❐	❐	❐
g. I will have a harder time unwinding.	❐	❐	❐	❐	❐
h. Others will admire my will power.	❐	❐	❐	❐	❐
i. I will simply feel better emotionally.	❐	❐	❐	❐	❐
j. I will gain weight.	❐	❐	❐	❐	❐
k. I will feel grumpy.	❐	❐	❐	❐	❐
l. I will be a good role model (e.g., for my partner or children).	❐	❐	❐	❐	❐
m. I will be more attractive to others (whiter teeth, clothes smell better).	❐	❐	❐	❐	❐

42. How much do you agree or disagree with the following statements?

check ✓ one box for each statement.

	Strongly Disagree	Disagree	Neither Agree nor Disagree	Agree	Strongly Agree
a. I'm willing to put up with whatever discomfort I have to in order to quit smoking.	❐	❐	❐	❐	❐
b. Once I quit, no matter how difficult it may be, I won't let myself smoke.	❐	❐	❐	❐	❐
c. Feeling very anxious or restless won't prevent me from quitting smoking.	❐	❐	❐	❐	❐
d. Once I quit, even if I really want one, I won't let myself pick up a cigarette.	❐	❐	❐	❐	❐
e. No matter how much I crave a cigarette when I quit, I'm going to resist the urge to smoke.	❐	❐	❐	❐	❐
f. Feeling very depressed or sad won't prevent me from quitting smoking.	❐	❐	❐	❐	❐
g. I'm not going to let anything get in the way of my quitting smoking.	❐	❐	❐	❐	❐
h. Feeling very angry and irritable won't prevent me from quitting smoking.	❐	❐	❐	❐	❐

Next → Go to #47 on page 14 →
(please skip #43 through #46)

For people who have <u>not smoked at all</u> in the last thirty days:

43. What are your reasons for not smoking?

check ✓ <u>all</u> your reasons for not smoking

- ❒ Most of the people I'm around don't smoke.
- ❒ I want to avoid getting a serious illness or disease.
- ❒ I want to prove to myself that I can quit smoking if I really want to.
- ❒ Smoking hurts my appearance and smells bad.
- ❒ Family or friends would hassle me about it.
- ❒ I want to feel in control of my life.
- ❒ I know people who died from smoking-related diseases.
- ❒ Not smoking proves I can accomplish things that are important to me.
- ❒ People I am close to would be upset with me if I smoke.
- ❒ I don't want to smoke because I'm a parent or plan to be one.
- ❒ I want to avoid addiction to cigarettes.
- ❒ I refuse to support the tobacco industry.
- ❒ I haven't been around the people I usually smoke with.
- ❒ Cigarettes are too expensive.
- ❒ I haven't been to a party in the last 30 days.
- ❒ I've been sick or had allergies and didn't feel like smoking.
- ❒ I never even thought about smoking.
- ❒ I quit or am trying to quit.
- ❒ Other: ____

44. How *important* is it for you to be a non-smoker for the rest of your life?

Not at all important .... ❒
Only a little important .... ❒
Fairly important .... ❒
Very important...................... ❒

45. How *confident* are you that you will be a non-smoker for the rest of your life?

Not at all confident............... ❒
Only a little confident............ ❒
Fairly confident .... ❒
Very confident .... ❒

46. How *committed* are you to being a non-smoker for the rest of your life?

Not at all committed............. ❒
Only a little committed.......... ❒
Fairly committed .... ❒
Very committed .... ❒

Questions for everyone.

47. **When was the last time you smoked, or even tried, a cigarette?**

Approximate date of last cigarette: ________/________/__________
Month Day (if known) Year

I have never smoked, or even tried, a cigarette. ....❒

Next, we're interested in how much you agree or disagree with these statements.

48. Smoking can help people relax.

- Strongly agree .... ❒
- Agree .... ❒
- Disagree.............................. ❒
- Strongly disagree .... ❒
- Don't know .... ❒

49. Most people smoke.

- Strongly agree .... ❒
- Agree .... ❒
- Disagree.............................. ❒
- Strongly disagree .... ❒
- Don't know .... ❒

Read each sentence, and then choose the answer that best describes you.

50. I am confident that I can resist smoking in stressful situations.

Just like me .... ❒
Somewhat like me .... ❒
Only a little like me .... ❒
Not like me.......................... ❒

51. I am able to refrain from smoking even when I'm with others who are smoking.

Just like me .... ❒
Somewhat like me .... ❒
Only a little like me .... ❒
Not like me.......................... ❒

52. I think about how my actions will affect others.

Just like me .... ❒
Somewhat like me .... ❒
Only a little like me .... ❒
Not like me.......................... ❒

53. I know how to keep from smoking in social situations (like parties) where I might feel tempted to smoke.

Just like me .... ❒
Somewhat like me .... ❒
Only a little like me .... ❒
Not like me.......................... ❒

54. I am financially independent of my parents.

Just like me .... ❒
Somewhat like me .... ❒
Only a little like me .... ❒
Not like me.......................... ❒

55. **I know how to manage stress without smoking.**

Just like me .... ❒
Somewhat like me .... ❒
Only a little like me .... ❒
Not like me.......................... ❒

56. **When I make a decision that turns out poorly, I take full responsibility.**

Just like me .... ❒
Somewhat like me .... ❒
Only a little like me .... ❒
Not like me.......................... ❒

57. **I am confident that I can resist smoking in social situations.**

Just like me .... ❒
Somewhat like me .... ❒
Only a little like me .... ❒
Not like me.......................... ❒

58. **I make independent decisions about things that concern my life.**

Just like me .... ❒
Somewhat like me .... ❒
Only a little like me .... ❒
Not like me.......................... ❒

59. **How many of the people living in your residence, including yourself, smoke cigarettes?**

____

60. **What is your current marital status?**

check* ✓ *the one that best describes your current situation

❒ Currently married
❒ Separated
❒ Divorced
❒ Widowed
❒ Never married
Other/comments: ____

61. How often do you currently smoke or use:

	Not at all	Less than once a month	Once a month or more, but not once a week	Once a week or more, but not daily	At least daily
a. chewing tobacco or snuff?	❒	❒	❒	❒	❒
b. snus (Camel Snus, Taboka, Marlboro Snus, etc.)?	❒	❒	❒	❒	❒
c. cigarillos or little cigars (Black and Mild, Swisher Sweets, Winchester, etc.)?	❒	❒	❒	❒	❒
d. full-size cigars (Dutch Masters, etc.)?	❒	❒	❒	❒	❒
e. tobacco pipes?	❒	❒	❒	❒	❒
f. clove cigarettes or bidis?	❒	❒	❒	❒	❒
g. nicotine-replacement products (gum, patch, etc.)?	❒	❒	❒	❒	❒
h. electronic cigarettes (Njoy, Blu, Ruyan, etc.)?	❒	❒	❒	❒	❒
i. other tobacco products (other than cigarettes)?	❒	❒	❒	❒	❒

→ Please specify which other tobacco products: ______________________________

Just a few more questions....

62. To confirm that you are the person who took part in this study previously, please provide your date of birth: __________/__________/____________

Month Day Year

63. Are you currently…

check ✓ all that apply

- ❒ in the military?
- ❒ working?
- ❒ attending vocational school?
- ❒ attending college?
- ❒ staying home to raise a family?
- ❒ unemployed or looking for work?
- ❒ Other: ______________________________

64. Are you raising any children in your household (who live with you at least part-time)?

- ❒ No
- ❒ Yes. What are their ages? _____, _____, _____, _____, _____, _____

65. What is the highest level of high school education that you have completed?

check ✓ one box

- ❒ Completed 10th grade (or less)
- ❒ Completed 11th grade
- ❒ Started 12th grade, but did not graduate
- ❒ Graduated from high school
- ❒ Received GED

66. What is the highest level of post-high school education that you have completed?

check ✓ one box

- ❒ No post-high school education or training
- ❒ Less than 1 year of college
- ❒ 1 or more years of college, no college degree
- ❒ Associate's degree
- ❒ Bachelor's degree
- ❒ Master's degree
- ❒ Ph.D., M.D., or J.D.
- ❒ Completed vocational/technical/career program: ______________________
- ❒ Completed other post-high school training: ______________________

67. Are you of Hispanic or Latino origin?

- ❒ No
- ❒ Yes

68. **Which one or more of the following would you say is your race?**

check ✓ all that apply

- ❒ American Indian or Alaska native
- ❒ Asian
- ❒ Black or African American
- ❒ Native Hawaiian or other Pacific Islander
- ❒ White
- ❒ Other: ______________________________

Finally, we'd like your feedback on the survey…

69. **Are you (the person filling out this survey) the study participant named in the letter that came with this survey?**

- ❒ No Comment: ____
- ❒ Yes

70. **Was this survey interesting to you?**

- Not at all interesting .... ❒
- Only a little interesting .... ❒
- Fairly interesting .... ❒
- Very interesting..................... ❒

71. **About how long did it take you to complete the survey?**

- 20 minutes or less................ ❒
- 21 – 30 minutes .... ❒
- More than 30 minutes .... ❒

72. **Did you give accurate answers to the survey questions?**

- I answered <u>some</u> of the questions as accurately as I could........ ❒
- I answered <u>most</u> of the questions as accurately as I could......... ❒
- I answered <u>all</u> of the questions as accurately as I could .... ❒
- Other: .... ❒

72a. **If you chose "some," "most" or "other" above, please give details, or say which answers were not accurate.**

____

____

73. Is there anything you'd like to say *about the survey questions*?

____

____

____

74. Is there anything you'd like to say about *your smoking or non-smoking*?

____

____

____

75. Any final comments?

____

____

____

That's it!

You have completed your

TOPS SURVEY

Your input may help us find new ways to prevent cancer.

THANK YOU SO MUCH!

NOW, PLEASE PUT IT IN THE ENVELOPE AND MAIL IT BACK TO US.

Form # TOPS03-0712
