# Supplementary material for: Does Effectiveness of Adolescent Smoking-Cessation Intervention Endure Into Young Adulthood? 7-Year Follow-Up Results from a Group-Randomized Trial
Source: PLoS One. 2016 Feb 1;11(2):e0146459. doi: 10.1371/journal.pone.0146459 (PMC4734743; doi:10.1371/journal.pone.0146459)
Supplement: S7 File — (PDF) [file pone.0146459.s007.pdf]

Smoking and Nonsmoking Attitudes and Practices

Your Changes from High School to Adulthood

The 2013 Short Survey

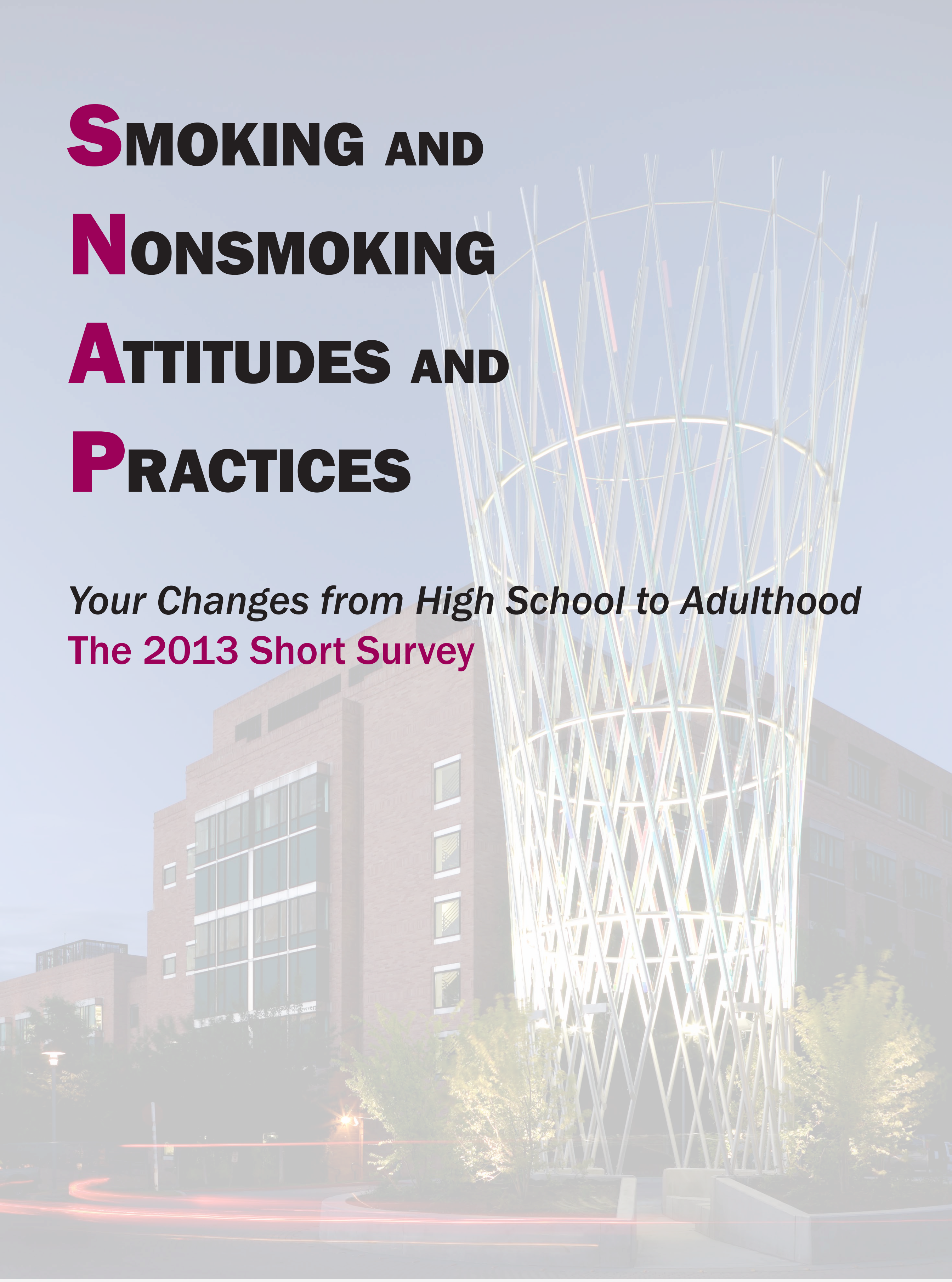

FRED HUTCHINSON
CANCER RESEARCH CENTER
A LIFE OF SCIENCE

Welcome to SNAP, the 2013 Short Survey.

Your responses will help find new ways to prevent cancer. Please give your accurate and honest answers, whatever they may be.

Thank you for your help!

1. **Today's Date:** ____________ ________ __________
   Month Day Year

First a few questions about your opinions and attitudes...

2. **How much have your attitudes about cigarette smoking *changed* between high school and now?**

   Not at all .... ❒
   Only a little .... ❒
   Quite a bit .... ❒
   A lot .... ❒

   2a. **Is there anything you want to say about how your attitudes have changed?**

   ____

   ____

   ____

3. **Do you think there are too many restrictions about where and when people may smoke cigarettes?**

   No .... ❒
   Yes .... ❒
   Don't know .... ❒

4. **Do you think anti-smoking ads on TV and radio exaggerate the dangers of smoking?**

   No .... ❒
   Yes .... ❒
   Don't know .... ❒
   I haven't seen any anti-smoking ads...... ❒

5. **How often do you currently smoke cigarettes?**

Not at all ❒
Less than once a month ❒
Once a month or more, but less than once a week ❒
Once a week or more, but not daily ❒
At least daily ❒

6. **When was the last time you smoked, or even tried, a cigarette?**

I have never smoked, or even tried, a cigarette ❒
Over 6 years ago ❒
Over 3 years ago ❒
Over 12 months ago ❒
Between 6 months and 12 months ago ❒
Between 3 months and 6 months ago ❒
Between 1 month and 3 months ago ❒
8 – 30 days ago ❒
1 – 7 days ago ❒
Earlier today ❒

7. **How many cigarettes have you smoked in your entire life?**

None, not even a puff ❒ } Go to #20 on page 5 →
One cigarette or less ❒
2 – 20 cigarettes (up to one pack) ❒ } Go to #8 below
21 – 100 cigarettes (2 – 5 packs) ❒
101 – 400 cigarettes (6 – 20 packs) ❒
Over 400 cigarettes (more than 20 packs) ❒

Now about your smoking or non-smoking in the last 6 years...

8. **In the last 6 years, have you smoked *at all*, even a puff?**
check ✓ one box

No. I have not smoked at all in the last 6 years. ❒ — Go to #20 on page 5 →
Yes. I have smoked one or more cigarettes. ❒ — Go to #9 below

9. **In the last 6 years, did you ever try to quit smoking entirely?**

No. I never tried to quit smoking entirely in the last 6 years. ❒ — Go to #12 on page 4 →
Yes. I tried to quit smoking entirely at least once in the last 6 years. ❒ — Go to #10 on page 3 →

10. What helped you try to stop smoking?

check ✓ all that apply

- ❒ Brochure or book:____
- ❒ Telephone counseling. From what organization:______________________
- ❒ Web or social networking site (Facebook, MySpace, etc.): ________________
- ❒ Boyfriend, girlfriend, spouse, or partner
- ❒ Nicotine patch, gum, etc.
- ❒ Chantix, Zyban, etc.
- ❒ TV or radio ads. Describe:___________________________________
- ❒ Friend
- ❒ Family members. What relationship(s):__________________________
- ❒ Pregnancy or parenthood
- ❒ Price went up
- ❒ Rules against smoking
- ❒ My doctor or personal health concerns
- ❒ Just decided on my own
- ❒ Other:____

11. What did you do to get ready before you tried to stop smoking?

check ✓ all that apply

- ❒ Made a list of my reasons to stop
- ❒ Figured out my "high risk" situations and how to keep from smoking
- ❒ Threw away all my cigarettes
- ❒ Told friends/family I was stopping
- ❒ Made a plan for stopping
- ❒ Set a date to stop
- ❒ Got a friend to stop with me
- ❒ Quit drinking caffeine or alcohol
- ❒ Washed my clothes/room/car to get rid of smoke smell
- ❒ Got substitutes for cigarettes—gum, cinnamon sticks, straws, etc.
- ❒ Planned ways to overcome cravings
- ❒ Planned ways to reward myself for stopping
- ❒ Asked people not to smoke around me
- ❒ Planned activities to keep myself busy
- ❒ Got the "patch" or nicotine gum
- ❒ Cut down or quit—just to try it out
- ❒ Started drinking lots of water
- ❒ Learned other ways to control stress
- ❒ Other:____

Now about your smoking or non-smoking in the last 12 months...

12. In the last 12 months, have you smoked at all, even a puff?
check ✓ one box

No. I have not smoked at all in the last 12 months. ❒ Go to #20 on page 5
Yes. I have smoked one or more cigarettes. ❒

13. In the last 12 months, did you ever try to quit smoking *entirely*?

No. I never tried to quit entirely in the last 12 months. ❒ Go to #16 below
Yes. I tried to quit at least once in the last 12 months. ❒

14. About how many times in the last 12 months did you try to quit smoking entirely?

_______ *times* ***(Your best estimate is fine.)***

15. In the last 12 months, what was the longest period of time you went without smoking (even a puff)?

Less than 24 hours .... ❒
24 hours .... ❒
2 – 7 days .... ❒
8 – 30 days .... ❒
Between 1 month and 3 months .... ❒
Between 3 months and 6 months .... ❒
6 months or more .... ❒

16. In the last 12 months, was it ever very *important* to you to quit smoking entirely?

No .... ❒
Yes .... ❒
Don't know .... ❒

17. In the last 12 months, were you ever very *confident* that you could quit smoking entirely for at least 6 months?

No .... ❒
Yes .... ❒
Don't know .... ❒

18. In the last 12 months, did you ever make plans and/or set a date to quit smoking entirely?

No. I never even thought about quitting. .... ❒
No. I tried to stop, but never made plans or set a date to quit smoking. ...... ❒
Yes. I made plans or set a date to quit smoking. .... ❒

19. Do you consider yourself to be a smoker?

- No ❐
- Yes ❐

Comment: ______________________________

Questions for everyone.

20. How acceptable do you think it is for adults your age to smoke cigarettes?

- Not at all acceptable ❐
- Slightly acceptable ❐
- Moderately acceptable ❐
- Mostly acceptable ❐
- Completely acceptable ❐

21. How many of your five closest friends smoke cigarettes?

- None ❐
- One ❐
- Two ❐
- Three ❐
- Four ❐
- Five ❐

22. Does your current spouse/romantic partner smoke cigarettes?

- ❐ No, never
- ❐ No, not since ____________ / ____________
  (approximate Month / Year)
- ❐ Yes, but not every day
- ❐ Yes, daily
- ❐ I have no current spouse/romantic partner

Now, about your smoking or non-smoking in the last 30 days...

23. Think about the last 30 days. *On how many* of the last 30 days have you smoked one or more cigarettes?

- Every day ❐
- 20 – 29 days ❐
- 10 – 19 days ❐
- 5 – 9 days ❐
- 2 – 4 days ❐
- 1 day ❐

Go to #24 on page 6 →

- 0 days ❐ Go to #30 on page 7 →

24. On average, on the days that you smoked in the past 30 days, how many cigarettes per day did you smoke?

_______ cigarettes per day

25. On the days that you smoke cigarettes, how soon after waking up do you smoke your first cigarette of the day?

0 – 5 minutes after waking up .... ❐
6 – 30 minutes after waking up .... ❐
31 – 60 minutes after waking up .... ❐
More than 60 minutes after waking up .... ❐

26. Which of the following statements describe situations where you typically smoke?

check ✓ all that apply

- ❐ While socializing with friends
- ❐ At parties
- ❐ At clubs or bars
- ❐ While working or studying
- ❐ When taking a break at work or school
- ❐ Inside your home or apartment
- ❐ Outside in public spaces
- ❐ Driving in your car

27. People who smoke (even once in a while) can be at different levels of thinking about quitting for good. ***Please check the one statement that indicates where you are now.***

I am not thinking of quitting. .... ❐
I think I need to consider quitting someday. .... ❐
I think I should quit, but I'm not quite ready. .... ❐
I'm starting to think about how to change my smoking patterns. .... ❐
I'm taking action now to quit smoking (for example, cutting down, using the "patch"). ... ❐

28. Would you cut down or quit smoking entirely *if* you knew an easy way to do so?

No, I wouldn't cut down or quit, even if I knew an easy way. ... ❐
Yes, I'd cut down or quit if I knew an easy way. .... ❐
Don't know. .... ❐
Other: ____

29. Do you *plan* to quit smoking entirely someday?

No .... ❐
Yes, in the next 30 days .... ❐
Yes, between 1 and 6 months from now .... ❐
Yes, sometime in the future, but not in the next 6 months....... ❐
Don't know .... ❐

Questions for everyone.

30. When was the last time you smoked, or even tried, a cigarette?

Approximate date of last cigarette: ____________ / ___________ / _____________
Month Day (if known) Year

I have never smoked, or even tried, a cigarette. .... ❒

31. How often do you currently smoke or use:

	Not at all	Less than once a month	Once a month or more, but not once a week	Once a week or more, but not daily	At least daily
a. chewing tobacco or snuff?	❒	❒	❒	❒	❒
b. snus (Camel Snus, Taboka, Marlboro Snus, etc.)?	❒	❒	❒	❒	❒
c. cigarillos or little cigars (Black and Mild, Swisher Sweets, Winchester, etc.)?	❒	❒	❒	❒	❒
d. full-size cigars (Dutch Masters, etc.)?	❒	❒	❒	❒	❒
e. tobacco pipes?	❒	❒	❒	❒	❒
f. clove cigarettes or bidis?	❒	❒	❒	❒	❒
g. nicotine-replacement products (gum, patch, etc.)?	❒	❒	❒	❒	❒
h. electronic cigarettes (Njoy, Blu, Ruyan, etc.)?	❒	❒	❒	❒	❒
i. other tobacco products (other than cigarettes)?	❒	❒	❒	❒	❒

→ Please specify which other tobacco products: ________________________________

32. How many of the people living in your residence, including yourself, smoke cigarettes?

____

Just a few more questions....

33. To confirm that you are the person who took part in this study previously, please provide your date of birth:

__________ / __________ / __________

Month Day Year

34. What is your current marital status?

check ✓ the one that best describes your current situation

- ❒ Currently married
- ❒ Separated
- ❒ Divorced
- ❒ Widowed
- ❒ Never married

Other/comments: ____

35. Are you currently...

check ✓ all that apply

- ❒ in the military?
- ❒ working?
- ❒ attending vocational school?
- ❒ attending college?
- ❒ staying home to raise a family?
- ❒ unemployed or looking for work?
- ❒ Other: ____

36. Are you raising any children in your household (who live with you at least part-time)?

- ❒ No
- ❒ Yes. What are their ages? _____, _____, _____, _____, _____, _____

37. What is the highest level of high school education that you have completed?

check ✓ one box

- ❐ Completed 10th grade (or less)
- ❐ Completed 11th grade
- ❐ Started 12th grade, but did not graduate
- ❐ Graduated from high school
- ❐ Received GED

38. What is the highest level of post-high school education that you have completed?

check ✓ one box

- ❐ No post-high school education or training
- ❐ Less than 1 year of college
- ❐ 1 or more years of college, no college degree
- ❐ Associate's degree
- ❐ Bachelor's degree
- ❐ Master's degree
- ❐ Ph.D., M.D., or J.D.
- ❐ Completed vocational/technical/career program: ______________________
- ❐ Completed other post-high school training: ___________________________

39. Are you of Hispanic or Latino origin?

- ❐ No
- ❐ Yes

40. Which one or more of the following would you say is your race?

check ✓ all that apply

- ❐ American Indian or Alaska native
- ❐ Asian
- ❐ Black or African American
- ❐ Native Hawaiian or other Pacific Islander
- ❐ White
- ❐ Other: ____________________________

Finally, your feedback on the survey is valuable to us...

41. Are you (the person filling out this survey) the study participant named in the letter that came with this survey?

❐ No Comment: ______________________________

❐ Yes

42. Was this survey interesting to you?

Not at all interesting................................❐
Only a little interesting............................❐
Fairly interesting.....................................❐
Very interesting.......................................❐

43. Did you give accurate answers to the survey questions?

I answered <u>some</u> of the questions as accurately as I could .... ❐
I answered <u>most</u> of the questions as accurately as I could .... ❐
I answered <u>all</u> of the questions as accurately as I could................. ❐
Other: .... ❐

43a. If you chose "some," "most" or "other" above, please give details, or say which answers were not accurate.

____

____

____

____

44. Is there anything you'd like to say *about the survey questions, about your smoking or non-smoking,* or about anything else?

____

____

____

____

____

Please turn the page for final instructions. →

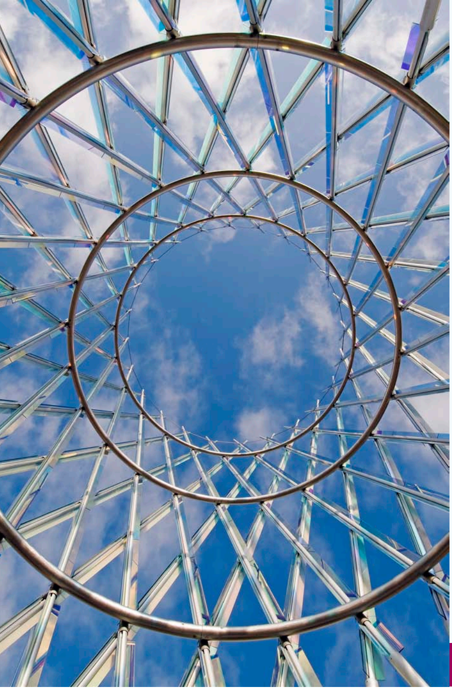

That's it!

You have completed your

SNAP SURVEY

Your input may help us find new ways to prevent cancer.

NOW, PLEASE PUT IT IN THE ENVELOPE AND MAIL IT BACK TO US.

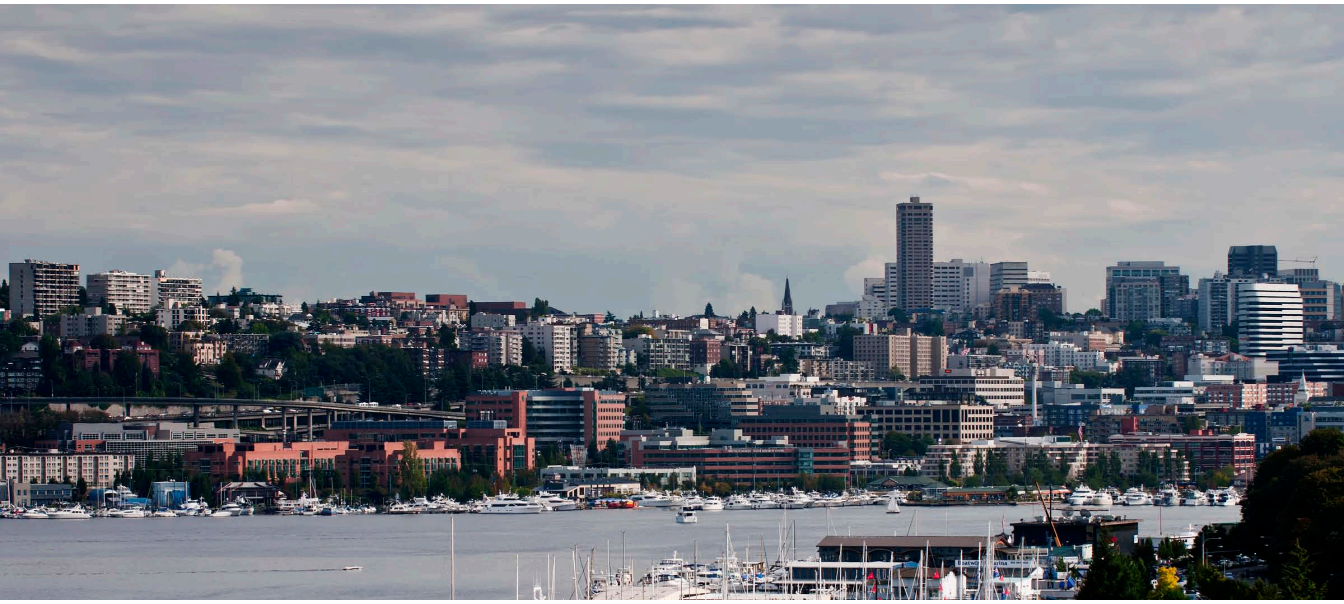

View towards Fred Hutchinson Cancer Research Center on Lake Union

THANK YOU SO MUCH!

yasr@fhcrc.org | 1.888.270.0620

Form # SNAP01-0713

Photo credits: Doug J. Scott (front cover) and Dean Forbes (back cover)
