# Supplementary material for: Does Effectiveness of Adolescent Smoking-Cessation Intervention Endure Into Young Adulthood? 7-Year Follow-Up Results from a Group-Randomized Trial
Source: PLoS One. 2016 Feb 1;11(2):e0146459. doi: 10.1371/journal.pone.0146459 (PMC4734743; doi:10.1371/journal.pone.0146459)
Supplement: S8 File — Intervention Impact on Progress Endpoints. (DOCX) [file pone.0146459.s008.docx]

**Appendix A. Intervention impact on sustained progress toward quitting.**

“Sustained” progress toward quitting was defined as progress from baseline to Plus 1 *that was followed by additional progress* from Plus 1 to Plus 7.

We evaluated three measures of sustained progress: (a) sustained reduction in level of smoking: ie, reductions in smoking frequency number of cigarettes smoked, and number of days smoked in the last month. [Tindle and Shiffman [45] found that conversion from daily smoking to less-than-daily smoking is a predictor of successful quitting.]; (b) sustained increase in length of quit attempts; and (c) sustained positive changes in readiness to change, both in Contemplation Ladder [46] and in stage of change (from the Transtheoretical Model [47]).

Results for intervention impact and these three measures of progress are shown below in Tables A1 (for all baseline smokers), A2 (for baseline daily smokers), and A3 (for baseline non-daily smokers). Footnotes for Tables A1, A2, and A3 are as follows:

* Δ = difference: percent or average in experimental high schools *minus* percent or average in control high schools. (However, whenever some high schools had no baseline smokers in the subgroup of interest, neither these high schools, *nor their pairs*, are included in the (matched-pair) permutation test, or in the computation of Δ reported here.) CI = confidence interval. *P* values (two-sided) were calculated using the group-randomized exact permutation test.

†*n* represents the number of valid responses, for females, males, and all, respectively. For some subgroups, *n* may differ among outcomes due to missing or incomplete responses.

§ Longest period of time without smoking in the last 12 months. Scale: 0 = Never tried to quit in the past year; 1 = “less than 24 hours”; 2 = “24 hours”; 3 = “2 – 7 days”; 4 = “8 – 30 days”; 5 =” between 1 month and 3 months”; 6 = “between 3 months and 6 months”; 7 = “six months or more”; 8 = Didn’t smoke at all in the past year.

¶  Readiness to quit: Scale: 1 = I am not thinking of quitting; 2 = I think I need to consider quitting someday; 3 = I think I should quit, but I’m not quite ready; 4 = I’m starting to think about how to change my smoking patterns; 5 = I’m taking actions now to quit smoking; 6 = Did not smoke in the past month (applicable for outcome value only) [46].

#  Stage of change. % who increase in stage of change. (1 = precontemplation; 2 = contemplation; 3 = preparation; 4 = action; 5 = maintenance [51])

†† Smoking frequency. Scale: 1 = “not at all”; 2 = “less than once a month”; 3 = “once a month or more, but less than once a week”; 4 = “once a week or more, but not daily”; 5 = “At least daily.”

‡‡ Number of days smoked in last month. Scale: 1 = “0 days”; 2 = “1 day”; 3 = “2-4 days”; 4 = “5-9 days”; 5 = “10-19 days”; 6 = “20-29 days”; 7 = “Every day”

§§ Average number of cigarettes smoked per day in the last 30 days.

[In order to measure change from Plus-1, the Plus-7 data item was converted to a categorical variable, with levels 0 (not a daily smoker or average smoked in last 30 days = 0); 1 (average at most 1 cig/day); 2 (average greater than one but ≤ 5); 3 (average greater than 5 but ≤ 10); 4 (average greater than 10 but ≤ 20); 5 (average >20).]

**Table A1.** Intervention impact on sustained progress toward quitting, among all baseline smokers.

| **Progress endpoints** | **Females** | | | |  | **Males** | | | |  | **All participants** | | | |  |
| --- | --- | --- | --- | --- | --- | --- | --- | --- | --- | --- | --- | --- | --- | --- | --- |
|  | **Control** | **Experi-mental** | **Δ, % (95% CI)** | ***P*** |  | **Control** | **Experi-mental** | **Δ, % (95% CI)** | ***P*** |  | **Control** | **Experi-mental** | **Δ, % (95% CI)** | ***P*** | |
|  |  |  |  |  |  |  |  |  |  |  |  |  |  |  |  |
| *Sustained reduction in level of smoking:* |  |  |  |  |  |  |  |  |  |  |  |  |  |  |  |
| In smoking frequency †† (*n* = 818, 849, 1667) † | 32.1 | 31.5 | -0.6 (-8.6, 7.5) | .87 |  | 24.6 | 29.2 | 4.6 (-2.6, 10.8) | .18 |  | 28.0 | 30.3 | 2.3 (-2.8, 7.0) | .35 |  |
| In number of cigs smoked per day §§. (*n* = 815, 844, 1659) | 48.8 | 54.4 | 5.6 (-1.3, 12.6) | .11 |  | 46.9 | 52.6 | 5.7 (-2.4, 13.0) | .15 |  | 47.5 | 53.6 | 6.1 (0.8, 11.4) | .03 |  |
| In number of days smoked in the past 30 days ‡‡ (*n* = 816, 842, 1658) | 32.6 | 33.8 | 1.2 (-5.5, 8.2) | .71 |  | 24.4 | 32.8 | 8.4 (1.3, 14.7) | .024 |  | 28.3 | 33.4 | 5.1 (-0.02, 9.9) | .051 |  |
| *Sustained increase in length of longest quit attempt:* § (*n* = 774, 821, 1595) | 26.2 | 24.9 | -1.3 (-8.5, 6.4) | .74 |  | 18.0 | 23.8 | 5.8 (.02, 11.2) | .049 |  | 22.1 | 24.4 | 2.3 (-2.6, 7.2) | .34 |  |
| *Sustained increase in readiness to quit:* |  |  |  |  |  |  |  |  |  |  |  |  |  |  |  |
| In Contemplation Ladder: ¶ (*n* = 756, 792, 1548) | 39.0 | 39.0 | 0.0 (-7.1, 7.7) | .99 |  | 31.9 | 37.1 | 5.2 (-3.5, 13.0) | .21 |  | 35.1 | 38.0 | 2.9 (-1.9, 7.2) | .21 |  |
| In stage of change, % # (*n* = 758, 796, 1554) | 29.2 | 31.1 | 1.9 (-6.7, 10.4) | .65 |  | 25.0 | 26.5 | 1.5 (-5.6, 7.7) | .66 |  | 26.9 | 28.7 | 1.8 (-3.6, 6.8) | .48 |  |

**Table A2.**  Intervention impact on sustained progress toward quitting among baseline daily smokers.

| **Progress endpoints** | **Females** | | | |  | **Males** | | | |  | **All participants** | | | |  |
| --- | --- | --- | --- | --- | --- | --- | --- | --- | --- | --- | --- | --- | --- | --- | --- |
|  | **Control** | **Experi-mental** | **Δ, % (95% CI)** | ***P*** |  | **Control** | **Experi-mental** | **Δ, % (95% CI)** | ***P*** |  | **Control** | **Experi-mental** | **Δ, % (95% CI)** | ***P*** | |
|  |  |  |  |  |  |  |  |  |  |  |  |  |  |  |  |
| *Sustained reduction in level of smoking:* |  |  |  |  |  |  |  |  |  |  |  |  |  |  |  |
| In smoking frequency †† (*n* = 316, 302, 618) | 16.6 | 19.4 | 1.6 (-7.6, 10.9) | .73 |  | 16.2 | 20.6 | 5.1 (-5.4, 14.4) | .30 |  | 16.4 | 20.0 | 3.6 (-3.1, 9.7) | .26 |  |
| In number of cigs smoked per day §§. (*n* = 317, 300, 617) | 19.7 | 31.5 | 10.4 (-0.5, 23.2) | .06 |  | 24.8 | 33.3 | 8.3 (-2.9, 18.0) | .13 |  | 22.2 | 32.4 | 10.2 (3.0, 17.5) | .007 |  |
| In number of days smoked in the past 30 days ‡‡ (*n* = 316, 300, 616) | 20.4 | 20.1 | -0.9 (-11.1, 9.2) | .86 |  | 15.6 | 21.4 | 5.8 (-4.3, 14.7) | .25 |  | 18.1 | 20.7 | 2.7 (-4.9, 9.4) | .45 |  |
| *Sustained increase in length of longest quit attempts:* § *(n* = 308, 298, 606) | 19.5 | 18.9 | -0.6 (-10.0, 9.9) | .90 |  | 14.4 | 16.4 | 1.8 (-6.4, 10.4) | .64 |  | 17.0 | 17.6 | 0.6 (-6.2, 8.2) | .86 |  |
| *Sustained increase in readiness to quit:* |  |  |  |  |  |  |  |  |  |  |  |  |  |  |  |
| In Contemplation Ladder: ¶ (*n* = 300, 293, 593) | 24.5 | 29.9 | 3.9 (-4.2, 11.2) | .33 |  | 23.7 | 27.8 | 5.1 (-6.3, 14.6) | .35 |  | 24.1 | 28.9 | 4.8 (-0.7, 9.6) | .08 |  |
| In stage of change, % # (*n* = 310, 291, 601) | 15.0 | 20.9 | 5.5 (-5.0, 15.7) | .28 |  | 17.2 | 15.9 | -0.9 (-10.9, 8.1) | .84 |  | 16.0 | 18.4 | 2.4 (-3.7, 7.8) | .40 |  |

**Table A3..** Intervention impact on sustained progress toward quitting among baseline non-daily smokers.

| **Progress endpoints** | **Females** | | | |  | **Males** | | | |  | **All participants** | | | |  |
| --- | --- | --- | --- | --- | --- | --- | --- | --- | --- | --- | --- | --- | --- | --- | --- |
|  | **Control** | **Experi-mental** | **Δ, % (95% CI)** | ***P*** |  | **Control** | **Experi-mental** | **Δ, % (95% CI)** | ***P*** |  | **Control** | **Experi-mental** | **Δ, % (95% CI)** | ***P*** | |
|  |  |  |  |  |  |  |  |  |  |  |  |  |  |  |  |
| *Sustained reduction in level of smoking:* |  |  |  |  |  |  |  |  |  |  |  |  |  |  |  |
| In smoking frequency †† (*n* = 502, 547, 1049) | 41.4 | 39.4 | -1.9 (-13.6, 10.0) | .73 |  | 29.2 | 33.5 | 4.3 (-4.2, 12.0) | .30 |  | 34.8 | 36.4 | 1.6 (-4.7, 7.7) | .61 |  |
| In number of cigs smoked per day §§. (*n* = 498, 544, 1042) | 66.4 | 69.1 | 2.7 (-7.2, 12.9) | .58 |  | 59.0 | 63.2 | 4.2 (-6.3, 14.4) | .40 |  | 62.4 | 66.1 | 3.7 (-3.4, 11.0) | .29 |  |
| In number of days smoked in the past 30 days ‡‡ (*n* = 500, 542, 1042) | 40.2 | 42.7 | 2.5 (-6.4, 12.1) | .57 |  | 29.3 | 39.1 | 9.8 (1.3, 17.7) | .03 |  | 34.4 | 40.9 | 6.5 (-0.1, 13.3) | .053 |  |
| *Sustained increase in length of longest quit attempts:* § (*n* = 466, 523, 989) | 30.6 | 28.9 | -1.7 (-11.4, 8.6) | .72 |  | 20.5 | 28.1 | 8.1 (-0.4, 16.0) | .06 |  | 25.2 | 28.5 | 3.3 (-2.7, 9.4) | .26 |  |
| *Sustained increase in readiness to quit:* |  |  |  |  |  |  |  |  |  |  |  |  |  |  |  |
| In Contemplation Ladder: ¶ (*n* = 456, 499, 955) | 47.8 | 45.4 | -2.4 (-13.0, 9.0) | .66 |  | 36.8 | 42.0 | 5.3 (-6.1, 16.0) | .34 |  | 42.0 | 43.7 | 1.7 (-5.4, 8.7) | .62 |  |
| In stage of change, % # (*n* = 448, 505, 953) | 38.7 | 38.1 | -0.6 (-11.9, 10.6) | .91 |  | 29.5 | 32.3 | 2.8 (-6.4, 11.4) | .52 |  | 33.7 | 35.1 | 1.5 (-5.8, 8.5) | .67 |  |
